# Dynamic Combinatorial Optimization of *In Vitro* and *In Vivo* Heparin Antidotes

**DOI:** 10.1021/acs.jmedchem.1c02054

**Published:** 2022-03-02

**Authors:** Daniel Carbajo, Yolanda Pérez, Marta Guerra-Rebollo, Eva Prats, Jordi Bujons, Ignacio Alfonso

**Affiliations:** ^†^Department of Biological Chemistry and ^‡^NMR Facility, Institute for Advanced Chemistry of Catalonia (IQAC-CSIC), Jordi Girona 18-26, 08034 Barcelona, Spain; § Grup d’Enginyeria de Materials (Gemat), Institut Químic de Sarriá (IQS), Universitat Ramon Llull (URL), Via Augusta 390, 08017 Barcelona, Spain; ∥ Research and Development Center (CID-CSIC), Jordi Girona 18-26, 08034 Barcelona, Spain

## Abstract

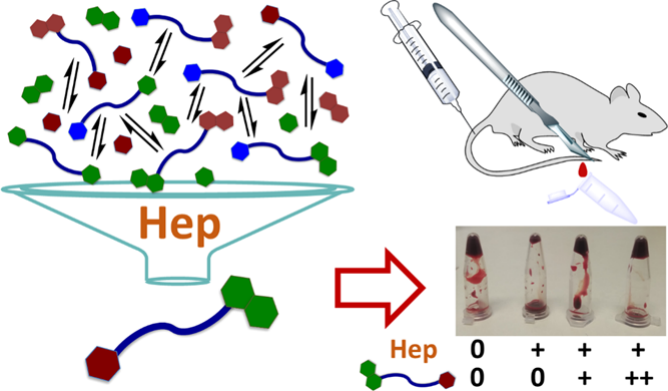

Heparin-like macromolecules are widely used in clinics as anticoagulant, antiviral, and anticancer drugs. However, the search of heparin antidotes based on small synthetic molecules to control blood coagulation still remains a challenging task due to the physicochemical properties of this anionic polysaccharide. Here, we use a dynamic combinatorial chemistry approach to optimize heparin binders with submicromolar affinity. The recognition of heparin by the most amplified members of the dynamic library has been studied with different experimental (SPR, fluorescence, NMR) and theoretical approaches, rendering a detailed interaction model. The enzymatic assays with selected library members confirm the correlation between the dynamic covalent screening and the *in vitro* heparin inhibition. Moreover, both *ex vivo* and *in vivo* blood coagulation assays with mice show that the optimized molecules are potent antidotes with potential use as heparin reversal drugs. Overall, these results underscore the power of dynamic combinatorial chemistry targeting complex and elusive biopolymers.

## Introduction

**Figure 1:**
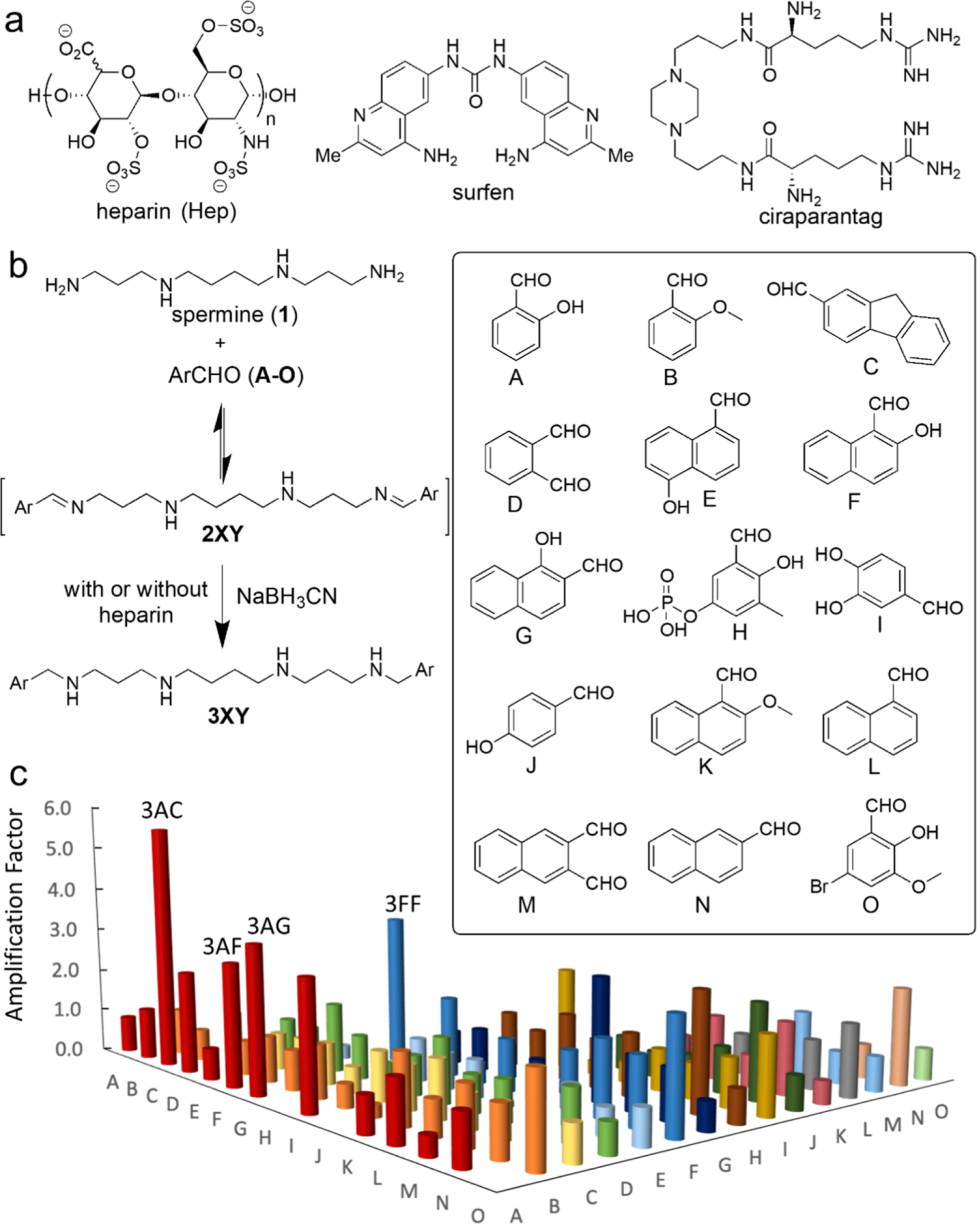
(a) Chemical structures of heparin (Hep), surfen, and ciraparantag. (b) Scheme of the DCC reductive amination reaction with the corresponding chemical structures of the aromatic aldehydes used in this work. (c) Plot of the averaged amplification factors obtained for every member of the DCL.

Heparin (Hep, Figure 1a) is a highly anionic glycosaminoglycan (GAG)^1^ extensively used in clinics, mainly as an anticoagulant^2,3^ and also as an antiviral^4,5^ and anticancer^6,7^ agent. For a safer usage of Hep, the accessibility to a family of efficient reversal agents is desirable, especially for cases of overdose, life-threatening bleeding, or urgent high-risk surgery.^8−10^ However, the number of antidotes for heparin-type drugs is quite limited: the one mostly used is protamine,^11^ a small arginine-rich nuclear protein. Recent reports on alternative macromolecules to reverse heparin drugs include a polymeric polycationic dendrimer (UHRA),^12^ a monoclonal antibody (idarucizumab),^13^ a modified version of the recombinant human coagulation factor Xa (andexanet alpha),^14^ as well as peptides^15^ and peptidomimetics^16^ mimicking protamine. Other representative Hep-binding molecular scaffolds are self-assembled cationic amphiphiles^17^ or peptide dendrimers.^18,19^ Their affinity toward Hep varies from the low micromolar to the high nanomolar ranges,^20^ being both idarucizumab and andexanet alpha currently approved reversal agents of different anticoagulants.^21^ However, considering the general drawbacks of using high-molecular-weight drugs,^22^ the search for alternative small molecules able to revert the anticoagulation effects of heparin is a hot topic in current research.^20,23,24^ Among these small molecules, probably surfen^25^ and ciraparantag^26,27^ (also named PER977) are the most representative examples (Figure 1a). The bis(quinolyl)urea surfen is an approved drug originally used as excipient for the production of depot insulin that has been recently identified as a heparan sulfate binder.^25,28^ This has led to the investigation of surfen derivatives as potential inhibitor of the GAG-protein interactions in different biological processes.^28^ However, the oncogenicity of surfen at the doses needed to observe effects on the coagulation rate has precluded further development in this specific field.^29^ On the contrary, the cationic synthetic ciraparantag molecule is currently in clinical trials as an inhibitor of different anticoagulants.^30^ Although its actual mechanism of action is still unclear, ciraparantag is proposed to bind heparin through H-bonding and ionic interactions.^31^ The lack of detailed structural information is a general loophole in the supramolecular investigations related to heparin and other GAGs, probably due to the chemical peculiarities of these macromolecules.^32^ Thus, heparin is a long linear highly sulfated polysaccharide with some heterogeneity in the repeating units and a large polydispersity when isolated from natural sources (unfractioned or low-molecular-weight heparin used in clinics).^33^ Moreover, the highly anionic charge density and polar nature of Hep make its molecular recognition extremely difficult, especially in highly solvating aqueous ionic media.^34,35^ This chemical complexity is also accompanied by high conformational flexibility in solution,^36^ complicating to establish a preferred three-dimensional structure by either experimental (X-ray diffraction, NMR) or theoretical (molecular modeling) approach.^37^ Overall, this makes the rational design of ligands against Hep an extremely challenging task.

Paradoxically, the structural heterogeneity and the lack of evident binding pockets make Hep a good candidate as a template in dynamic combinatorial chemistry.^38−41^ Dynamic covalent chemistry does not require detailed knowledge of the structural characteristics of the target since the target itself selects and amplifies the most efficient binders from a dynamic combinatorial library (DCL) of potential ligands.^42−47^ We have recently demonstrated this approach as a proof of concept with a small and simple dynamic library of imines that, after reduction, led to the identification of a heparin ligand with an affinity in the micromolar range.^48^ In this work, we expand the molecular diversity of the dynamic library, thus generating a much wider molecular recognition space. This library better covers the structural basis for the efficient recognition of heparin, identifying more potent binders with promising applications as potential drugs,^49^ as shown by *in vitro*, *ex vivo*, and *in vivo* assays. Moreover, the larger structural diversity allows the validation of the dynamic combinatorial chemistry approach to the development of potent hits, by correlating the response in the dynamic covalent screening with the activity in blood coagulation.

## Results and Discussion

### Dynamic Combinatorial Screening Experiments

In the design of the building blocks for the dynamic covalent screening experiments, we followed a rational approach. The highly anionic Hep target can be counterbalanced by the positively charged nitrogen atoms of spermine (**1** in Figure 1b) in aqueous media at neutral pH.^50,51^ On the other hand, we selected a family of aromatic aldehydes (A–O, Figure 1b) that could potentially establish CH−π interactions^52,53^ with the carbohydrate rings of Hep. We included different structural variables in the aromatics of A–O: H-bonding groups, extended aromatic surfaces, and dialdehydes possibly leading to oligomers. We also considered isomers with different geometrical dispositions of the groups (A vs J, L vs N, and E vs F vs G). In an aqueous environment, the combination of **1** and A–O leads to the formation of imines **2XY**, while the spermine structure also favors transient cyclic aminals^54,55^ and additional isomers. Overall, this would possibly yield more than 120 virtual library members. The dynamic imine mixture responds to the presence of Hep as a template, with the potential binders being stabilized and accordingly amplified by the GAG. *In situ* reduction leads to the corresponding amines (**3XY**), which can be identified and quantified. The large size of the whole DCL with high structural similarity of the members made it impossible to analyze the whole mixture due to chromatographic peak overlapping and molecular weight degeneration. Therefore, we deconvoluted the mixture into 28 sublibraries (Table S1) to allow full crossed comparison but avoid molecules with the same mass. Each sublibrary was incubated with Hep and reduced *in situ* to the corresponding amines. The UPLC-MS analysis of the reaction mixtures allowed the identification and quantification of the members of the library. The area of the corresponding UPLC-MS peaks for each compound (*A*_T_) was compared with the ones obtained in the same reactions performed in the absence (*A*_0_) of Hep, yielding the amplification factor (AF = *A*_T_/*A*_0_) for each compound. The experiments were performed in triplicate, and the AFs for those members appearing in several sublibraries were averaged. The plot of the final amplification factors is shown in Figure 1c (Table S2), demonstrating that several members of the library are strongly amplified by the presence of Hep.

The AFs here presented are an average of those measured in different DCLs, and thus, they can be only used with comparative purposes. Moreover, those AFs coming from barely detected peaks in the control reactions (absence of Hep) were double-checked and, in some cases, not considered since they are artificially high. The results show interesting trends. First, the ligand previously identified in a smaller DCL (3AL)^48^ is only slightly favored in this larger system with a broader structural diversity. This suggests that the members with higher AFs in the current DCL might be stronger binders to Hep. Moreover, there are common structural features in the amplified molecules. All of them bear at least one phenol-type residue with the hydroxyl in *ortho* position and one large aromatic ring (naphthol or fluorene). The most amplified members, 3AC and 3FF, are worth mentioning in this regard. The molecule 3FF can be considered as the symmetric combination of the two structural features (large aromatic and ortho phenol-type residues). On the other hand, 3AC is a nonsymmetric structure that would have been difficult to predict from the previous structural data, but it shows the largest AF of the series. Selected ligands were synthesized at a preparative scale for further binding and biochemical assays.

### Binding of the Amplified Ligands to the Heparin Target

We used surface plasmon resonance (SPR) on heparin-functionalized chips to determine the corresponding apparent dissociation constant of selected molecules (*K*_D_^app^, Table 1 and Figures S10–S13).^56^ Although the obtained *K*_D_^app^ assumes an oversimplification of the actual binding mode (see below), it is highly convenient to compare the affinity of the different molecules to Hep. The SPR-determined *K*_D_^app^ for 3AA and 3AL (entries 1 and 2 in Table 1) are consistent with those previously obtained by ITC,^48^ supporting our simplified approximation. The symmetric molecules 3AA (entry 1) and 3BB (entry 5) show the weakest heparin binding of the series, in good agreement with the absence of amplification in the DCL assays. Other molecules containing an *ortho* phenol and a large aromatic ring (3AL, 3AF, and 3AG) display moderate amplification factors (AF ≈ 1.5–3.0, Figure 1C) and a *K*_D_^app^ in the low micromolar range (entries 2–4). Quite remarkably, those showing the highest AFs (3FF and 3AC) render submicromolar affinity to Hep by SPR (entries 6 and 7 in Table 1). Therefore, there is a good correlation between the AFs and the binding affinities, which spread over almost 2 orders of magnitude for molecules showing subtle structural variations. Remarkably, two of the identified hits (3AC and 3FF) show higher affinity to Hep than ciraparantag (entry 8).

**Table 1:** Apparent Dissociation Constants, *K*_D_^app^ (μM), for the Interaction of Selected Molecules with Hep^a^

		*K*_D_^app^ (μM)	
entry	binder	SPR	ITC/fluor
1	3AA	21.5 ± 5.7	22.5 ± 9.9 (ITC)^b^
2	3AL	1.89 ± 0.13	1.3 ± 0.4 (ITC)^b^
3	3AF	2.62 ± 0.20	n.d.^c^
4	3AG	1.86 ± 0.12	n.d.^c^
5	3BB	33.7 ± 8.4	n.d.^c^
6	3FF	0.797 ± 0.240	n.d.^c^
7	3AC	0.567 ± 0.013	0.50 ± 0.09 (fluor.)^d^
8	ciraparantag	1.53 ± 0.12	n.d.^c^

a All measurements were performed at pH 7.5 in 25 mM Tris as the working buffer.

b Measured by ITC, see ref (48).

c n.d. means not determined.

d Estimated from the equilibrium constants obtained by fitting the fluorescence emission titration to the model displayed in Figure 2b (see the Supporting Information for details).

Fortunately, many binders are fluorescent and their emission spectra are perturbed by the presence of Hep (Figures S14, S21, and S29–S31), allowing us to obtain additional information about the supramolecular complexes in solution. For instance, the emission spectrum of 3AC is strongly reduced upon the addition of up to two equivalents of the Hep disaccharide repeating units, slightly recovering upon additional Hep (Figure 2a). Despite the complexity of the titration isotherm, this could be fitted to a reasonable binding mode considering the disaccharide repeating unit of Hep as the binding motif (inset in Figure 2a). The proposed binding model is depicted in Figure 2b: 3AC forms a stable species with one ligand per disaccharide repeating unit ([3AC-Hep] complex), which evolves to the [3AC-Hep_2_] complex upon additional Hep, or to a less specific species [3AC_2_-Hep] in the presence of excess 3AC. This detailed binding model nicely explains the observed fluorescence emission results: the proximity of the chromophores in the [3AC-Hep] and [3AC_2_-Hep] complexes accounts for the fluorescence quenching that is partially recovered when the 3AC bound molecules are spread along the Hep surface in the [3AC-Hep_2_] species. A putative *K*_D_^app^ = 0.5 μM can be estimated from the fluorescence binding data (Table 1, entry 7, see the Supporting Information for details), which is in very good agreement with the SPR results.

**Figure 2:**
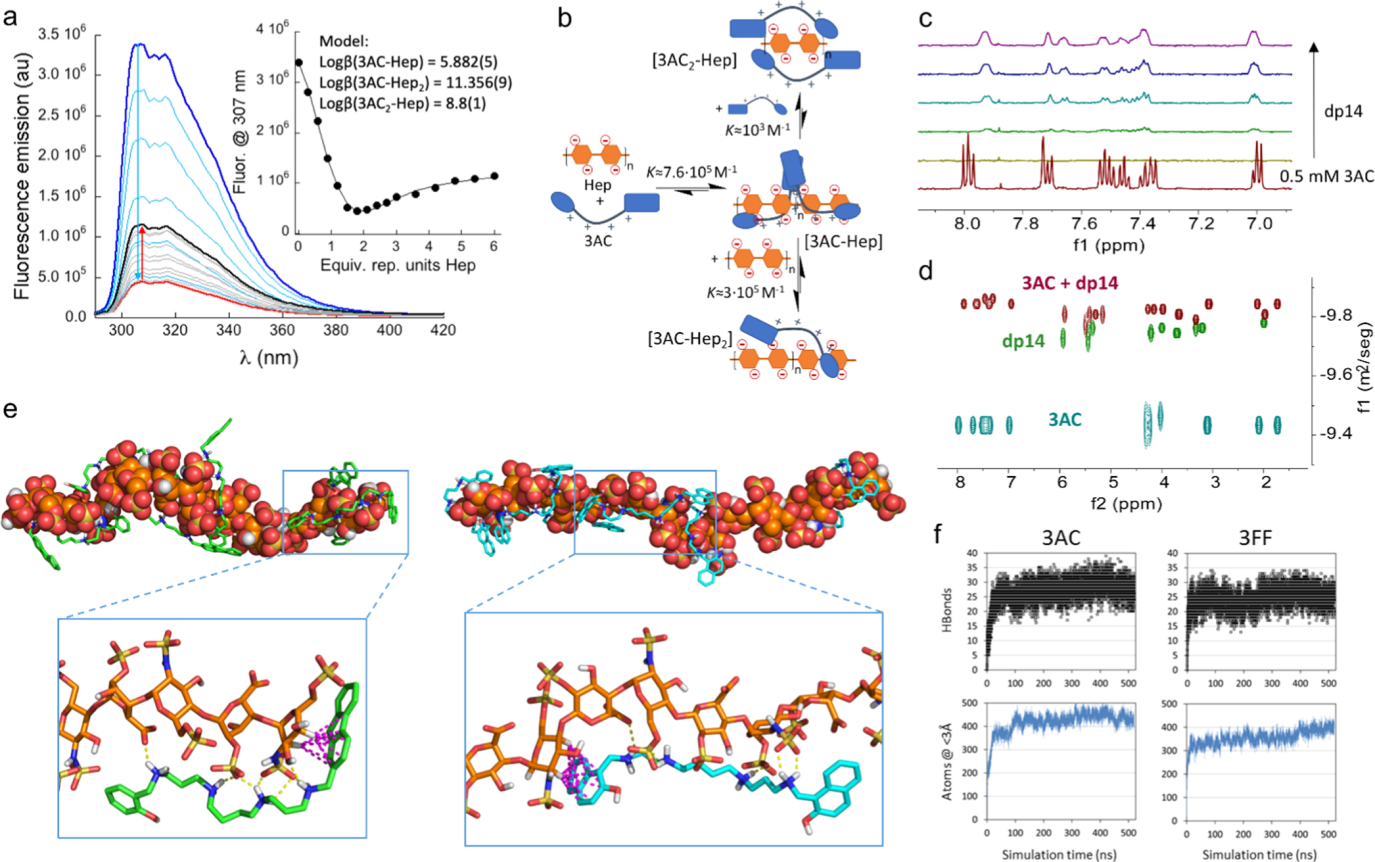
(a) Fluorescence emission spectra of 3AC (10 μM in 1 mM Bis-Tris buffer at pH 7.5) upon titration with heparin. The inset shows the variation of the emission fluorescence at 307 nm against the equivalents of repeating units of Hep (with the fitting to the proposed binding model). (b) Schematic representation of the binding model between 3AC and Hep, with the corresponding stepwise equilibrium constants shown for each step. (c) Stacked partial ^1^H NMR spectra of 0.5 mM 3AC (5 mM Tris-d11, pH* 7.0, 303 K, 50 mM NaCl) upon increasing addition of dp14 heparin (concentration of dp14 related to the disaccharide repeating unit, from 0.5 to 2.5 mM). (d) Superimposed DOSY NMR spectra of 3AC (cyan), dp14 (green), and [3AC-dp14] complex (brown). The diffusion axis is in the logarithmic scale. (e) Representative snapshots from simulations of heparin (orange C-atoms) plus 6 ligand molecules: ligand = **3AC** (green C-atoms, left) or **3FF** (cyan C-atoms, right). Insets show the interactions between **3AC** or **3FF** and heparin (H-bonds/salt bridges: yellow dashed lines; CH−π interactions: magenta dashed lines). (f) Results from a representative competence simulation of one molecule of dp16 heparin plus 6 molecules of **3AC** and 6 molecules of **3FF**. Graphs represent the total number of hydrogen bonds between ligands and heparin and the total number of ligand atoms within a distance of 3 Å from heparin vs simulation time. Average values for the last 250 ns: **3AC**, HBonds = 28.4 ± 3.0, Atoms = 441 ± 22; **3FF**, HBonds = 25.6 ± 2.9, Atoms = 373 ± 26.

We also studied the 3AC-Hep binding by ^1^H NMR spectroscopy, in this case using a shorter heparin oligomer, dp14, which on average contains seven disaccharide repeating units. The titration of 3AC with dp14 (Figure 2c) shows two different phases. At the beginning of the titration (high 3AC: dp14 ratio), we observed a large broadening of the 3AC spectrum leading to the disappearance of some proton signals. Under these conditions, the [3AC_2_-dp14] and [3AC-dp14] complexes prevail (Figure S15), which leads to an electrostatic charge compensation of dp14 favoring the aggregation of the species in solution. The addition of more equivalents of Hep produces the complete formation of [3AC-dp14_2_] species, which is clearly visible in the ^1^H NMR spectrum by the growth of broad signals at different chemical shifts from those of 3AC alone (Figures 2c and S16). The NMR titration experiment strongly correlates with the binding mode inferred from the fluorescence titration, while the slow exchange observed in the chemical shift NMR timescale is consistent with the very strong binding quantified by SPR and fluorescence spectroscopy. Complementary titration experiments of a dp14 sample with 3AC confirm these conclusions. Thus, the addition of a substoichiometric amount of 3AC produces a shift in several signals of dp14 as clearly observed in the corresponding ^1^H-^1^H TOCSY (Figure S18), ^1^H-^13^C HSQC (Figure S19), and ^1^H-^1^H COSY (Figure S20) experiments. Most of the signals from the carbohydrate backbone shift upfield, presumably due to the anisotropy effect of the aromatic rings of 3AC in the CH−π interactions. Remarkably, some anomeric proton signals of dp14 split upon 3AC binding, suggesting slow exchange between different species in solution, namely, either free and bound or structurally different complexes. Besides, diffusion ordered spectroscopy (DOSY) NMR experiments of the systems are very clarifying (Figures 2d, S17, and Table S7): the translational self-diffusion rate (*D*) of the [3AC-dp14] complex, observed in the proton signals from both components, is lower than for dp14 and much lower than for 3AC alone, further supporting the strong 3AC-dp14 interaction forming bigger noncovalent species.

On the other hand, and unlike 3AC (Figure S9), 3FF has a high tendency to aggregate in an aqueous medium at a neutral pH, as reflected by NMR (Figures S2–S5 and Table S7), fluorescence spectroscopy (Figures S6 and S7), and dynamic light scattering (DLS, Figure S8 and Table S3). This aggregation complicates the quantitative analysis of the corresponding solution binding experiments with 3FF. Nevertheless, the titration of 3FF with different forms of Hep shows a strong 3FF-Hep interaction, which can be qualitatively monitored by fluorescence spectroscopy (Figure S21), DLS (Figures S22 and S23 and Table S6), 1D ^1^H NMR titration (Figures S24–S26), DOSY (Figure S27 and Table S7), and ^1^H-^13^C HSQC (Figure S28) experiments. Moreover, the 3FF-Hep supramolecular species displayed a somehow complex behavior that evolved with time, suggesting the coexistence of binding and aggregation dynamic processes.

To illustrate the binding of the amplified molecules to Hep, we performed molecular dynamics (MD) simulations of short Hep oligomers, dp16, containing eight disaccharide repeating units with six molecules of either 3AC or 3FF. The inspection of the MD snapshots identifies the binding motifs as the proposed salt bridges and H-bonds with the polyammonium moiety, in addition to the CH−π sugar-aromatic interactions (Figures 2e and S34). Additional MD simulations with six 3FF and six 3AC molecules competing for dp16 clearly show the stronger binding of 3AC since 3AC establishes a higher number of H-bonds and close contacts than 3FF, on average (Figures 2f, S35, and Video S1). In similar MD assays, 3AC also showed to be a better Hep-binder than 3AL (Figure S36 and Video S2), the molecule amplified in our previous Hep-templated DCL.^48^ Thus, the molecular models strongly parallel with the obtained experimental results.

### *In Vitro* Inhibition of Heparin Activity

**Figure 3:**
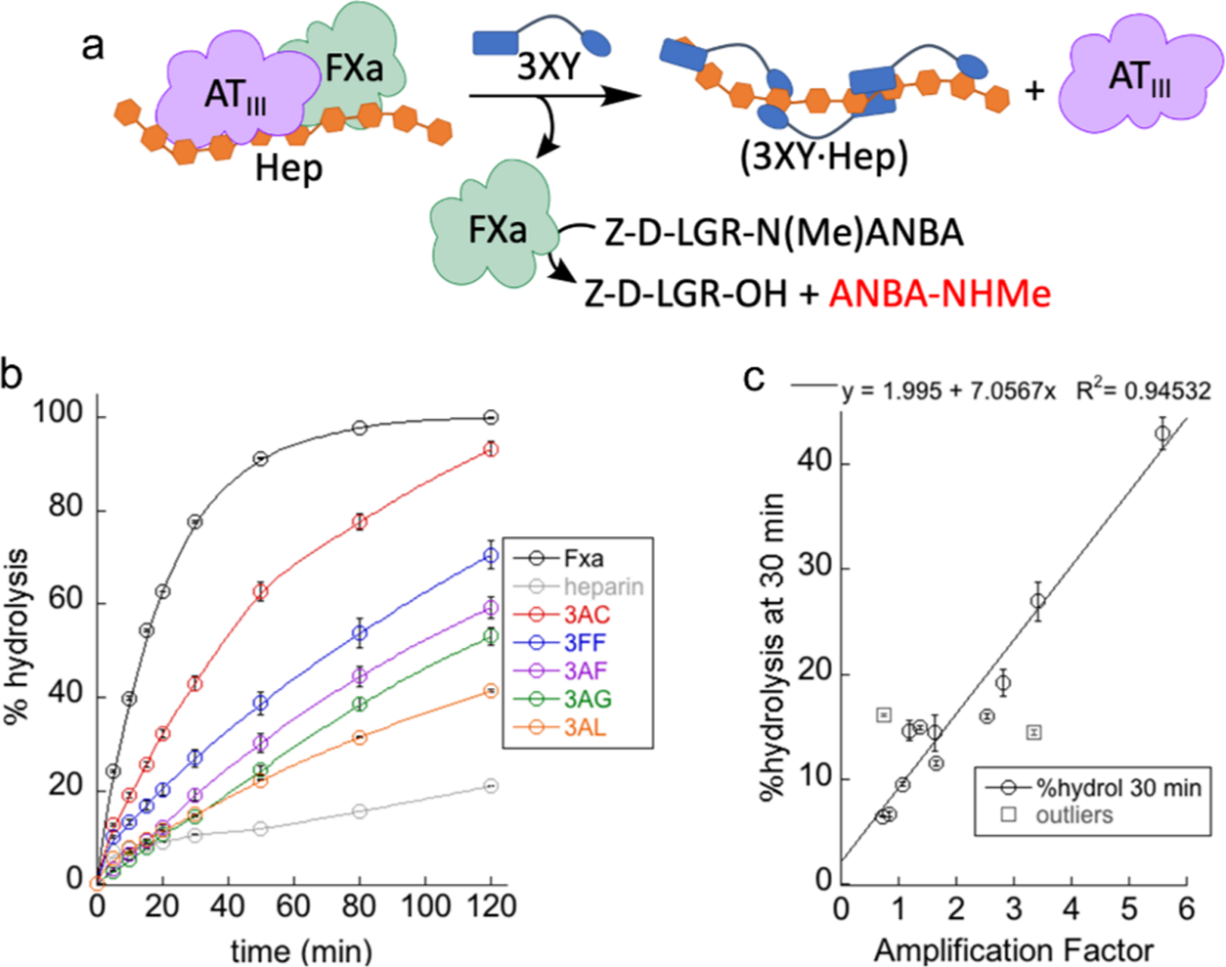
(a) Schematic representation of the *in vitro* enzymatic assay used to test the reversal effect of the heparin ligands. (b) Plot of the time evolution of the hydrolysis of the peptide substrate (485 μM) under different conditions: all traces (0.085 μg/mL of FXa and 0.02 IU/mL of AT_III_), gray and colored traces (0.1 IU/mL of Hep), and colored traces (1 μM of each ligand). (c) Plot of the percent hydrolysis at 30 min vs the corresponding AF for selected ligands.

The effect of the binders as Hep antidotes can be tested *in vitro* using an enzymatic reaction related to the blood coagulation process (Figure 3a).^57^ This test employs human recombinant coagulation factor Xa (FXa) and antithrombin III (AT_III_), with the synthetic peptide Z-D-LGR-N(Me)ANBA as the substrate of the FXa protease to mimic the prothrombin-to-thrombin transformation that triggers blood coagulation. Heparin forms a ternary complex with AT_III_ and FXa with low hydrolytic activity, as a model for the anticoagulation effect of Hep. The addition of an efficient Hep binder dissociates the [FXa-Hep-AT_III_] complex, thus releasing the active form of FXa that cleaves the substrate. The peptide hydrolysis can be readily monitored by analytical HPLC thanks to the ANBA chromophore (Figure 3b), as an accurate measurement of the reaction kinetics. We have previously used this method to demonstrate the inhibitory activity of 3AL.^48^ Here, we slightly modified the experimental conditions to adapt the assay to the higher potency of the new molecules.

Figure 3b shows the kinetic of peptide hydrolysis under different reaction conditions. In the absence of Hep (black trace), FXa exhibits the highest activity, which is clearly inhibited when Hep is present (0.1 IU/mL, gray). In parallel experiments, the addition of equal concentration (1 μM) of each of the binders produces a different level of recovery of the FXa activity. Thus, the *in vitro* potency as Hep antidote grows in the series 3AL ≤ 3AG < 3AF < 3FF < 3AC. This trend nicely correlates with the AF values observed in the DCC experiments and the binding data shown in Table 1. Actually, we tested up to 13 selected molecules from the library in the enzymatic assay (Figure S32), and their ability to reverse the Hep inhibitory effect is linearly proportional to the AFs observed in the DCL screening (Figure 3c). Only those ligands bearing the aromatic naphthol from aldehyde G (3AG and 3GG) can be considered outliers, probably due to their lower solubility in water. This excellent correlation means a definitive validation of our dynamic combinatorial screening protocol. Also in the *in vitro* assays, 3FF and 3AC are the most active Hep antidotes. Moreover, the *in vitro* comparison of these two molecules with the drug ciraparantag (Figure S33) shows that 3FF performs very similarly, while 3AC is a more potent Hep reversal agent.

### Reversing the Anticoagulant Activity of Heparin

**Figure 4:**
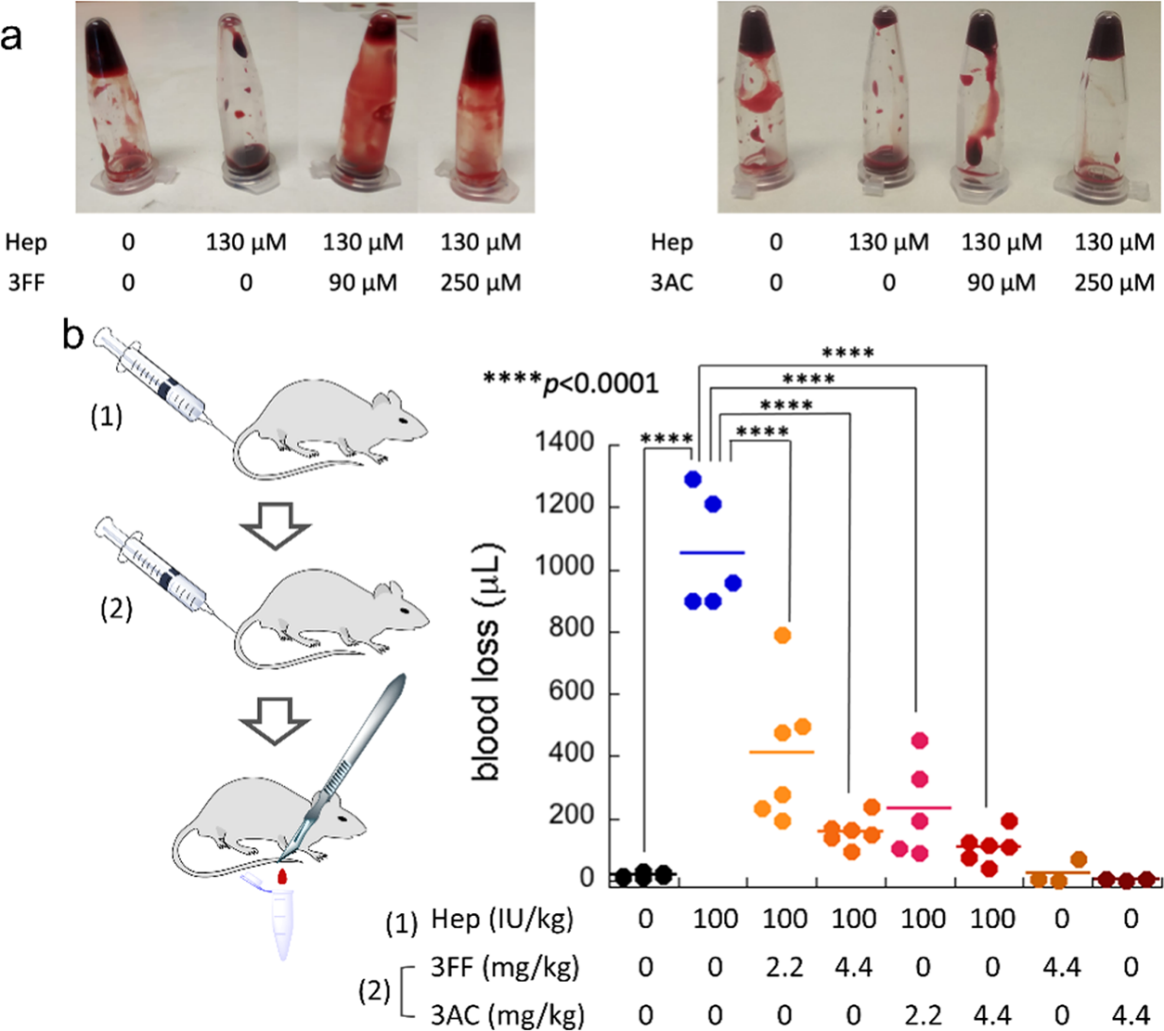
(a) *Ex vivo* coagulation test with freshly extracted mouse blood using Hep in combination with 3FF and 3AC antidotes. Molar concentrations of Hep are related to the disaccharide repeating units. (b) *In vivo* tail transection assay performed in mice to test the reversal of Hep activity by 3AC or 3FF. Statistically different (ANOVA) results (*p* < 0.0001) are labeled.

To check the efficacy of the antidotes in a more representative environment, we decided to test 3FF and 3AC in real blood coagulation assays. Thus, we monitored the coagulation of freshly extracted mouse blood in the absence or presence of Hep, and upon addition of different concentrations of 3FF and 3AC. By simply turning around the vials after 15 min (Figure 4a), the differences in their viscosity are evident: the control samples are completely coagulated, Hep-containing vials remain liquid (the blood flows down), while 3FF and 3AC are able to revert the anticoagulation effect of Hep in a dose–response manner. Actually, at least qualitatively, 3AC seems to be a better antidote than 3FF in this simple *ex vivo* experiment since a more robust clot was observed at equal doses. The microscopic morphology of the samples was analyzed by scanning electron microscopy (SEM, Figure S37) to better understand the macroscopic behavior. Thus, while the heparinized blood sample shows clean blood cells, the cloths formed in the control sample (without Hep) display aggregated cells connected by fibrin fibers. The samples containing both Hep and the binders (3FF or 3AC) show an aggregation of the cells that is similar to the control, with a denser fibrous network in the case of 3AC. It is also noticeable that the shape and size of the cells remain unaffected by the drugs.

Encouraged by these results, we tested 3FF and 3AC in an *in vivo* coagulation model.^23^ Moreover, both 3FF and 3AC show low cytotoxicity (Figure S38) at the highest concentrations used for the *in vivo* assays. We thus performed the tail transection assay with mice after consecutive injection of Hep, and the drugs at two different doses each (Figure 4b). The control groups injected with saline or Hep, and those corresponding to just the drugs were also included. The results clearly show that both 3FF and 3AC are efficient antidotes of Hep also *in vivo*, strongly reducing the bleeding of heparinized mice. Remarkably, the amount of blood loss is dose responsive for both drugs, and 3AC is a more potent reversal agent than 3FF. At the highest doses of 3FF/3AC here used, the blood loss of heparinized mice was reversed to values very similar to those of the control group injected with saline.

## Conclusions

Dynamic combinatorial covalent chemistry is an extremely convenient screening protocol to identify strong binders to elusive biomacromolecules. This approach is especially appealing for heterogeneous flexible targets without clearly defined binding pockets, such as heparin. Our results underscore the success of the methodology, by optimizing two very strong binders with submicromolar affinity, as demonstrated using different experimental techniques (SPR, fluorescence, and NMR titrations). The combination of spectroscopic (fluorescence and NMR) measurements with molecular dynamics simulations has led to propose a detailed molecular recognition model, which is rather challenging with these anionic polysaccharides. We also found an excellent correlation between the results of the dynamic combinatorial screening and the heparin inhibition, as measured with an *in vitro* enzymatic assay related to blood coagulation. This agreement means a definitive validation of our approach. Remarkably, the selected binders show to be potent heparin antidotes *in vitro*, *ex vivo*, and *in vivo* using a mouse model. Thus, the dynamic combinatorial screening demonstrates to be a very powerful tool for the discovery of new hits in the development of future drugs where conventional approaches have found many difficulties to succeed.

## Experimental Section

### General

Reagents and solvents were purchased from commercial suppliers (Aldrich, Fluka, or Merck) and were used without further purification. Flash chromatographic purifications and preparative reversed-phase purifications were performed on a Biotage Isolera Prime equipment. TLCs were performed using 6 × 3 cm^2^ SiO_2_ precoated aluminum plates (ALUGRAM SIL G/UV254).

### Nuclear Magnetic Resonance (NMR)

Spectroscopic experiments for the characterization of compounds were carried out on an Agilent VNMRS400 instrument (400 MHz for ^1^H and 101 MHz for ^13^C). Chemical shifts (δH) are quoted in parts per million (ppm) and referenced to the appropriate NMR solvent peak(s). 2D-NMR experiments COSY, HSQC, and HMBC were used where necessary in assigning NMR spectra. Spin–spin coupling constants (*J*) are reported in hertz (Hz). For the experiments performed in aqueous buffer, a low-molecular-weight heparin was used: dp14 (from Iduron, prepared by high-resolution gel filtration of partial heparin lyase digestion of high-quality heparin, MW average ∼4100). One- and two-dimensional (1D and 2D) NMR experiments were performed at 298 K on a 500 MHz Bruker AVANCEIII-HD equipped with a *z*-gradient (65.7 G cm^–1^) inverse TCI-cryoprobe. Samples were dissolved in 5 mM Tris-d11 buffer with 50 mM NaCl (in D_2_O, pH 7.5, uncorrected pH meter reading). Bruker TopSspin 3.5pl6 standard pulse sequences were used for 1D and 2D experiments. For DOSY experiments, the stebpgp1s19 pulse sequence with WATERGATE 3919 for water suppression and one spoil gradient was used.

### Liquid Chromatography Coupled to Mass Spectrometry

Analyses were carried out at the IQAC Mass Spectrometry Facility, using a UPLC-ESI-TOF equipment: [Acquity UPLC BEH C_18_ 1.7 mm, 2.1 × 100 mm^2^, LCT Premier X_e_, Waters]. (CH_3_CN + 20 mM HCOOH and H_2_O + 20 mM HCOOH) mixtures at 0.3 mL/min were used as mobile phase.

### Dynamic Combinatorial Covalent Screening of Heparin Binders

A stock solution of imines was prepared by dissolving the necessary amounts of spermine (**1**) and the aromatic aldehydes in MeOH rendering a final concentration of 10 mM in all of the reagents. Then, two reaction mixtures were prepared in separate Eppendorf tubes from the same stock solution of the imines by mixing 10 μL of the imine stock solution with 90 μL of a solution of 5 mM NaBH_3_CN in aqueous 50 mM Tris buffer at pH 7.5 (1 mM final concentration of both spermine and each of the aldehydes) either in the absence or presence of 1.2 mg of heparin (1 mM final concentration of heparin, ca. 17–18 mM on repeating unit). The mixtures were allowed to react at room temperature for 24 h, then quenched with 50 μL of 2 M HCl, twofold diluted with water, and analyzed by UPLC-MS. The assignment of the peaks observed in the reactions was done on the basis of the *m*/*z* values and confirmed by injection of samples obtained from deconvoluted sublibraries. The amplification factors were calculated by dividing the normalized areas of the corresponding HPLC peaks in the presence of heparin (*A*_T_) by the areas obtained in the absence of heparin (*A*_0_). The observed species were assigned by ion detection UPLC-MS experiments. A total of 28 sublibraries were designed to make sure to cover all possibilities feasible within the whole compounds of the library. Nevertheless, the final products were not discernible by HPLC-MS in some cases (e.g., 3AA, 3AJ, and 3JJ), which prevented us to obtain a reliable *A*_T_/*A*_0_. We, thus, avoided the combination of aldehydes leading to degenerate masses. The experiments were performed in at least triplicate, and the AFs for those members appearing in several sublibraries were averaged.

### Synthesis and Characterization of Compounds

Compounds 3AA, 3AL,^48^ and 3BB^56^ have been previously described. The other molecules used in this work were synthesized as follows. All of the final compounds used for binding and biological assays showed ≥95% purity as determined by analytical HPLC.

#### 1,1′-(2,6,11,15-Tetraazahexadecane-1,16-diyl)bis(naphthalen-2-ol) (**3FF**)

Spermine (60 mg, 0.297 mmol) was dissolved in 25 mL of MeOH. 2-Hydroxy-1-naphthaldehyde (193 mg, 1.039 mmol) was then added dissolved in 12 mL of MeOH. The solution was stirred for 6 h. Then, NaBH_3_CN (126 mg, 2 mmol) was added and the reaction was stirred for 24 h. After addition of H_2_O (2 mL) and 1 M HCl (2 mL), the reaction was stirred for 1 h. The solvent was removed in vacuum. The reaction mixture was purified by reversed-phase chromatography with a gradient of ACN (0.1% TFA) and water (0.1% TFA) to yield 78 mg (51%) of pure product (99% by HPLC). ^1^H RMN (D_2_O, 400 MHz): δ 7.86 (m, 6H), 7.54 (t, 2H, *J*_1_ = 8 Hz), 7.36 (t, 2H, *J*_2_ = 8 Hz), 7.18 (d, 2H, *J*_3_ = 12 Hz), 4.65 (s, 2H), 3.14 (m, 4H), 3.00 (m, 8H), 2.04 (m, 4H), 1.62 (m, 4H). ^13^C RMN (D_2_O, 100 MHz): 154.1, 132.4, 132.1, 128.9, 128.4, 127.8, 123.7, 121.6, 117.3, 46.8, 44.4, 43.8, 41.6, 22.6, 22.4. ESI-MS: Calculated 514.3308; found 515.3389 (M + H)^+^.

#### 2,2′-(2,6,11,15-Tetraazahexadecane-1,16-diyl)bis(naphthalen-1-ol) (**3GG**)

Spermine (20 mg, 0.099 mmol) was dissolved in 5 mL of MeOH at 0 °C. 1-Hydroxy-2-naphthaldehyde (40 mg, 0.215 mmol) was carefully added dissolved in 2 mL of MeOH. After 6 h, the solution turned clear red. NaBH_3_CN (28 mg, 0.444 mmol) was then added. The day after, the solution turned brown. The reaction was stopped by the addition of 0.5 mL of H_2_O and 0.5 mL of 1 M HCl. The solvent was removed, and crude was purified by reversed-phase chromatography with a gradient of ACN (0.1% TFA) and water (0.1% TFA). A brownish solid (8.5 mg) was obtained as a pure product (purity = 97.4%, yield = 16%). ^1^H RMN (D_2_O, 400 MHz): δ 8.06 (2H, dd), 7.82 (2H, dd), 7.48 (6H, m), 7.3 (2H, d), 4.33 (4H, s), 3.05 (4H, tr), 2.93 (8H, m), 1.99 (4H, m), 1.57 (4H, m). ^13^C RMN (D_2_O, 100 MHz): δ 150.9, 135.0, 128.1, 127.8, 127.4, 126.3, 124.9, 121.4, 121.2, 113.1, 46.8, 44.4, 43.6, 22.6, 22.4. ESI-MS: Calculated 514.3308; found 515.3438 (M + H)^+^.

#### N^1^,N^1′^-(Butane-1,4-diyl)bis(N^3^-((2-methoxynaphthalen-1-yl)methyl)propane-1,3-diamine) (**3KK**)

Spermine (51 mg, 0.25 mmol) was dissolved in 20 mL of MeOH, and 2-methoxy-1-naphthaldehyde (116 mg, 0.625 mmol) was added dissolved in 5 mL of MeOH. After 4 h of stirring, the solvent was replaced by 20 mL of anhydrous THF (THF_anh_). NaBH_3_CN was then added, and the reaction was stirred overnight. The day after, the reaction was stopped by the addition of 2 mL of H_2_O and 2 mL of 1 M HCl. The solvent was removed, and crude was purified by reversed-phase chromatography with a gradient of ACN (0.1% TFA) and water (0.1% TFA). Pure 3KK (75 mg) was obtained as a white powder (purity = 99%, yield 60%). ^1^H RMN (H_2_O/D_2_O): δ 7.98 (2H, d, *J*_1_ = 8 Hz), 7.88 (2H, d, *J*_2_ = 8 Hz) 7.84 (2H, d, *J*_3_ = 8 Hz), 7.52 (2H, dd, *J*_1_ = *J*_4_ = 8 Hz), 7.37 (4H, m), 4.6 (m), 3.9 (6H, s), 3.1 (4H, m), 2.93 (8H, m), 1.99 (4H, m), 1.58 (4H, m). ^13^C RMN (D_2_O): δ 156.5, 132.4, 132.1, 129.0, 128.6, 128.0, 124.1, 121.3, 112.9, 110.2, 56.1, 46.9, 44.4, 43.9, 41.6, 22.6, 22.5. ESI-MS: Calculated 542.3621; found 543.3602 (M + H)^+^.

#### N^1^,N^1′^-(Butane-1,4-diyl)bis(N^3^-(naphthalen-2-ylmethyl)propane-1,3-diamine) (**3NN**)

Spermine (41 mg, 0.203 mg) was dissolved in 20 mL of THF_anh_ at 0 °C. 2-Naphthaldehyde (79 mg, 0.507 mmol) was dissolved in 10 mL of THF_anh_ and added dropwise. After 12 h of stirring, NaBH_3_CN (64 mg, 1.015 mmol) was added and the reaction was left overnight. The day after, the reaction was stopped by the addition of 2 mL of H_2_O and 2 mL of 1 M HCl. The solvent was removed, and crude was purified by reversed-phase chromatography with a gradient of ACN (0.1% TFA) and water (0.1% TFA). The product was isolated as 37 mg of a white fluffy solid (purity = 94.5% by HPLC, yield = 38%). ^1^H RMN (D_2_O, 400 MHz): δ 7.86 (8H, m), 7.49 (4H, m), 7.42 (2H, dd, *J*_1_ = 8.5 Hz, *J*_2_ = 1.5 Hz), 4.28 (4H, s), 2.99 (12H, m), 1.96 (4H, m), 1.57 (4H, m). ^13^C RMN (D_2_O, 100 MHz): δ 133.1, 132.7, 129.6, 129.0, 127.9, 127.7, 127.4, 127.0, 126.4, 117.7, 51.3, 46.9, 44.4, 43.8, 22.6, 22.5. ESI-MS: Calculated 482.3409; found 483.3493 (M + H)^+^.

#### 6,6′-(2,6,11,15-Tetraazahexadecane-1,16-diyl)bis(4-bromo-2-methoxyphenol) (**3OO**)

Spermine (52 mg, 0.257 mmol) was dissolved in 30 mL of MeOH. 5-Bromo-2-hydroxy-3-methoxybenzaldehyde (121 mg, 0.566 mmol) was then added dissolved in 10 mL of MeOH. After 4 h of stirring, the solvent was removed and replaced by 30 mL of THF_anh_. After that, NaBH_3_CN (65 mg, 1.028 mmol) was added and stirred overnight. The day after, the reaction was stopped by the addition of 2 mL of H_2_O and 2 mL of 1 M HCl. THF was carefully removed by vacuum evaporation, and the crude mixture was purified by reversed-phase chromatography with a gradient of ACN (0.1% TFA) and water (0.1% TFA). Pure product (66 mg) (purity by HPLC = 99%, yield = 40%) was obtained as a brownish powder. ^1^H RMN (D_2_O, 400 MHz): δ 7.16 (2H, s), 6.98 (2H, s), 4.1 (4H, s), 3.74 (6H, s), 2.98 (12H, m), 1.97 (4H, m), 1.61 (4H, m). ^13^C RMN (D_2_O, 100 MHz): 148.4, 144.0, 125.2, 118.5, 116.6, 111.1, 56.3, 46.8, 45.8, 44.4, 43.6, 22.6, 22.4. ESI-MS: Calculated 630.1416 and 632.1396; found 631.3065 and 633.2315 (M + H)^+^.

#### 2-(((3-((4-((3-Aminopropyl)amino)butyl)amino)propyl)amino)methyl)phenol (**3A**)

Spermine (120 mg, 0.594 mmol) was dissolved in 75 mL of THF_anh_ at 0 °C. 2-Hydroxybenzaldehyde (58 μL, 0.534 mmol) dissolved in 20 mL of MeOH was dropwise added, and the reaction was stirred overnight. The day after, NaBH_3_CN (67 mg, 1.07 mmol) was added and stirred for 24 h. The reaction was stopped by the addition of 3 mL of H_2_O and 3 mL of 1 M HCl. THF was carefully evaporated, and the reaction crude was purified by reversed-phase chromatography with a gradient of ACN (0.1% TFA) and water (0.1% TFA). 3A (77 mg) was obtained as a white powder (yield = 47%, purity = 91.1%). Some 3AA was also isolated. ^1^H RMN (D_2_O, 400 MHz): δ 7.2 (2H, m), 6.85 (2H, m), 4.11 (2H, s, overlapped with water signal), 2.97 (12H, m), 1.94 (4H, m), 1.61 (4H, m). ^13^C RMN (D_2_O, 100 MHz): δ 154.9, 131.7, 131.5, 120.6, 115.5, 114.8, 46.9, 44.4, 43.6, 36.4, 23.6, 22.6, 22.4. ESI-MS: Calculated 308.2576; found 309.2684 (M + H)^+^.

#### 2-(16-(9*H*-Fluoren-2-yl)-2,6,11,15-tetraazahexadecyl)phenol (**3AC**)

3A (30 mg, 0.097 mmol) was dissolved in 5 mL of methanol at 0 °C. 9*H*-Fluorene-2-carbaldehyde (110 mg, 0.580 mmol) was added dissolved in 15 mL of methanol. The day after, NaBH_3_CN (70 mg, 1.15 mmol) was added and the reaction was stirred overnight. The reaction was stopped by the addition of 2 mL of H_2_O. The solvent was removed, and crude was purified by reversed-phase chromatography with a gradient of ACN (0.1% TFA) and water (0.1% TFA). 3AC (5 mg) was obtained as a white powder (purity > 99%, yield = 12%). ^1^H RMN (D_2_O, 400 MHz): δ 7.82 (2H, m), 7.56 (2H, m), 7.25 (3H, m), 7.21 (2H, m), 6.88 (2H, m), 4.18 (2H, s), 4.14 (2H, s), 3.85 (2H, s), 3.00 (12H, m), 2.01 (4H, m), 1.62 (4H, m). ^13^C NMR (D_2_O, 100 MHz): δ 155.0, 144.4, 143.9, 142.5, 140.2, 131.7, 131.6, 128.7, 128.6, 127.7, 127.1, 126.6, 120.5, 120.4, 120.3, 117.7, 115.5, 114.5, 51.4, 46.9, 44.4, 43.6, 36.3, 22.6, 22.4. ESI-MS: Calculated 486.3359; found 487.4005 (M + H)^+^.

#### 1-(16-(2-Hydroxyphenyl)-2,6,11,15-tetraazahexadecyl)naphthalen-2-ol (**3AF**)

3A (20 mg, 0.065 mmol) was dissolved in 10 mL of THF_anh_ at 0 °C. 2-Hydroxy-1-naphthaldehyde (13 mg, 0.078 mmol) was added dissolved in 1 mL of THF_anh_. After 4 h, NaBH_3_CN (9 mg, 0.143 mmol) was added and the reaction stirred overnight. The day after, the reaction was stopped by the addition of 0.5 mL of H_2_O and 0.5 mL of 1 M HCl. The solvent was removed, and crude was purified by reversed-phase chromatography with a gradient of ACN (0.1% TFA) and water (0.1% TFA). 3AG (6 mg) was obtained as a light brown powder (purity > 99%, yield = 19%). ^1^H RMN (D_2_O, 400 MHz): δ 7.90 (3H, m, H20), 7.57 (1H, dd, H22, *J* = 8 Hz), 7.4 (1H, dd, *J* = 8 Hz), 7.29 (2H, m), 7.21 (1H, d, *J* = 9 Hz), 6.91 (2H, m), 4.68 (2H, s), 4.19 (2H, s), 3.17 (2H, m), 3.02 (10H, m), 2.08 (4H, m), 1.66 (4H, m). ^13^C RMN (D_2_O, 100 MHz): δ 155.1, 154.2, 132.5, 132.2, 131.7, 131.6, 129.0, 128.5, 127.9, 123.8, 121.3, 120.6, 117.8, 117.1, 115.6, 114.9, 108.3, 46.9, 44.5, 43.9, 43.7, 41.7, 22.7, 22.5, 22.4. ESI-MS: Calculated 464.3151; found 465.3169 (M + H)^+^.

#### 2-(16-(2-Hydroxyphenyl)-2,6,11,15-tetraazahexadecyl)naphthalen-1-ol (**3AG**)

3A (53 mg, 0.171 mmol) was dissolved in 15 mL of THF_anh_ at 0 °C. 1-Hydroxy-2-naphthaldehyde (35 mg, 0.205) was added dissolved in 1 mL of THF_anh_. After 4 h, NaBH_3_CN (24 mg, 0.377 mmol) was added and the reaction was stirred overnight. The day after, the reaction was stopped by the addition of 0.5 mL of H_2_O and 0.5 mL of 1 M HCl. The solvent was removed, and crude was purified by reversed-phase chromatography with a gradient of ACN (0.1% TFA) and water (0.1% TFA). 3AG (32 mg) was obtained as a light brown powder (purity 97.4%, yield = 40%). ^1^H RMN (D_2_O, 400 MHz): δ 8.06 (1H, dd, *J*_1_ = 3.4 Hz, *J*_2_ = 6.3 Hz); 7.82 (1H, dd, *J*_1_ = 3.4 Hz, *J*_2_ = 6.3 Hz); 7.49 (3H, m), 7.31 (1H, d, *J*_3_ = 8.4 Hz), 7.22 (2H, m), 6.85 (2H, m), 4.32 (2H, s), 4.11 (2H, s), 3.00 (12H, m), 1.99 (4H, m), 1.59 (4H, m). ^13^C RMN (D_2_O, 100 MHz): 155.0, 150.9, 135.0, 131.7, 131.5, 128.1, 127.8, 127.4, 126.3, 125.1, 121.4, 121.2, 120.5, 117.0, 115.5, 113.1, 46.9, 44.4, 43.6, 22.6, 22.4. ESI-MS: Calculated 464.3151; found 465.3228 (M + H)^+^.

#### 2-(16-(Naphthalen-2-yl)-2,6,11,15-tetraazahexadecyl)phenol (**3AN**)

3A (15 mg, 0.0485 mmol) was dissolved in 5 mL of THF_anh_ at 0 °C. 2-Naphthaldehyde (9 mg, 0.0582 mmol) was added dissolved in 1 mL of THF_anh_. After 4 h, NaBH_3_CN was added and the reaction was stirred overnight. The day after, the reaction was stopped by the addition of 0.5 mL of H_2_O and 0.5 mL of 1 M HCl. The solvent was removed, and crude was purified by reversed-phase chromatography with a gradient of ACN (0.1% TFA) and water (0.1% TFA). 3AN (15 mg) was obtained as a white powder (purity > 99%, yield = 69%). ^1^H RMN (D_2_O, 400 MHz): δ 7.87 (m, 4H, m), 7.50 (m, 2H), 7.43 (d, *J* = 8.44 Hz, 1H), 7.22 (m, 2H, H1), 6.85 (tr, *J* = 6.97 Hz, 2H), 4.3 (s, 2H), 4.12 (s, 2H), 3.00 (m, 12H), 1.98 (m, 4H), 1.59 (m, 4H). ^13^C RMN (D_2_O, 100 MHz): δ 155.0, 133.1, 132.7, 131.7, 131.5, 129.6, 129.1, 128.0, 127.7, 127.4, 127.0, 126.4, 120.5, 117.0, 115.5, 51.3, 46.9, 46.8, 44.4, 44.3, 43.7, 43.6, 22.6, 22.5, 22.4. ESI-MS: Calculated 448.3202; found 449.3520 (M + H)^+^.

#### 4-(((3-((4-((3-Aminopropyl)amino)butyl)amino)propyl)amino)methyl)phenol (**3J**)

Spermine (62 mg, 0.307 mmol) was dissolved in 40 mL of THF_anh_ at 0 °C. 4-Hydroxybenzaldehyde (30 μL, 0.276 mmol) dissolved in 10 mL of MeOH was dropwise added, and the reaction was stirred overnight. The day after, NaBH_3_CN (34 mg, 0.552 mmol) was added and stirred for 24 h. The reaction was stopped by the addition of 2 mL of H_2_O and 2 mL of 1 M HCl. THF was carefully evaporated in vacuum, and reaction crude was purified by reversed-phase chromatography with a gradient of ACN (0.1% TFA) and water (0.1% TFA). 3J (55 mg) was obtained as a white powder (yield = 66%, purity = 98.1%). Some minor amount of 3JJ was also isolated. ^1^H RMN (D_2_O, 400 MHz): δ 7.18 (2H, d, *J* = 8 Hz), 6.78 (2H, d, *J* = 8 Hz), 3.95 (2H, s), 2.92 (12H, m), 1.89 (4H, m), 1.58 (4H, m). ^13^C RMN (D_2_O, 100 MHz): 156.7, 131.4, 123.3, 115.9, 50.8, 47.1, 47.0, 44.8, 44.6, 43.9, 36.7, 24.2, 23.3, 23.2. ESI-MS: Calculated 308.2576; found 309.2698 (M + H)^+^.

#### 1-(16-(4-Hydroxyphenyl)-2,6,11,15-tetraazahexadecyl)naphthalen-2-ol (**3FJ**)

3J (23 mg, 0.074 mmol) was dissolved in 10 mL of THF_anh_ at 0 °C. After that, 2-hydroxy-1-naphthaldehyde (15 mg, 0.089 mmol) was added dissolved in 5 mL of THF_anh_. The day after, NaBH_3_CN (9 mg, 0.148 mmol) was added and the reaction was stirred overnight. The reaction was stopped by the addition of 1 mL of H_2_O and 1 mL of 1 M HCl. The solvent was removed and crude was purified by reversed-phase chromatography with a gradient of ACN (0.1% TFA) and water (0.1% TFA). 3FJ (28 mg) was obtained as a white fluffy solid (purity = 98.5%, yield 82%). ^1^H RMN (D_2_O, 400 MHz): δ 7.81 (3H, m), 7.5 (1H, dd, *J*_1_ = 7.5 Hz, *J*_2_ = 1 Hz), 7.32 (1H, dd, *J*_1_ = 7.5 Hz, *J*_2_ = 1 Hz), 7.19 (2H, d, *J*_3_ = 8.5 Hz), 7.14 (1H, d, *J*_5_ = 8 Hz), 6.79 (2H, d, *J*_3_ = 8.5 Hz), 4.6 (overlapped with water signal), 4.02 (3H, s), 3.09 (2H, m), 2.95 (10H, m), 1.95 (4H, m), 1.58 (4H, m). ^13^C RMN (D_2_O, 100 MHz): δ 156.7, 154.2, 132.4, 132.1, 132.0, 131.6, 129.0, 128.4, 127.8, 123.7, 121.2, 117.3, 115.9, 108.2, 50.6, 46.9, 44.4, 43.7, 43.4, 41.7, 40.0, 22.6, 22.5. ESI-MS: Calculated 464.3151; found 465.3317 (M + H)^+^.

#### 2-(((3-((4-((3-Aminopropyl)amino)butyl)amino)propyl)amino)methyl)-4-bromo-6-methoxyphenol (**3O**)

Spermine (54 mg, 0.267 mmol) was dissolved in 25 mL of THF_anh_ at 0 °C. 5-Bromo-2-hydroxy-3-methoxybenzaldehyde (54 mg, 0.24 mmol) dissolved in 10 mL of MeOH was dropwise added, and the reaction was stirred overnight. The day after, NaBH_3_CN (30 mg, 0.48 mmol) was added and stirred for 24 h. The reaction was stopped by the addition of 2 mL of H_2_O and 2 mL of 1 M HCl. THF was carefully evaporated, and reaction crude was purified by reversed-phase chromatography with a gradient of ACN (0.1% TFA) and water (0.1% TFA). 3O (30 mg) was obtained as a brown powder (yield = 31%, purity = 97.3%). Some amount of 3OO was also isolated. ^1^H RMN (D_2_O, 400 MHz): δ 7.18 (1H, s), 7.01 (1H, s), 4.11 (2H, s), 3.77 (3H, s), 2.98 (12H, m), 1.96 m (4H, m), 1.65 (4H, m). ^13^C RMN (D_2_O, 100 MHz): δ 148.4, 144.0, 125.2, 118.5, 116.6, 111.1, 56.3, 46.9, 45.8, 44.4, 44.3, 36.4, 23.6, 22.7, 22.4. ESI-MS: Calculated 416.1787 and 418.1766; found 417.1704 and 419.1703 (M + H)^+^.

#### 1-(16-(5-Bromo-2-hydroxy-3-methoxyphenyl)-2,6,11,15-tetraazahexadecyl)naphthalen-2-ol (**3FO**)

3O (15 mg, 0.036 mmol) was dissolved in 5 mL of THF_anh_ at 0 °C. 2-Hydroxy-1-naphthaldehyde (7 mg, 0.043 mmol) was added dissolved in 1 mL of THF_anh_. The day after, NaBH_3_CN (4.5 mg, 0.072 mmol) was added and the reaction was stirred overnight. The reaction was stopped by the addition of 0.5 mL of H_2_O and 0.5 mL of 1 M HCl. The solvent was removed, and crude was purified by reversed-phase chromatography with a gradient of ACN (0.1% TFA) and water (0.1% TFA). 3FO (5 mg) was obtained as a white powder (purity > 99%, yield = 25%). ^1^H RMN (D_2_O, 400 MHz): δ 7.86 (3H, m), 7.54 (1H, dd, *J* = 7.5 Hz), 7.36 (1H, dd, *J* = 7.5 Hz), 7.18 (2H, m,), 7.01 (1H, d, *J* = 2 Hz), 4.64 (2H, s), 4.12 (2H, s), 3.88 (3H, s), 3.12 (2H, m), 3.00 (10H, m) 2.03 (4H, m), 1.63 (4H, m). ^13^C RMN (D_2_O, 100 MHz): δ 154.2, 148.4, 144.4, 132.5, 132.1, 129.0, 128.4, 127.9, 125.2, 123.7, 121.2, 117.3, 116.6, 114.8, 111.2, 108.2, 56.3, 46.9, 44.4, 43.8, 41.6, 22.6, 22.5. ESI-MS: Calculated 572.2362 and 574.2342; found 573.2471 and 575.2441 (M + H)^+^.

#### 4-Bromo-2-methoxy-6-(16-(naphthalen-1-yl)-2,6,11,15-tetraazahexadecyl)phenol (**3LO**)

3O (15 mg, 0.036 mmol) was dissolved in 5 mL of THF_anh_ at 0 °C. 1-Naphthaldehyde (6 μL, 0.043 mmol) was added dissolved in 1 mL of THF_anh_. The day after, NaBH_3_CN (4.5 mg, 0.072 mmol) was added and the reaction was stirred overnight. The reaction was stopped by the addition of 0.5 mL of H_2_O and 0.5 mL of 1 M HCl. The solvent was removed, and crude was purified by reversed-phase chromatography with a gradient of ACN (0.1% TFA) and water (0.1% TFA). 3LO (7 mg) was obtained as a brownish powder (purity 98.7%, yield = 35%). ^1^H RMN (D_2_O, 400 MHz): δ 7.90 (3H, m), 7.50 (4H, m), 7.14 (1H, d, *J* = 4 Hz), 6.97 (1H, d, *J* = 4 Hz), 4.65 (2H, s), 4.08 (2H, s), 3.73 (3H, s), 3.12 (2H, m), 2.96 (10H, m), 1.98 (4H, m), 1.59 (4H, m). ^13^C RMN (D_2_O, 100 MHz): δ 148.4, 144.1, 133.6, 130.8, 130.6, 129.5, 129.1, 127.5, 126.7, 126.3, 125.6, 125.3, 122.4, 118.6, 116.7, 111.2, 56.4, 48.1, 46.9, 45.9, 44.3, 22.7, 22.6, 22.4. ESI-MS: Calculated 556.2413 and 558.2392; found 557.2440 and 559.2421 (M + H)^+^.

### Dynamic Light Scattering (DLS)

DLS experiments were carried out in a 3DDLS photon crossed correlation spectrometer (LS Instruments). This equipment has a He–Ne (632.8 nm) laser that allows to us measure samples with higher turbidity because the crossed correlation technology (3D-cross) minimizes the multiple dispersion. The measurements were carried out at 298 K and a 90° angle for 100 s, in triplicate, and the results are given as the mean with standard deviation.

### Surface Plasmon Resonance (SPR)

Affinity experiments between inhibitors and heparin were performed on an Open SPR (Nicoya). All measurements were performed at 25 °C using a working buffer of 25 mM Tris buffer at pH 7.5. Biotin-loaded sensor chips (NICOYA) were further functionalized with streptavidin (50 μg/mL) and later with biotin-heparin (50 μg/mL). Binding experiments to heparin were performed by injecting inhibitors at desired concentrations and at a rate of 40 μL/min. Between binding assays, the surface was regenerated by exposure to an injection of 10 mM HCl. Fitting was performed by Trace Drawer software using a “one-to-one two-state algorithm”, which considers a 1:1 binding with a further equilibrium like a conformational change. Results obtained from three independent experiments at three different concentrations of the ligands were fit globally to render the corresponding on/off rate constants and the apparent dissociation constants (*K*_D_^app^). The somehow large errors in the determined *K*_D_^app^ can be a consequence of the rough approximations assumed with the applied binding mode and also due to the polydispersity of the heparin sample. Anyway, since the difference in the corresponding dissociation constants generally exceeds the estimated errors, we are confident that they can be used for comparison purposes.

### Fluorescence Spectroscopy Titration

Fluorescence emission and excitation spectra were collected on a Photon Technology International Instrument, the Fluorescence Master Systems, using the Software Felix32 and cuvettes with 10 mm path length. Stock solutions of the corresponding binder (10 μM) and heparin (18 IU/mL) were prepared in 1 mM Bis-Tris buffer at pH 7.5. Then, 2 mL of the binder solution was placed on a quartz cell and the emission fluorescence spectrum was measured upon excitation at 280 nm. Then, small volumes of the heparin stock solution were added to the cell, and the fluorescence spectra were acquired after each addition. For 3AC, the titration experiments were fitted using HypeSpec2008 software,^58,59^ which allows a nonlinear global fitting of the full emission spectra to a binding mode as defined by the user.

### Computational Methods

All of the molecular dynamics simulations were performed with AMBER 2020^60^ with resort to GPU acceleration.^61−63^ The GLYCAM_06j-1 force field^64^ with additional parameters for glycosaminoglycans^65^ was used to parameterize the Hep molecules, while GAFF2 parameters^66^ and atomic RESP charges^67^ were used for the heparin ligands. The Hep model molecules were built with the GAG Builder^68^ implemented in GLYCAM-Web^69^ which allows us to build carbohydrates with different sequences and conformations. All sulfate, sulfamate, and carboxylate groups of the heparin models were modeled in their ionized state (i.e., total charge −32) and GLYCAM partial charges were assigned adjusting the partial charge on the sulfur-bound O and N atoms according to the GLYCAM procedure for charge development. On the other hand, the ligands 3AC, 3FF, and 3AL were built within Maestro^70^ with their four amino groups protonated (i.e., total charge +4) and energy-minimized with the program Macromodel^71^ using its default force field OPLS4^72^ and GB/SA water solvation conditions.^73^ Next, these ligand structures were optimized and their electrostatic potential (ESP) was calculated at the HF/6-31G* level with Gaussian 09.^74^ The atomic charges on each molecule were then calculated by RESP fitting using Antechamber.^75^ The simulation systems were built by merging into a single structure one Hep dp16 molecule plus 6 randomly placed ligand molecules of a single type (3AC, 3FF, or 3AL) or of two types (competence simulations: 3AC vs 3FF or 3AC vs 3AL). Then, tLEAP was used to assign parameters to the structures of the heparin-ligand systems using GLYCAM and GAFF2 force fields for heparin and the ligands, respectively, as well as to solvate them with a truncated octahedral TIP3P box that extended 12 Å away from any solute atom, adding enough chloride or sodium ions to reach neutrality. Thus, the final systems included 1 Hep, 6 ligands, 8 Na^+^, and ∼14 000 water molecules for the simulations with a single ligand, or one Hep, six of each ligand, 16 Cl^–^, and ∼26 500 water molecules for the competence simulations. The solvated systems were minimized with 1000 steps of Steepest-Descent plus 4000 steps of Conjugate-Gradient. Systems were then thermalized and equilibrated in three successive MD steps: (1) 9000 MD steps from 0 to 300 K, 2 fs per step, periodic boundary conditions (PBC) on the NVT ensemble, Langevin dynamics for temperature control and thermostat collision frequency of 1.0 ps^–1^, cutoff of 10 Å for short-range interactions, and particle mesh Ewald (PME) method for long-range interactions, constraining bonds to hydrogens with SHAKE; (2) 11 000 MD steps at 300 K, rest of conditions as before; (3) 10^7^ MD steps (i.e., 20 ns), conditions as before except for PBC on the NPT ensemble, at 1 atm with isotropic scaling and Monte Carlo barostat control. Production MD simulations (250 or 500 ns, 2 fs per step) were run using the same parameters as the previous equilibration step, saving snapshots and energy information every 50 ps. Trajectories were analyzed with Cpptraj^76^ and visualized with Pymol.^77^

### Blood Coagulation Factor *In Vitro* Enzymatic Assays

Recombinant antithrombin III and coagulation Factor Xa were obtained from the Berichrom Heparin test, supplied by SIEMENS. Stock solutions were prepared following the indications of the test kit: Human antithrombin III (1 IU/mL), Factor Xa reagent (0.4 μg/mL, human plasma fraction with the additives Tris, sodium chloride, and EDTA), and a chromogenic substrate specific for factor Xa (4 mM of *Z*-d-Leu-Gly-ArgANBA-methyl amide). On the other hand, 4-nitroaniline (Sigma-Aldrich) was added to the substrate solution at a concentration of 1.6 mM and was used as an internal standard to quantify the hydrolysis of the chromogenic substrate. Heparin (sodium salt from porcine intestinal mucosa, polydisperse, from 6000 to 30 000 Daltons) was purchased from Sigma-Aldrich. All compounds were dissolved in Milli-Q water at the desired stock concentrations prior to starting the assays and kept at 4 °C. Various concentrations of the ligand, heparin (0.1 IU/mL), human antithrombin III solution (3.5 μL), and Factor Xa reagent solution (35 μL) were consecutively added to an Eppendorf and brought to a total volume of 145 μL. Then, 20 μL of Reagent Substrate solution was added to start the experiment and the mixture was vigorously shaken at 25 °C. The samples (20 μL) were taken at minutes 5/10/15/20/30/50/80/120, diluted with 40 μL of acetic acid (20% v/v), and injected in the analytical HPLC. The gradient ranged from 5% of ACN (0.1% formic acid) in water (0.1% formic acid) to 100% of ACN in 24 min using a 15 × 4.6 mm^2^ KROMAPHASE C_18_ 5.0 μm column (retention time of cleaved chromophore 9.1 min, retention time of chromogenic substrate 13.9 min, retention time of 4-nitroaniline 15.4 min). The concentrations of reagents have been adjusted compared with previous studies with the intention to slow down the total exhaustion of peptide substrate. In this way, the activity can be more carefully modulated and any change is easier to detect. FXa/AT_III_ activity was represented as the percent of hydrolysis, which was calculated from the normalized area of the cleaved peptide at 405 nm at each corresponding time point. The experiment carried out in the absence of heparin was considered as the maximum of activity while experiment with heparin and no ligand was considered as negative control (maximum inhibition of FXa by heparin). The concentration of heparin was selected for the measurement to render a significant inhibition within experimental time, while allowing the reaction to proceed.

### *Ex Vivo* Blood Coagulation Assays

Freshly collected mouse blood was collected and directly used without further treatment. Aliquots (300 μL) were prepared. To them, Hep (130 μM) and various concentrations of ligand (3AC or 3FF) were added. The samples of the different conditions were added to an Eppendorf tube, and a picture was taken after 15 min. The clot formation was confirmed by turning around the Eppendorf vials.

### Scanning Electron Microscopy (SEM)

Mouse fresh blood was collected in 300 μL fractions. Then, Heparin (130 μM), 3AC (90 μM), and 3FF (90 μM) were added where corresponding. After gently shaking the blood samples for 10 min, 30 μL of each aliquot was disseminated on a glass coverslip. Then, the samples were fixed by the addition of 2.5% glutaraldehyde in 100 mM sodium cacodylate buffer at pH 7.0 (overnight at 4 °C). After that, the samples were successively washed with: (1) sodium cacodylate buffer (1 × 2 min), (2) Milli-Q water (2 × 2 min), (3) 25% EtOH in H_2_O (1 × 5 min), (4) 50% EtOH in H_2_O (1 × 5 min), (5) 75% EtOH in H_2_O (1 × 5 min), (6) 95% EtOH in H_2_O (3 × 5 min), and (7) 100% EtOH (3 × 10 min). Finally, the samples were dried and kept at 4 °C. NOTE: It is handier to put the coverslips in 12-well plates as operations become easier to manage. The samples were mounted in double-coated carbon conductive tape, and pictures were taken by a Hitachi TM-4000 Plus II SEM microscope (1000 or 2000 magnifications). Pictures (Figure S37) are shown with no further postprocessing treatment.

### Cell Cytotoxicity Assays

For the cell growth, A549 were maintained in DMEM (4500 mg/mL glucose) culture medium (Sigma) containing 10% fetal calf serum (FCS), 2 mM glutamine, 50 U/mL penicillin, and 0.05 g/mL streptomycin, at 37 °C under 5% CO_2_ atmosphere. The viability of A549 cells was tested using the 3-(4,5-dimethylthiazol-2-yl)-2,5-diphenyltetrazolium bromide (MTT) assay. In it, exponentially growing cells were detached from the culture flasks using a trypsin—0.25% EDTA solution and the cell suspension was seeded onto 96-well (Nunclon) at a concentration of 7000 cells/well. The toxicity of 3XX compounds was tested as follows: 24 h after seeding, the culture medium was discarded and replaced by compound solution in medium to the desired concentration. After 1 h of incubation at 37 °C under a 5% CO_2_ atmosphere, the solvent was discarded and replaced with MTT (0.5 mg/mL). After 2 h of incubation with MTT, the medium was discarded by aspiration and DMSO was added to dissolve formazan, a dark blue colored crystal observed in the wells. Absorbance was measured at 570 nm in a spectrophotometric Biotek Sinergy 2 Microplate Reader (Agilent) 30 min after the addition of DMSO. Cell viability is expressed as an absorbance percent ratio of cells treated with compound to untreated cells, which were used as a control. Results are the average from three independent experiments (Figure S38).

### *In Vivo* Tail Transection Assays with Mice

Eight-week-old CD-1 male mice (Janvier-Labs) were used for the study. The animals were housed in the Research and Development Center animal facility (CID-CSIC) under a 12 h light–dark cycle in an environmentally controlled room with free access to water and food. All procedures were approved by the Animal Care and Use Committees of CID-CSIC and conducted in accordance with the institutional guidelines under a license from the local government (agreement number 11120). The mice were randomly assigned into six groups (*n* = 6 for each group). They were anesthetized by i.p. injections of 0.465 mg/kg xylazine (Rompun, Bayer DVM) and 1.395 mg/kg ketamine (Imalgene 100, Merial Laboratorios). Then, different solutions were injected intravenously according to the assigned group: saline group (only saline was given), Hep group (Hep injection, 100 IU/kg), 3AC (two groups, Hep injection followed by injection of 3AC at 2.2 or 4.4 mg/kg), and 3FF (two groups, Hep injection followed by injection of 3FF at 2.2 or 4.4 mg/kg). Two additional control groups (*n* = 3) were included, in which only the ligand (3AC or 3FF) at the highest dose (4.4 mg/kg) was injected. At 5 min after injections, tails were transected at 5 mm from the tip and immediately inserted in a tube containing 1 mL of saline buffer immersed in a water bath at 37 °C, where the blood draining out of the wound was collected for 10 min. The blood draining out from the wound was collected in each tube. Tails were let bleed for 10 min. The total blood volume in each tube was quantified by spectrophotometry (absorbance at 414 nm) from a standard curve that was constructed with known volumes of blood hemoglobin concentration and the corresponding absorbance value. Statistical analysis of the data was done with Kaleida Graph 4.5.4 using ANOVA.

## Supporting Information Available

DCC screening; assignment of the NMR signals; copies of NMR spectra, ESI-MS spectra, and HPLC traces; and additional tables and supporting figures (PDF)
Representative simulation of the competence between 6 molecules of 3AC (green C-atoms) and 6 molecules of 3FF (cyan C-atoms) for binding to a dp16 Hep model ((^1^C_4_)Ido-(^4^C_1_)Glc, orange C-atoms). Only the initial 170 ns of the simulation are shown (Video S1) (MOV)
Representative simulation of the competence between 6 molecules of 3AC (green C-atoms) and 6 molecules of 3AL (pink C-atoms) for binding to a dp16 Hep model ((^1^C_4_)Ido-(^4^C_1_)Glc, orange C-atoms). Only the initial 170 ns of the simulation are shown (Video S2) (MOV)

(PDF)
(MOV)
(MOV)

## Supplementary Material

jm1c02054_si_001.pdf

jm1c02054_si_002.mov

jm1c02054_si_003.mov

## Nonstandard Abbreviations

AF: amplification factors
AT_III_: antithrombin III
DCC: dynamic combinatorial chemistry
DLS: dynamic light scattering
DOSY: diffusion ordered spectroscopy
FXa: coagulation factor Xa
Hep: heparin
MTT: 3-(4,5-dimethylthiazol-2-yl)-2,5-diphenyltetrazolium bromide
SEM: scanning electron microscopy
SPR: surface plasmon resonance
