# Supplementary material for: Dynamic Combinatorial Optimization of In Vitro and In Vivo Heparin Antidotes
Source: J Med Chem. 2022 Mar 2;65(6):4865–77. doi: 10.1021/acs.jmedchem.1c02054 (PMC8958503; doi:10.1021/acs.jmedchem.1c02054)
Supplement: Supplementary file 1 — jm1c02054_si_001.pdf [file jm1c02054_si_001.pdf]

Supporting Information for:

Dynamic combinatorial optimization of in vitro and in vivo heparin antidotes

Daniel Carbajo,[a] Yolanda Pérez,[b] Marta Guerra-Rebollo,[c] Eva Prats,[d] Jordi Bujons[a] and Ignacio Alfonso[a,*]

[a]Department of Biological Chemistry and [b]NMR Facility, Institute for Advanced Chemistry of Catalonia (IQAC-CSIC), Jordi Girona 18-26, 08034, Barcelona, Spain.

[c]Grup d'Enginyeria de Materials (Gemat), Institut Químic de Sarriá (IQS), Universitat Ramon Llull (URL), Via Augusta 390, 08017 Barcelona, Spain.

[d]Research and Development Center (CID-CSIC), Jordi Girona 18-26, 08034, Barcelona, Spain.

e-mail: ignacio.alfonso@iqac.csic.es

<u>Table of contents:</u>

Dynamic combinatorial screening setup and results	S-3
Synthetic protocols and characterization data	S-6
Aggregation studies with 3FF	S-56
Binding studies on a heparin functionalized SPR chip	S-63
Binding studies in solution	S-65
In vitro enzymatic assays	S-86
Molecular modeling studies	S-88
Scanning Electron Microscopy of ex vivo experiments	S-93
In vitro cytotoxicity assays	S-94
References	S-95

Dynamic combinatorial screening setup and results.

Table S1. Selected combination of building blocks for the dynamic deconvolution of the DCL.

Sub-DCL	Amine	Aldehyde	Aldehyde	Aldehyde	Aldehyde
1	3	A	F	L	N
2	3	A	L	F	C
3	3	C	M	H	N
4	3	L	D	I	J
5	3	E	F	G	O
6	3	C	M	I	J
7	3	A	L	F	O
8	3	A	D	N	G
9	3	C	M	K	L
10	3	N	E	I	K
11	3	B	F	I	O
12	3	J	K	G	C
13	3	L	B	I	R
14	3	C	H	J	E
15	3	M	H	O	B
16	3	E	B	D	H
17	3	D	J	O	I
18	3	H	N	I	G
19	3	I	G	O	L
20	3	C	M	O	H
21	3	M	I	E	A
22	3	A	B	J	G
23	3	A	G	L	H
24	3	N	B	O	H
25	3	C	M	D	F
26	3	E	O	D	L
27	3	A	B	K	L
28	3	F	J	L	O

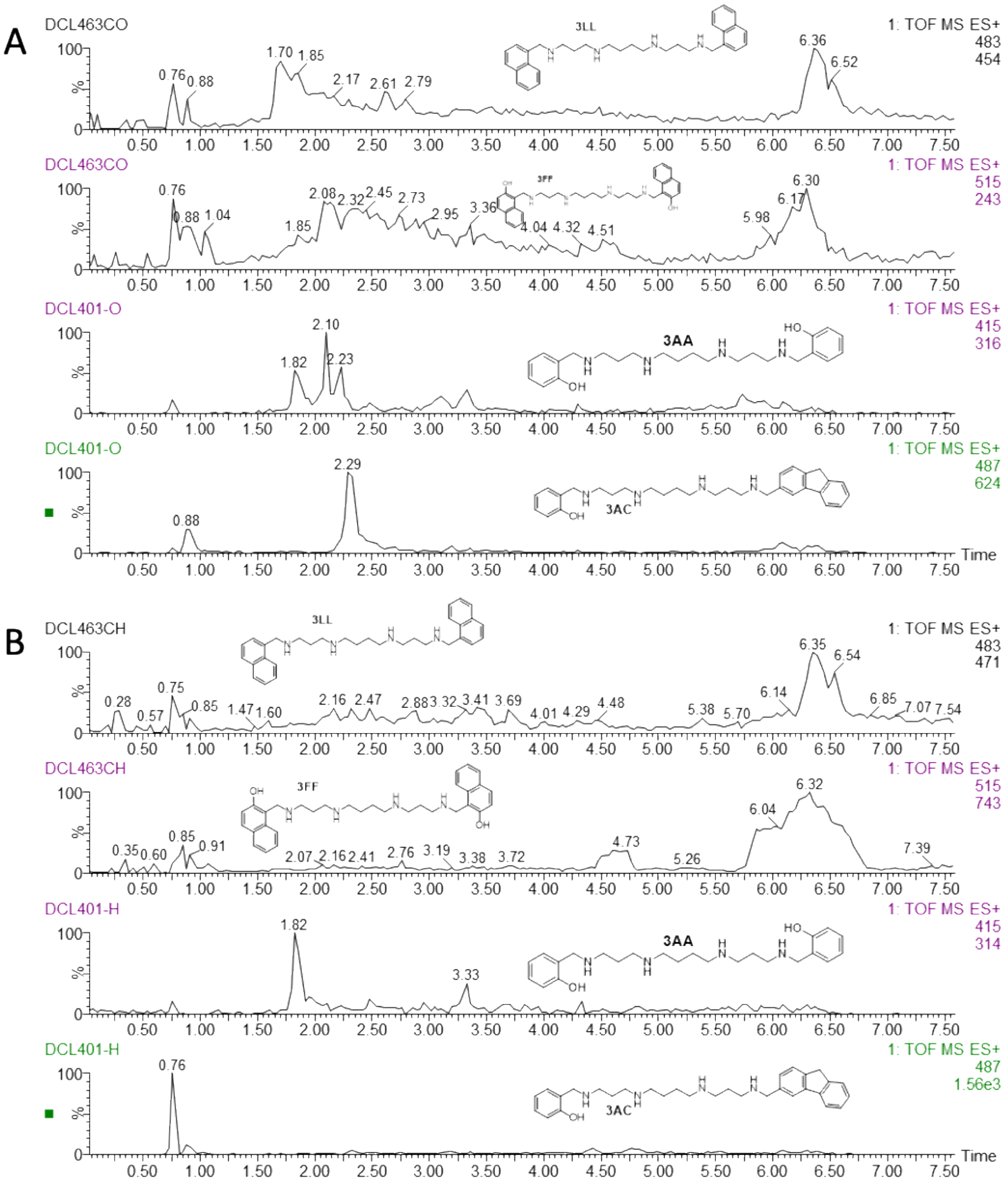

Figure S1. Selected representative Ion-Selective UPLC traces for library members obtained from the reductive amination reaction in the absence (A) or in the presence (B) of heparin. The two numbers on the up-right corner of each chromatogram correspond to the m/z values and the peak area, respectively. In many cases, the reaction with Hep produced a simplified IS-UPLC trace, suggesting that in the absence of the template, several isomers corresponding to different alkylation sites were obtained. The identity of the amplified isomer was corroborated in each case by co-injection of the DCL with the pure molecules synthesized as a single isomer bearing the alkylation at the primary amine nitrogen atoms (see below).

Table S2. Averaged amplification factors for the complete DCL (n.d. means not determined).

	**A**	**B**	**C**	**D**	**E**	**F**	**G**	**H**	**I**	**J**	**K**	**L**	**M**	**N**	**O**
**A**	0.836	1.180	5.580	2.330	0.720	2.813	3.360	n.d.	2.910	n.d.	0.830	1.382	0.440	1.120	n.d.
**B**		1.063	0.720	n.d.	0.780	1.060	0.910	1.207	0.530	0.280	1.580	0.805	1.310	1.130	1.985
**C**			1.032	0.630	0.850	0.900	0.960	0.590	1.120	1.000	1.310	0.785	0.803	0.990	0.810
**D**				0.913	0.870	1.575	1.000	0.540	0.890	1.465	0.840	0.930	0.530	1.160	0.677
**E**					0.312	n.d.	n.d.	0.976	0.825	0.850	0.120	0.905	0.880	0.610	0.810
**F**						3.417	n.d.	1.800	0.470	1.190	0.270	1.282	1.705	1.540	2.530
**G**							0.754	0.967	0.785	0.600	0.270	2.747	1.230	1.150	0.650
**H**								1.248	0.978	1.515	1.110	1.410	n.d.	2.660	0.760
**I**									2.308	0.310	0.470	0.940	0.803	1.110	1.770
**J**										0.782	0.590	0.695	1.045	2.180	0.770
**K**											0.843	1.460	0.740	1.640	0.520
**L**												0.928	0.920	1.120	1.646
**M**													1.488	0.740	0.800
**N**														0.782	2.215
**O**															0.729

Synthetic protocols and characterization data.

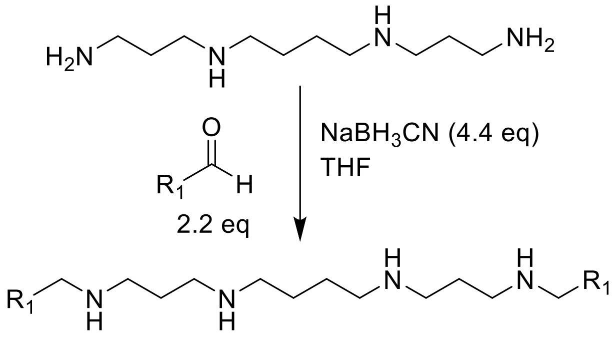

Scheme S1. General synthesis of symmetric polyamine binders.

Spermine (1 eq) was dissolved in THF_{anh} at 0°C. Aldehyde of interest $\mathbf{R_1}$ (2.2 eq) was then added dissolved in THF_{anh}. The solution was stirred overnight. Then, $NaBH_3CN$ (4.4 eq) was added and the reaction was stirred 24h. After addition of H_2O and HCl 1M (wait 1h after additions), THF was removed by rotavaporation. Reaction mixture was purified by reverse phase chromatography with a gradient of ACN (0.1% TFA) and water (0.1% TFA) to yield $\mathbf{3R_1R_1}$ as pure product (Yield around 60% and purity 90-99% by HPLC).

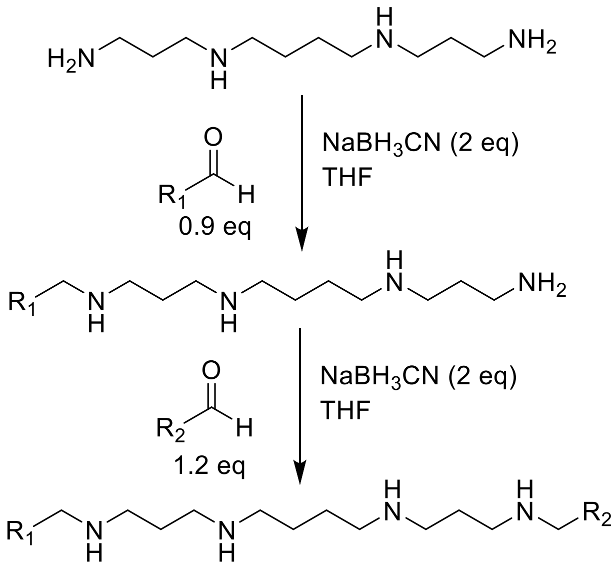

Scheme S2. General synthesis of asymmetric polyamine binders.

Step 1: Spermine (1eq) was dissolved in anhydrous THF at 0°C. First aldehyde of interest $\mathbf{R_1}$ (0.9 eq) dissolved in anhydrous THF was dropwise added and the reaction was stirred overnight. The day after, $NaBH_3CN$ (2 eq) was added and stirred 24h. Reaction was stopped by addition of H_2O and 1M HCl (1h after additions). THF was carefully evaporated in vacuum and reaction crude was purified by reverse phase chromatography with a gradient of ACN (0.1% TFA) and water (0.1% TFA). $\mathbf{3R_1}$ were obtained as pure product (yield around 30% and purity 90-99% by HPLC). Some $\mathbf{3R_1R_1}$ was also isolated.

Step 2: $\mathbf{3R_1}$ (1eq) was dissolved in 5mL of Methanol at 0°C. Second aldehyde of interest $\mathbf{R_2}$ (1.2 eq) was added dissolved in MeOH. The day after, $NaBH_3CN$ (2 eq) was added and the reaction stirred overnight. Reaction was stopped by addition of H_2O. Solvent was removed and crude purified by reverse phase chromatography with a gradient of ACN (0.1% TFA) and water (0.1% TFA). $\mathbf{3R_1R_2}$ was obtained as pure product (yield around 70% and purity 90-99% by HPLC).

Detailed characterization:

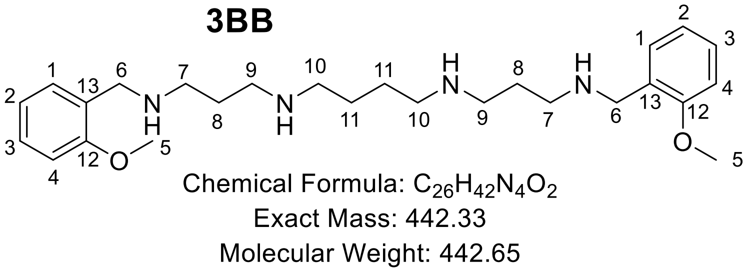

^{1}H RMN (D_2O): δ 7.37 (ddd; 2H; J_1=7.5Hz, J_2=8.3Hz, J_3=1.4Hz; H3), 7.22 (dd; 2H, J_4=7.5Hz, J_3= 1.4Hz; H1), 6.99 (d; 2H; J_2=8.3Hz; H4), 6.91 (dd; 2H, J_1=7.5Hz; J_4=7.5Hz; H2), 4.10 (s; 4H, H6), 3.74 (s; 6H, H5), 2.98 (m, 12H, H7, H9, H10), 1.97 (m, 4H, H8), 1.6 (m, 4H, H11).

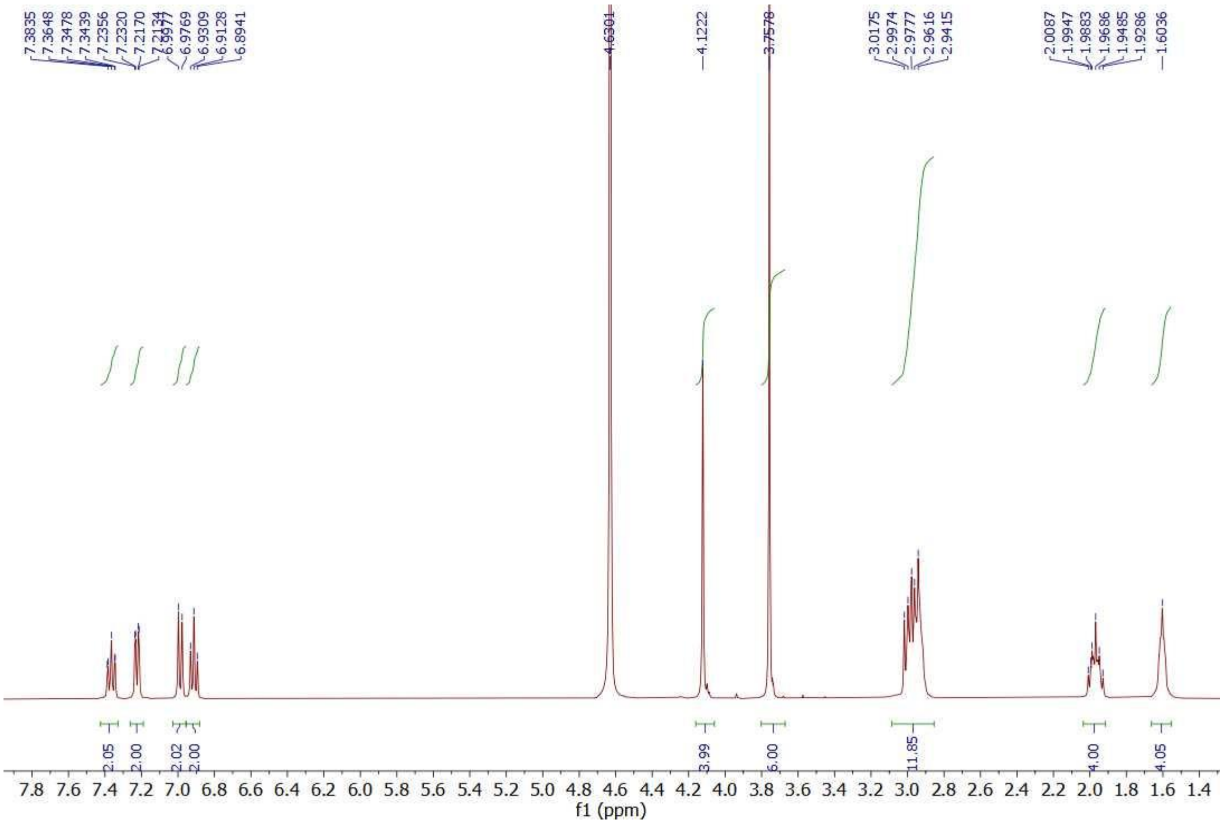

^{13}C RMN (D_2O): δ 157.7 (C12), 131.7 (C1), 131.5 (C3), 120.9 (C13), 118.2 (C2), 111.2 (C4), 55.3 (C5), 47.0 (C10), 46.9 (C6), 44.4 (C9), 43.7 (C7), 22.6 (C11), 22.4 (C8).

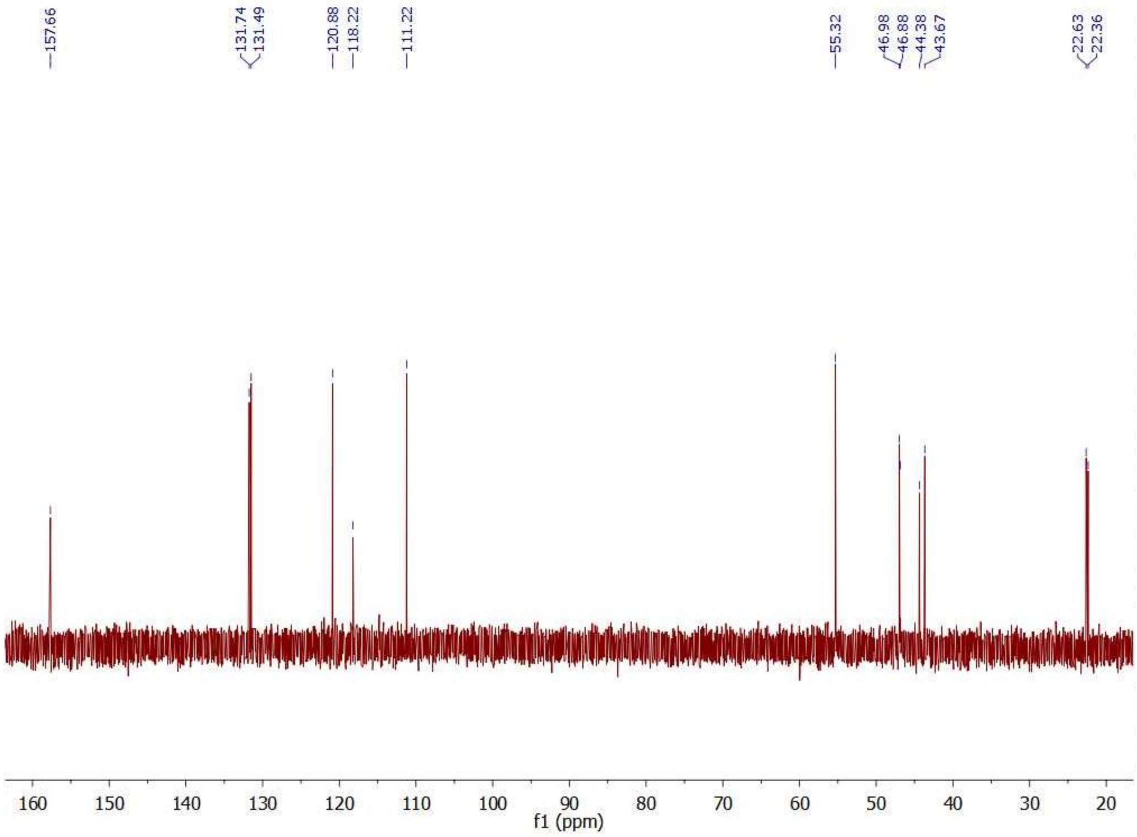

HMBC:

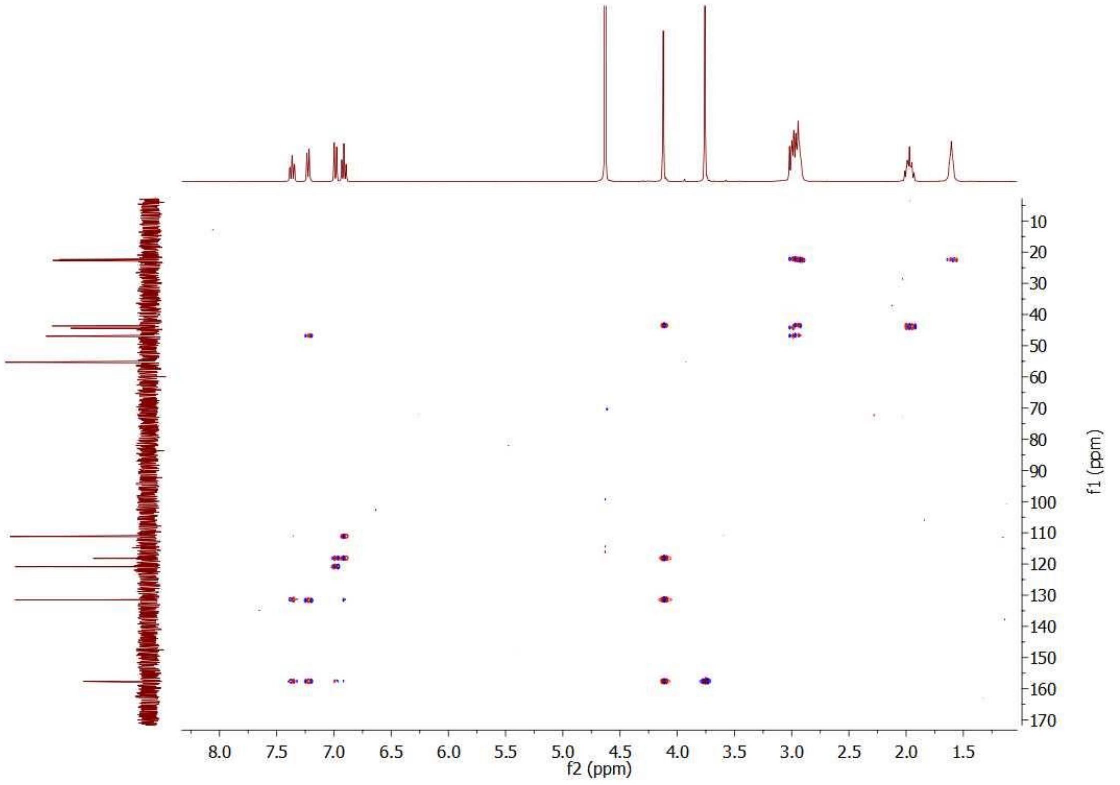

HSQC:

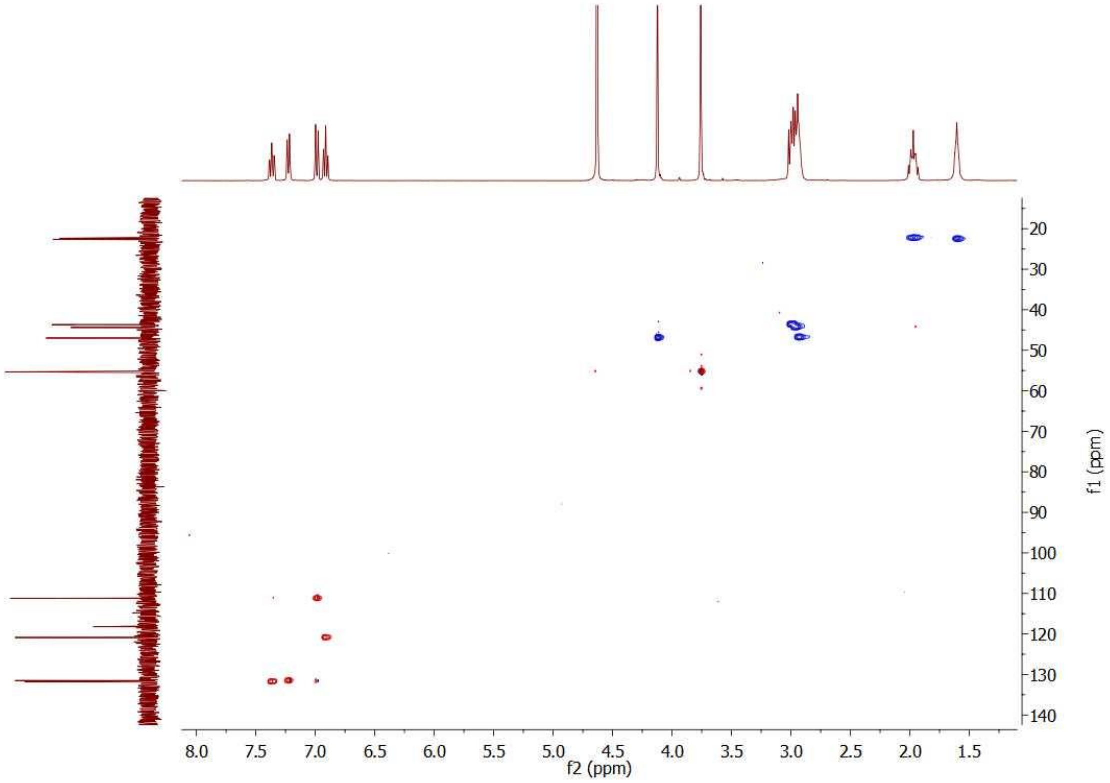

HPLC: t_r = 8.6min Purity >99% at 220nm.

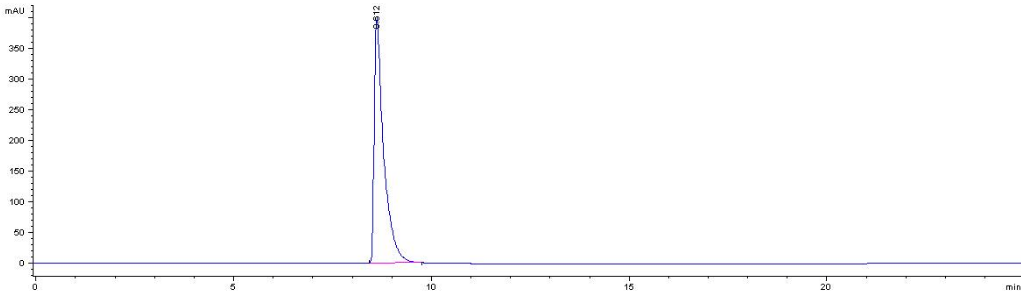

MS: Calculated MS= 442.3308; Found MS= 443.3527 $(M + H)^+$

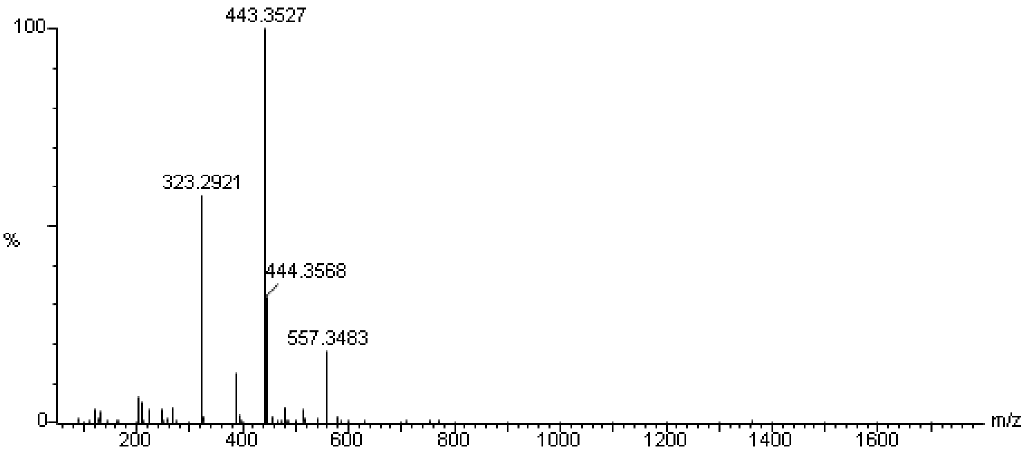

3FF

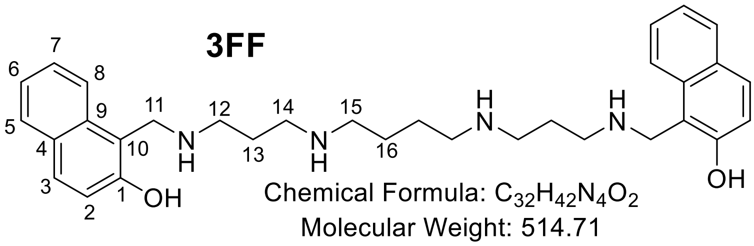

^{1}H RMN (D_2O): δ 7.86 (m, 6H, H3, H5, H8), 7.54 (t, 2H, J_1= 8Hz, H6), 7.36 (t, 2H, J_2=8Hz, H7), 7.18 (d, 2H, J_3=12Hz, H2), 4.65 (s, 2H, H11), 3.14 (m, 4H, H12), 3.00 (m, 8H, H14, H15), 2.04 (m, 4H, H13), 1.62 (m, 4H, H16).

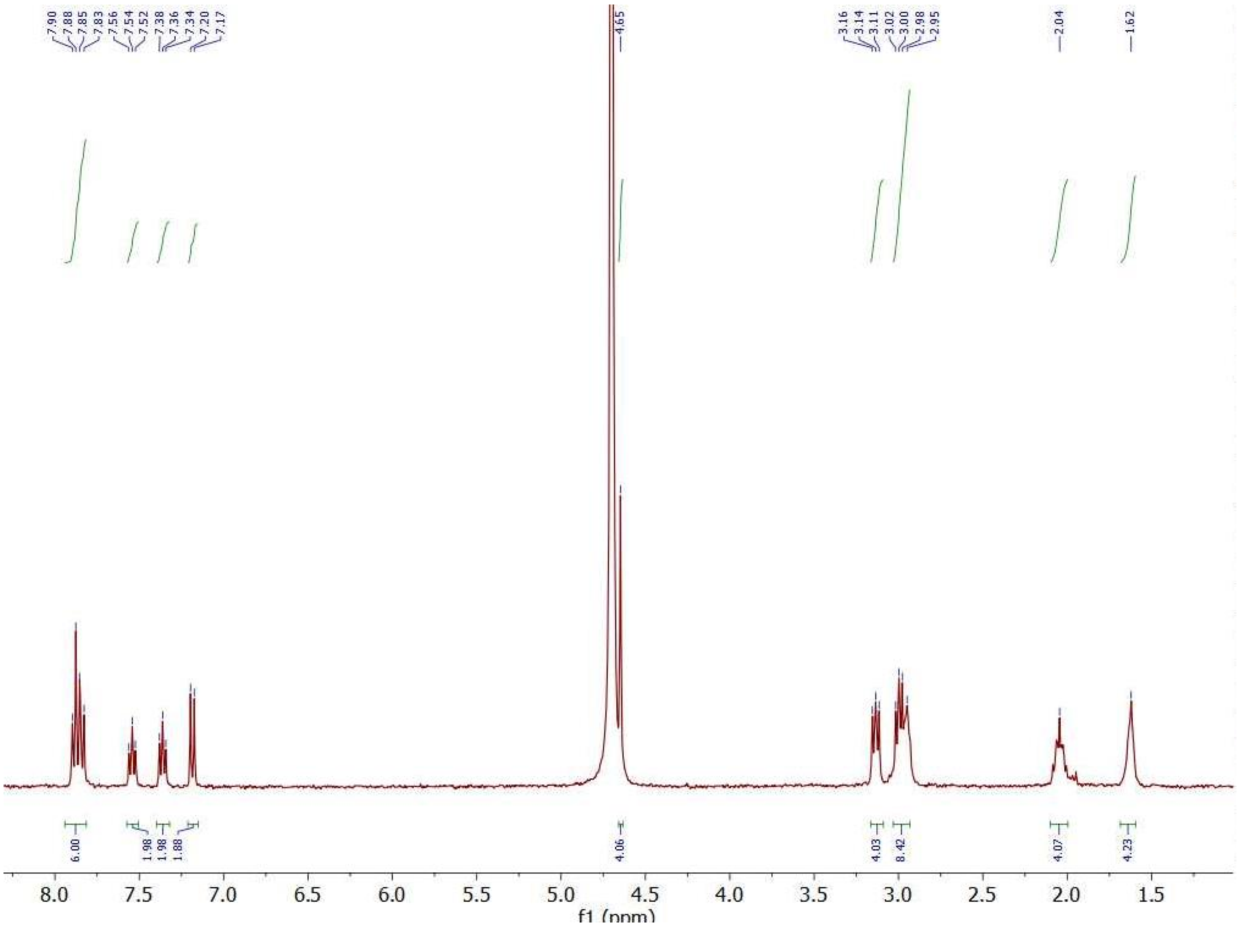

^{13}C RMN (D_2O): 154.1 (C1), 132.4 (C4), 132.1 (C3), 128.9 (C9), 128.4 (C5), 127.8 (C7), 123.7 (C8), 121.6 (C2), 117.3 (C10), 46.8 (C11), 44.4 (C14), 43.8 (C12), 41.6 (C15), 22.6 (C13), 22.4 (C16).

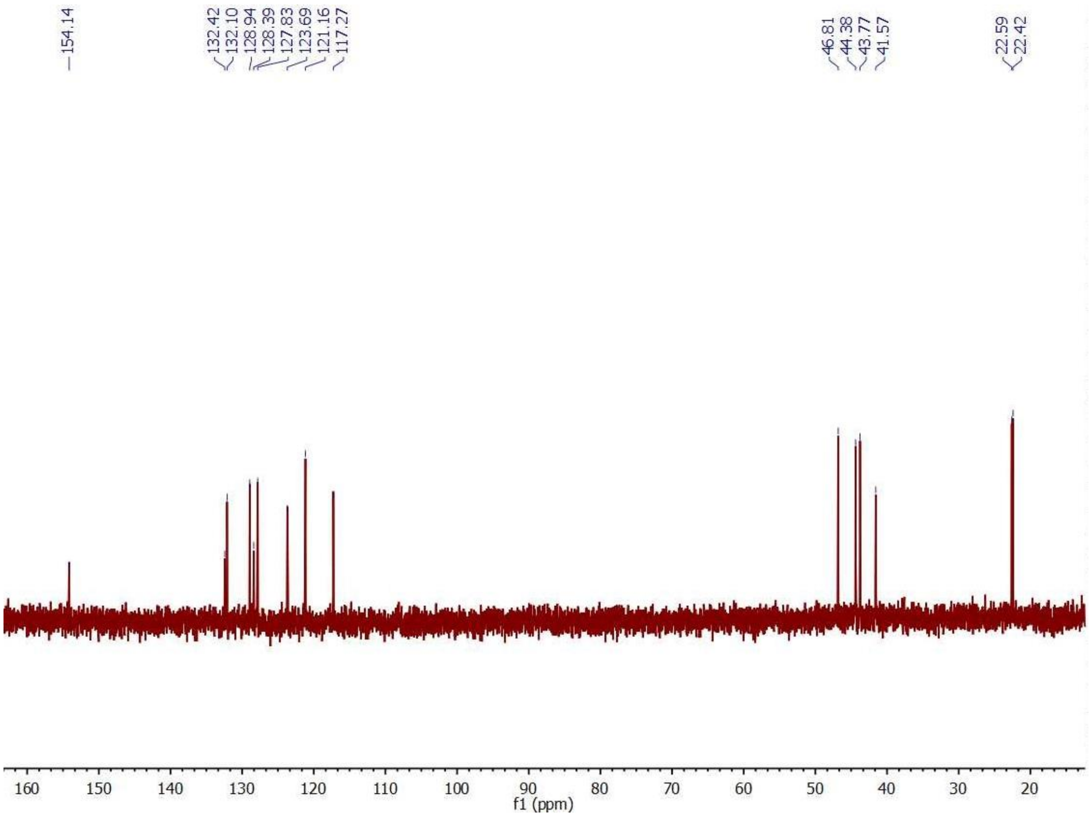

HMBC:

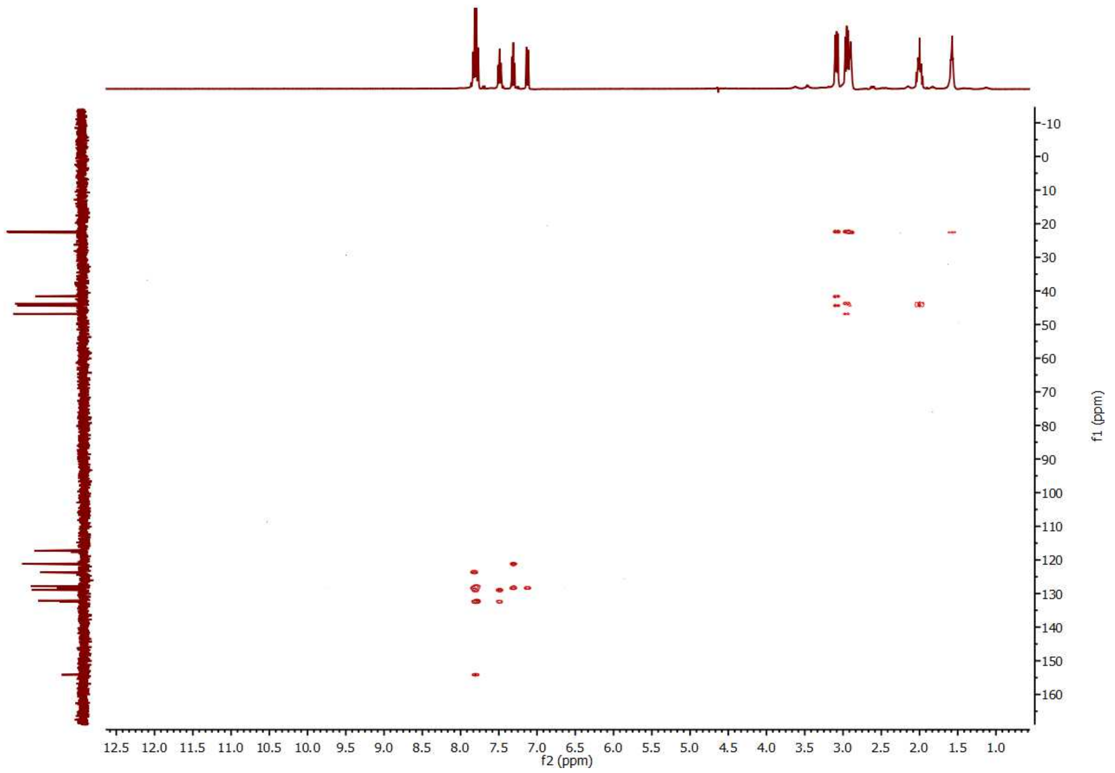

HPLC: t_r = 9.4min Purity 99% at 220nm.

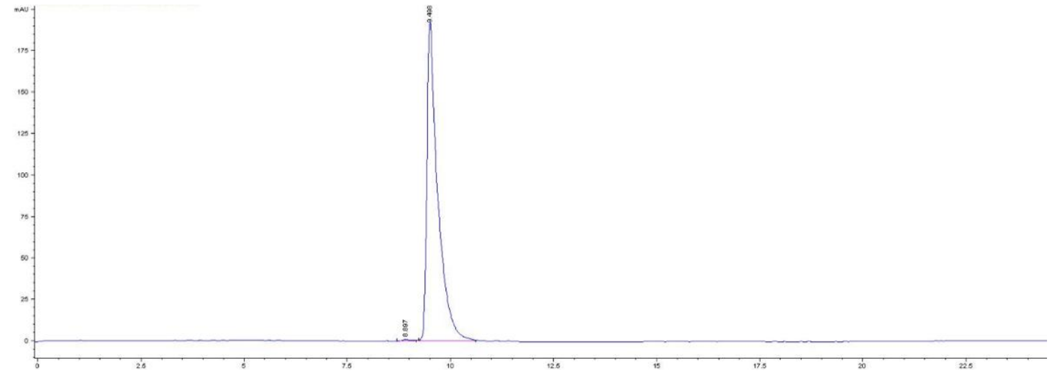

MS: Calculated MS= 514.3308; Found MS= 515.3389 $(M + H)^+$

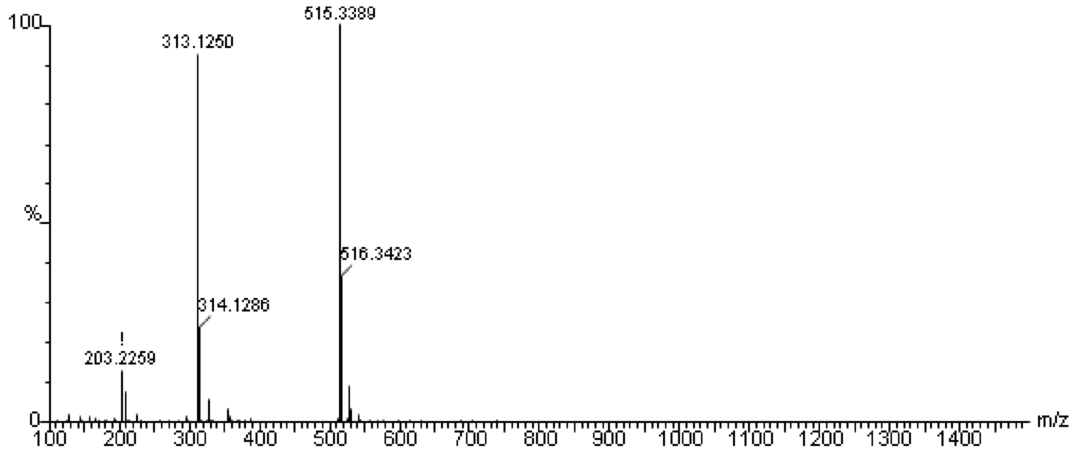

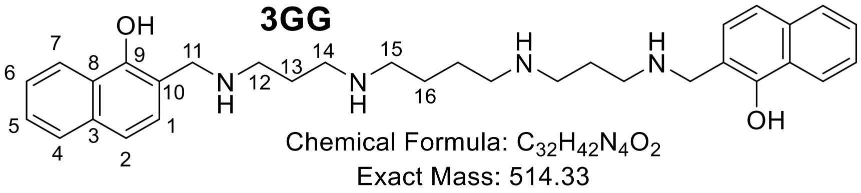

^{1}H RMN (D_2O): δ 8.06 (2H, dd, H7), 7.82 (2H, dd, H4), 7.48 (6H, m, H2, H5, H6), 7.3 (2H, d, H1), 4.33 (4H, s, H11), 3.05 (4H, tr, H12), 2.93 (8H, m, H14, H15), 1.99 (4H, m, H13), 1.57 (4H, m, H16).

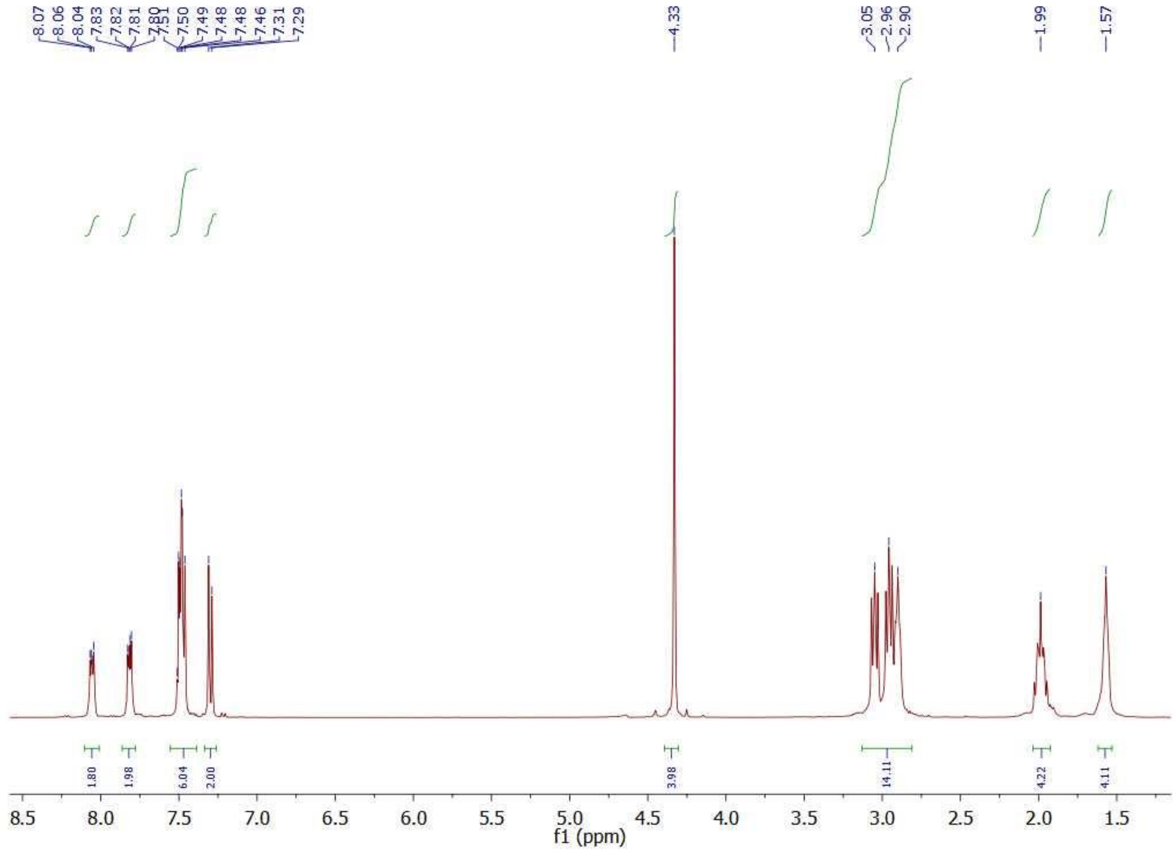

^{13}C RMN (D_2O): δ 150.9 (C9), 135.0 (C8), 128.1 (C4), 127.8 (C2), 127.4 (C6), 126.3 (C1), 124.9 (C5), 121.4 (C7), 121.2 (C3), 113.1 (C10), 46.8 (C11), 44.4 (C15), 43.6 (C14), 22.6 (C13), 22.4 (C16).

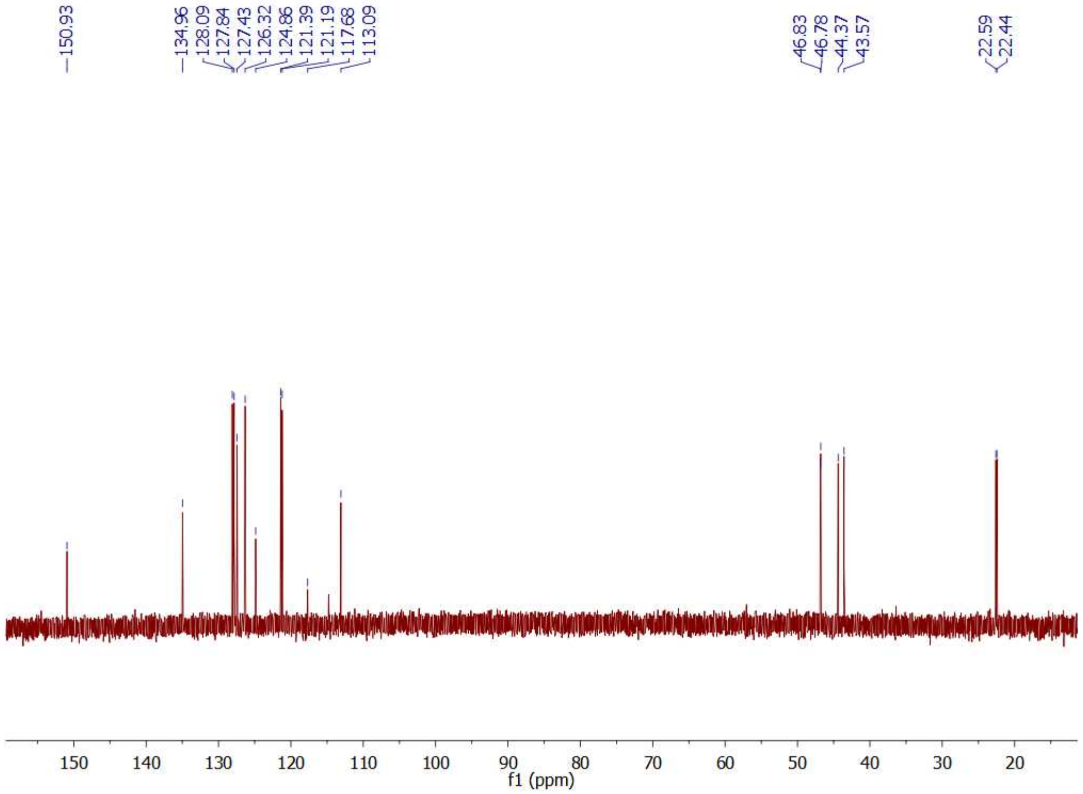

HMBC:

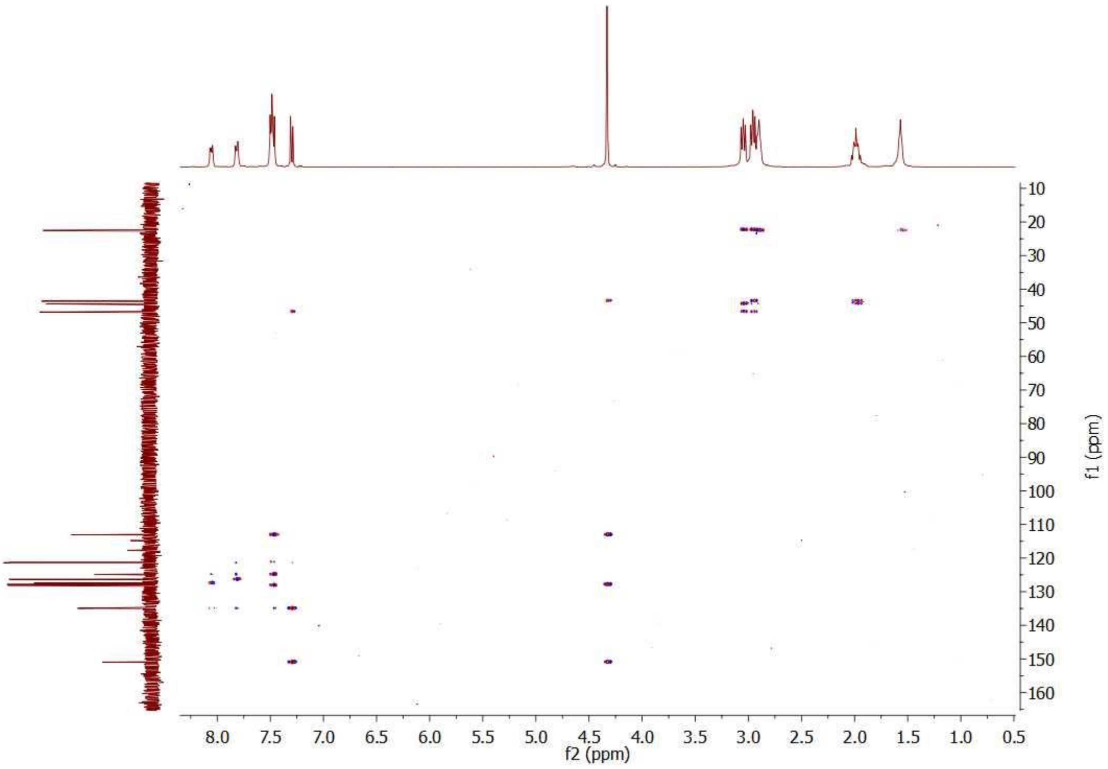

HSQC:

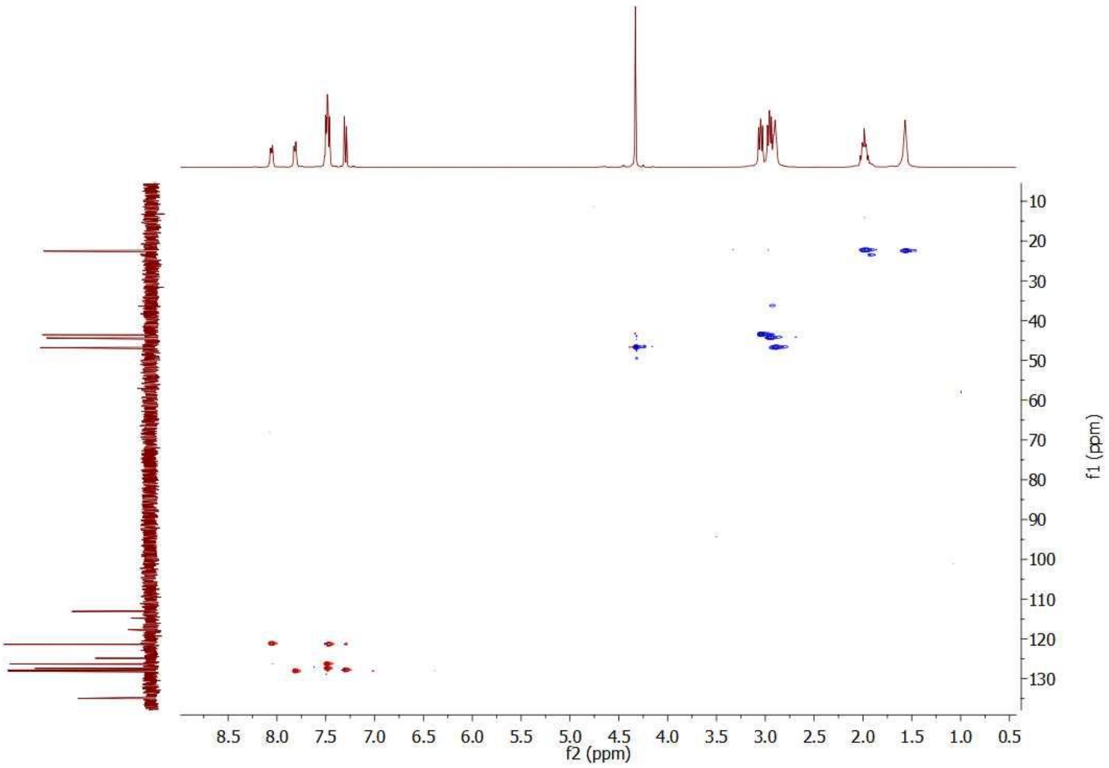

HPLC: t_r = 10.1min, Purity at 220nm = 97.4%

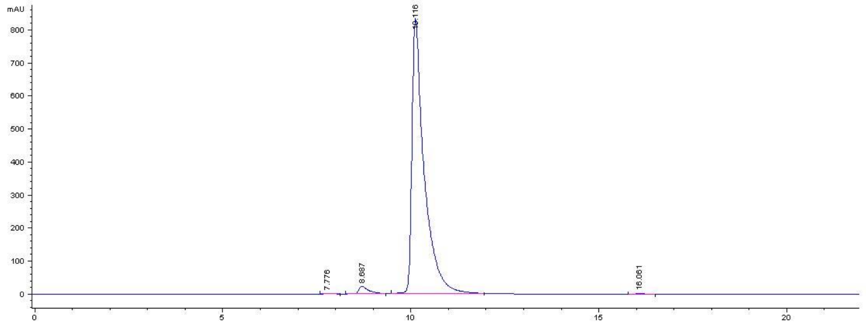

MS: Calculated MS= 514.3308; Found MS = 515.3438 (M + H)$^+$

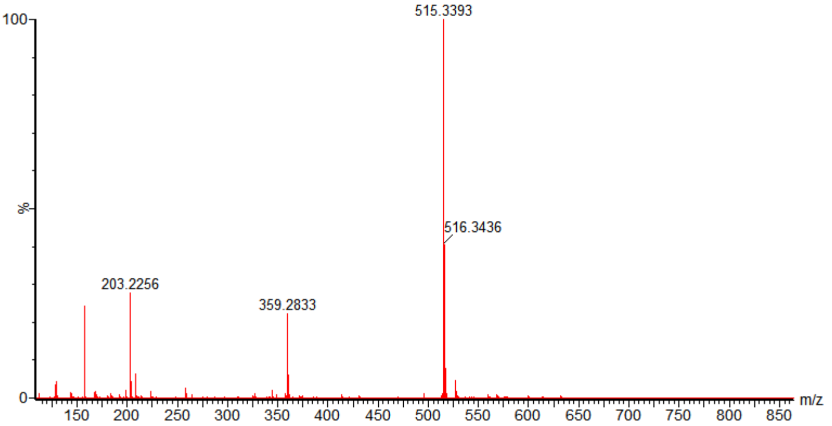

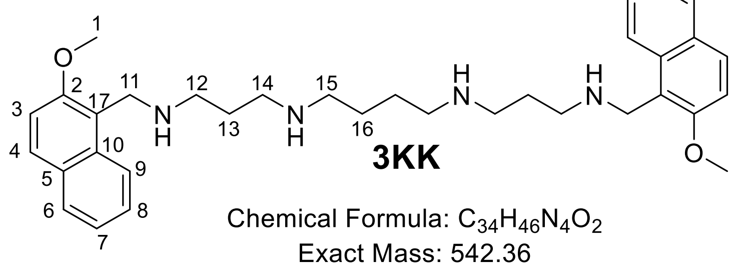

3KK

Chemical Formula: $C_{34}H_{46}N_4O_2$
Exact Mass: 542.36

^{1}H RMN (D_2O): δ 7.98 (2H, d, H4, J_1=8Hz), 7.88 (2H, d, H9, J_2=8Hz) 7.84 (2H, d, H6, J3=8Hz), 7.52 (2H, dd, H7, J_1=J_4=8Hz), 7.37 (4H, m, H8, H3), 4.6 (H11), 3.9 (6H, s, H1), 3.1 (4H, m, H15), 2.93 (8H, m, H12, H14), 1.99 (4H, m, H13), 1.58 (4H, m, H16).

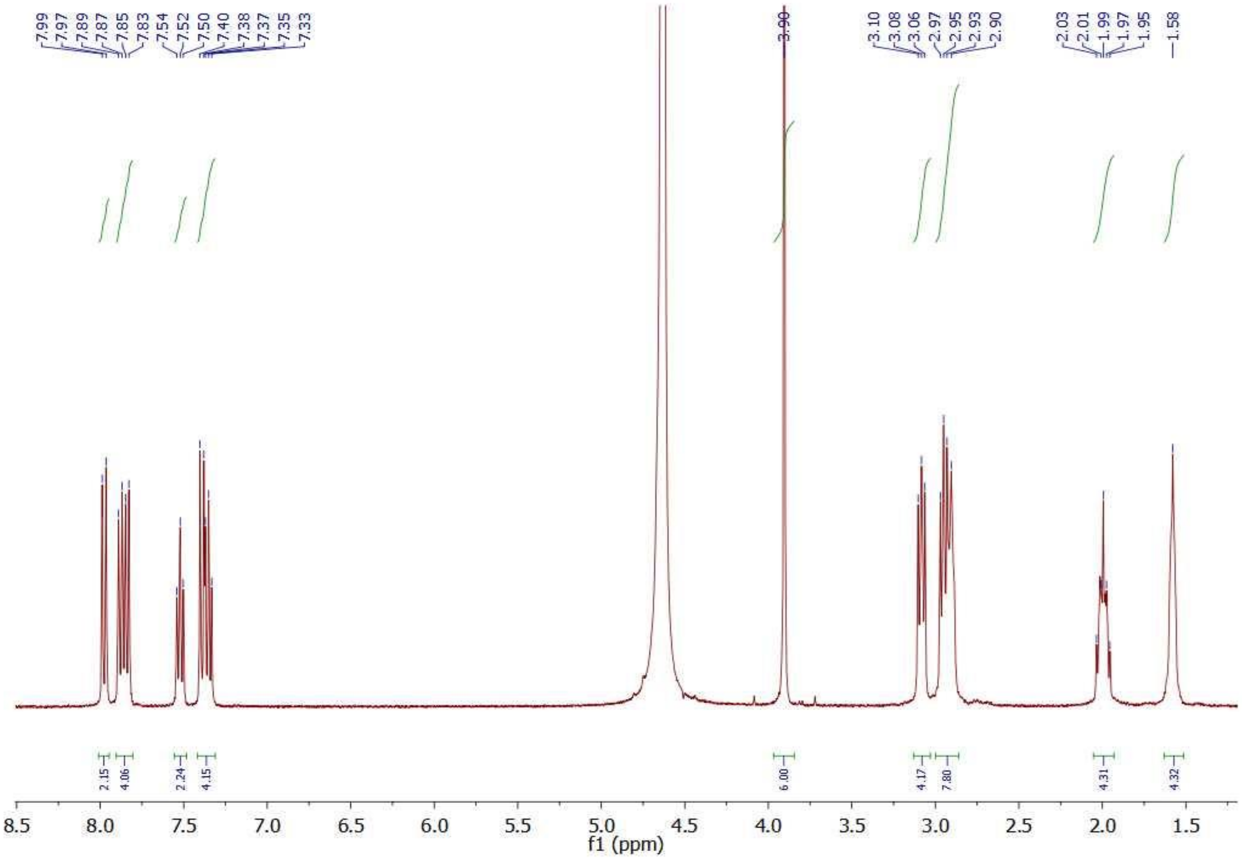

^{13}C RMN (D_2O): δ 156.5 (C2), 132.4 (C4), 132.1 (C5), 129.0 (C6), 128.6 (C10), 128.0 (C7), 124.1 (C8), 121.3 (C9), 112.9 (C3), 110.2 (C17), 56.1 (C1), 46.9 (C12), 44.4 (C14), 43.9 (C15), 41.6 (C11), 22.6 (C13), 22.5 (C16).

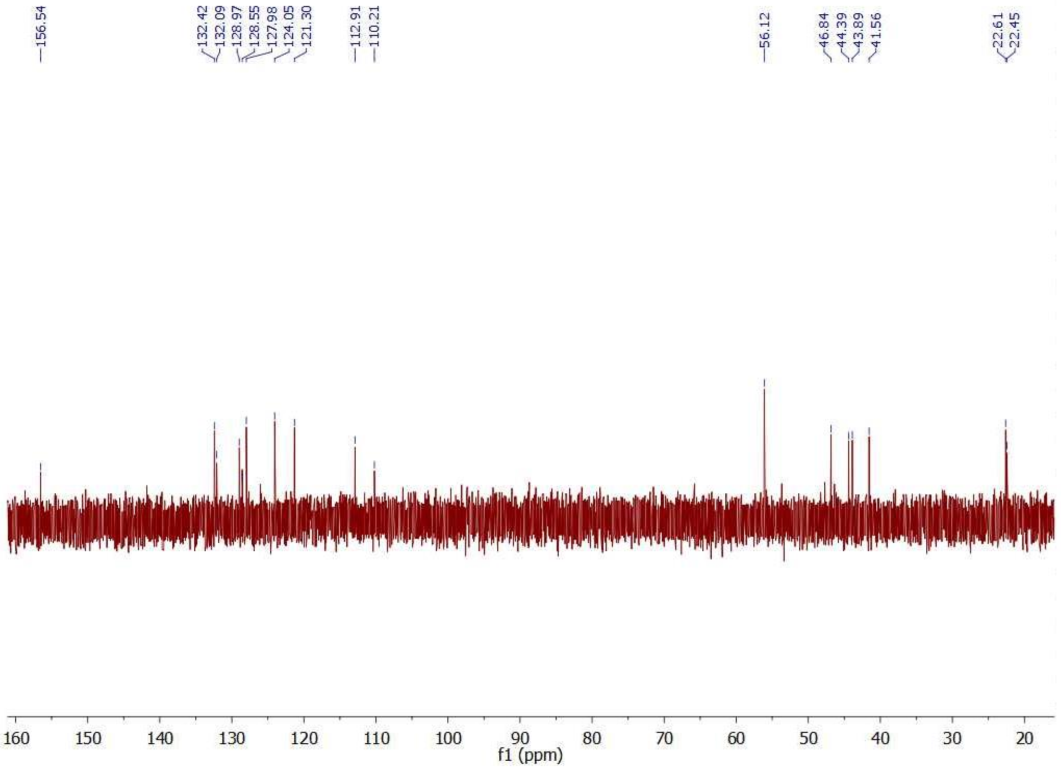

HMBC:

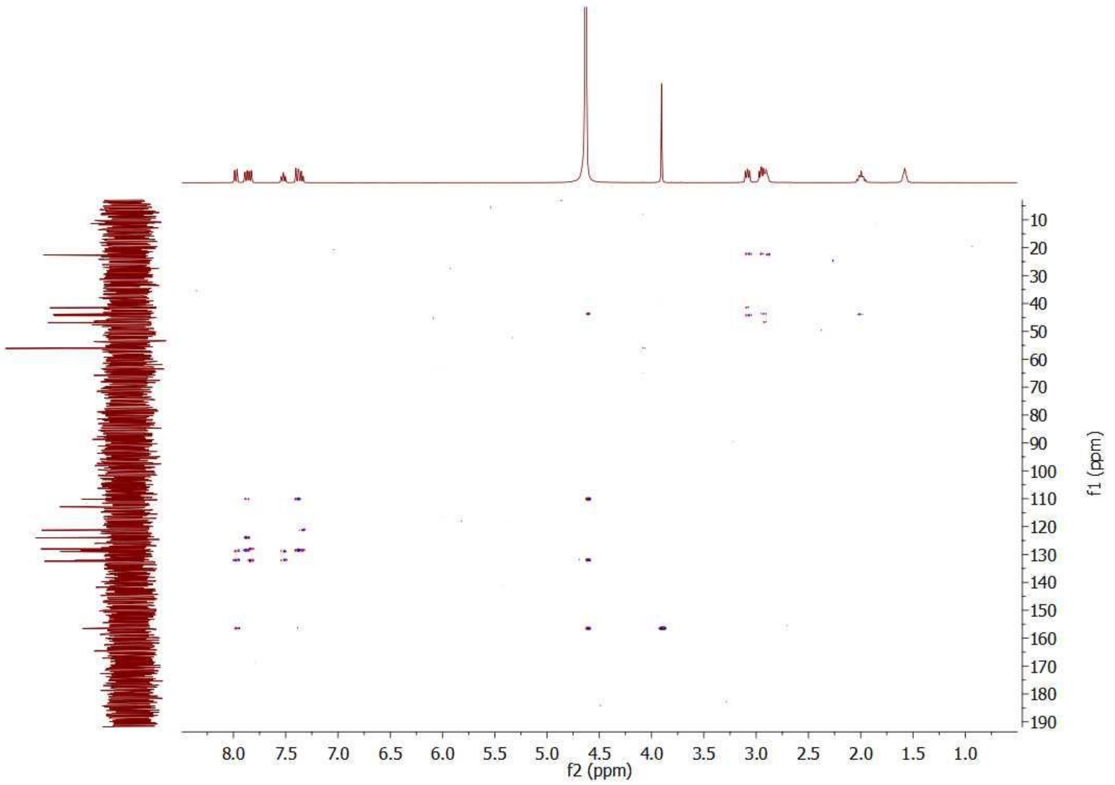

HSQC:

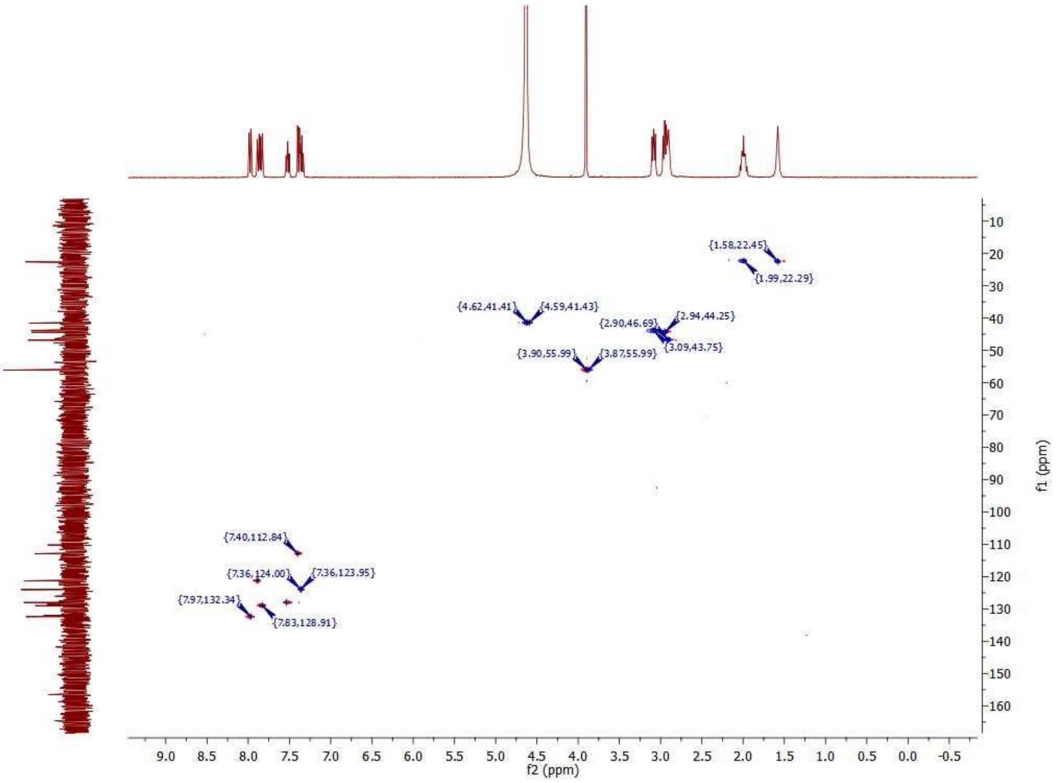

MS: Calculated MS= 542.3621; Found MS = 543.3602 $(M + H)^+$

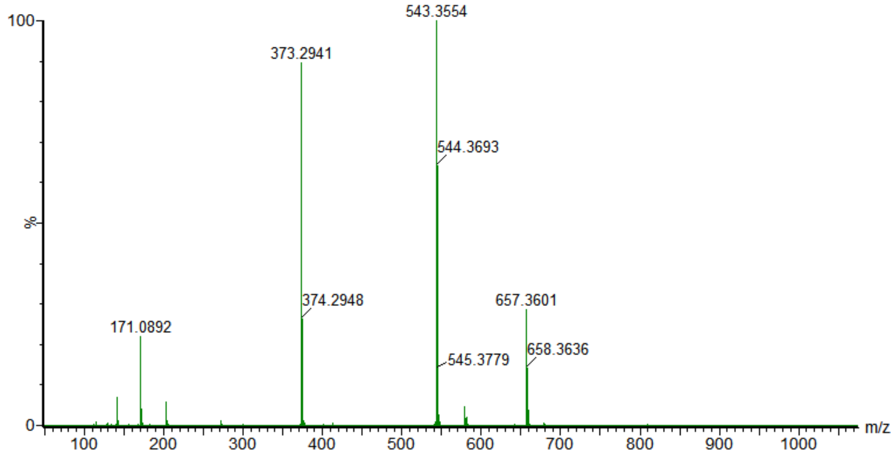

HPLC: t_r = 10.3min, Purity at 220nm = 99.0%

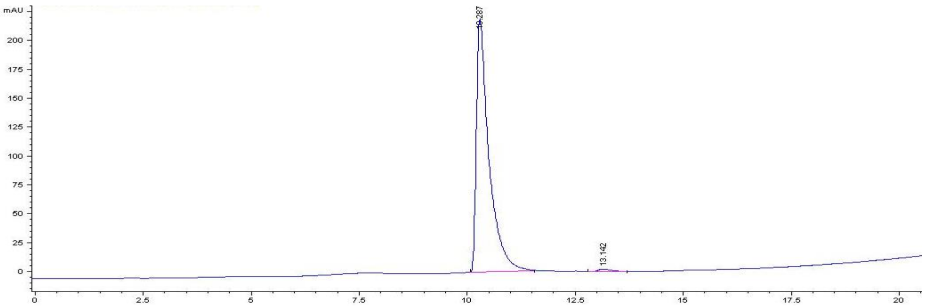

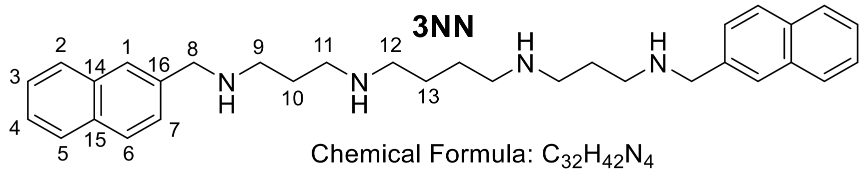

Chemical Formula: $C_{32}H_{42}N_4$
Exact Mass: 482.34

^{1}H RMN (D_2O): δ 7.86 (8H, m, H2, H5, H1, H6), 7.49 (4H, m, H3, H4), 7.42 (2H, dd, J1=8.5Hz, J2=1.5Hz, H7), 4.28 (4H, s, H8), 2.99 (12H, m, H9, H11, H12), 1.96 (4H, m, H10), 1.57 (4H, m, H13).

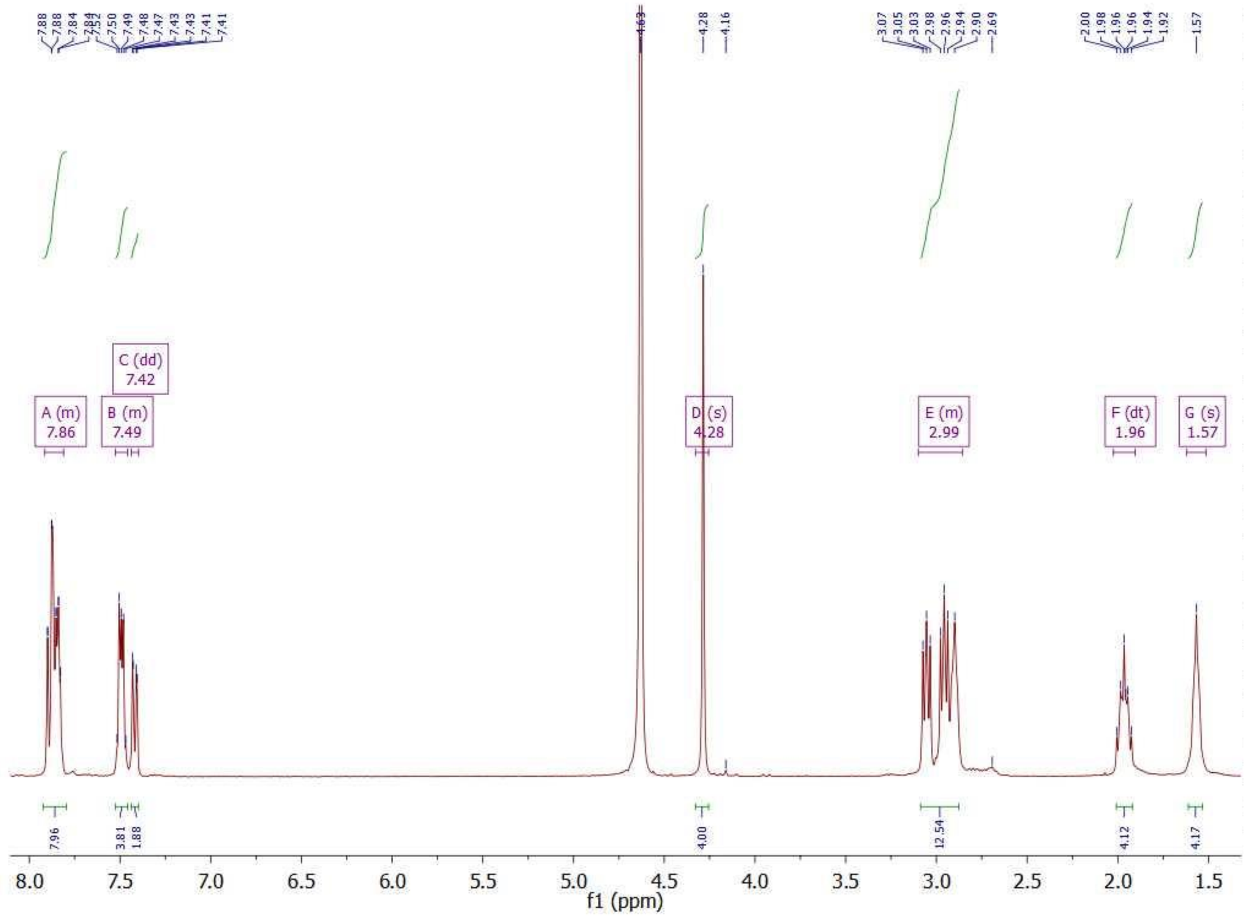

^{13}C RMN (D_2O): δ 133.1 (C15), 132.7(C14), 129.6(C1), 129.0 (C2), 127.9 (C5), 127.7 (C6), 127.4 (C4), 127.0 (C3), 126.4 (C7), 117.7 (C16), 51.3 (C8), 46.9 (C9), 44.4 (C12), 43.8 (C11), 22.6 (C13), 22.5 (C10)

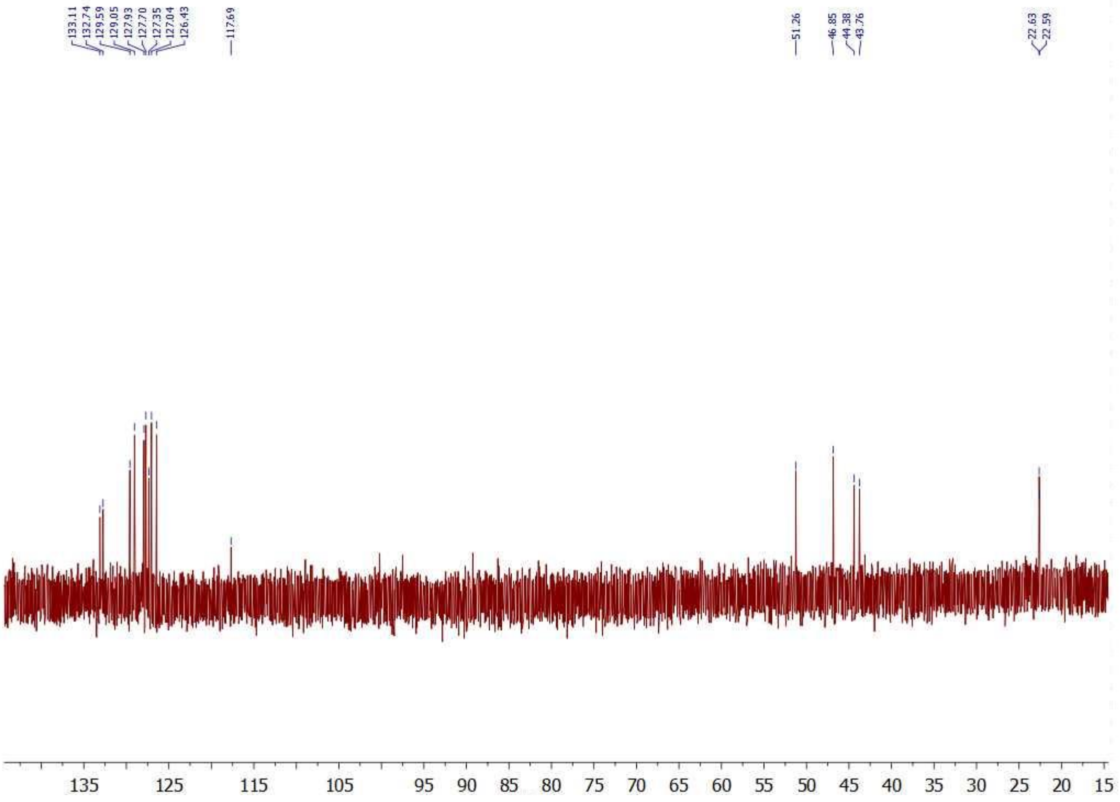

HMBC:

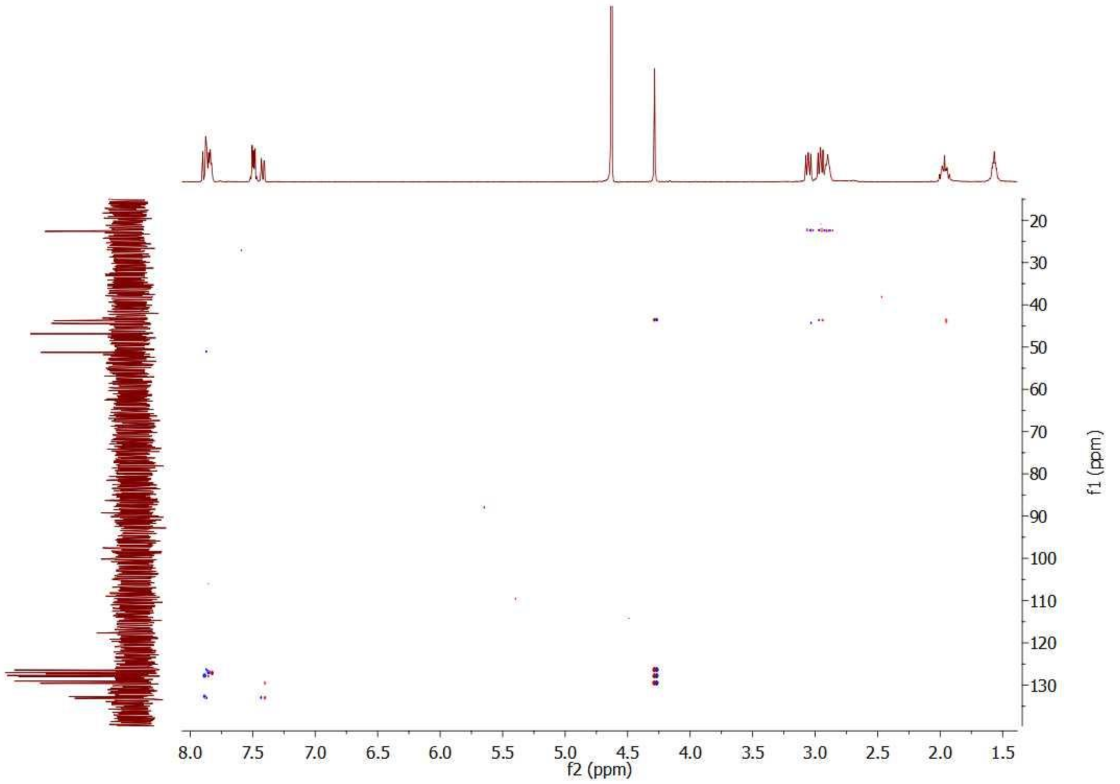

HSQC:

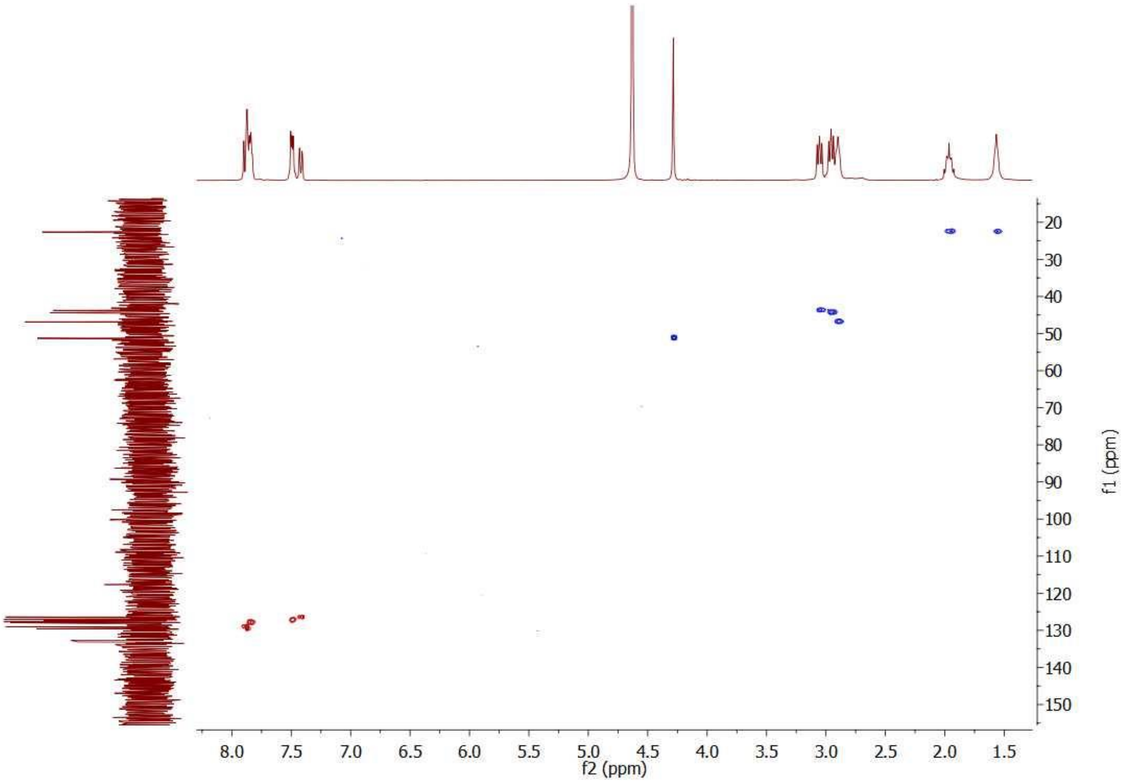

HPLC: t_r = 10.74min. Purity at 220nm = 94.5%

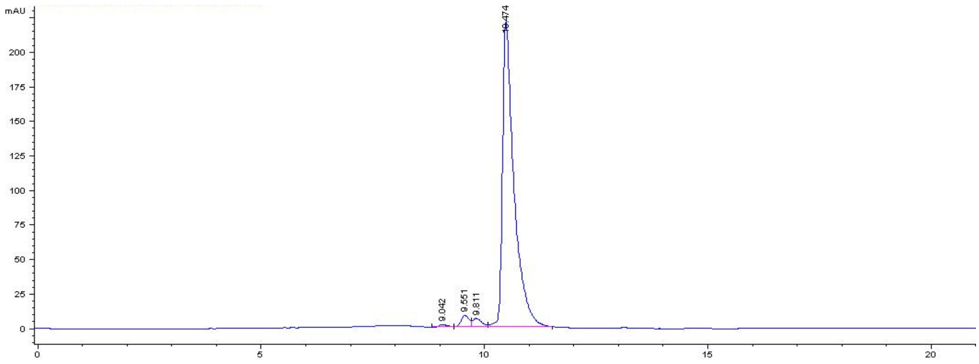

MS: Calculated MS= 482.3409; Found MS = 483.3493 $(M + H)^+$

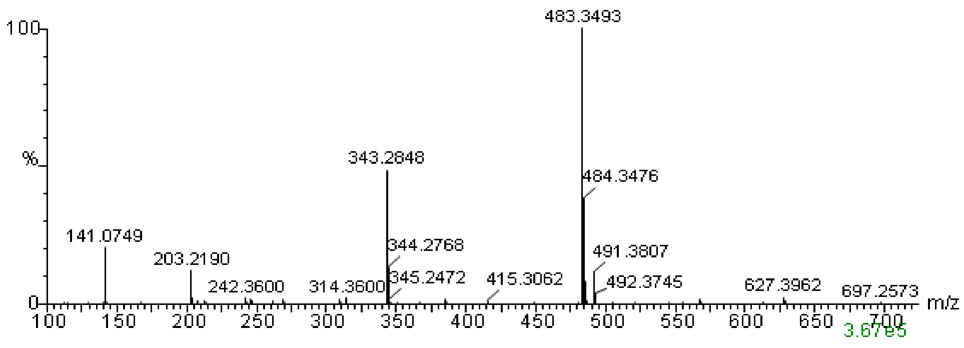

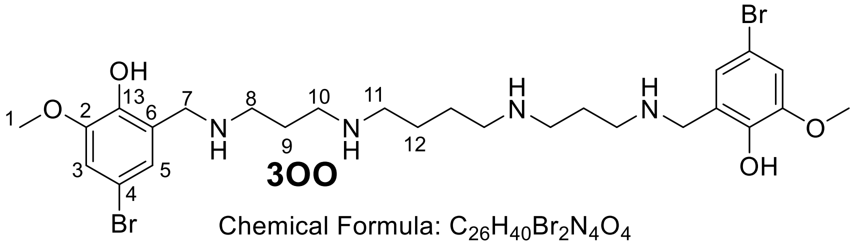

3OO

Chemical Formula: $C_{26}H_{40}Br_2N_4O_4$
Molecular Weight: 632.44

^{1}H RMN (D_2O): δ 7.16 (2H, s, H5), 6.98 (2H, s, H3), 4.1 (4H, s, H7), 3.74 (6H, s, H1), 2.98 (12H, m, H8, H10, H11), 1.97 (4H, m, H9), 1.61 (4H, m, H12)

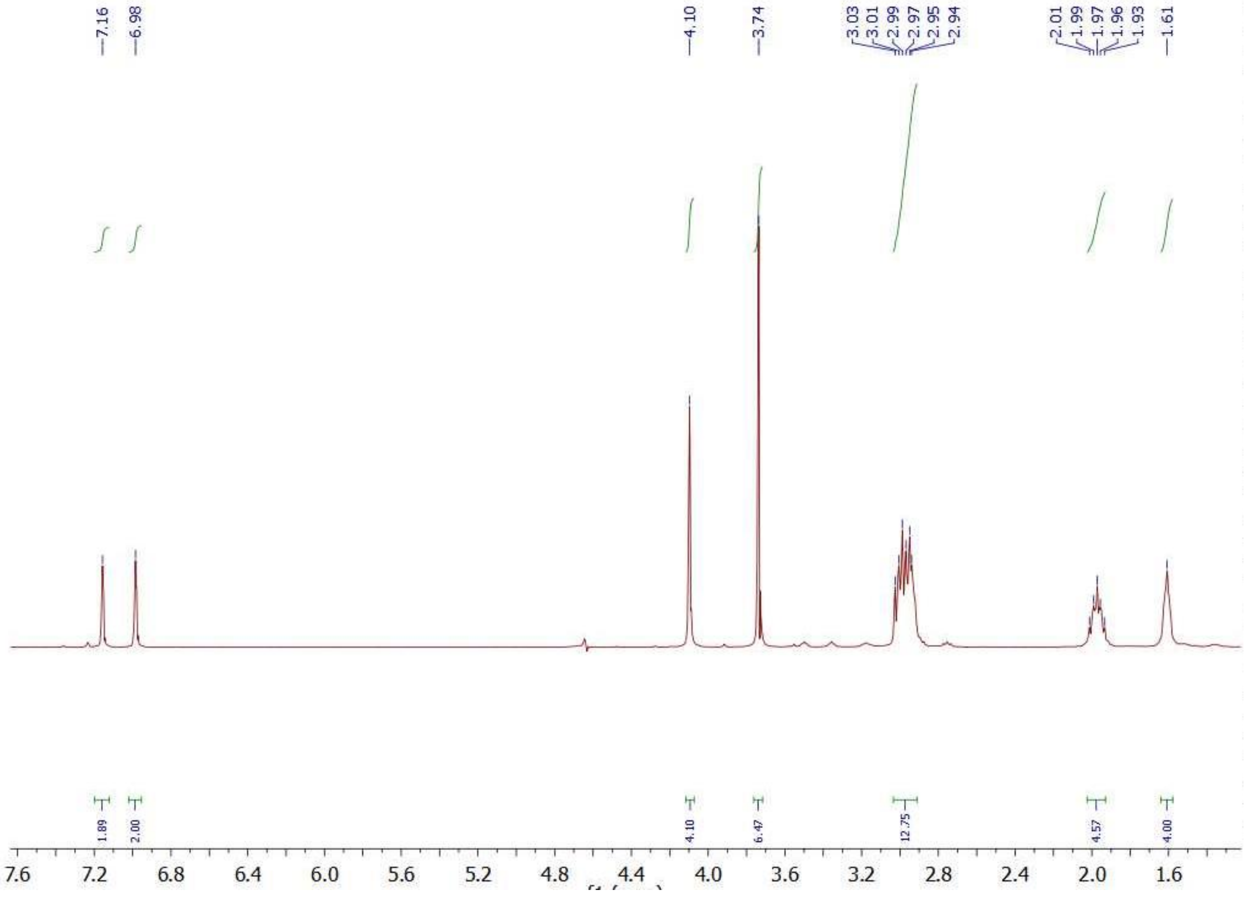

^{13}C RMN (D_2O): 148.4 (C2), 144.0 (C13), 125.2 (C3), 118.5 (C6), 116.6 (C5), 111.1 (C4), 56.3 (C1), 46.8 (C7), 45.8 (C8), 44.4 (C10), 43.6(C12), 22.6 (C9), 22.4 (C12)

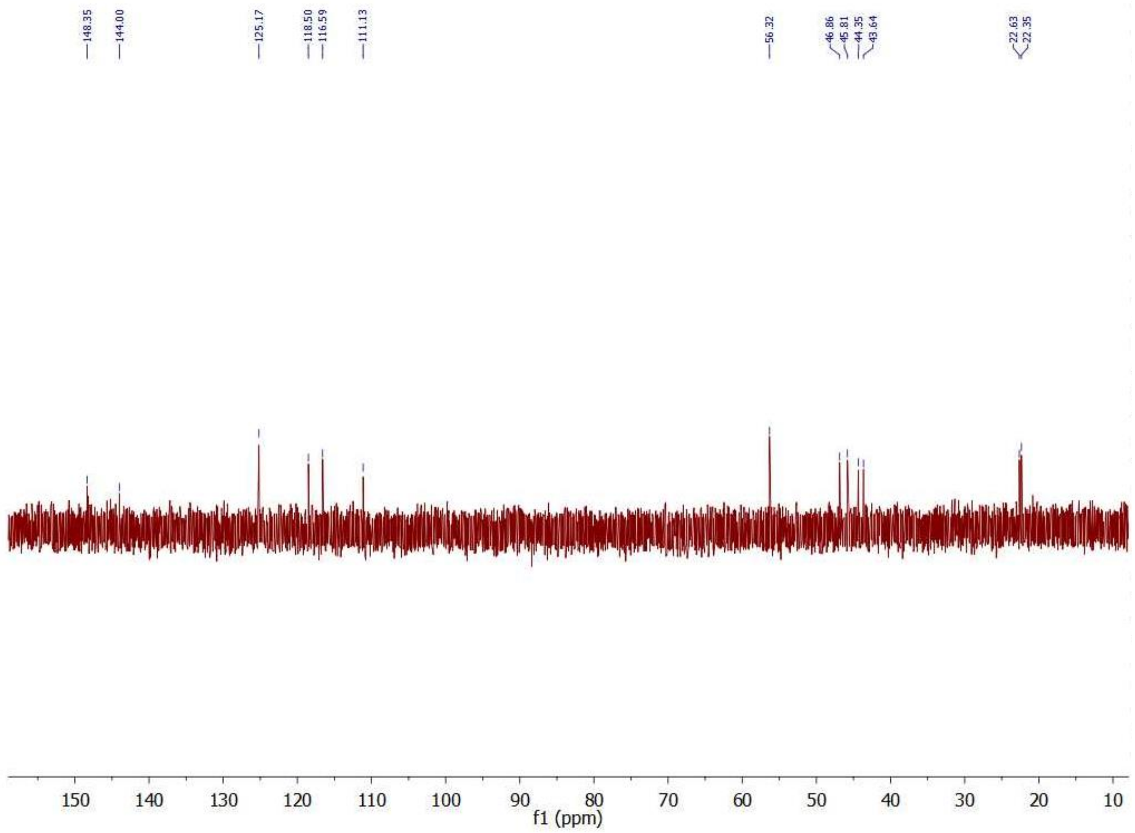

HSQC:

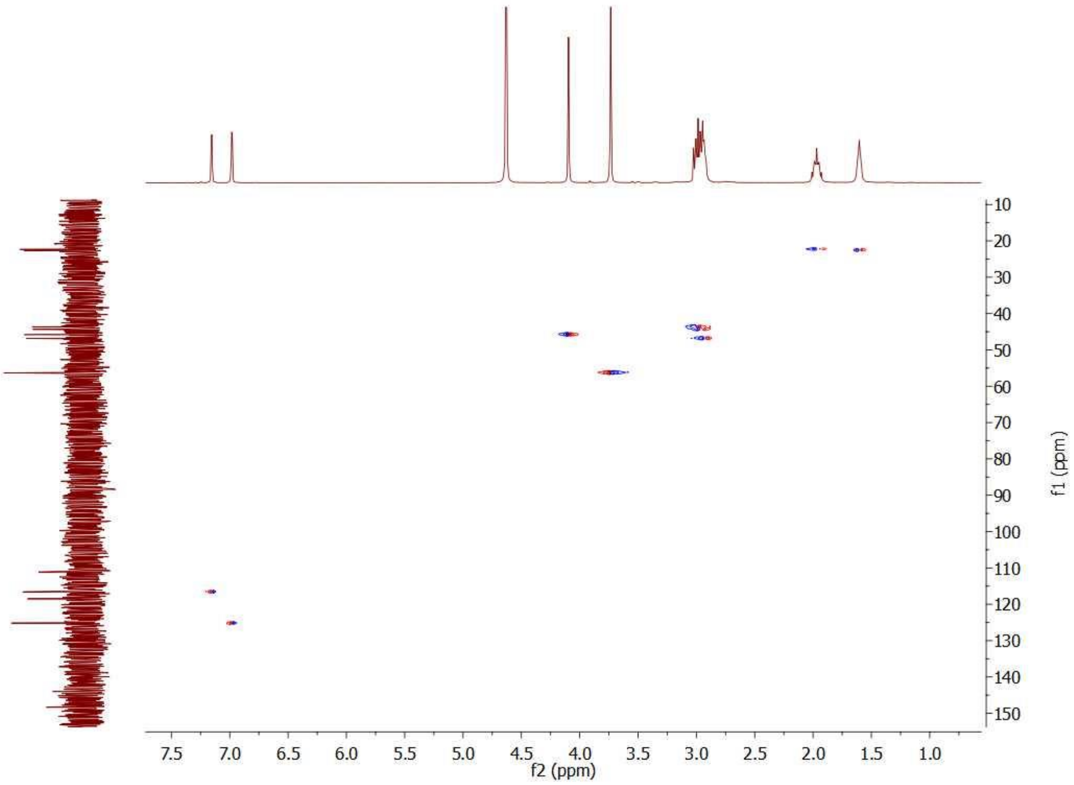

HMBC:

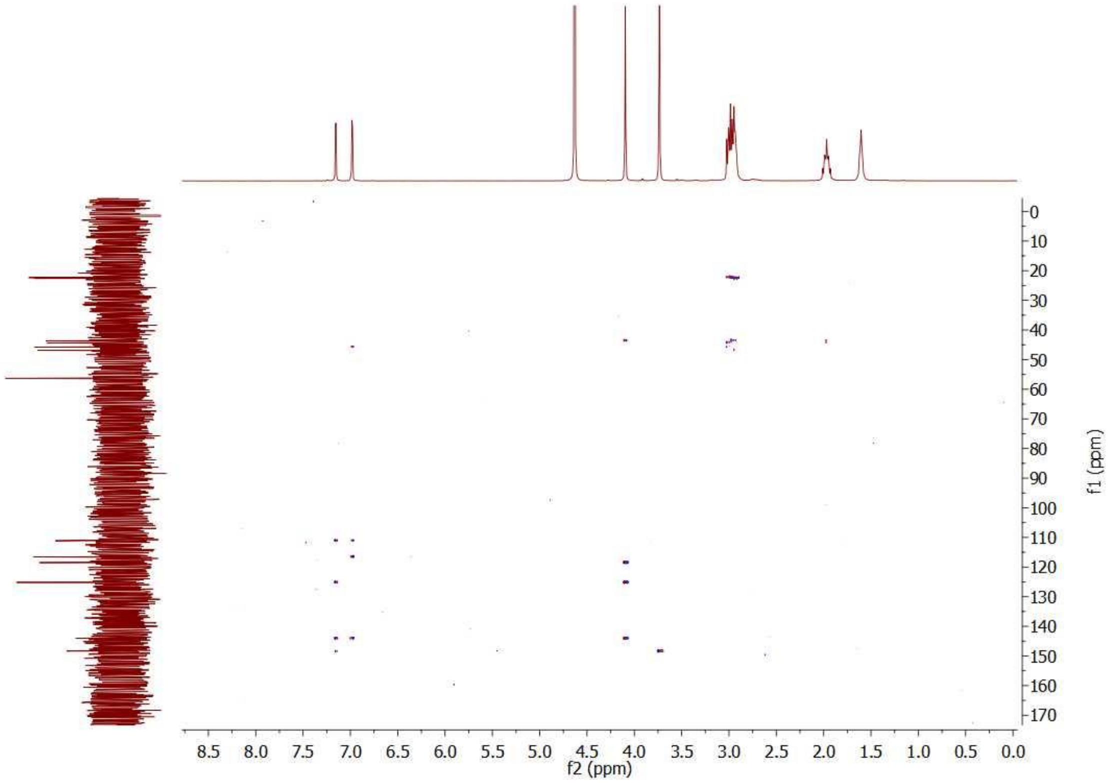

HPLC: t_r = 13.7min Purity >99% at 220nm.

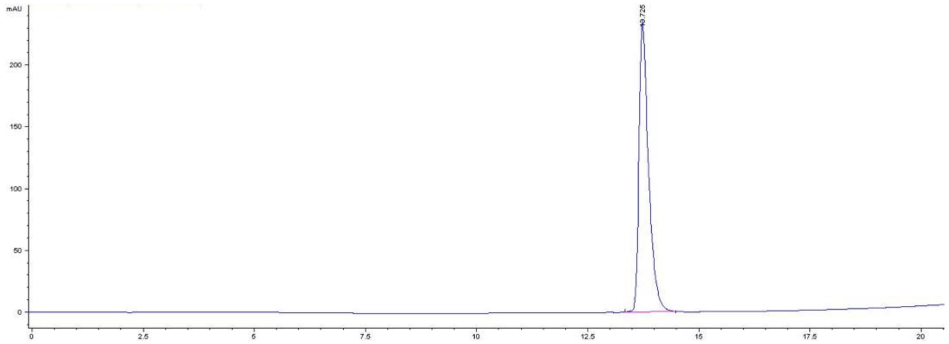

MS: Calculated MS= 630.1416, 632.1396; Found MS = 631.3065 and 633.2315 $(M + H)^+$

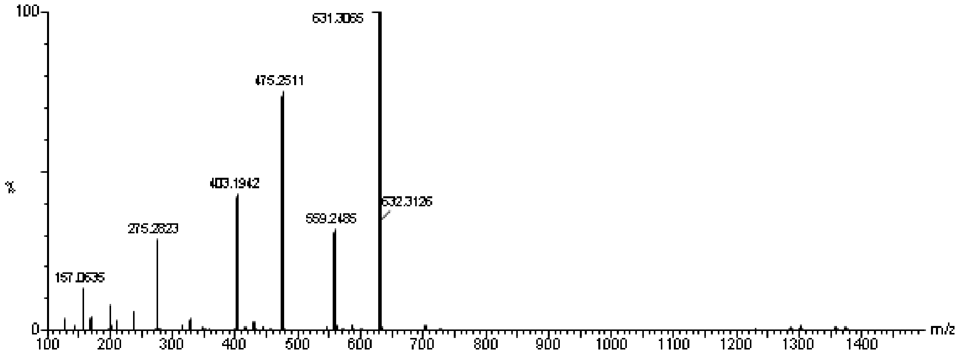

3A

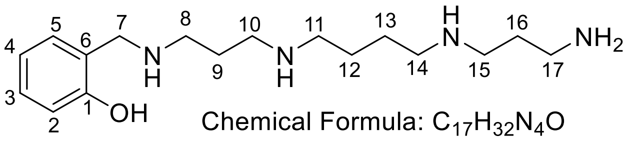

Chemical Formula: $C_{17}H_{32}N_4O$
Molecular Weight: 308.47

^{1}H RMN (D_2O): δ 7.2 (2H, m, H3, H5), 6.85 (2H, m, H2, H4), 4.11 (2H, s, H7, inside water signal), 2.97 (12H, m, H8, H10, H11, H14, H15, H17), 1.94 (4H, m, H9, H16), 1.61 (4H, m, H12, H14).

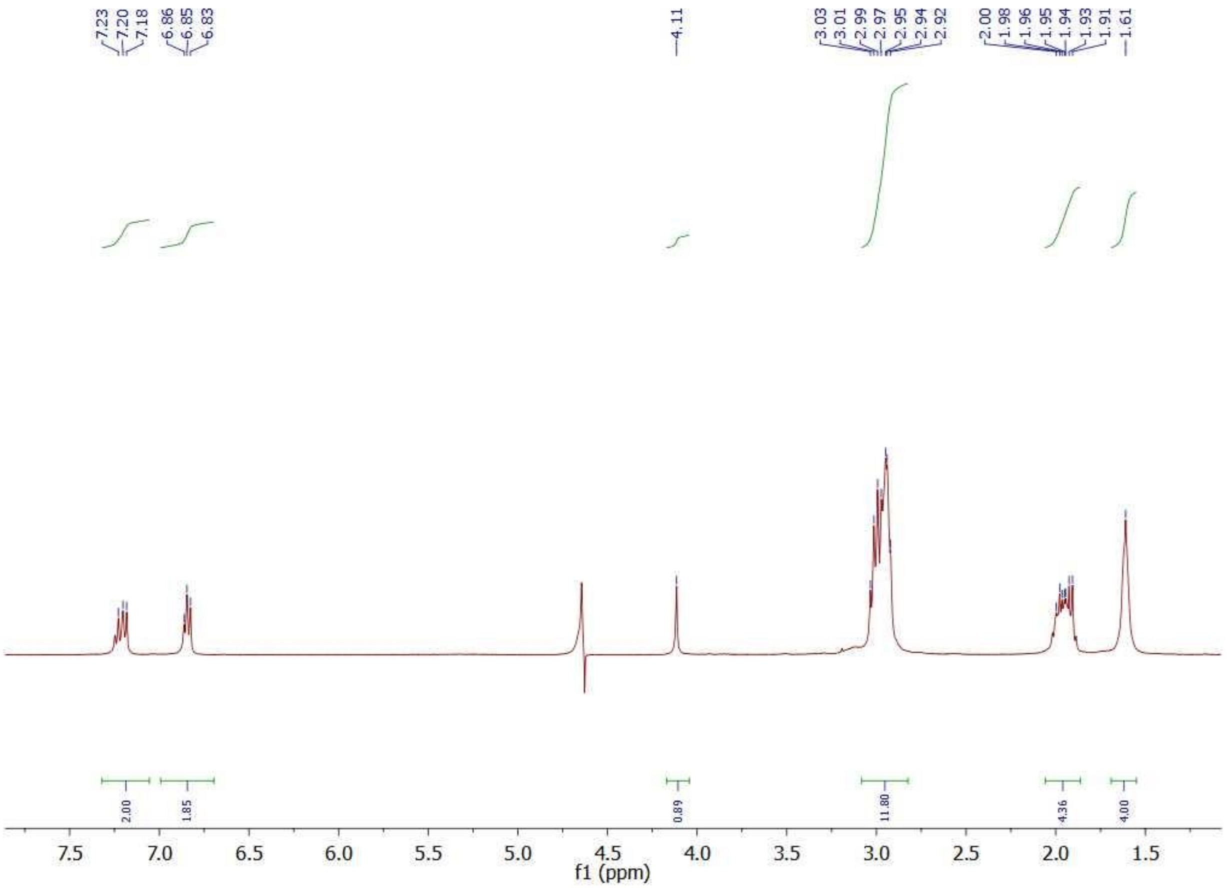

^{13}C RMN (D_2O): δ 154.9 (C1), 131.7 (C5), 131.5 (C3), 120.6(C4), 115.5 (C2), 114.8 (C6), 46.9 (C7), 44.4 (C11, C14, C15, C8), 43.6 (C17), 36.4 (C16), 23.6 (C9), 22.6 (C13), 22.4(C12).

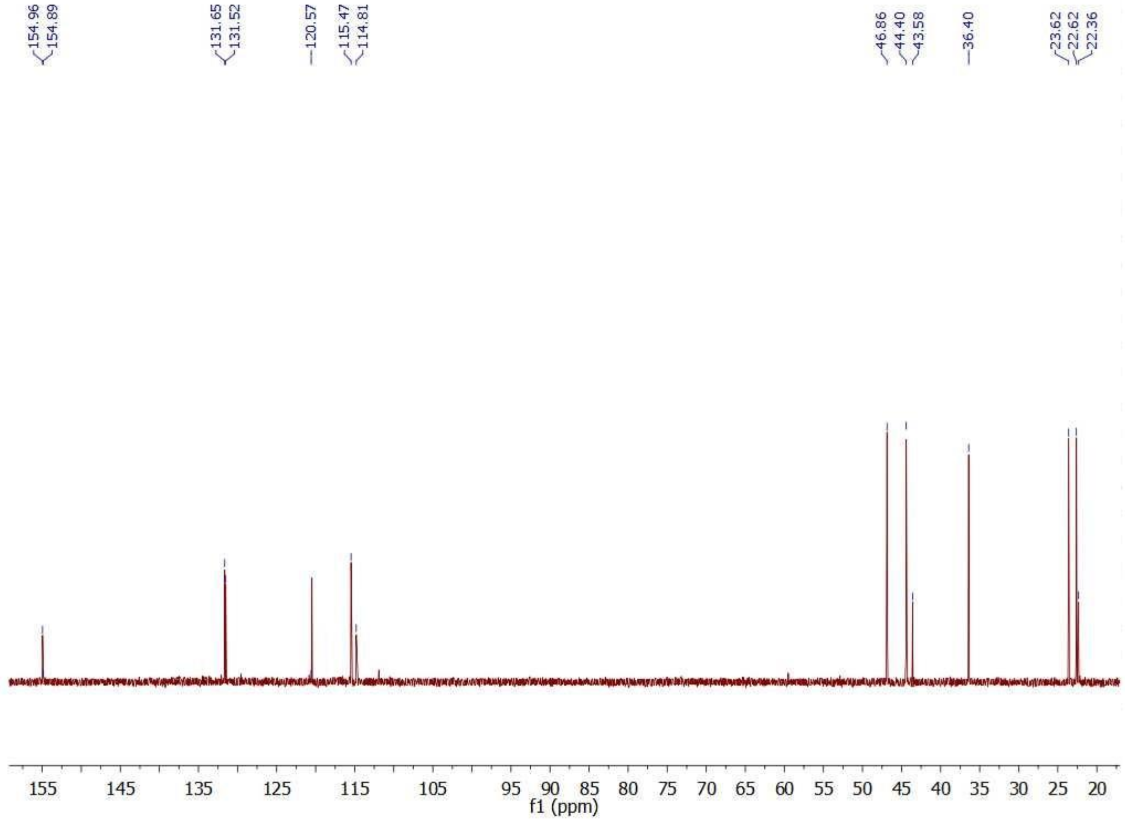

HMBC:

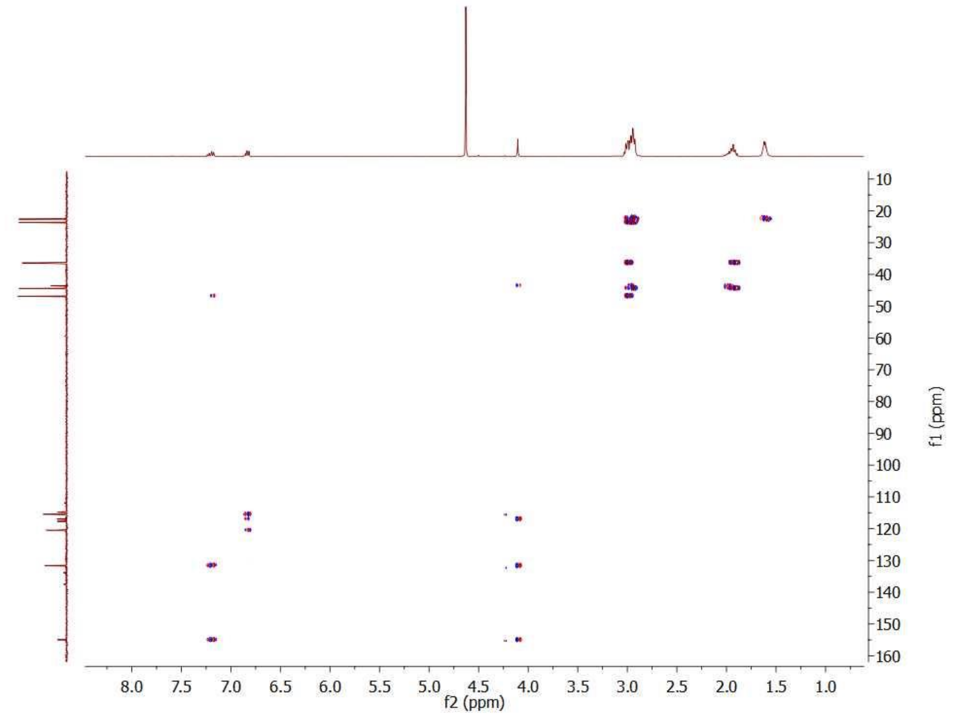

HSQC:

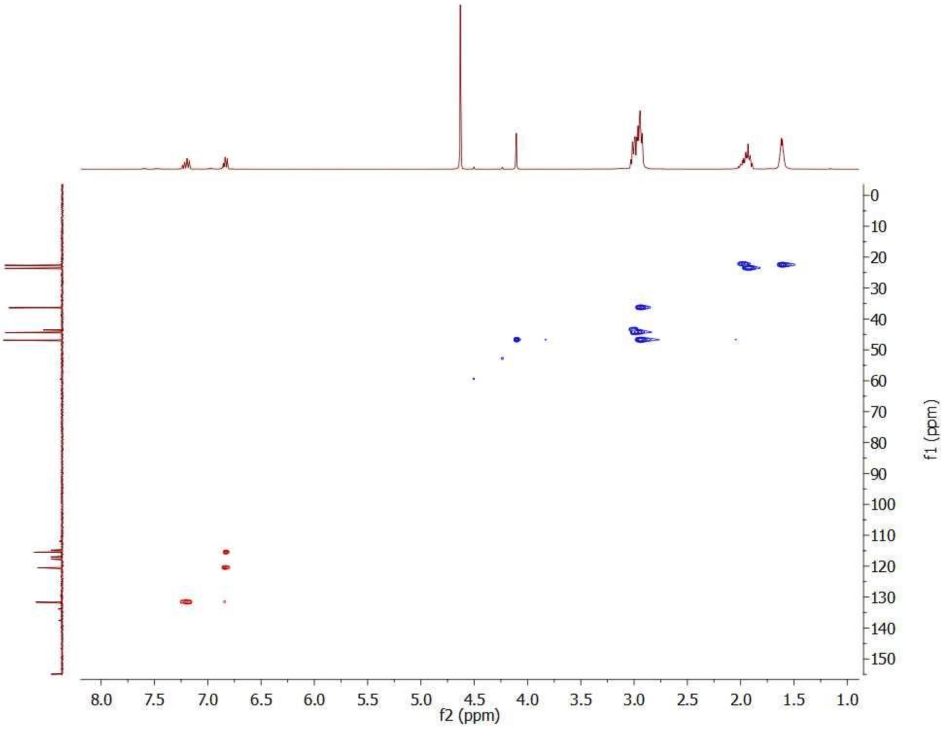

HPLC: t_r = 7.2min, Purity at 220nm = 91.1%

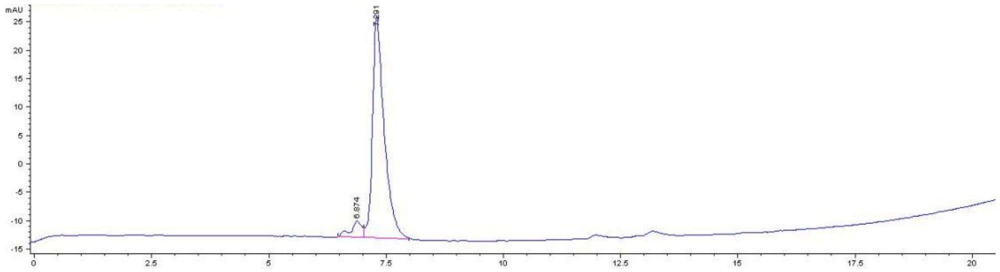

MS: Calculated MS= 308.2576; Found MS = 309.2684 $(M + H)^+$

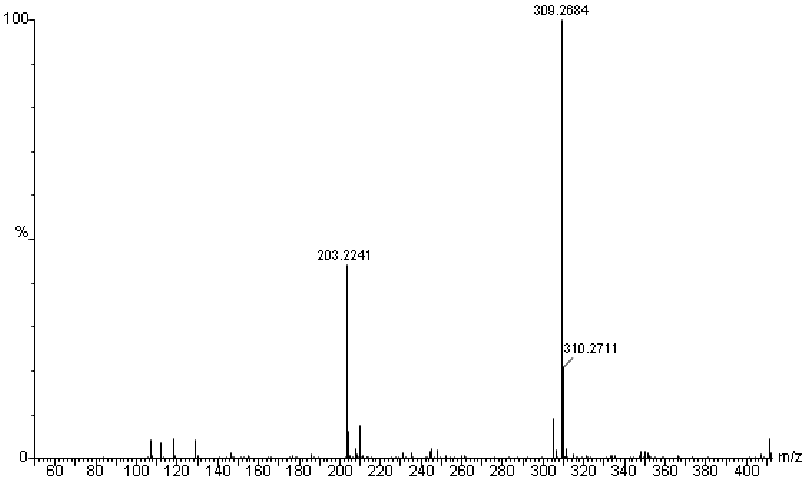

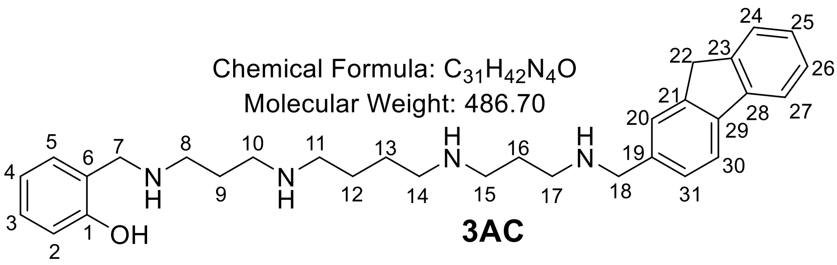

3AC

2-(16-(9H-fluoren-3-yl)-2,6,11,15-tetraazahexadecyl)phenol

3A (30mg, 0.097mmol) was solved in 5mL of Methanol at 0ºC. 9H-fluorene-2-carbaldehyde (110mg, 0.580mmol) was added solved in 15mL of Methanol. The day after, $NaBH_3CN$ (70mg, 1.15mmol) was added and the reaction stirred overnight. Reaction was stopped by addition of 2mL of H_2O. Solvent was removed and crude purified by reverse phase chromatography with a gradient of ACN (0.1% TFA) and water (0.1% TFA). 5mg of **3AC** were obtained as a white powder (Purity >99%, Yield = 12%)

^{1}H RMN (H_2O/D_2O): δ 7.82 (2H, m, H27, H30), 7.56 (2H, m, H20, H24), 7.25 (3H, m, H20, H25, H26), 7.21 (2H, m, H3, H5), 6.88 (2H, m, H2, H4), 4.18 (2H, s, H18), 4.14 (2H, s, H7), 3.85 (2H, s, H22), 3.00 (12H, m, H8, H10, H11, H14, H15, H17), 2.01 (4H, m, H9, H16), 1.62 (4H, m, H12, H13).

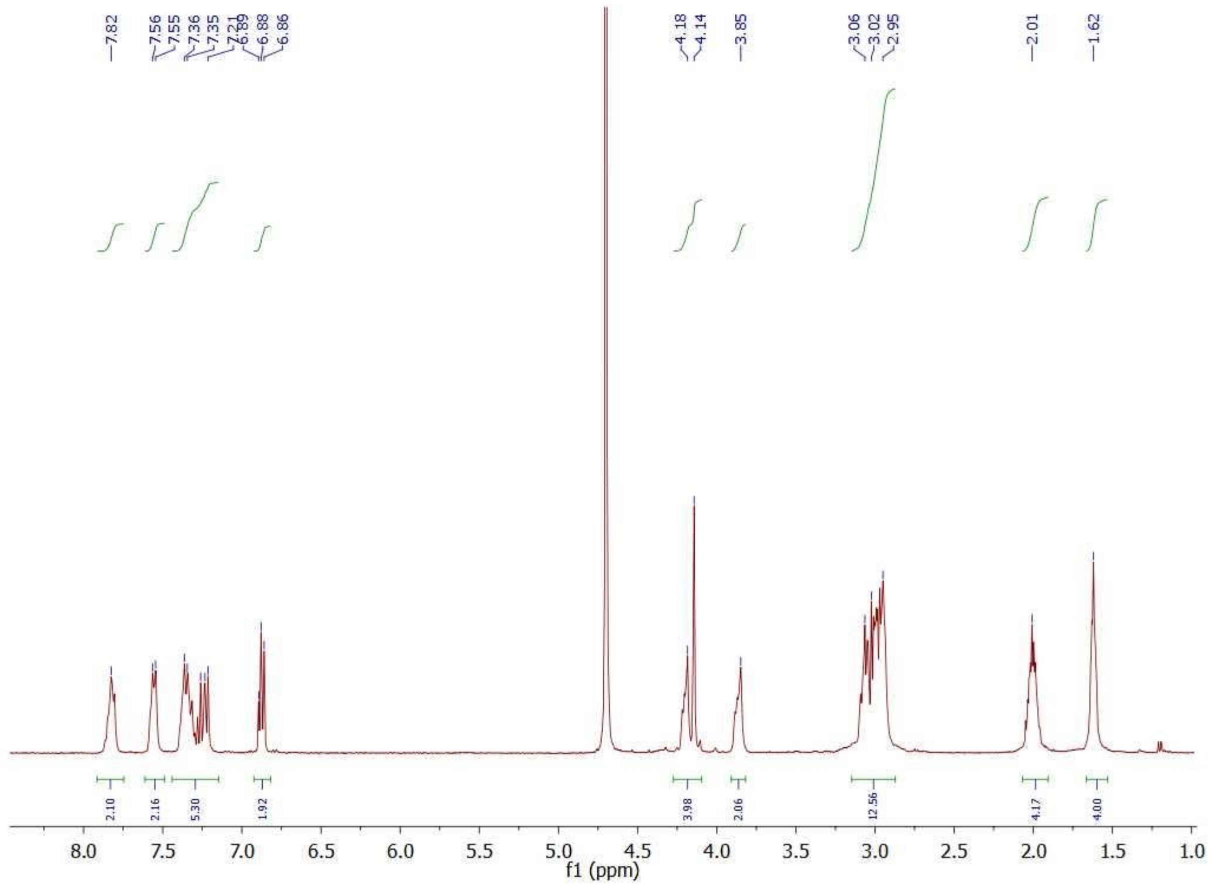

^{13}C NMR (H_2O/D_2O): δ 155.0 (C1), 144.4 (C23), 143.9 (C28, C29), 142.5 (C6), 140.2 (C19), 131.7 (C5), 131.6 (C3), 128.7 (C31), 128.6 (C24), 127.7 (C26), 127.1 (C26), 126.6 (C24), 120.5 (C4), 120.4 (C27), 120.3 (C30), 117.7 (C28), 115.5 (C21), 114.5 (C2), 51.4 (C23), 46.9 (C11, C14), 44.4 (C18), 43.6 (C7, C10, C15), 36.3 (C8, C17, C23), 22.6 (C12, C13), 22.4 (C9, C16).

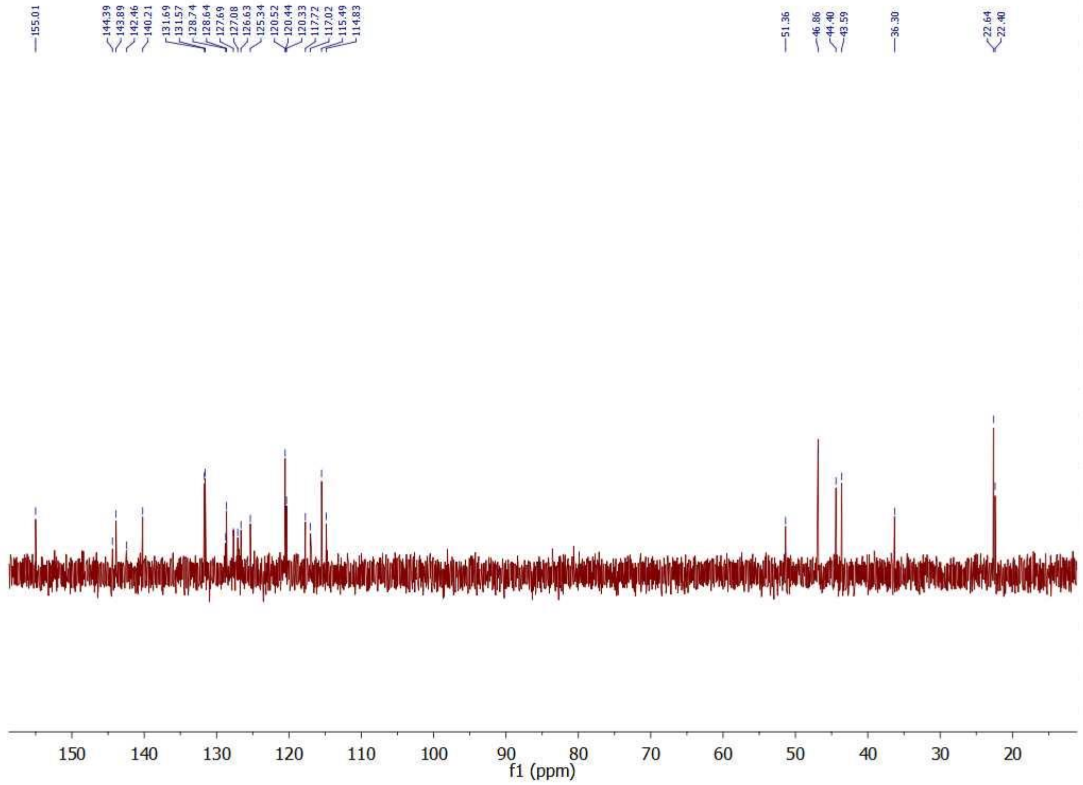

HMBC:

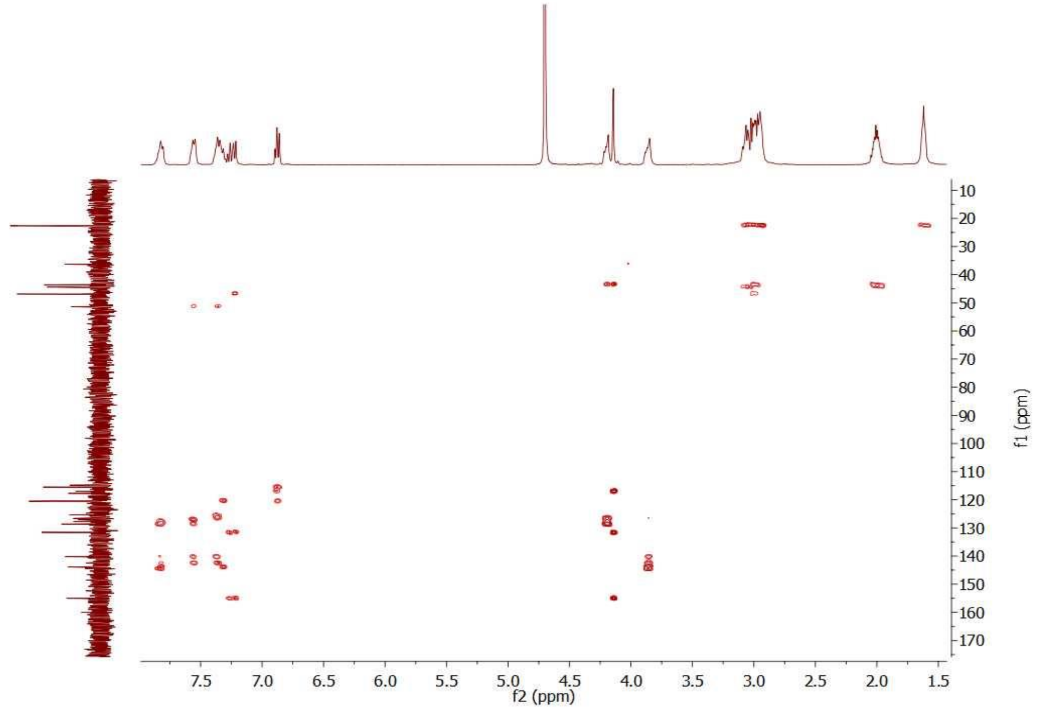

HSQC:

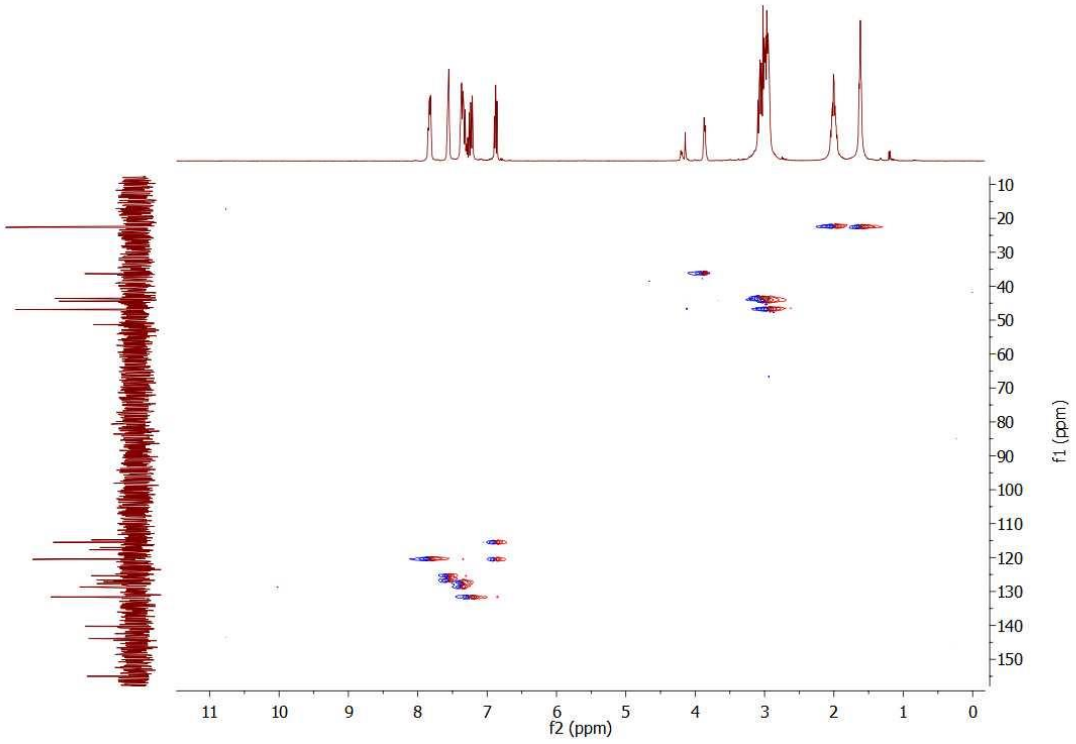

10 20 30 40 50 60 70 80 90 100 110 120 130 140 150
f1 (ppm)
11 10 9 8 7 6 5 4 3 2 1 0
f2 (ppm)

COSY:

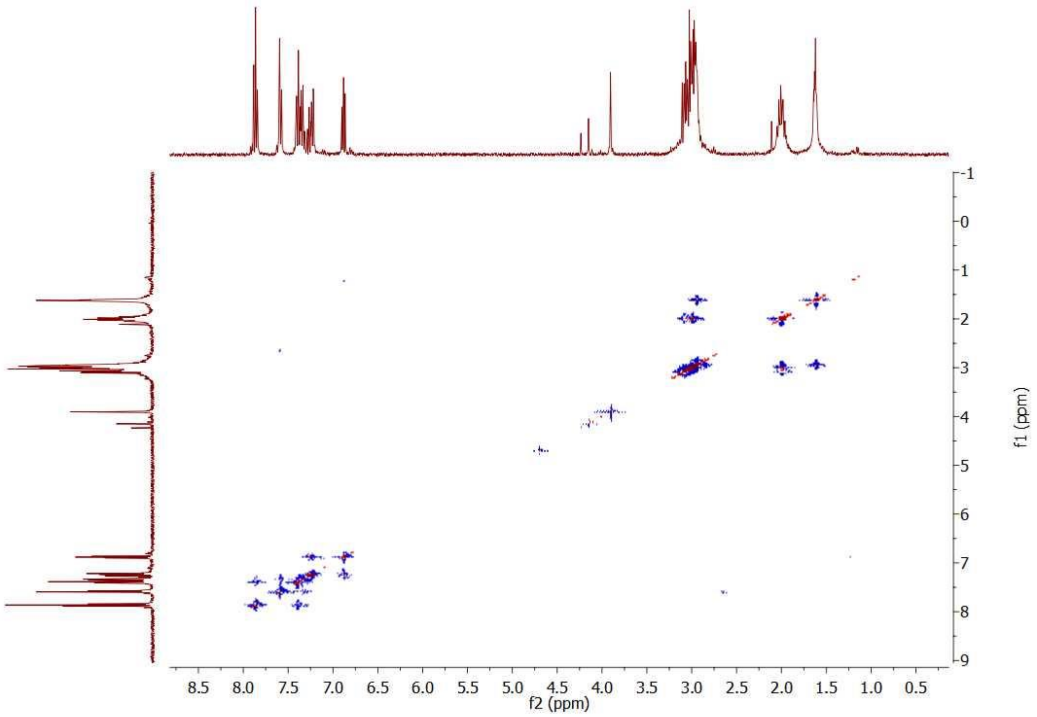

-1 0 1 2 3 4 5 6 7 8 9
f1 (ppm)
8.5 8.0 7.5 7.0 6.5 6.0 5.5 5.0 4.5 4.0 3.5 3.0 2.5 2.0 1.5 1.0 0.5
f2 (ppm)

HPLC: t_r = 8.7min, Purity at 220nm = >99%

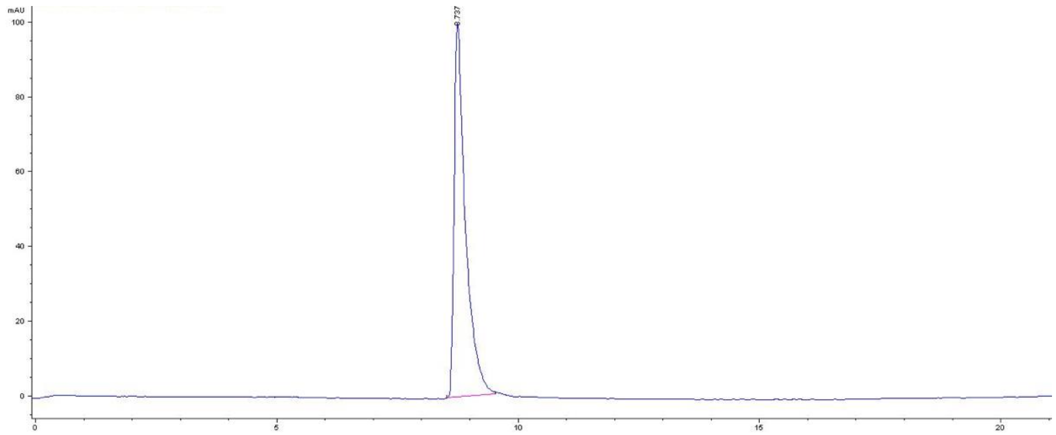

MS: Calculated MS= 486.3359; Found MS = 487.4005 $(M + H)^+$

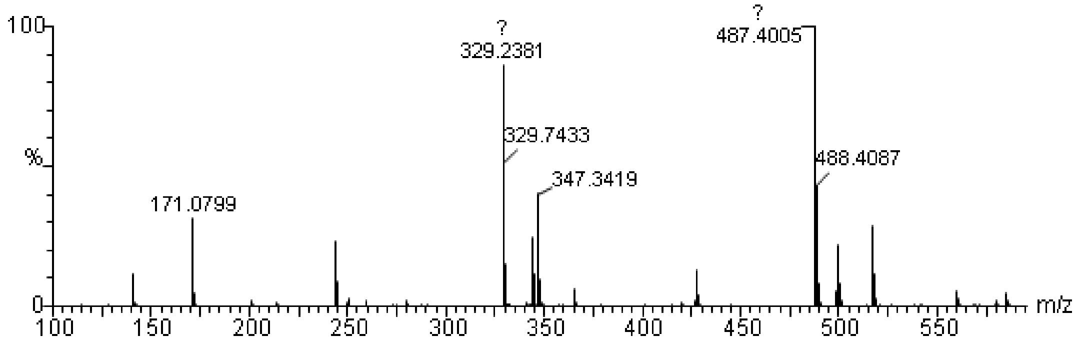

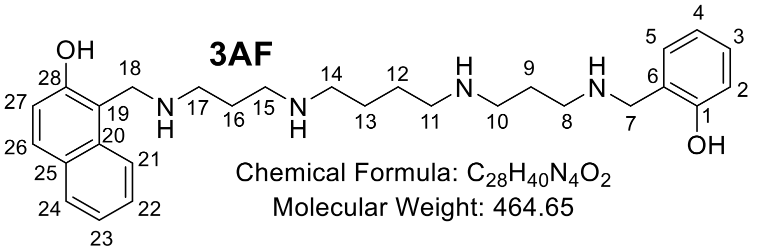

^{1}H RMN (D_2O): δ 7.90 (3H, m, H20, H23, H25), 7.57 (1H, dd, H22, J=8Hz), 7.4 (1H, dd, H21, J=8Hz), 7.29 (2H, m, H5, H3), 7.21 (1H, d, H26, J=9Hz), 6.91 (2H, m, H4, H2), 4.68 (2H, s, H17), 4.19 (2H, s, H7), 3.17 (2H, m, H28), 3.02 (10H, m, H8, H10, H11, H14, H15), 2.08 (4H, m, H9, H16), 1.66 (4H, m, H12, H13).

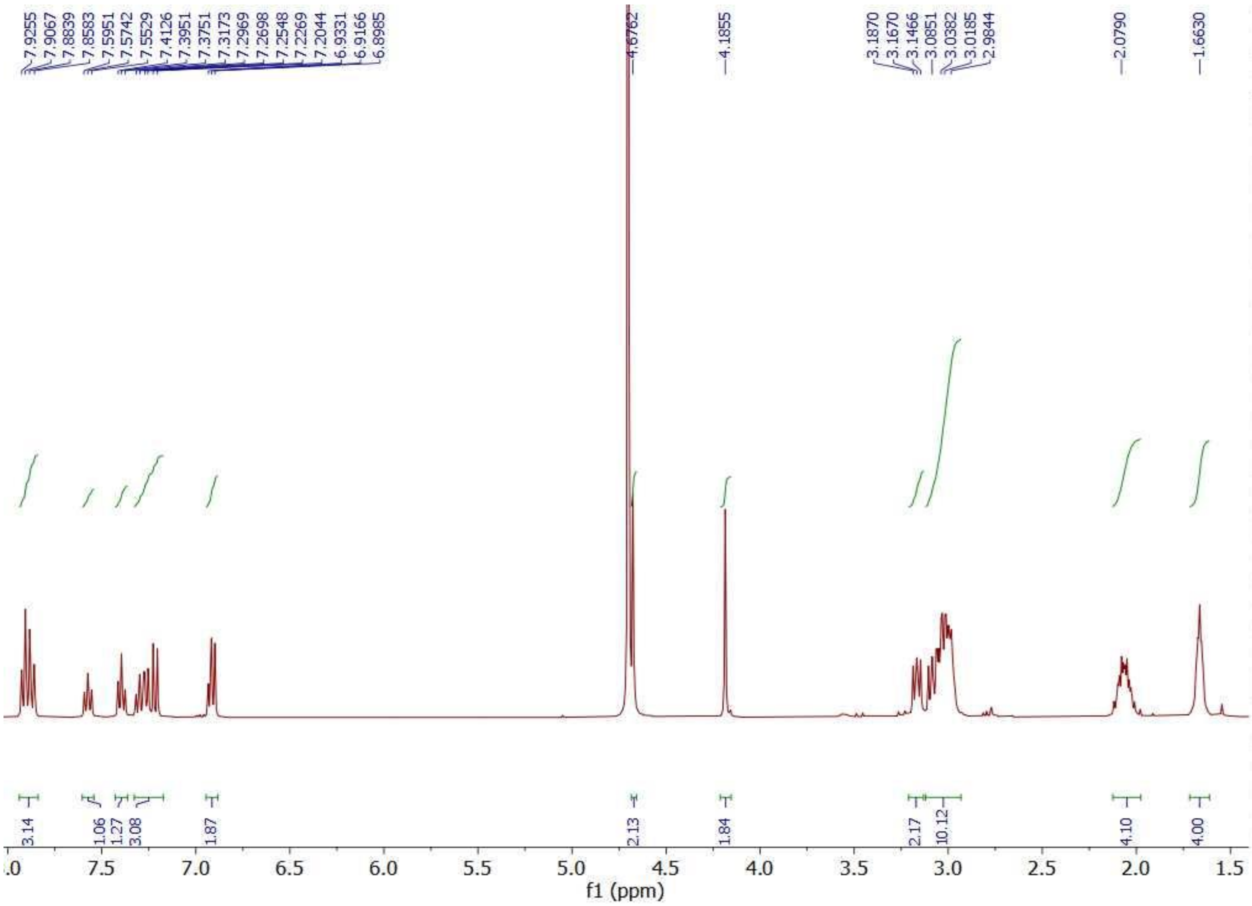

^{13}C RMN (H_2O/D_2O): δ 155.1 (C1), 154.2 (C27), 132.5 (C24), 132.2 (C23), 131.7 (C5), 131.6 (C6), 129.0 (C25), 128.5 (C19), 127.9 (C22), 123.8 (C21), 121.3 (C20), 120.6 (C3), 117.8 (C4), 117.1 (C26), 115.6 (C2), 114.9 (C4), 108.3 (C18), 46.9 (C10, C11), 44.5 (C8), 43.9 (C7), 43.7 (C28), 41.7 (C17), 22.7 (C12, C13), 22.5 (C9), 22.4 (C16).

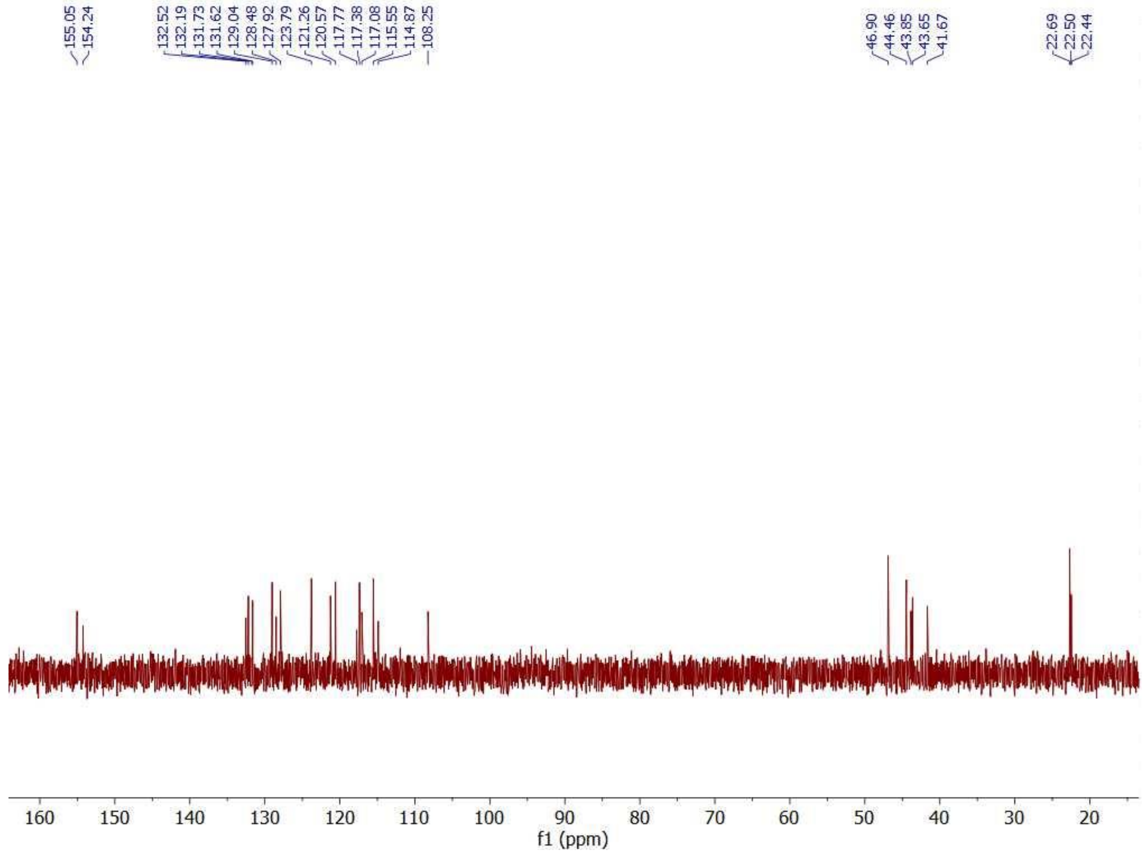
155.05
154.24
132.52
132.19
131.73
131.62
129.04
128.48
127.92
123.79
121.26
120.57
117.77
117.38
117.08
115.55
114.87
108.25
46.90
44.46
43.85
43.65
41.67
22.69
22.50
22.44
f1 (ppm)

HMBC:

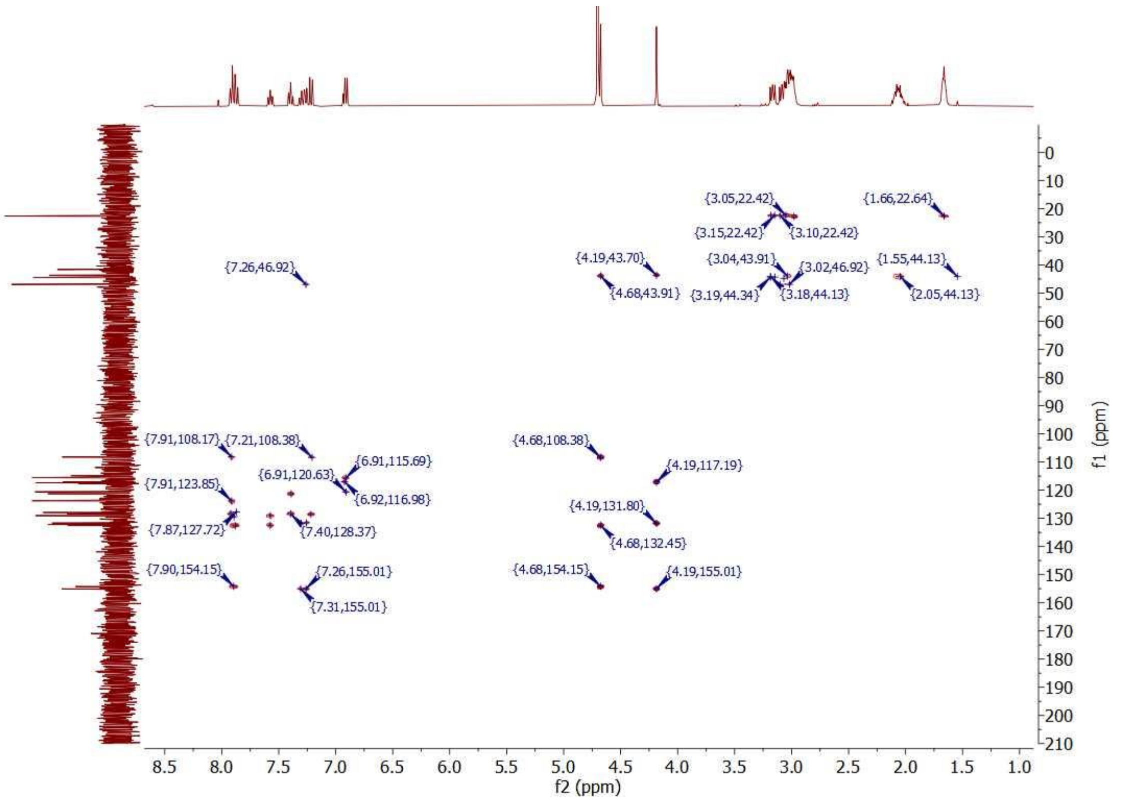
{3.05,22.42}
{1.66,22.64}
{3.15,22.42}
{3.10,22.42}
{4.19,43.70}
{3.04,43.91}
{3.02,46.92}
{1.55,44.13}
{7.26,46.92}
{4.68,43.91}
{3.19,44.34}
{3.18,44.13}
{2.05,44.13}
{7.91,108.17}
{7.21,108.38}
{4.68,108.38}
{6.91,115.69}
{6.91,120.63}
{4.19,117.19}
{7.91,123.85}
{6.92,116.98}
{4.19,131.80}
{7.87,127.72}
{7.40,128.37}
{4.68,132.45}
{7.90,154.15}
{7.26,155.01}
{4.68,154.15}
{4.19,155.01}
{7.31,155.01}
f2 (ppm)
f1 (ppm)

HSQC:

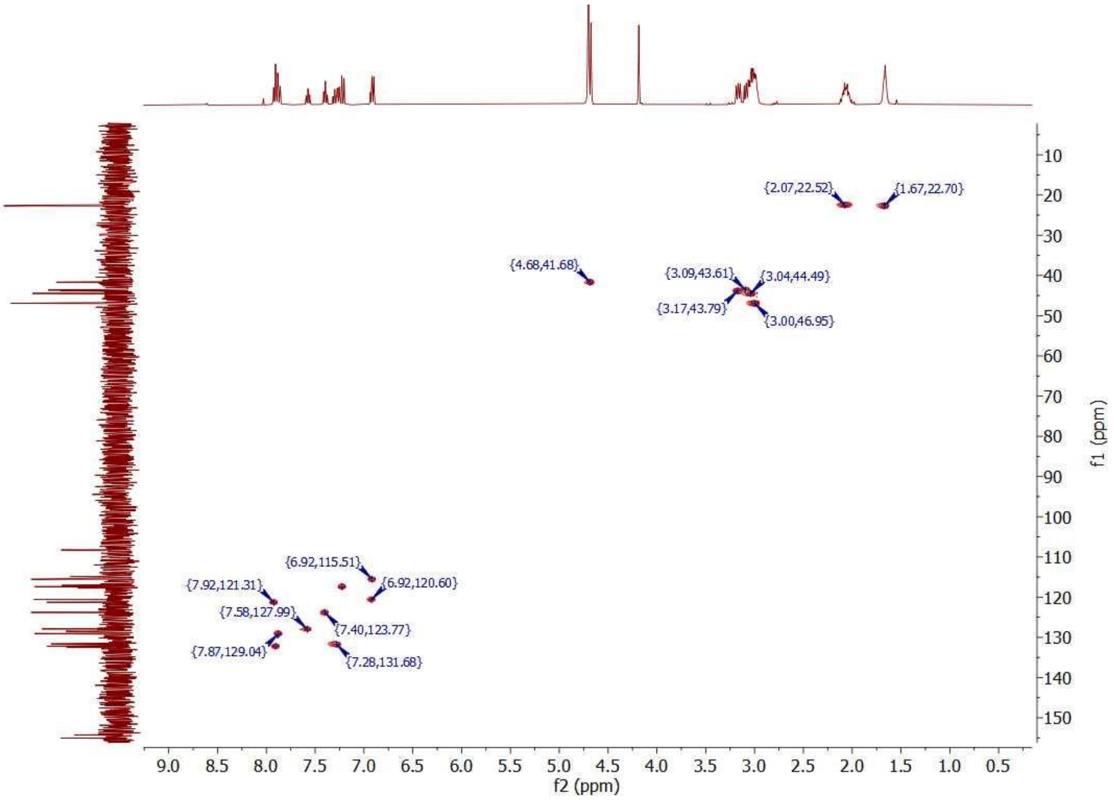

HPLC: t_r = 8.8min, Purity at 254nm = 95.9%

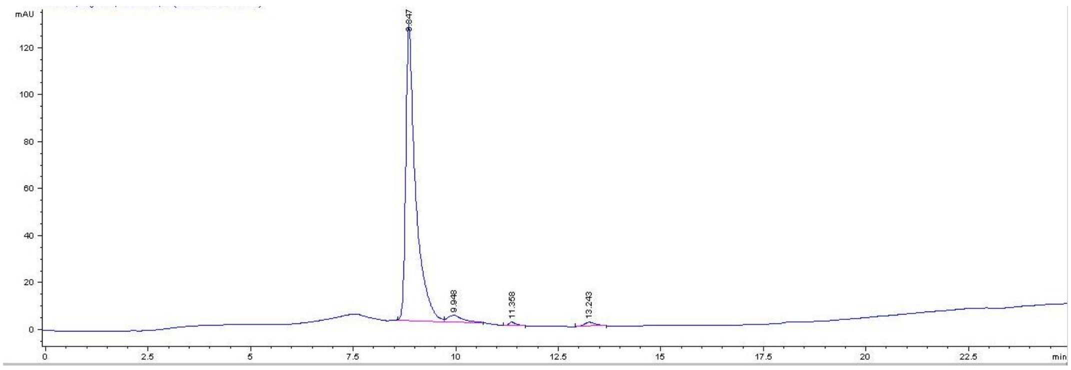

MS: Calculated MS= 464.3151; Found MS = 465.3169 $(M + H)^+$

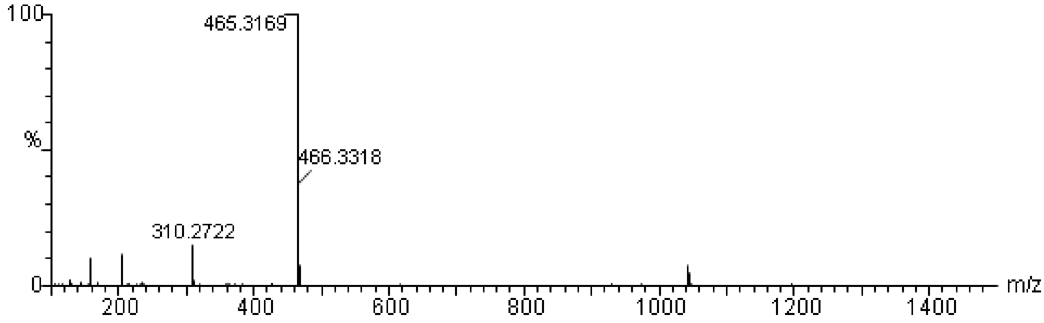

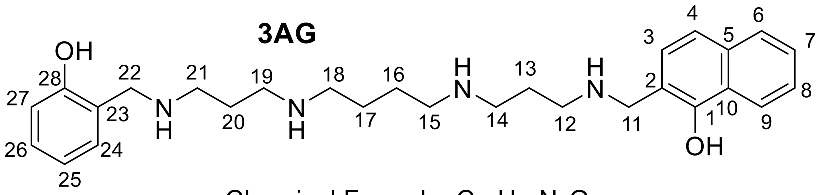

Chemical Formula: $C_{28}H_{40}N_4O_2$
Exact Mass: 464.32

^{1}H RMN (D_2O): δ 8.06 (1H, dd, J1= 3.4Hz, J2=6.3Hz, H9); 7.82 (1H, dd, J1= 3.4Hz, J2=6.3Hz, H6); 7.49 (3H, m, H4, H7, H8), 7.31 (1H, d, J3=8.4Hz, H3), 7.22 (2H, m, H26, H24), 6.85 (2H, m, H25, H27), 4.32 (2H, s, H11), 4.11 (2H, s, H22), 3.00 (12H, m, H21, H18, H18, H15, H14, H12), 1.99 (4H, m, H20, H13), 1.59 (4H, m, H16, H17)

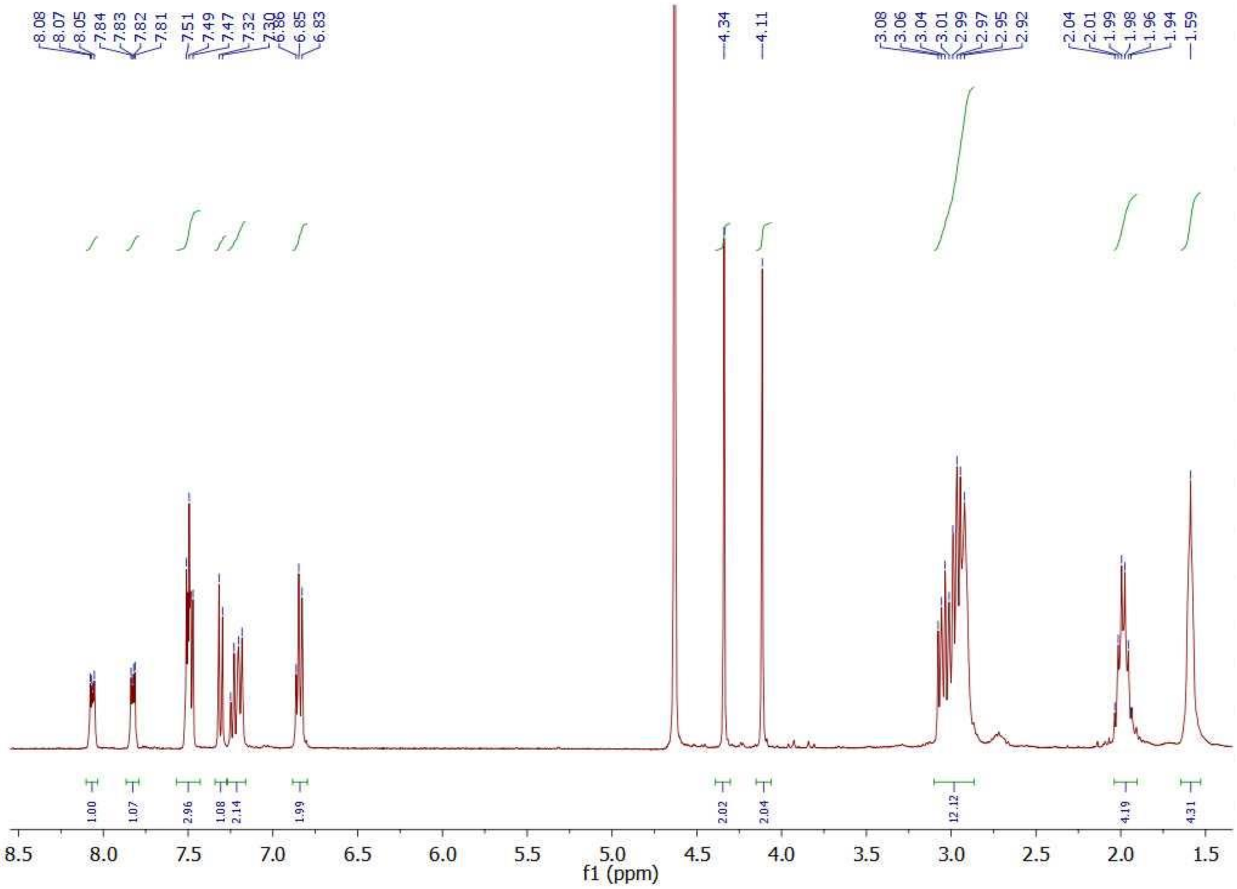

^{13}C RMN (D_2O): 155.0 (C28), 150.9 (C1), 135.0 (C5), 131.7 (C24), 131.5 (C26), 128.1 (C6), 127.8 (C3), 127.4 (C8), 126.3 (C7), 125.1 (C10), 121.4 (C4), 121.2 (C9), 120.5 (C25), 117.0 (C23), 115.5(C27), 113.1(C2), 46.9 (C22, C11), 44.4 (C18, C15), 43.6 (C21, C19, C14, C12), 22.6 (C16, C17), 22.4 (C20, C13).

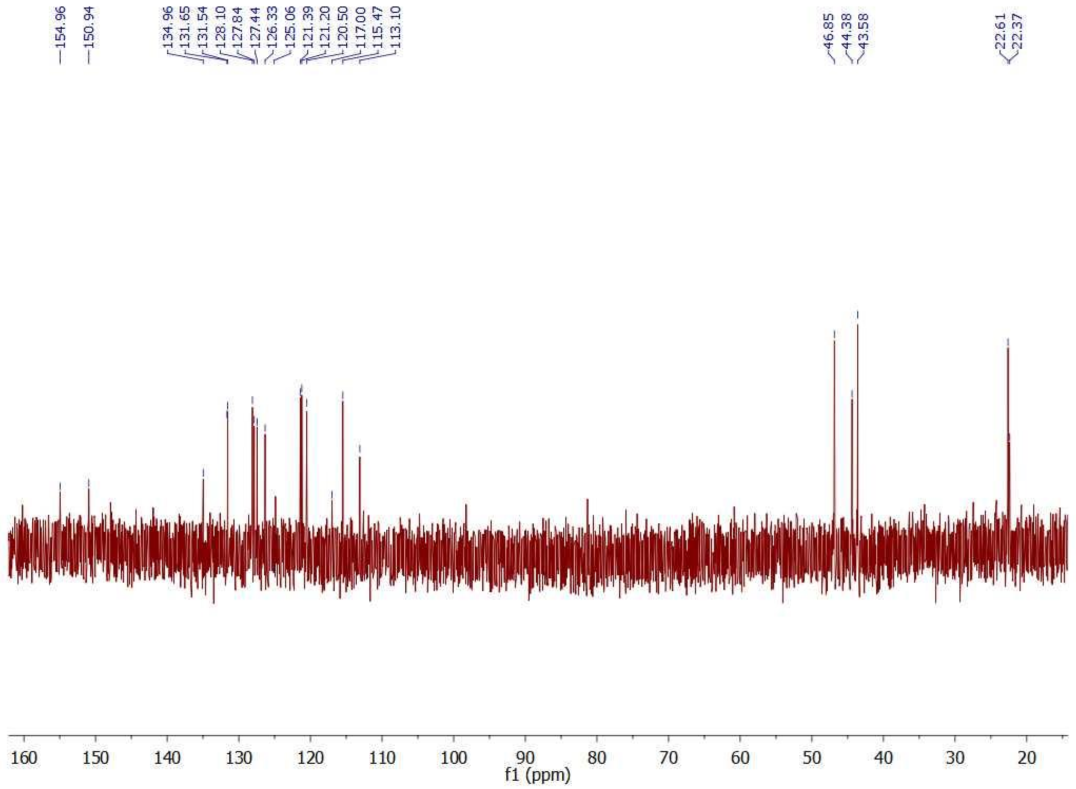

HMBC:

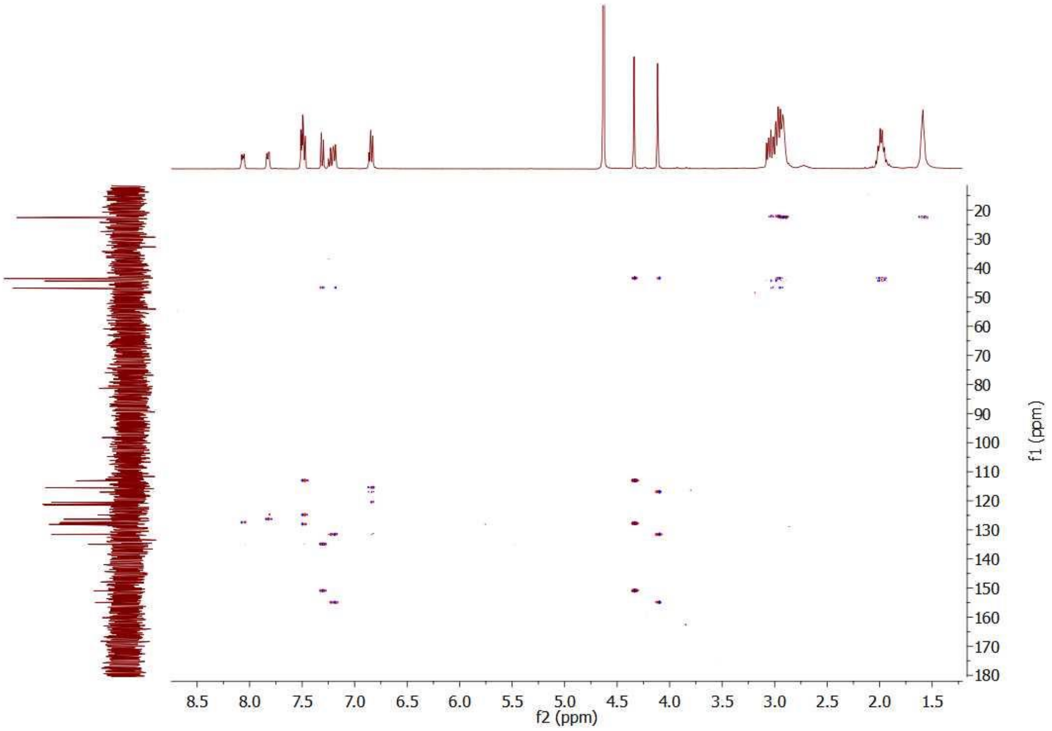

HSQC:

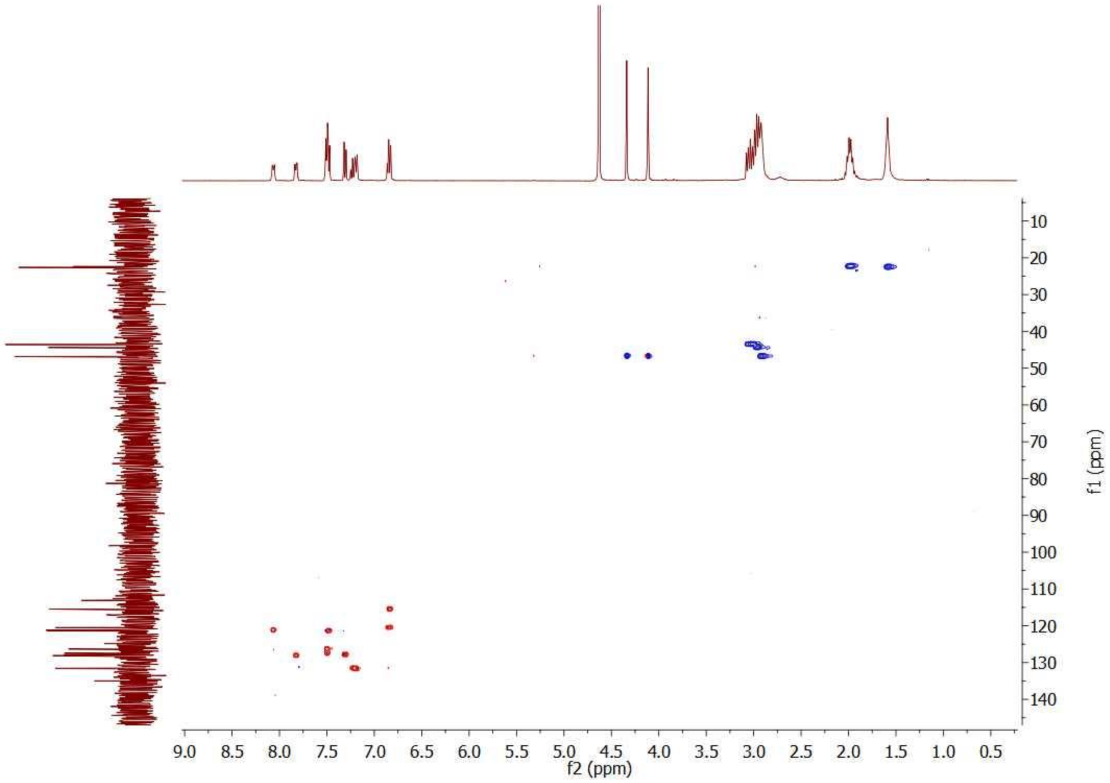

HPLC: t_r = 9.2min, Purity at 220nm = 97.4%

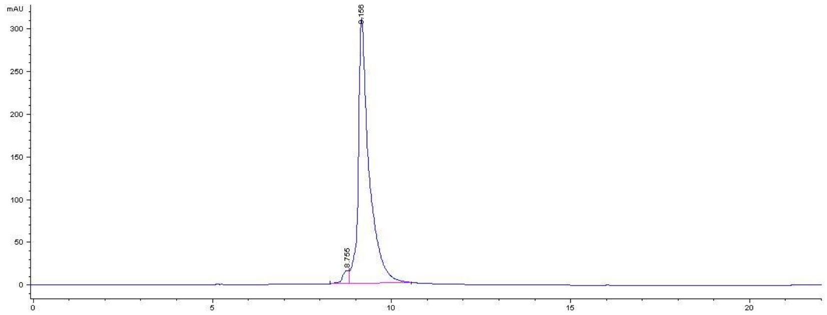

MS: Calculated MS= 464.3151; Found MS = 465.3228 $(M + H)^+$

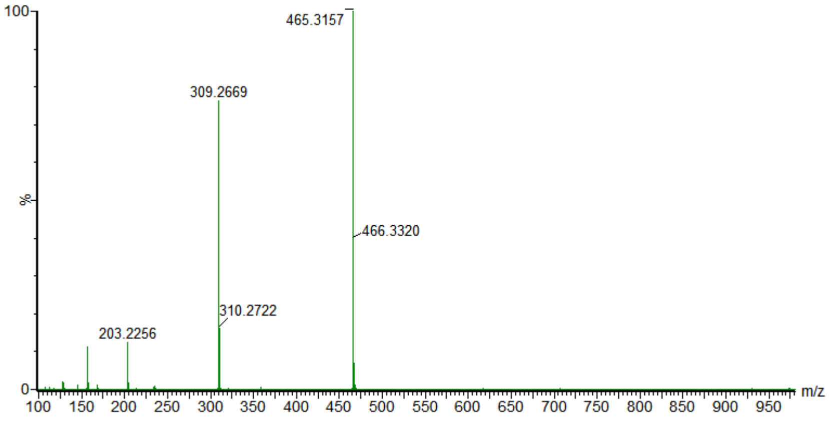

3AN

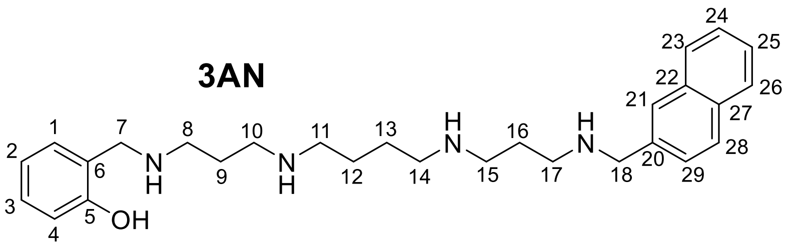

Chemical Formula: $C_{28}H_{40}N_4O$
Exact Mass: 448.32
Molecular Weight: 448.66

^{1}H RMN (D_2O): δ 7.87 (m, 4H, m, H21, H23, H26, H28), 7.50 (m, 2H, H24, H25), 7.43 (d, J=8.44Hz, 1H, H29), 7.22 (m, 2H, H1, H3), 6.85 (tr, J=6.97Hz, 2H, H2, H4), 4.3 (s, 2H, H18), 4.12 (s, 2H, H7), 3.00 (m, 12H, H8, H10, H11, H14, H15, H17), 1.98 (m, 4H, H9, H16), 1.59 (m, 4H, H12, H13).

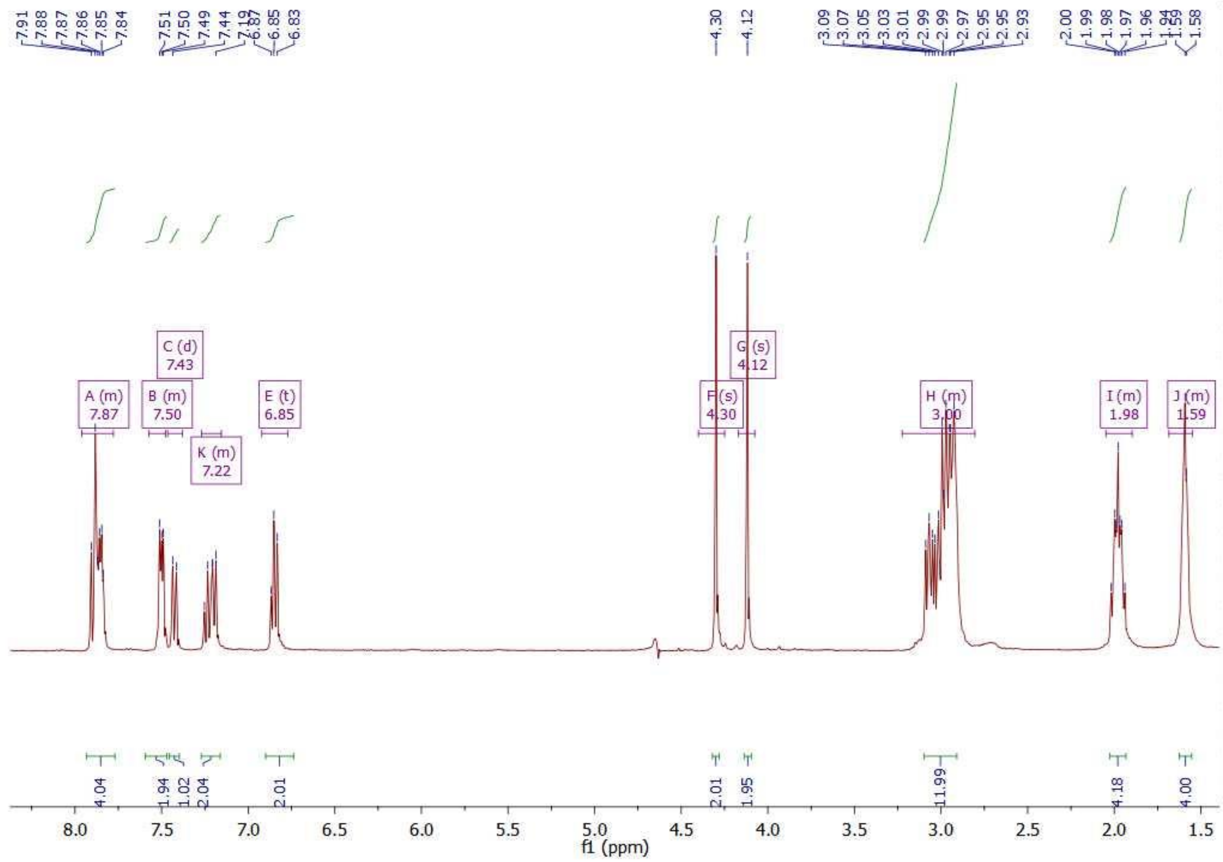

^{13}C RMN (D_2O): δ 155.0 (C5), 133.1 (C20), 132.7(C22), 131.7(C27), 131.5(C1), 129.6(C3), 129.1(C29), 128.0(C23), 127.7(C28), 127.4(C24), 127.0(C25), 126.4(C21), 120.5(C2), 117.0(C18), 115.5(C4), 51.3(C18), 46.9(C11, C14), 46.8(C7), 44.4(C10), 44.3(C15), 43.7(C17), 43.6(C8), 22.6(C13), 22.5(C12), 22.4(C9, C16).

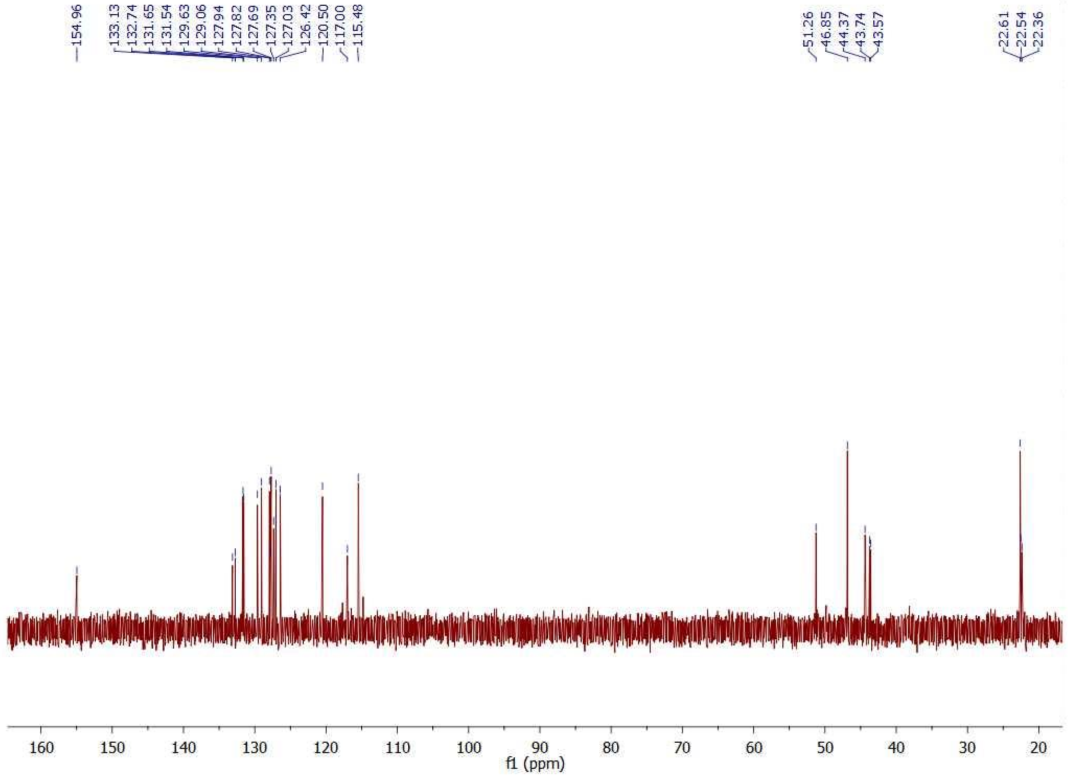

HMBC:

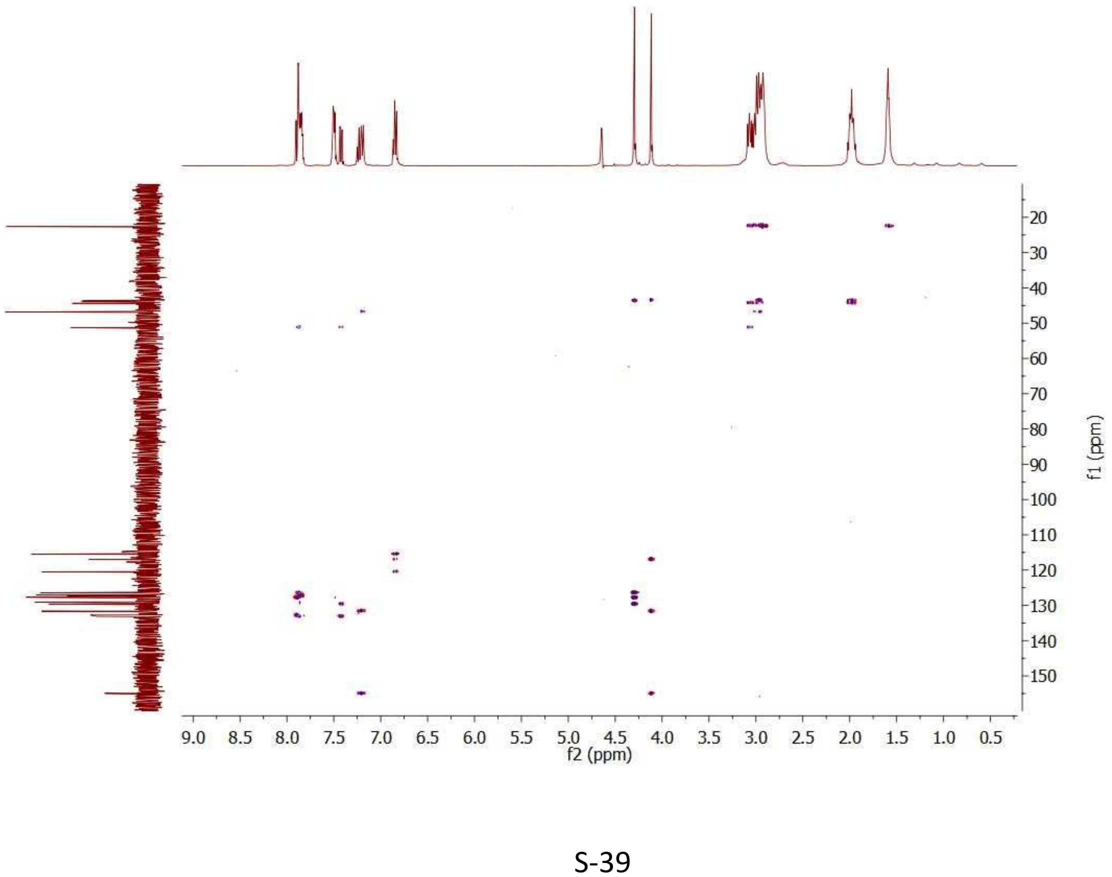

HSQC:

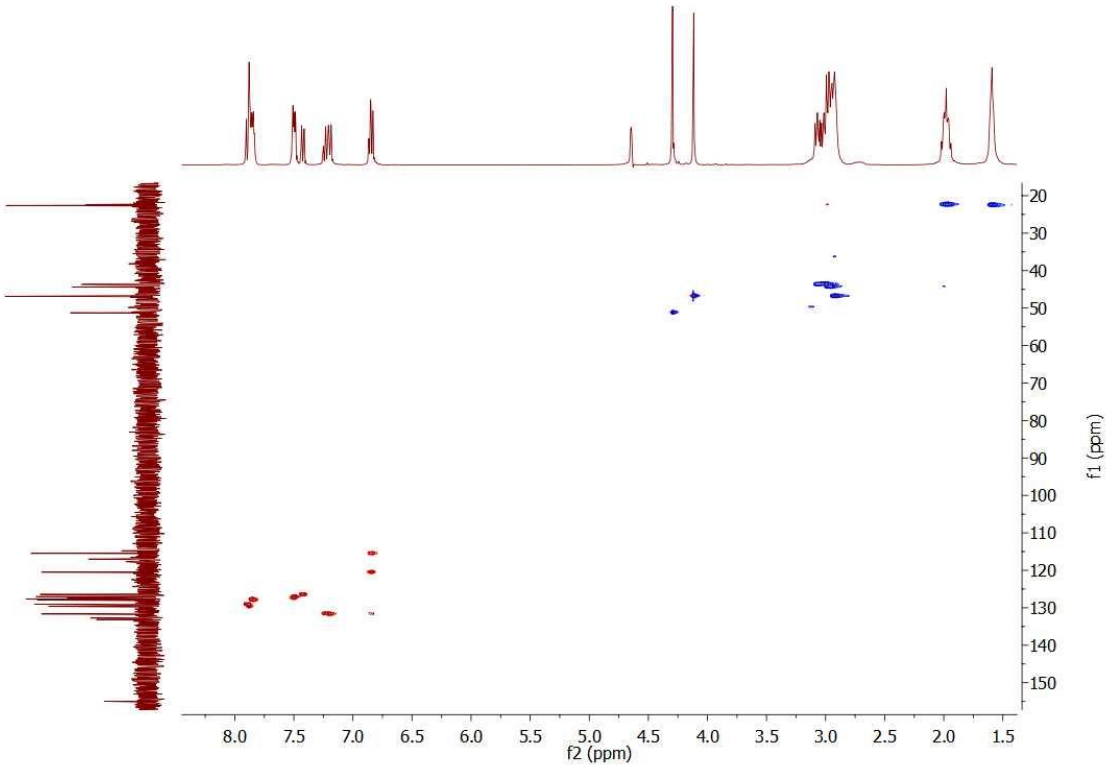

HPLC: t_r = 8.6min, Purity at 220nm = >99%

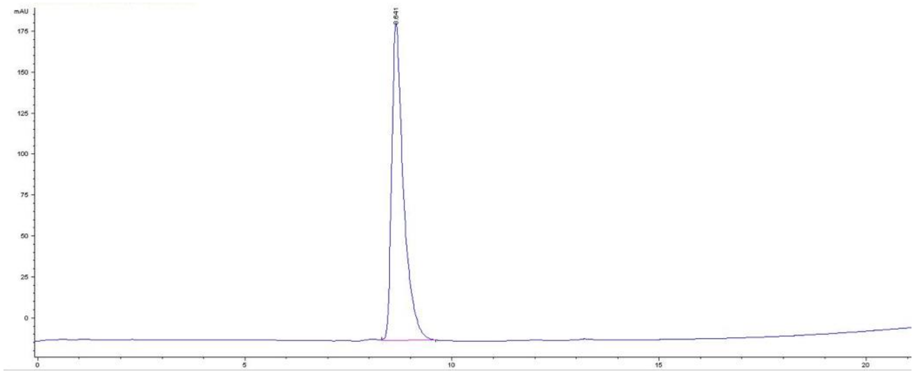

MS: Calculated MS= 448.3202; Found MS = 449.3520 $(M + H)^+$

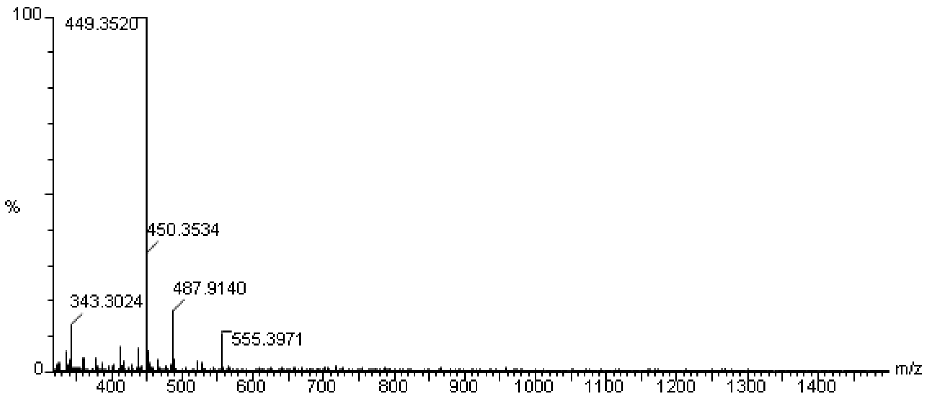

3J

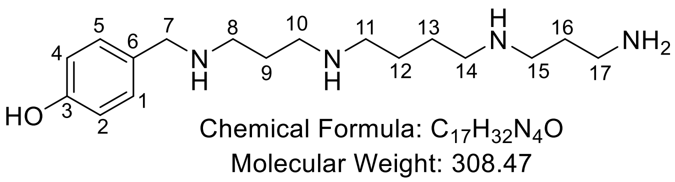

^{1}H RMN (D_2O): δ 7.18 (2H, d, J=8Hz, H1, H5), 6.78 (2H, d, J=8Hz, H2, H4), 3.95 (2H, s, H7), 2.92 (12H, m, H8, H10, H11, H14, H15, H17), 1.89 (4H, m, H9, H16), 1.58 (4H, m, H12, H13).

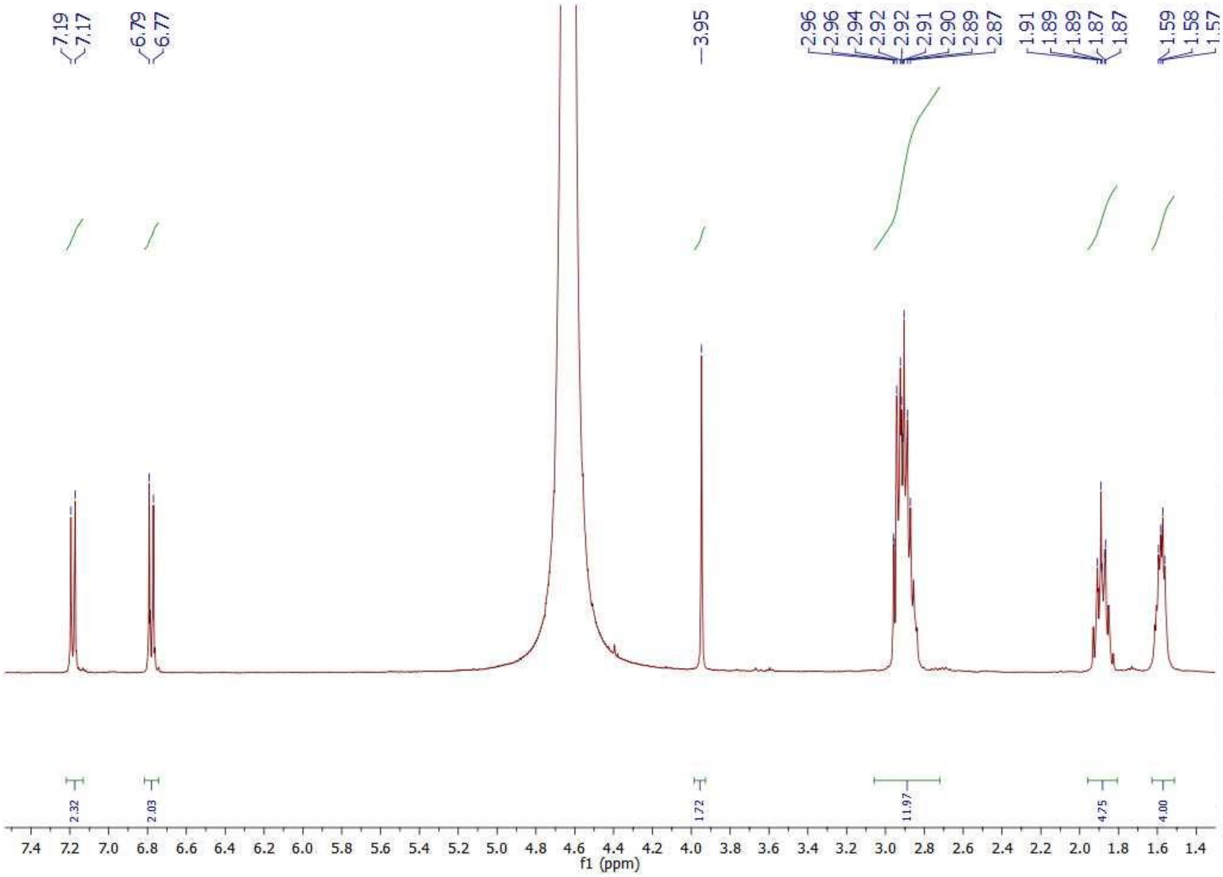

^{13}C RMN (H_2O/D_2O): 156.7 (C3), 131.4 (C1, C5), 123.3 (C6), 115.9 (C2, C4), 50.8 (C7), 47.1(C11), 47.0 (C14), 44.8 (C10), 44.6 (C15), 43.9 (C17), 36.7 (C16), 24.2 (C9), 23.3 (C12), 23.2 (C13).

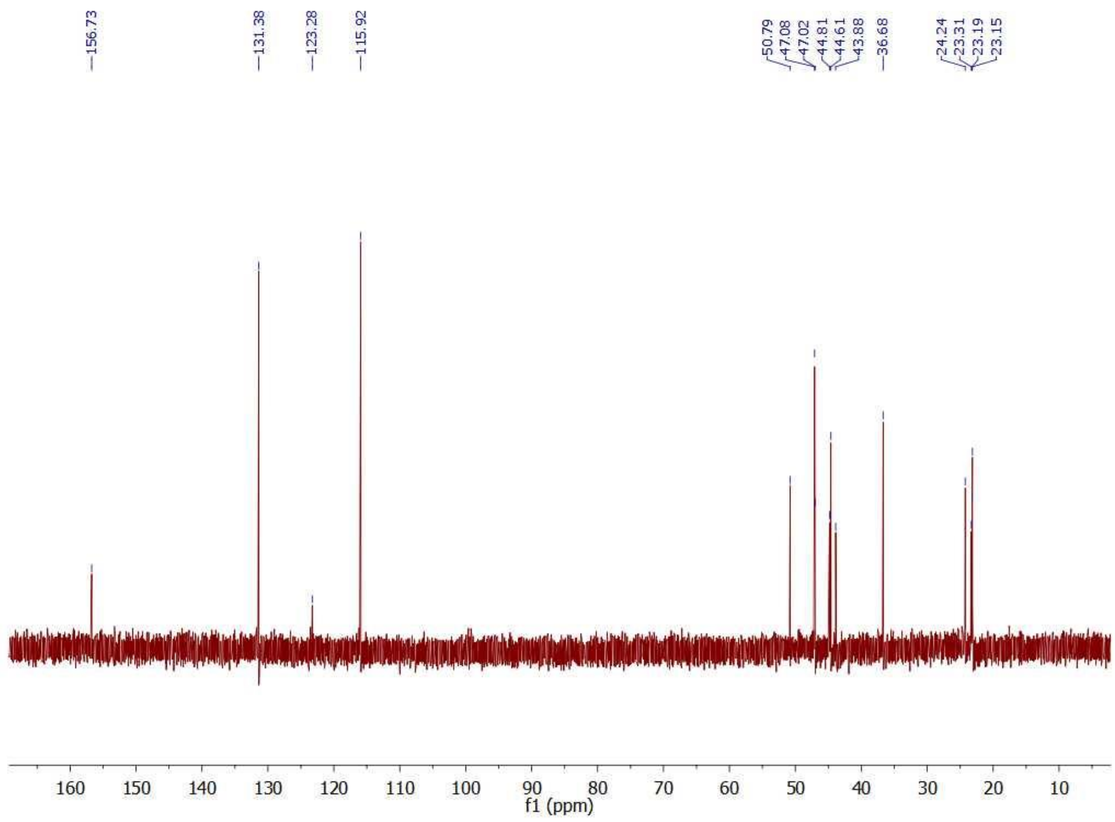
156.73
131.38
123.28
115.92
50.79
47.08
47.02
44.81
44.61
43.88
36.68
24.24
23.31
23.19
23.15
f1 (ppm)

HMBC:

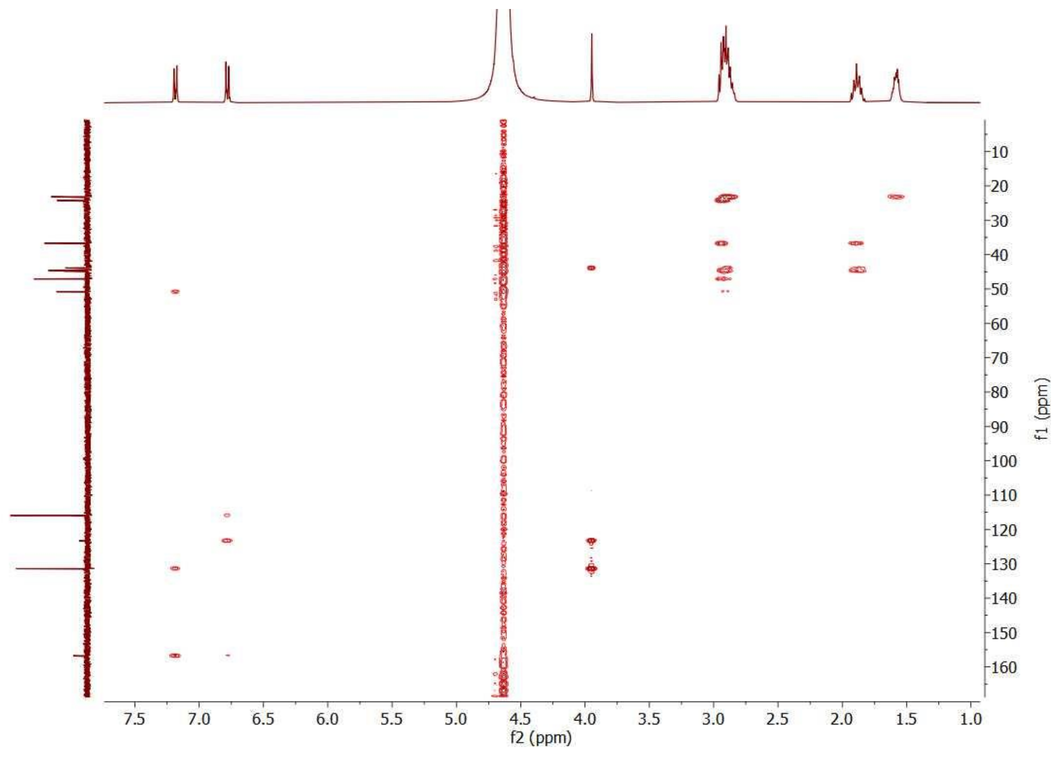
f1 (ppm)
f2 (ppm)

HSQC:

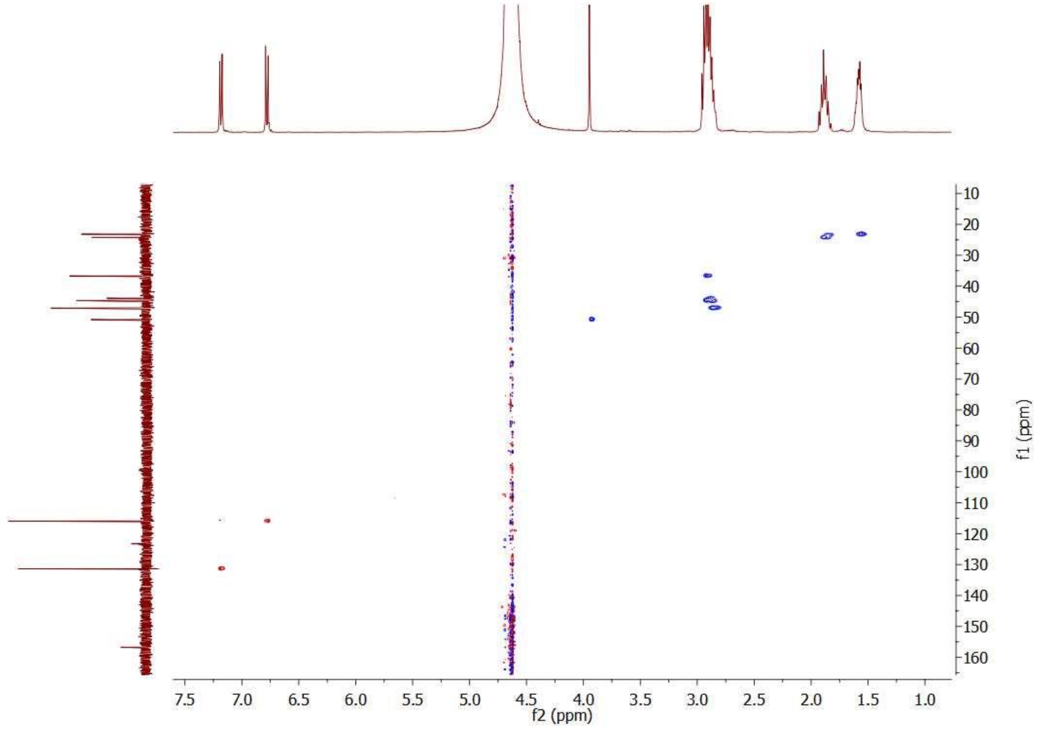

HPLC: t_r = 7.6min, Purity at 220nm = 98.1%

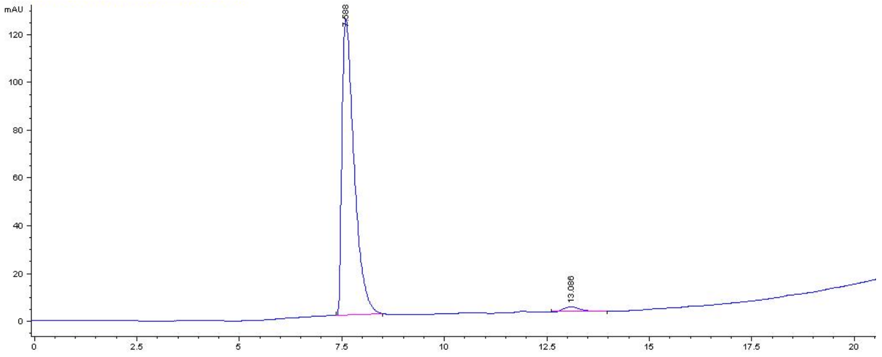

MS: Calculated MS= 308.2576; Found MS = 309.2698 $(M + H)^+$

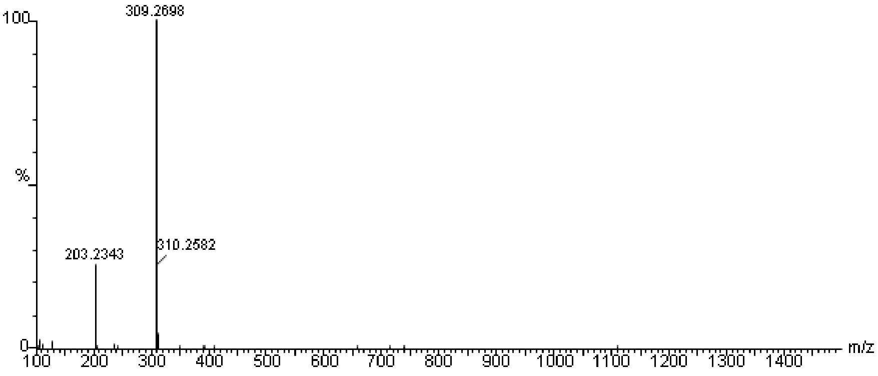

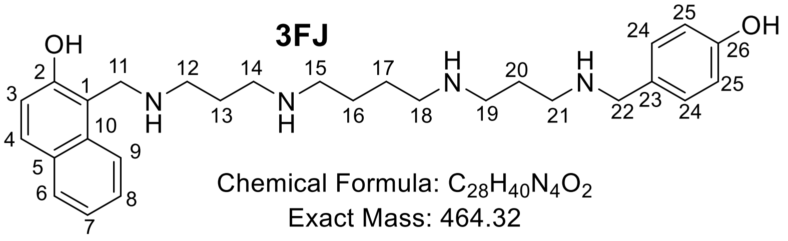

¹H RMN (D_2O): δ 7.81 (3H, m, H4, H6, H9), 7.5 (1H, dd, J1=7.5Hz, J2=1Hz, H7), 7.32 (1H, dd, J1=7.5Hz, J2=1Hz, H8), 7.19 (2H, d, J3=8.5Hz, H24), 7.14 (1H, d, J5=8Hz, H3), 6.79 (2H, d, J3=8.5Hz, H25), 4.6 (inside water, s, H11), 4.02 (3H, s, H22), 3.09 (2H, m, H21), 2.95 (10H, m, H12, H14, H15, H17, H19), 1.95 (4H, m, H13, H20), 1.58 (4H, m, H16, H17).

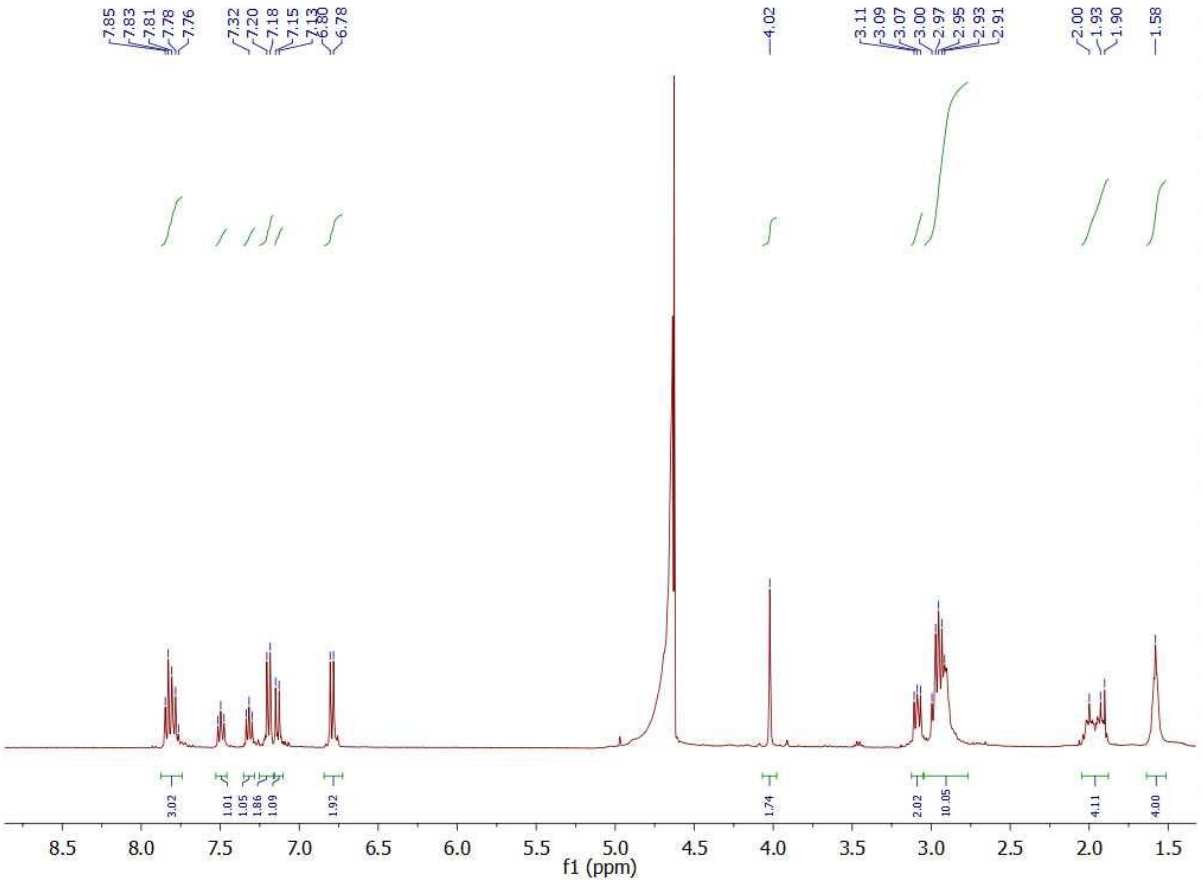

^{13}C RMN (D_2O): δ 156.7(C26), 154.2(C2), 132.4(C5), 132.1(C6), 132.0 (C23), 131.6 (C24), 129.0 (C4), 128.4 (C10), 127.8(C7), 123.7(C8), 121.2 (C9), 117.3(C3), 115.9(C25), 108.2 (C1), 50.6 (C22), 46.9 (C15, C18), 44.4 (C19), 43.7 (C21), 43.4 (C12), 41.7 (C11), 40.0 (C14), 22.6 (C16, C17), 22.5 (C13, C20).

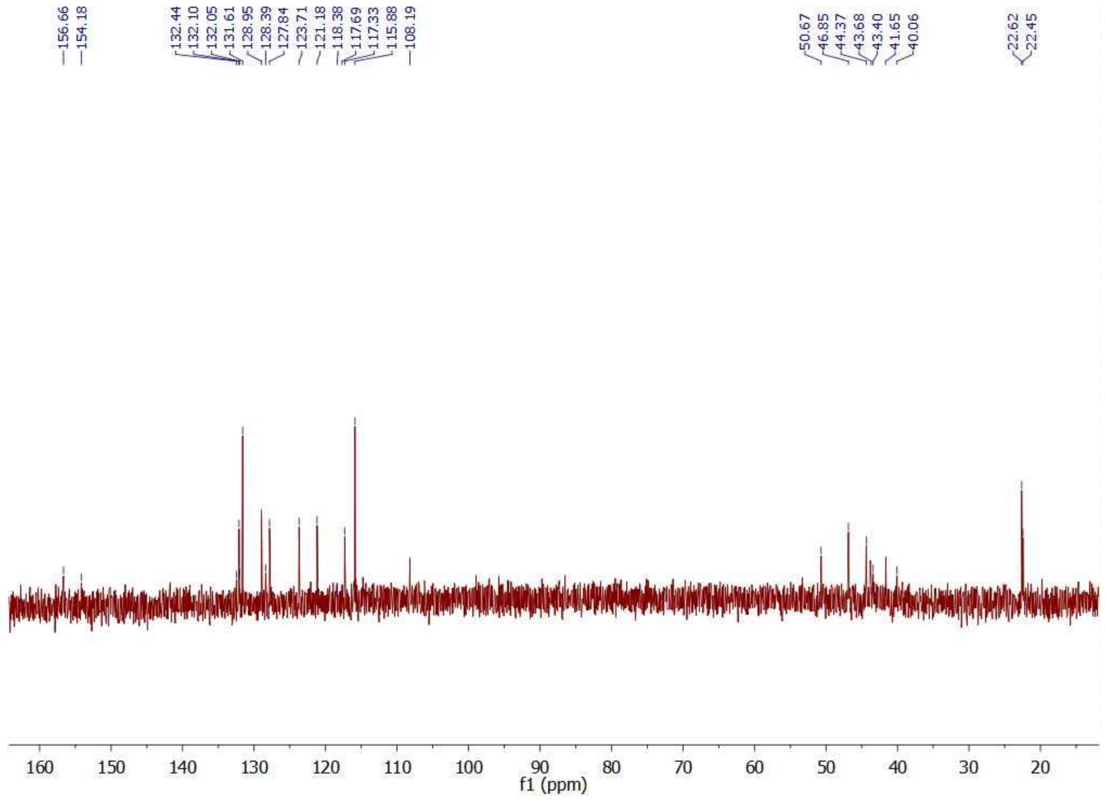

HMBC:

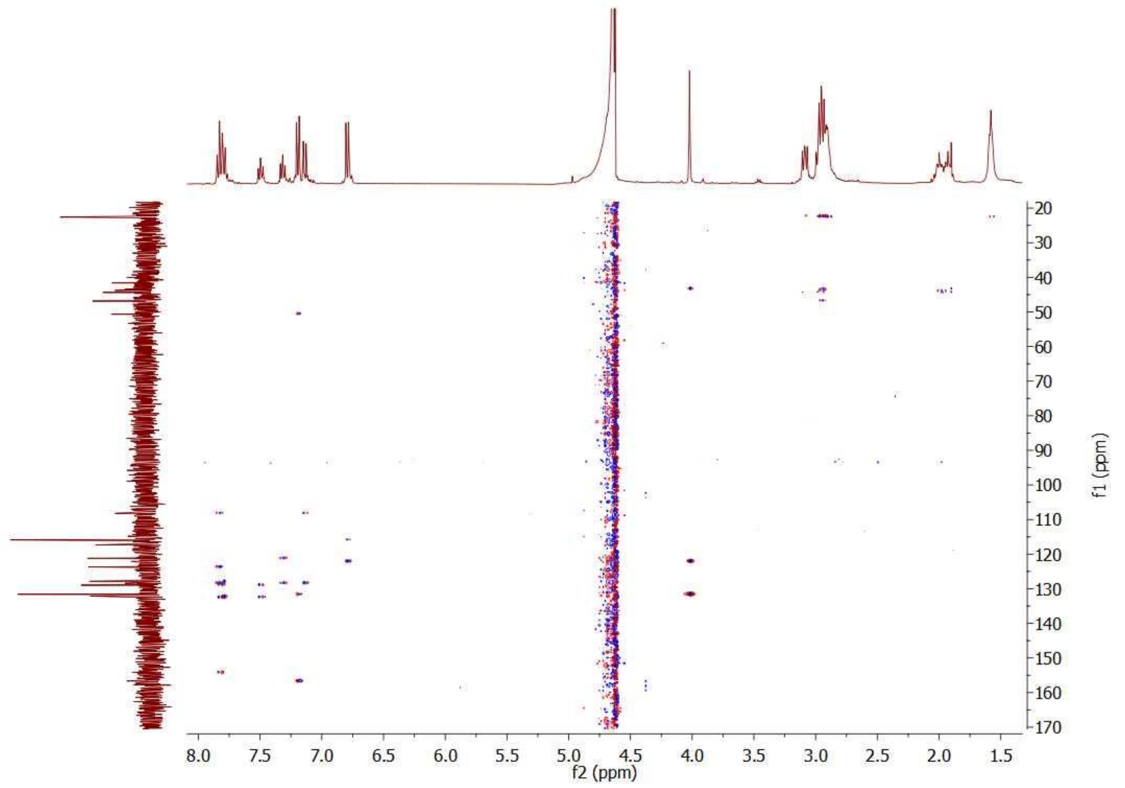

HSQC:

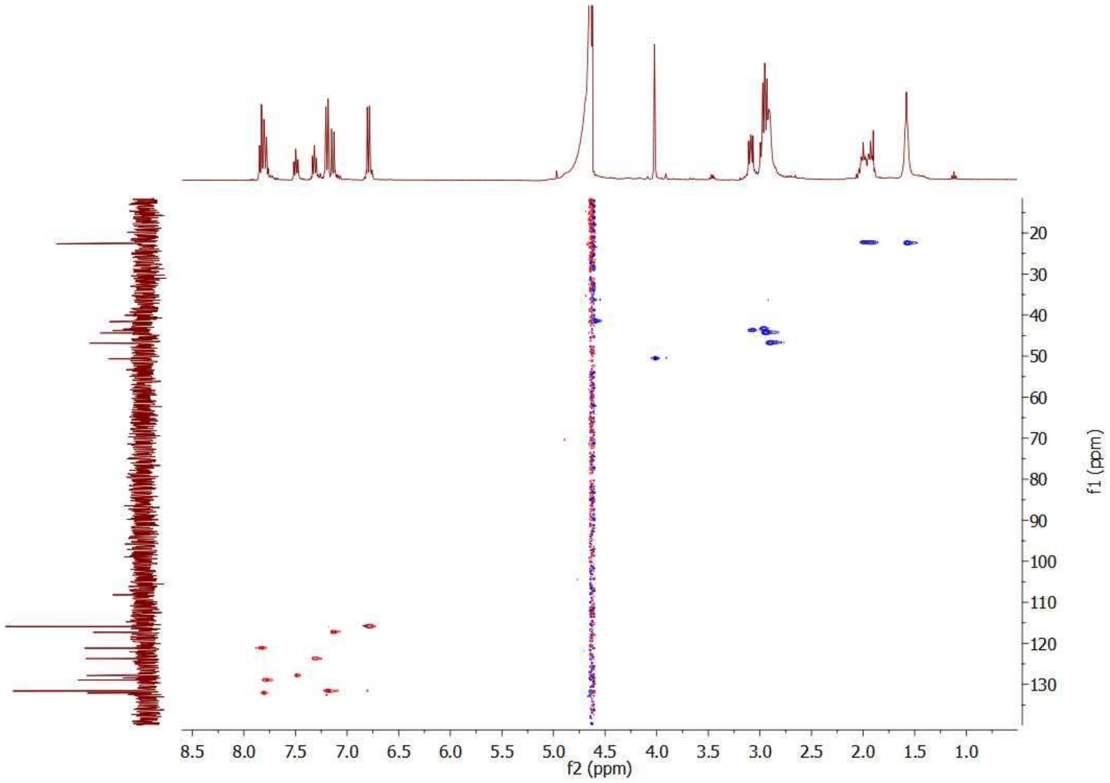

HPLC: t_r = 9.05min, Purity at 220nm = 98.5%

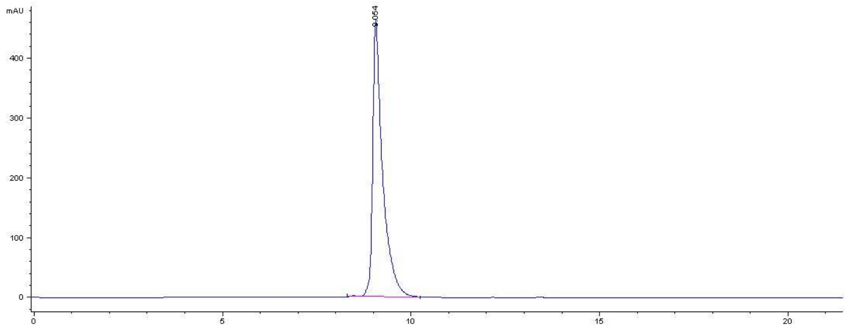

MS: Calculated MS= 464.3151; Found MS = 465.3317 $(M + H)^+$

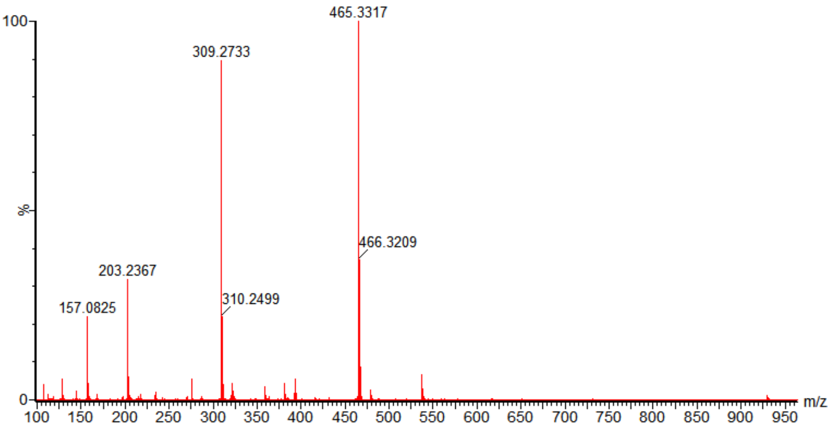

3O

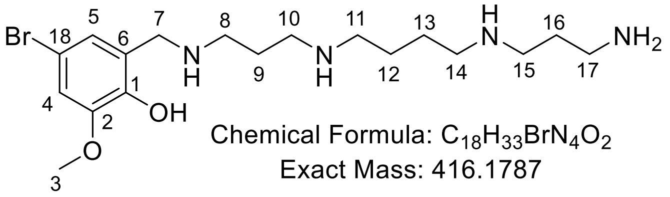

Chemical Formula: $C_{18}H_{33}BrN_4O_2$
Exact Mass: 416.1787

^{1}H RMN (D_2O): δ 7.18 (1H, s, H5), 7.01 (1H, s, H4), 4.11 (2H, s, H7), 3.77 (3H, s, H3), 2.98 (12H, m, H8, H10, H11, H14, H15, H17), 1.96 m (4H, H9, m, H16), 1.65 (4H, m, H12, H13)

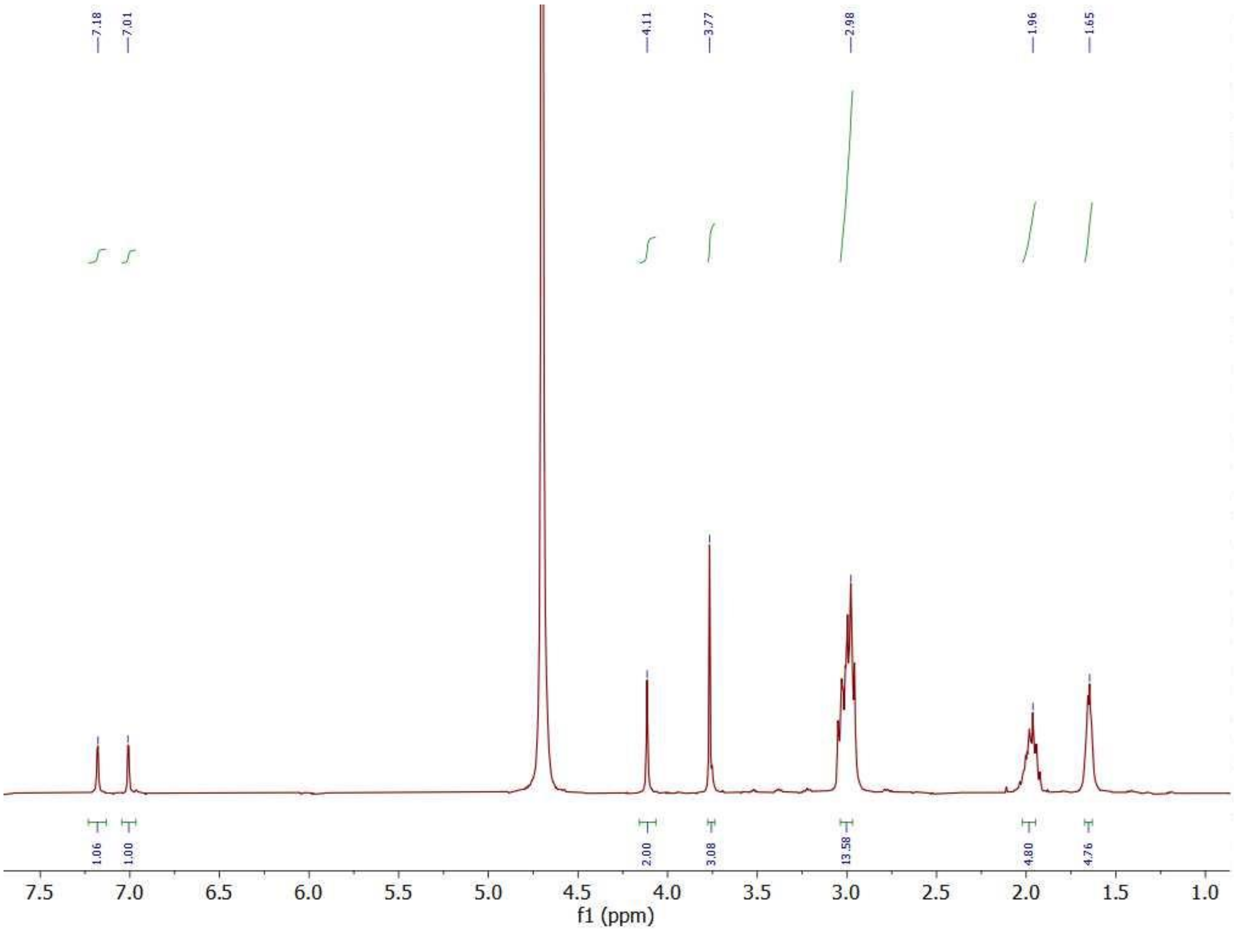

^{13}C RMN (D_2O): δ 148.4 (C1), 144.0 (C2), 125.2 (C4), 118.5 (C18), 116.6 (C5), 111.1 (C6), 56.3 (C3), 46.9 (C7), 45.8 (C11, C14), 44.4 (C8), 44.3 (C15), 36.4 (C16), 23.6 (C9), 22.7 (C13), 22.4 (C12).

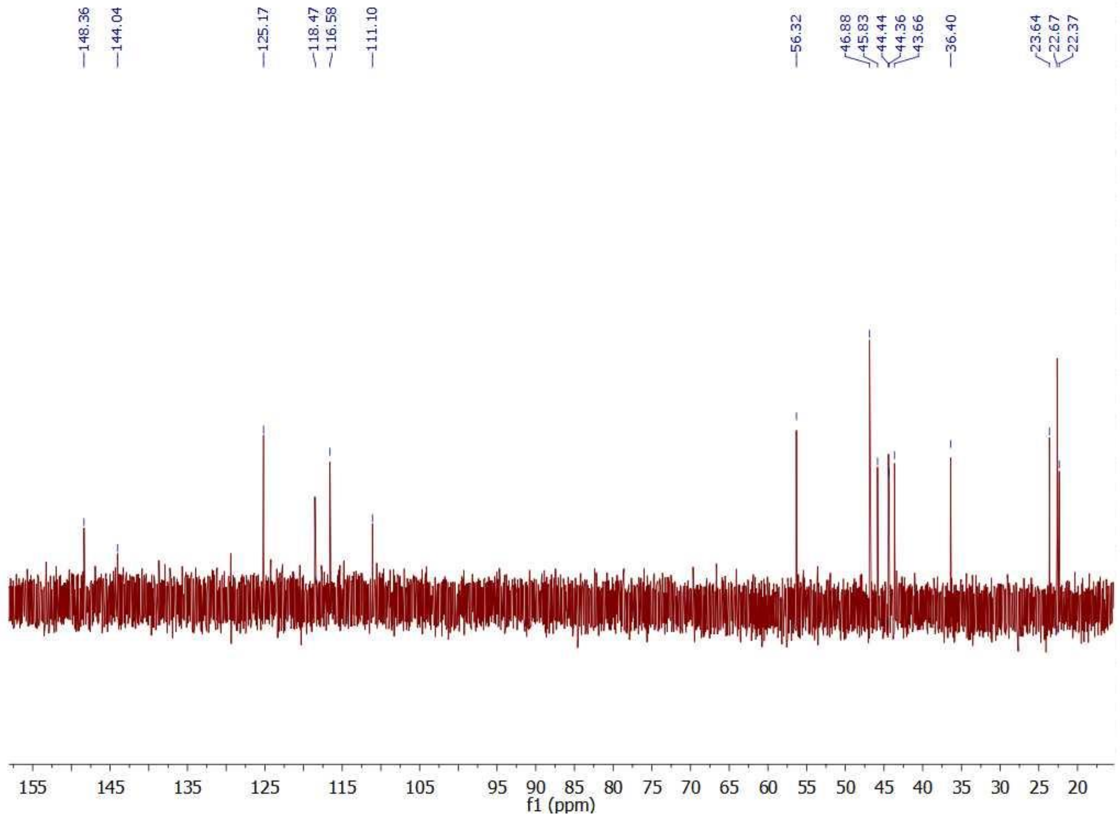

HMBC:

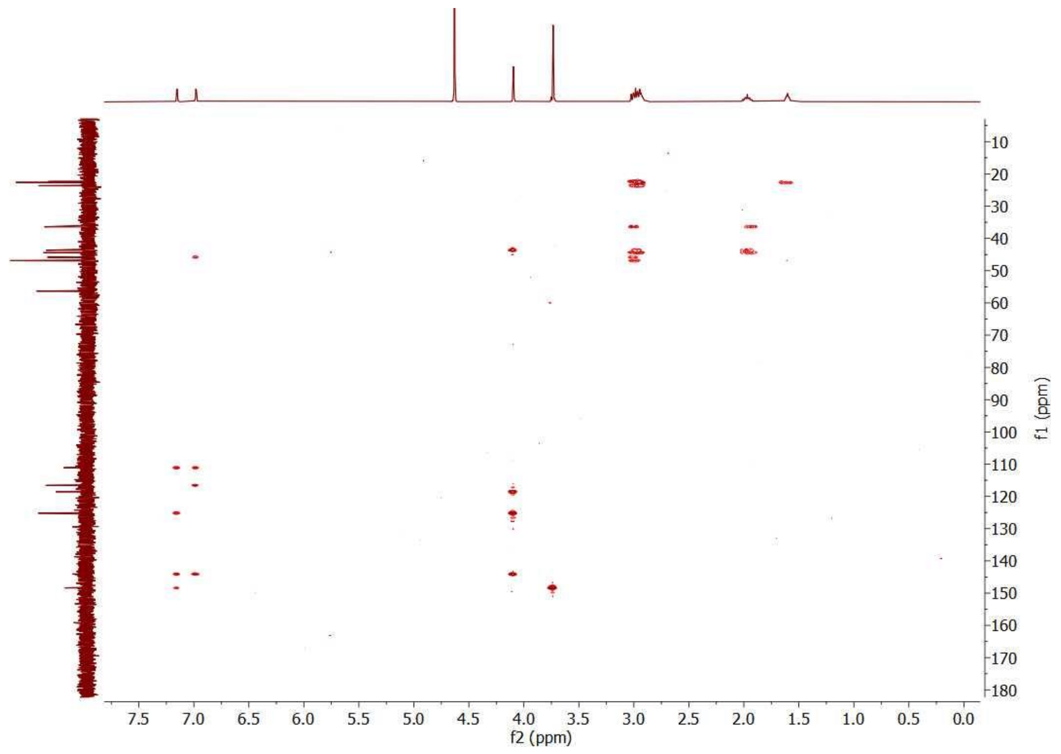

HSQC:

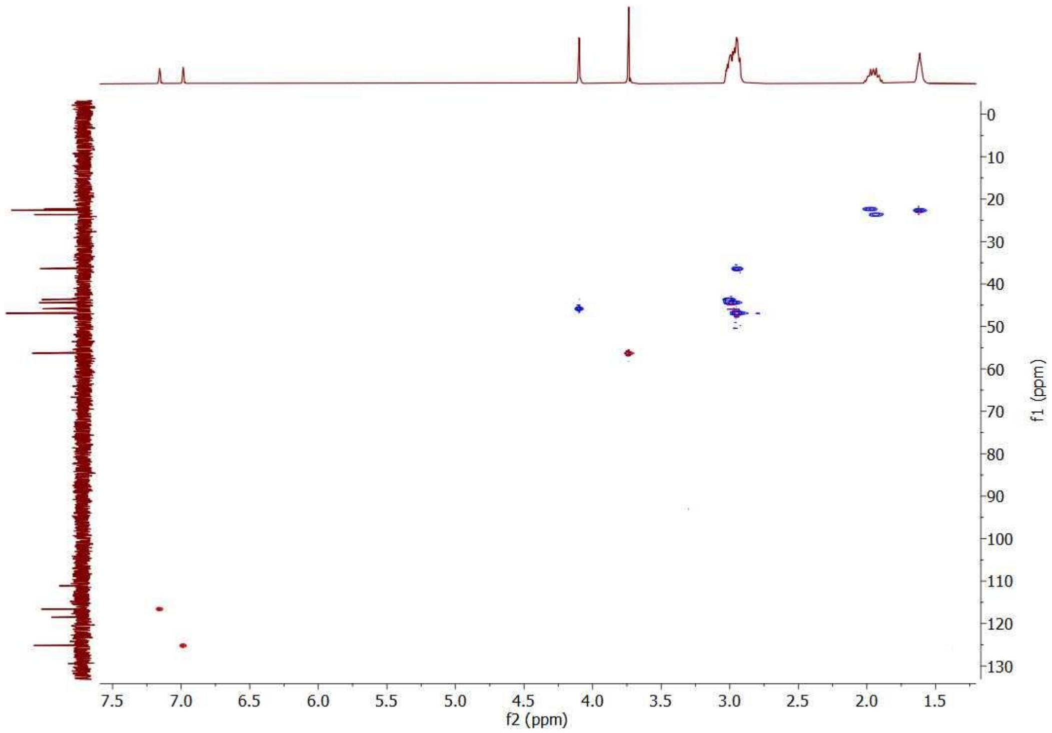

HPLC: t_r = 9.5min, Purity at 220nm = 97.3%

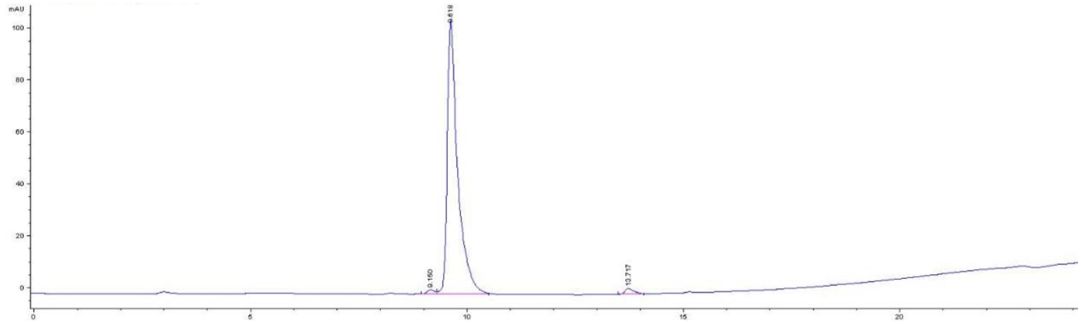

MS: Calculated MS= 416.1787 and 418.1766; Found MS = 417.1704 and 419.1703 $(M + H)^+$

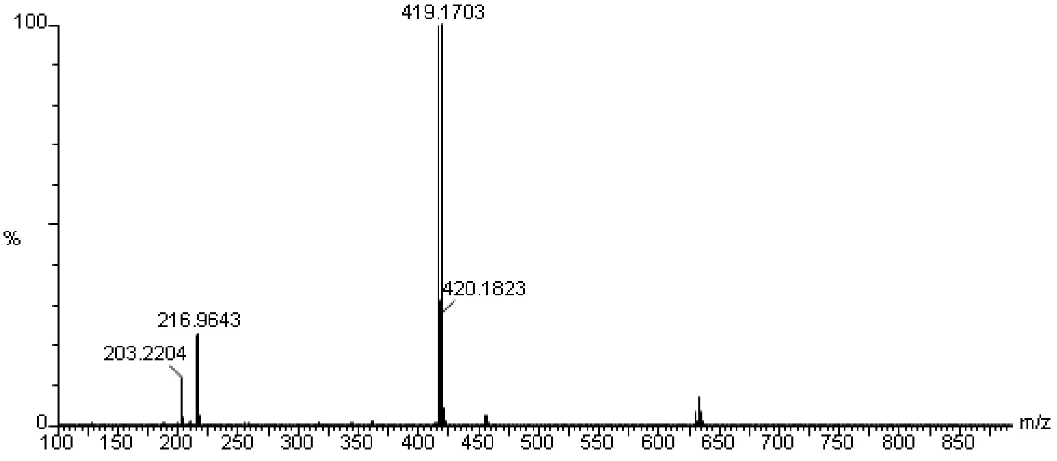

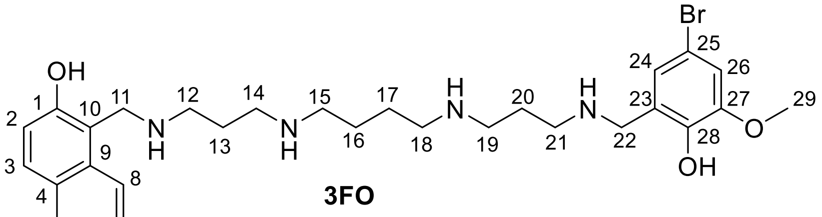

^{1}H RMN (D_2O): δ 7.86 (3H, m, H3, H5, H8), 7.54 (1H, dd, H6, J=7.5Hz), 7.36 (1H, dd, H7, J=7.5Hz), 7.18 (2H, m, H2, H24), 7.01 (1H, d, H26, J=2Hz), 4.64 (2H, s, H11), 4.12 (2H, s, H22), 3.88 (3H, s, H29), 3.12 (2H, m, H12, H21), 3.00 (10H, m, H14, H15, H18, H19) 2.03 (4H, m, H13, H20), 1.63 (4H, m, H16, H17)

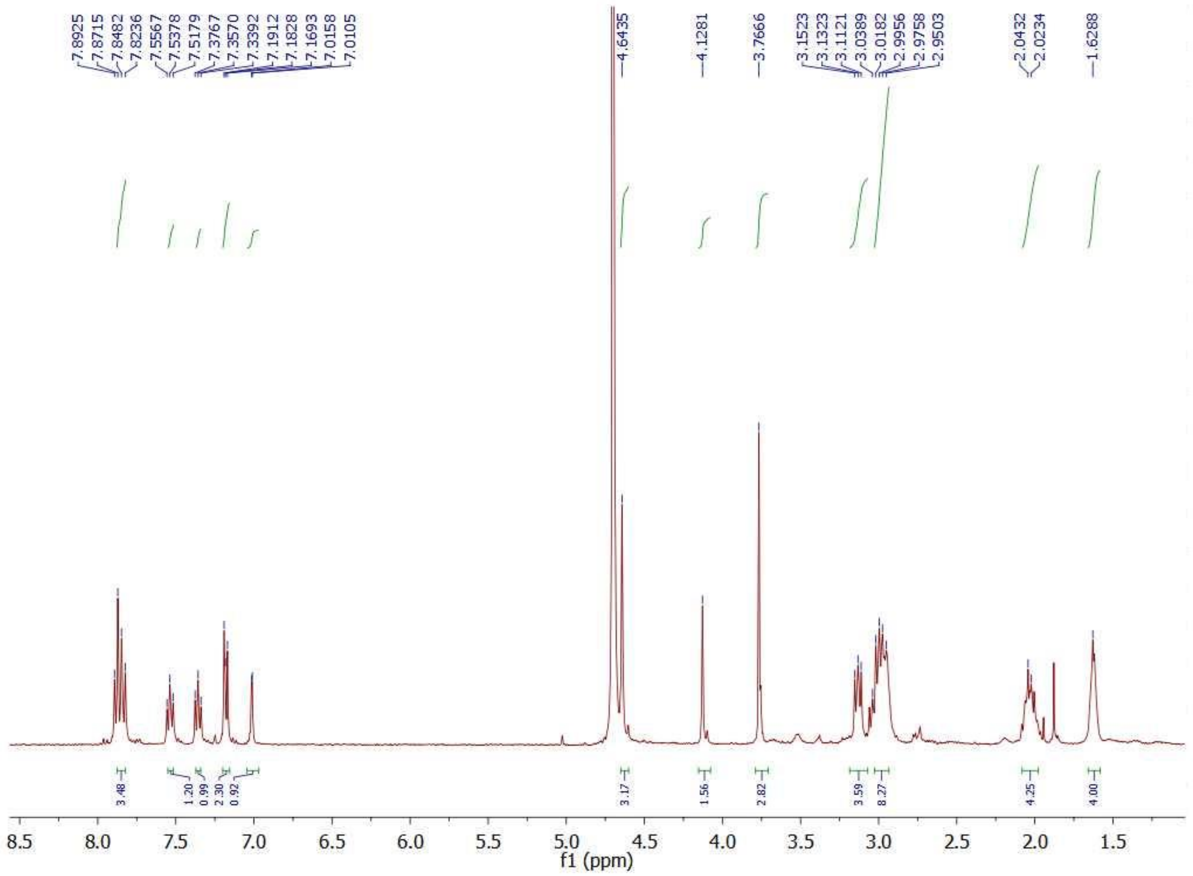

^{13}C RMN (D_2O): δ 154.2 (C1), 148.4 (C27), 144.4 (C28), 132.5 (C4), 132.1 (C5), 129.0 (C3), 128.4 (C9), 127.9 (C6), 125.2 (C26), 123.7 (C7), 121.2 (C8), 117.3 (C2), 116.6 (C24), 114.8 (C23), 111.2 (C25), 108.2 (C10), 56.3 (C29), 46.9 (C22), 44.4(C14, C19) , 43.8 (C15, C18), 41.6 (C11, C21), 22.6 (C16, C17), 22.5 (C13, C20).

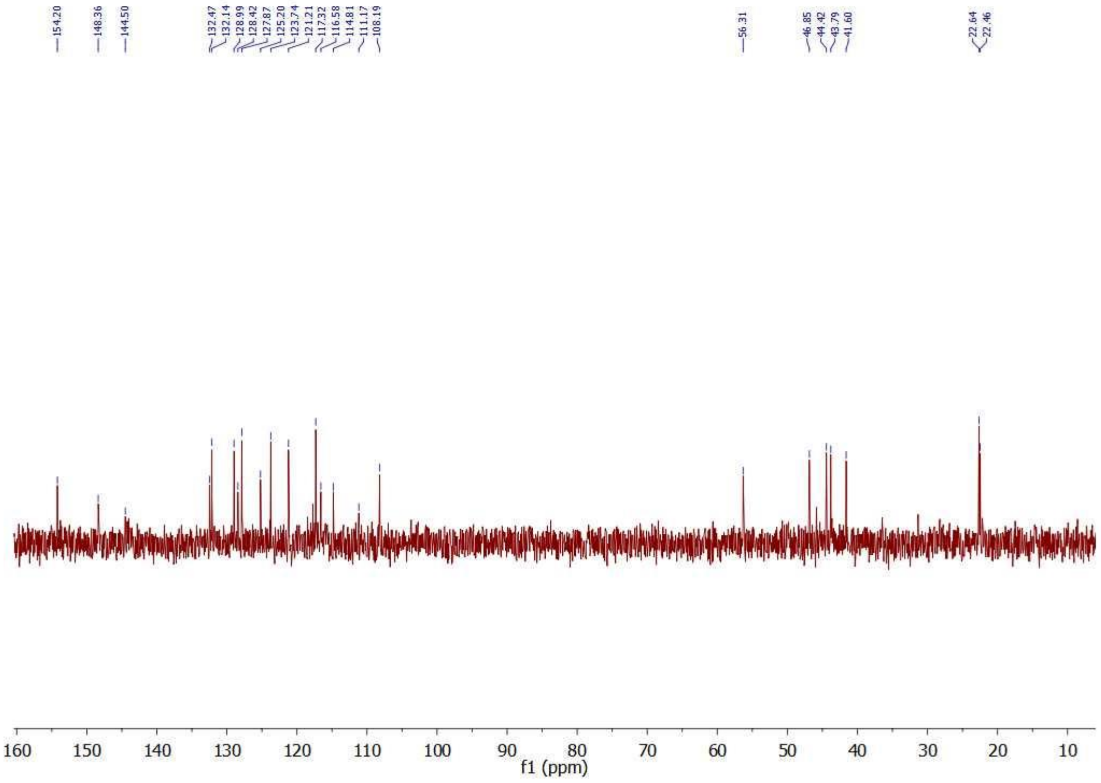

HMBC:

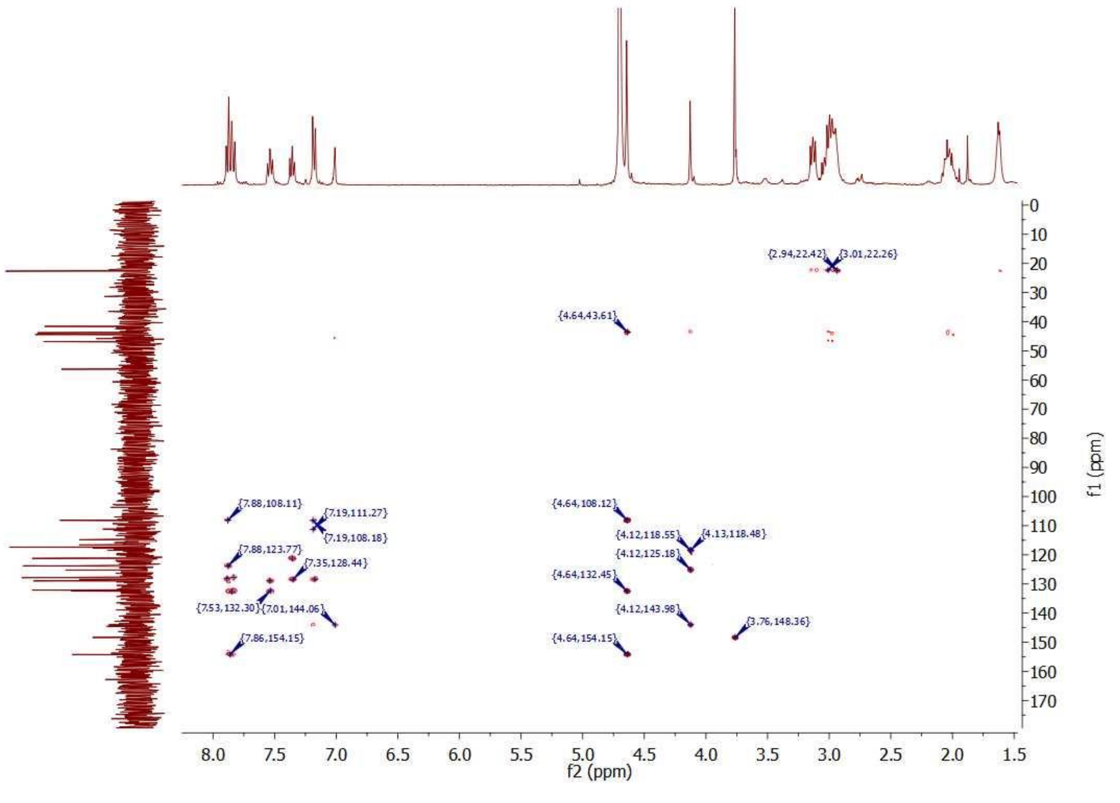

HSQC:

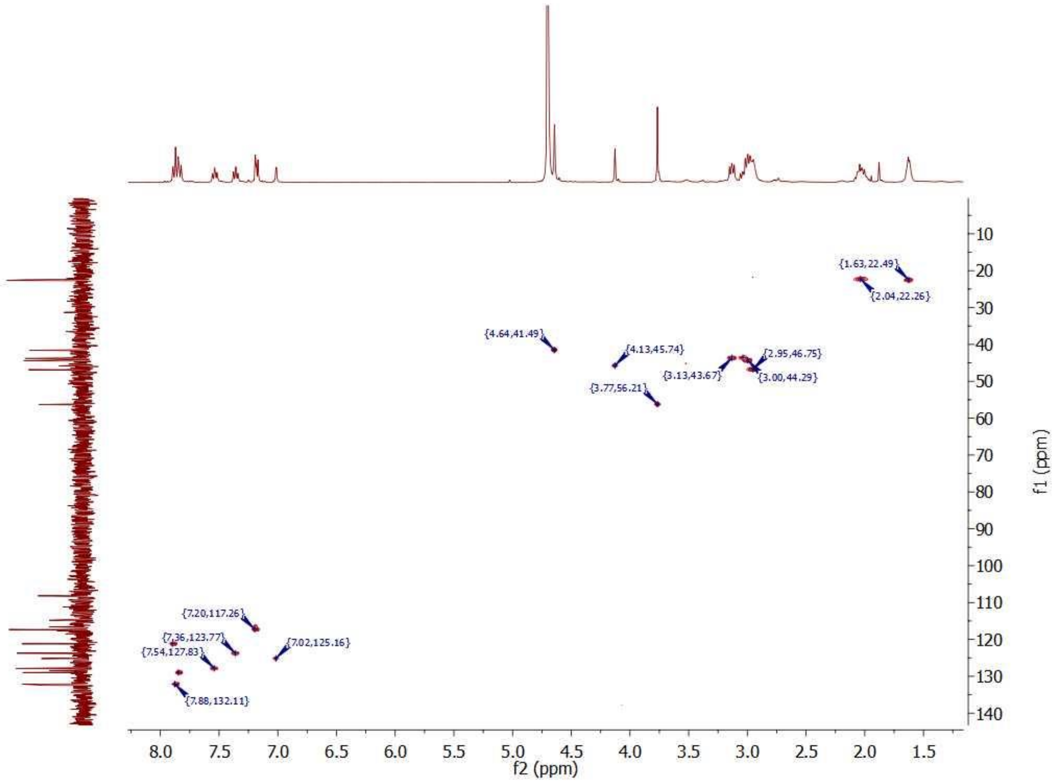

HPLC: t_r = 9.5min. Purity at 220nm >99%

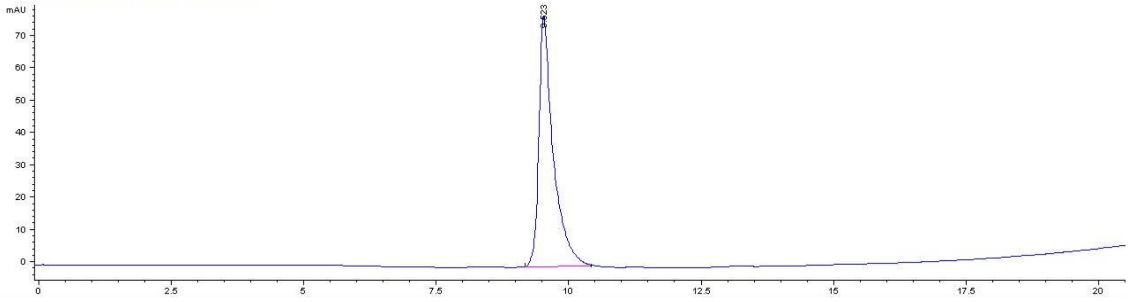

MS: Calculated MS= 572.2362 and 574.2342; Found MS = 573.2471 and 575.2441 $(M + H)^+$

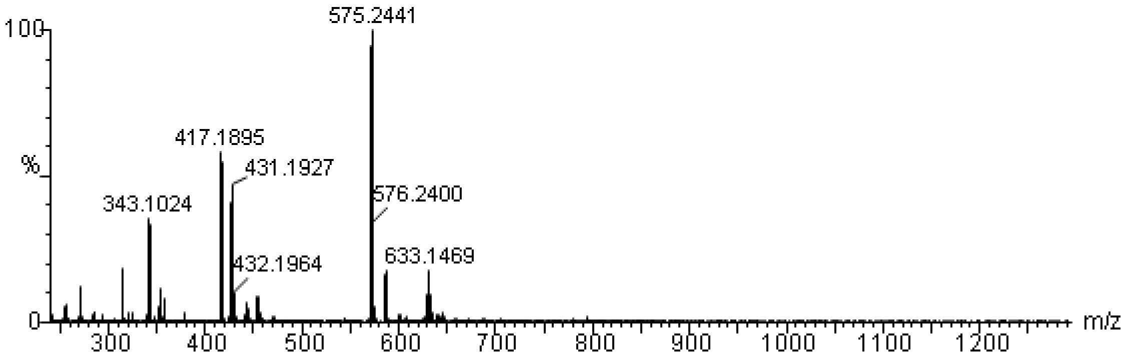

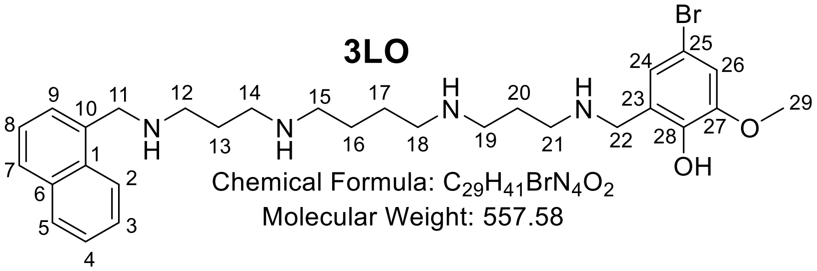

^{1}H RMN (D_2O): δ 7.90 (3H, m, H7, H8, H9), 7.50 (4H, m, H2, H3, H4, H5), 7.14 (1H, d J=4Hz, H24), 6.97 (1H, d, J=4Hz, H26), 4.65 (2H, s, H11), 4.08 (2H, s, H22), 3.73 (3H, s, H29), 3.12 (2H, m, H15), 2.96 (10H, m, H12, H14, H18, H19, H21), 1.98 (4H, m, H13, H20), 1.59 (4H, m, H16, H17)

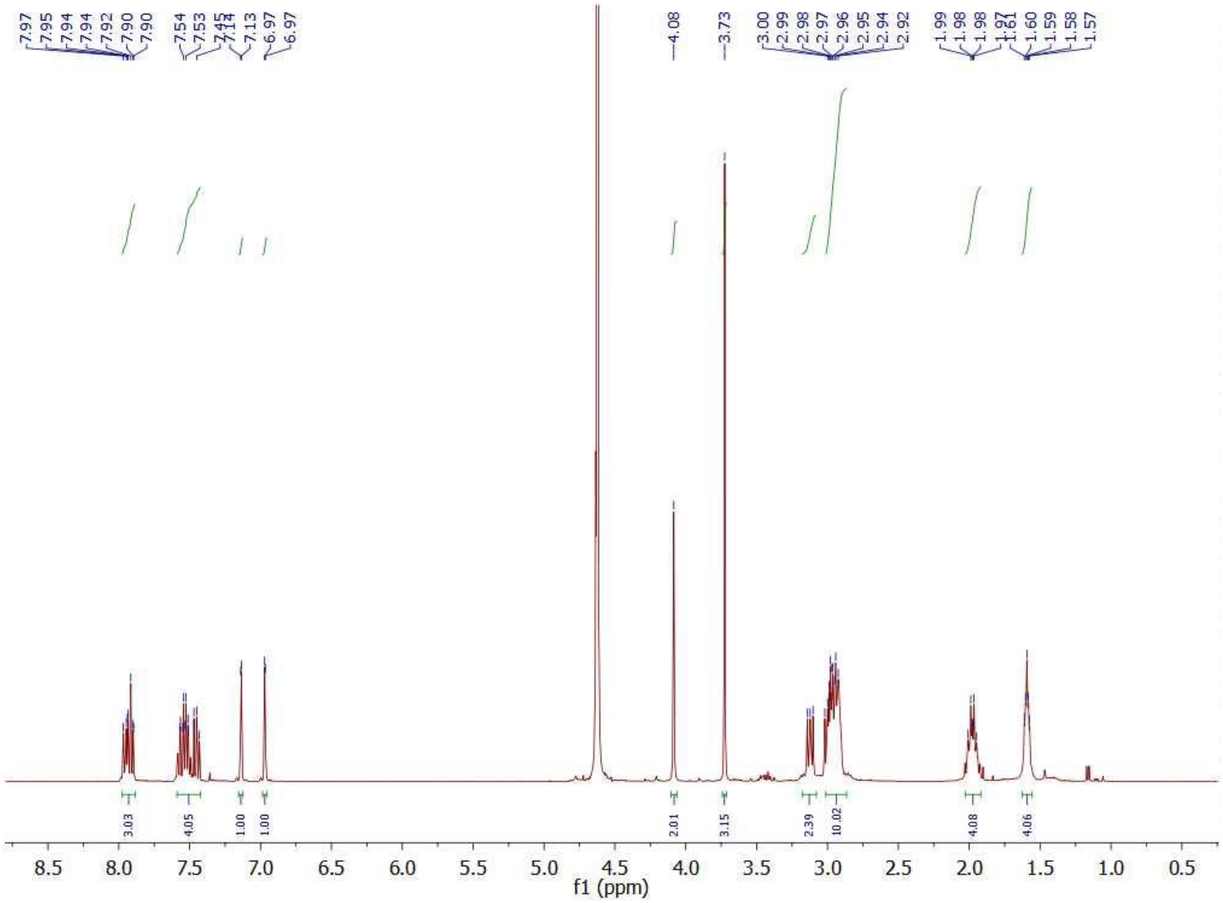

^{13}C RMN (D_2O): δ 148.4 (C28), 144.1 (C27), 133.6 (C10), 130.8 (C6), 130.6 (C9), 129.5 (C5), 129.1 (C8), 127.5 (C3), 126.7(C4), 126.3 (C1), 125.6 (C2), 125.3 (C24), 122.4 (C7), 118.6 (C23), 116.7 (C26), 111.2 (C25), 56.4 (C29), 48.1 (C11), 46.9 (C14), 45.9 (C22), 44.3 (C15, C18, C19, C21), 22.7 (C16, C17), 22.6 (C13), 22.4 (C20).

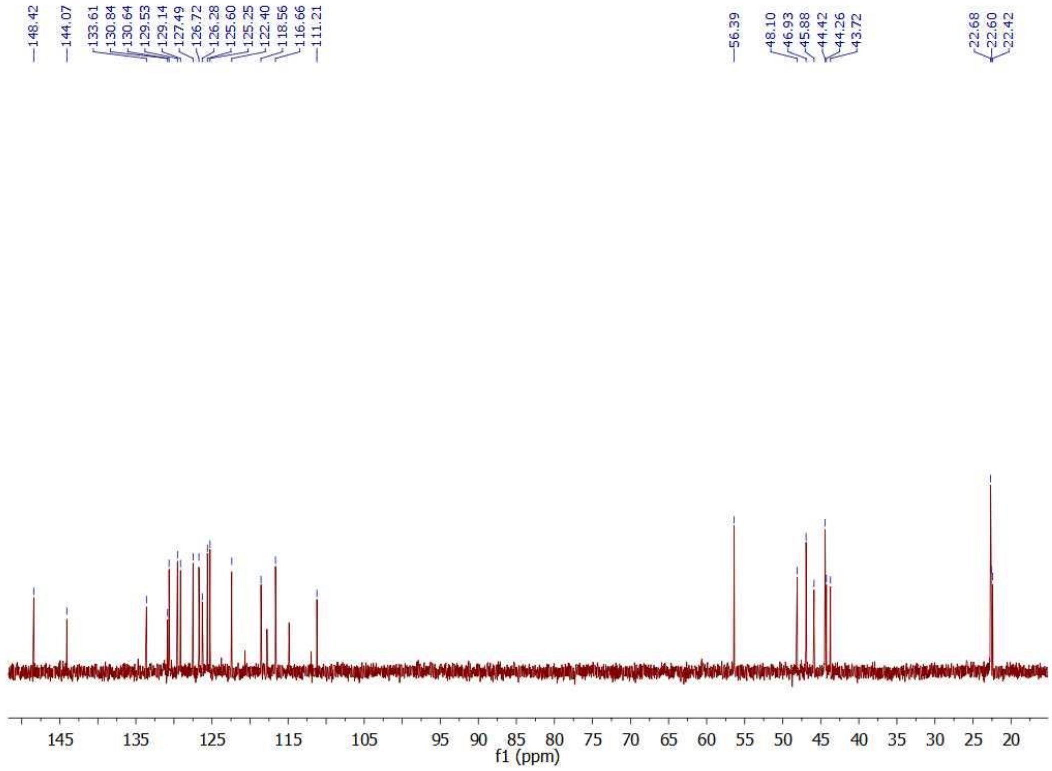

HMBC:

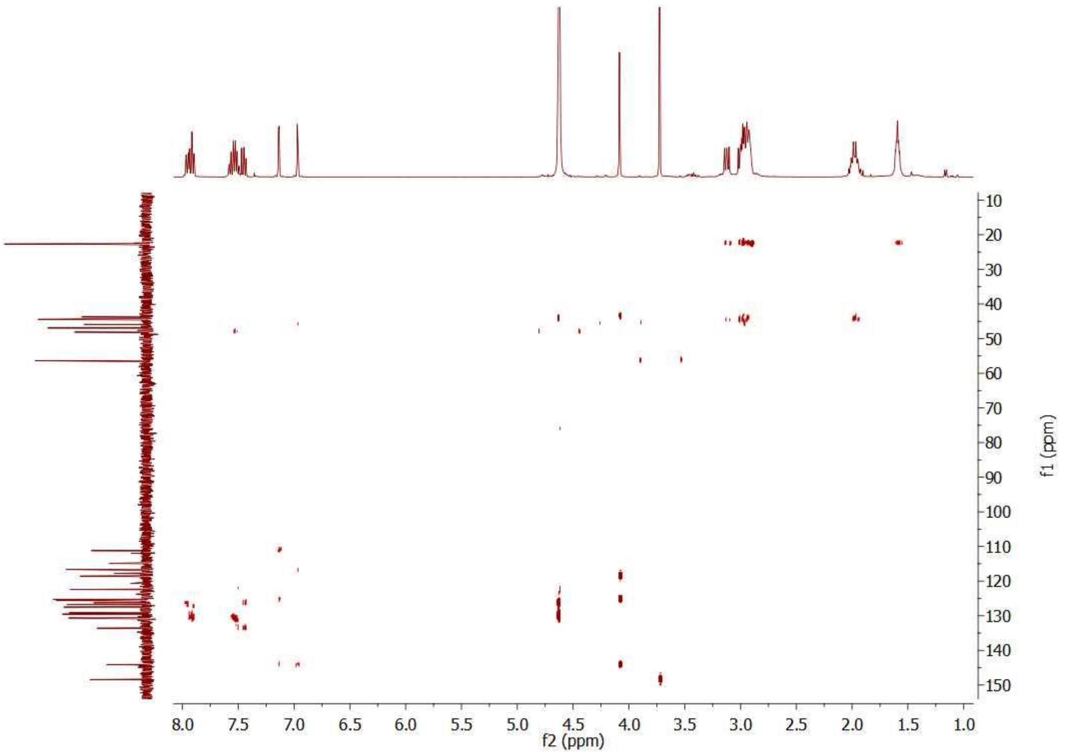

HSQC:

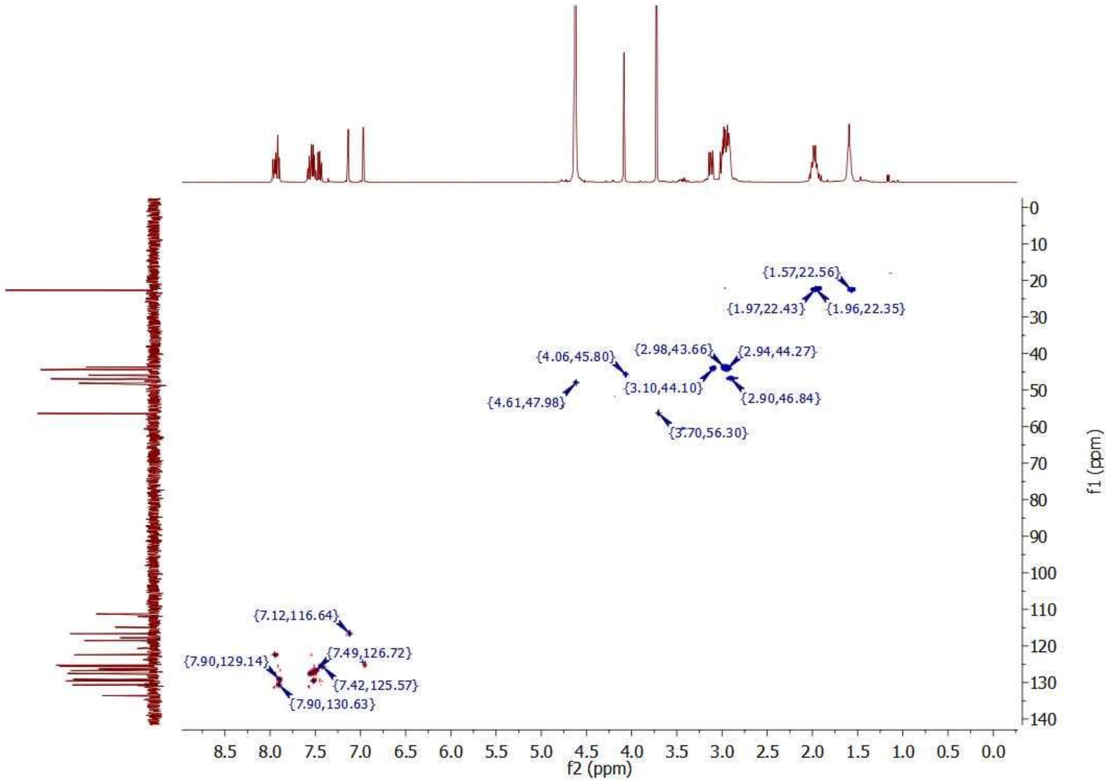

HPLC: t_r = 11.2min. Purity at 220nm 98.7%

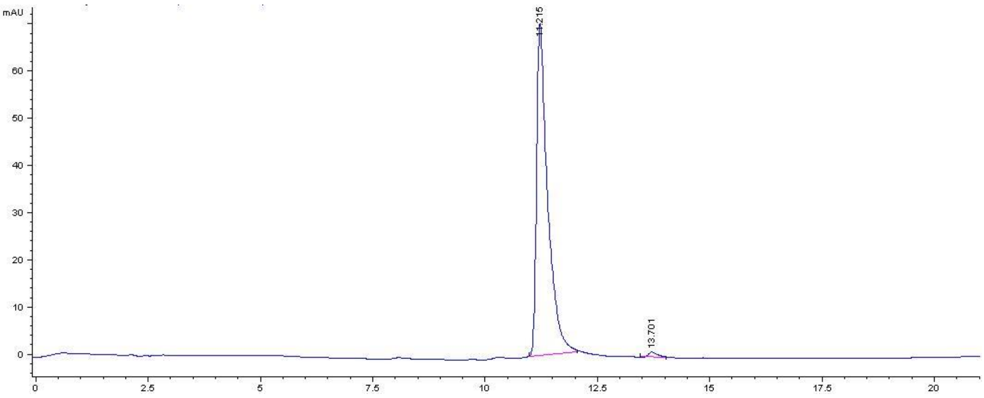

MS: Calculated MS= 556.2413 and 558.2392; Found MS = 557.2440 and 559.2421 $(M + H)^+$

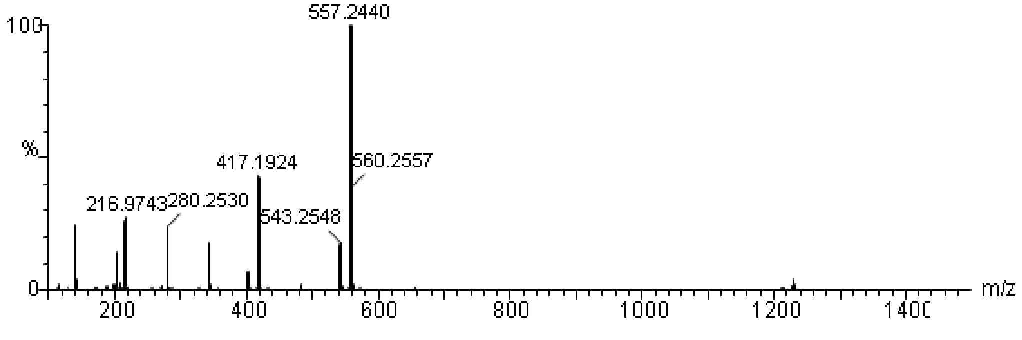

Aggregation studies with 3FF

The aggregation trend of 3FF in aqueous media was studied using three experimental techniques: NMR, fluorescence spectroscopy and DLS. Different parameters were tested, such as pH, time, temperature and overall concentration of the samples.

Aggregation of 3FF by NMR.

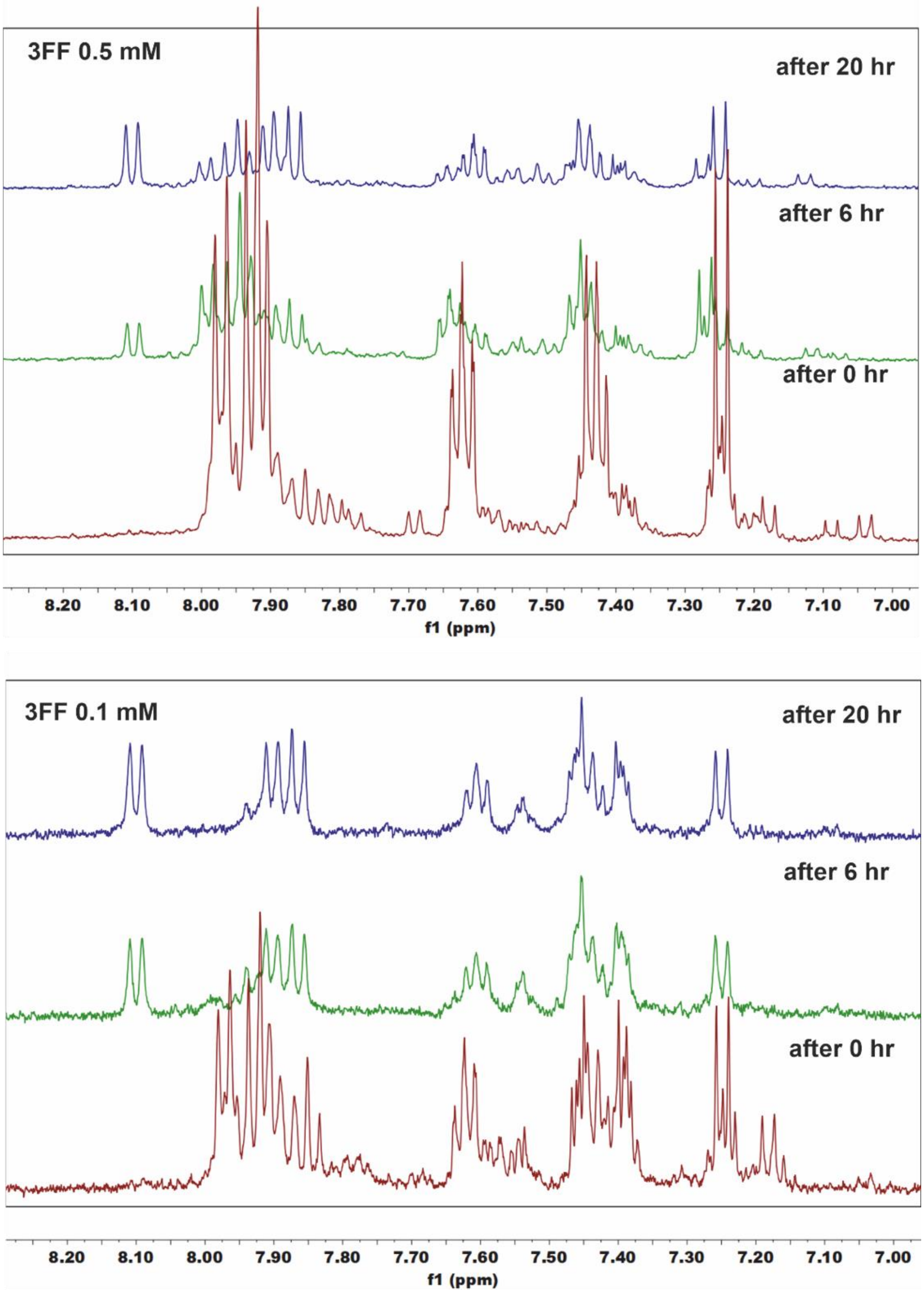

Figure S2. Effect of the overall concentration of 3FF: 1D ^{1}H NMR spectra of 3FF at two concentrations, 0.1 (Bottom) and 0.5 mM (Top). NMR samples prepared in aqueous buffer (5 mM Tris/50 mM NaCl pH* 7.2) from stored samples, so we already observe aggregation just after dilution from stock solution (prepared in DMSO-d6).

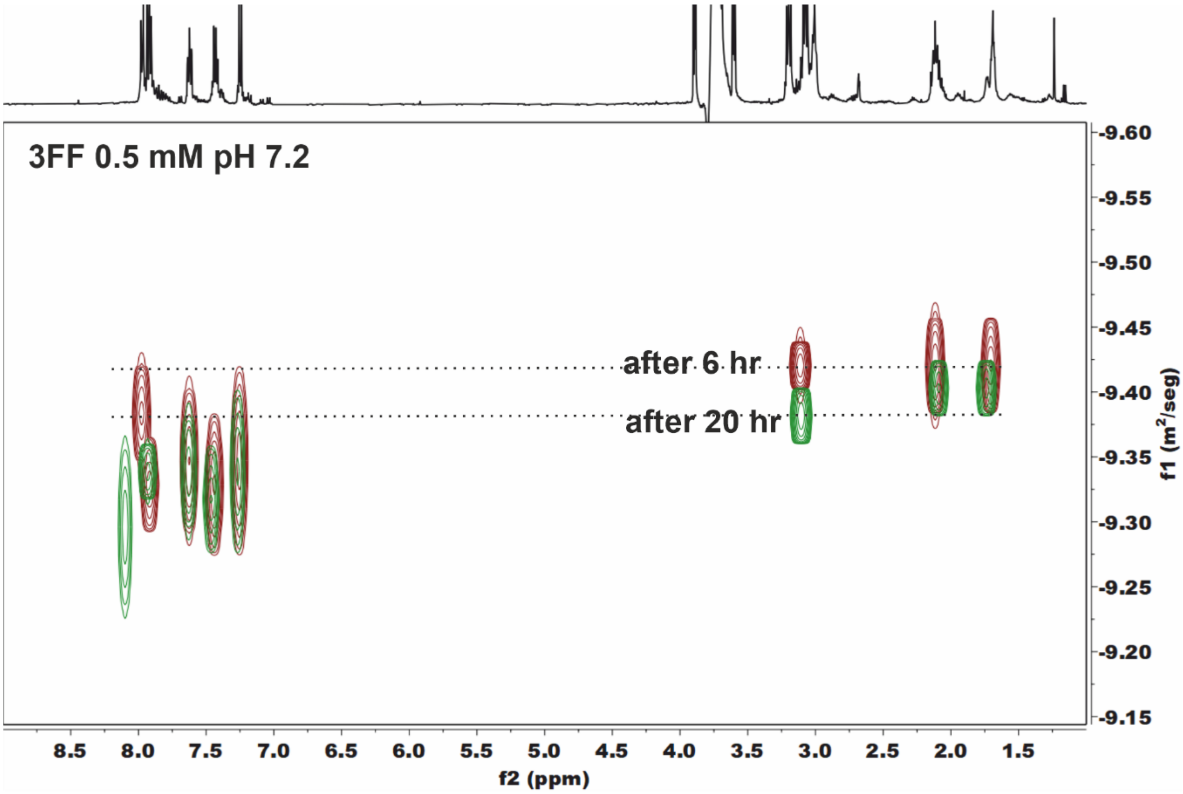

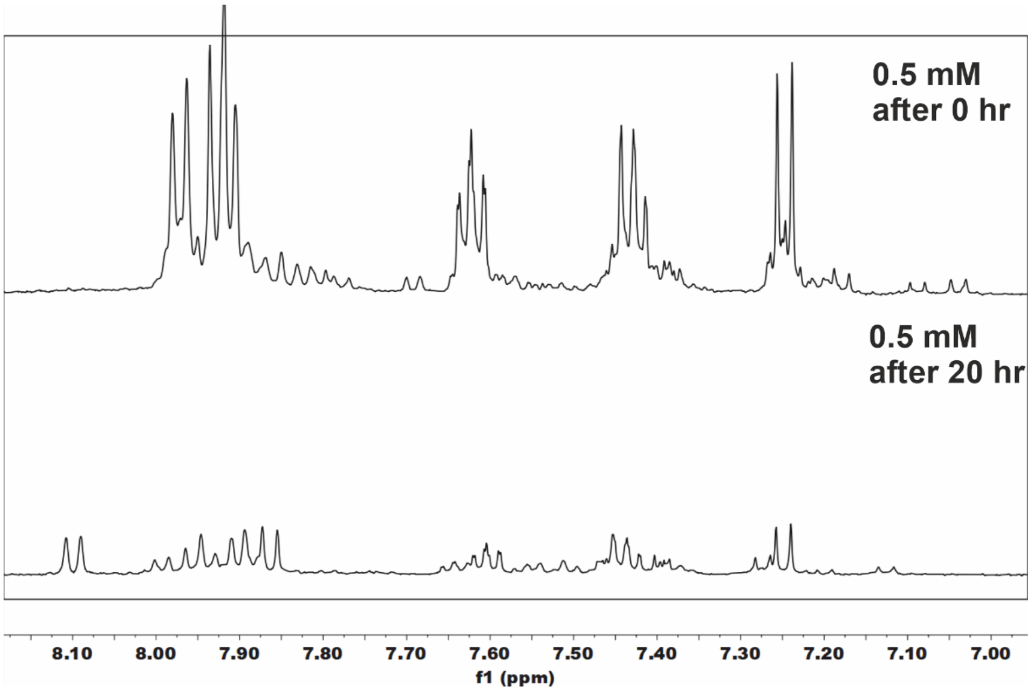

Figure S3. Time dependent aggregation of 3FF. (Top) 0.5 mM 3FF 2D DOSY (Δ = 80 ms) and (bottom) partial 1D ^{1}H NMR spectra at neutral pH at two times after sample preparation (from stored sample, pH* 7.2, 298K, 5 mM Tris-d11, 50 mM NaCl). The corresponding D values are displayed in Table S7.

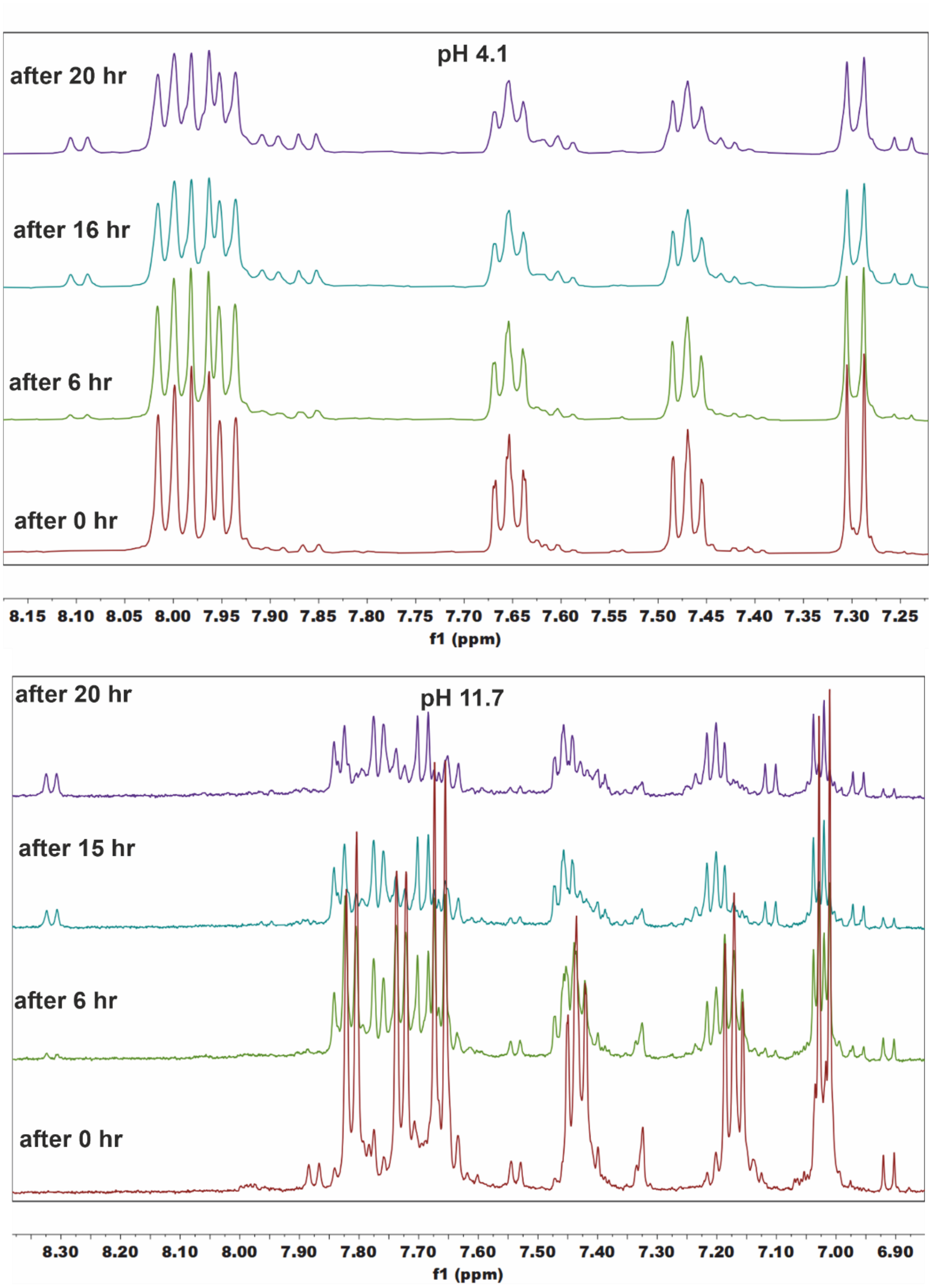

Figure S4. Effect of the pH on the aggregation of 3FF. (Top) 0.5 mM 3FF 1D ^{1}H NMR spectra at pH* 4.1 and (bottom) pH* 11.7 (from stored sample, 5 mM Tris/50 mM NaCl, 298K).

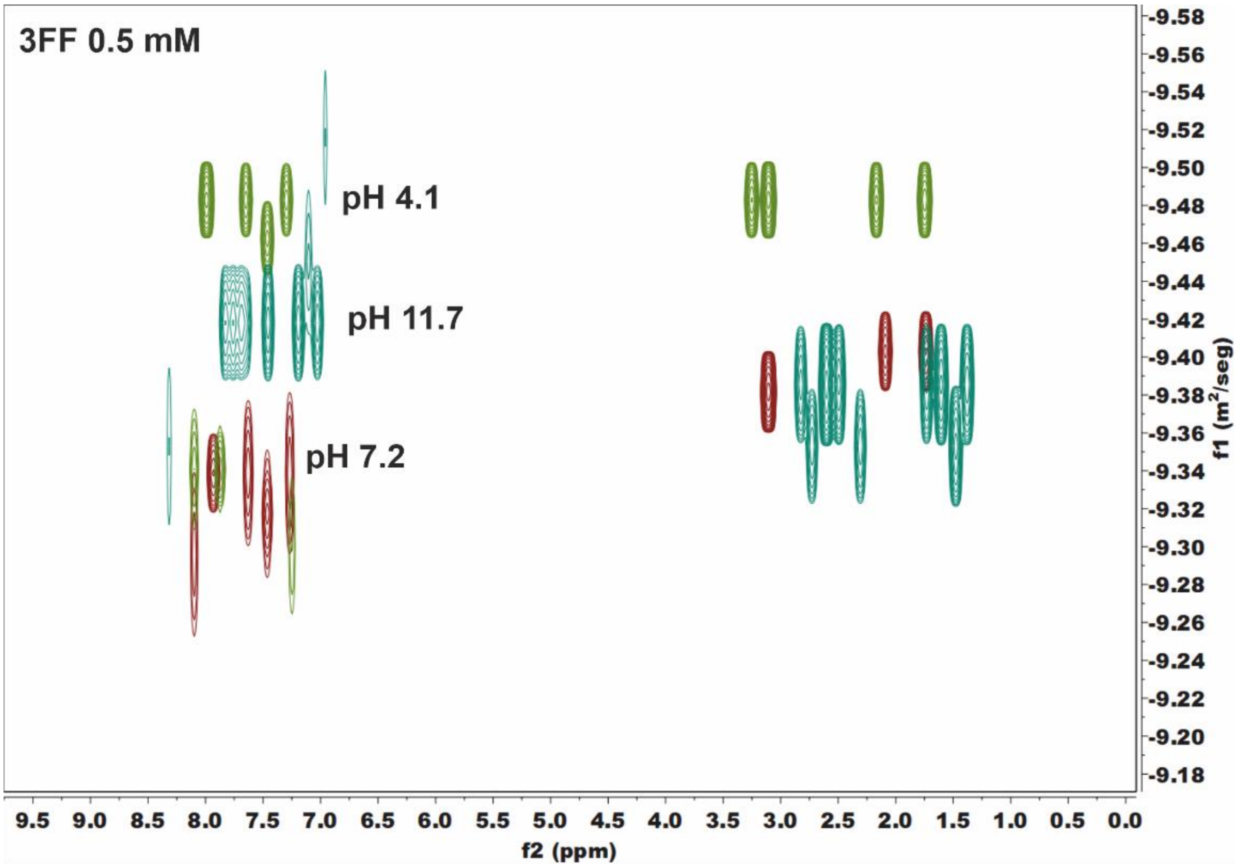

Figure S5. Effect of the pH on the aggregation of 3FF. Overlay of the 2D DOSY (Δ = 80 ms) NMR spectra of 0.5 mM 3FF at different pH values (after 20 h from stored sample, 298 K, 5 mM Tris-d11, 50 mM NaCl). The corresponding D values are displayed in Table S7.

Aggregation of 3FF by fluorescence spectroscopy

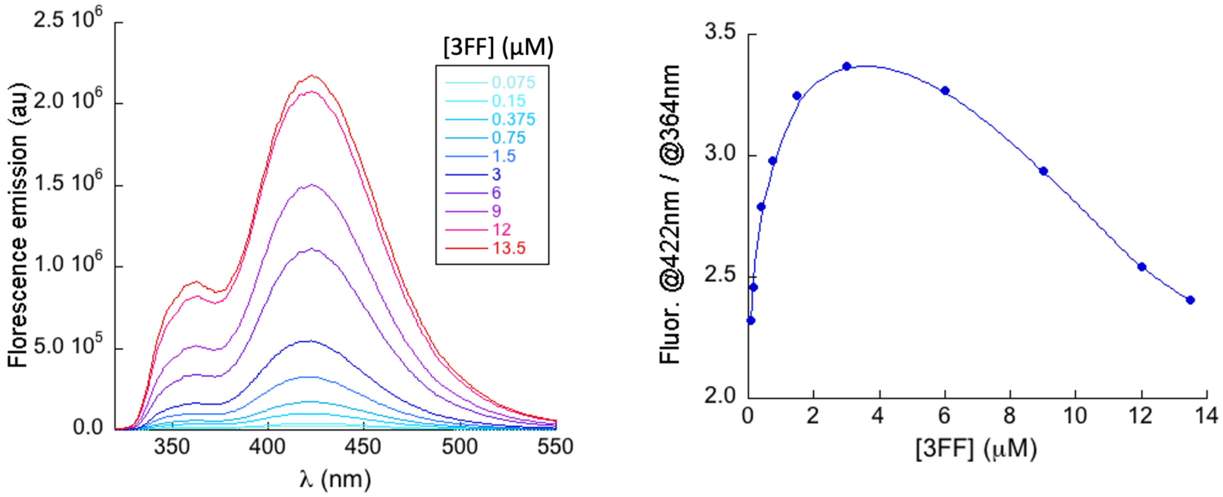

Figure S6. Effect of the concentration on the aggregation of 3FF: Fluorescence spectra (left) of 3FF in Tris buffer at pH 7.1 at different concentrations. The ratio between the excimer (422 nm) and monomer (364 nm) emission bands of the naphthol fluorophore (right) shows a complex dependence on the overall concentration of 3FF, which is an indication of aggregation in solution, especially when [3FF] > 4 μM.

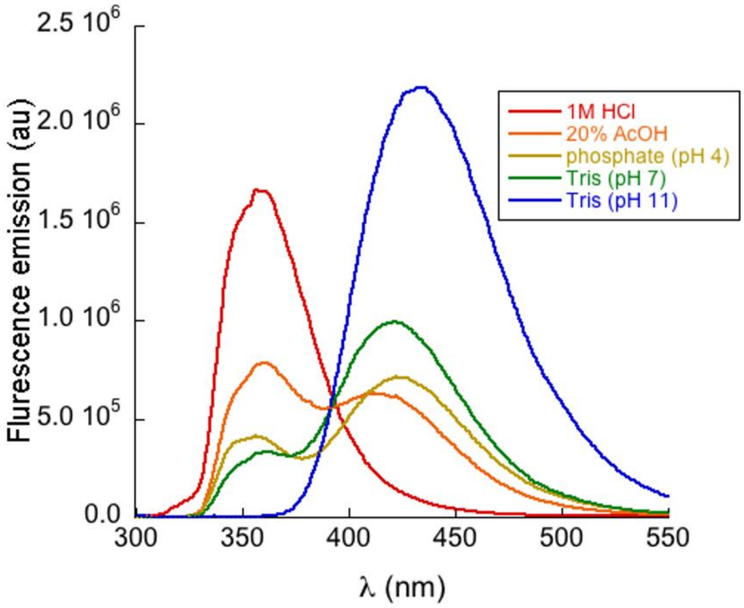

Figure S7. Fluorescence emission spectra of 3FF (10 μM) at different pH values. The spectra show a higher amount of the excimer when increasing the pH.

Aggregation of 3FF by Dynamic Light Scattering (DLS).

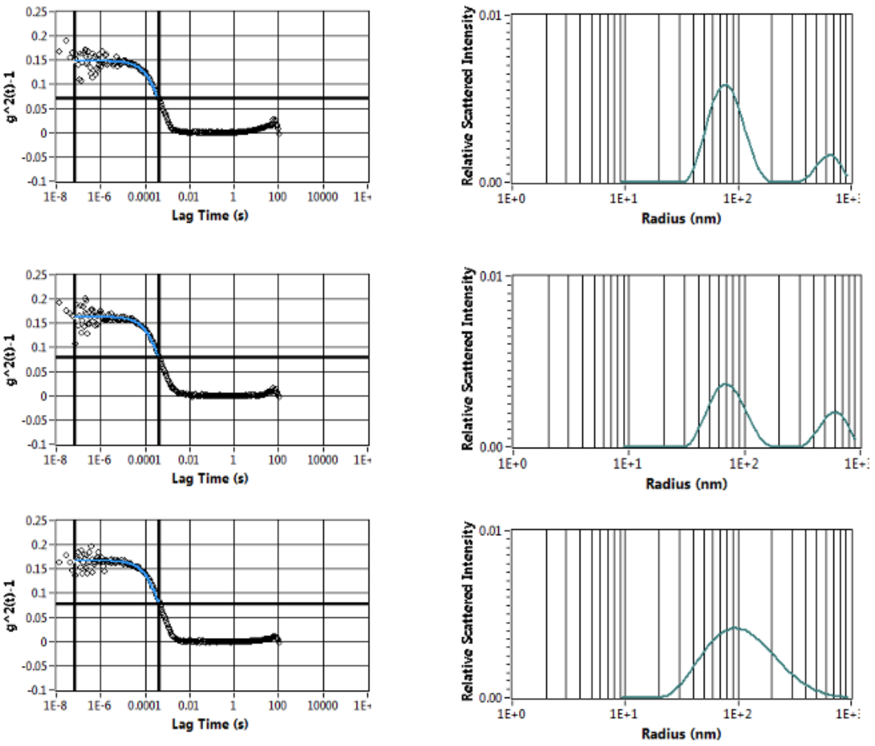

Figure S8. Autocorrelation function (left) and particle size distribution (right) obtained from three DLS experiments performed with 20 μM solutions of 3FF in 3 mM BisTris buffer at pH 7.5.

Table S3. Hydrodynamic radius and polydispersity index obtained from the analyses.

Sample	Replicate	Rh (nm)	PI
	**1**	90.2	0.1
**3FF**	**2**	93.5	0.3
	**3**	91.0	0.3
	**mean**	**91.6 ± 1.7**	**0.2 ± 0.1**

We repeated the DLS measurements after 48 hours of sample preparation and, despite we observed scattering, the autocorrelation function did not allow the accurate determination of the particle size. The obtained scattering suggests the presence of bigger particles after 48 hours, implying a further aggregation in solution at longer times.

We additionally carried out DLS measurements at either pH 4.0 or at lower at concentration of 3FF (1 μM and 4 μM). The scattering data showed the absence of detectable particles in these conditions, implying that aggregation of 3FF depends on its overall concentration and on the pH of the medium.

Stability of 3AC in aqueous solution

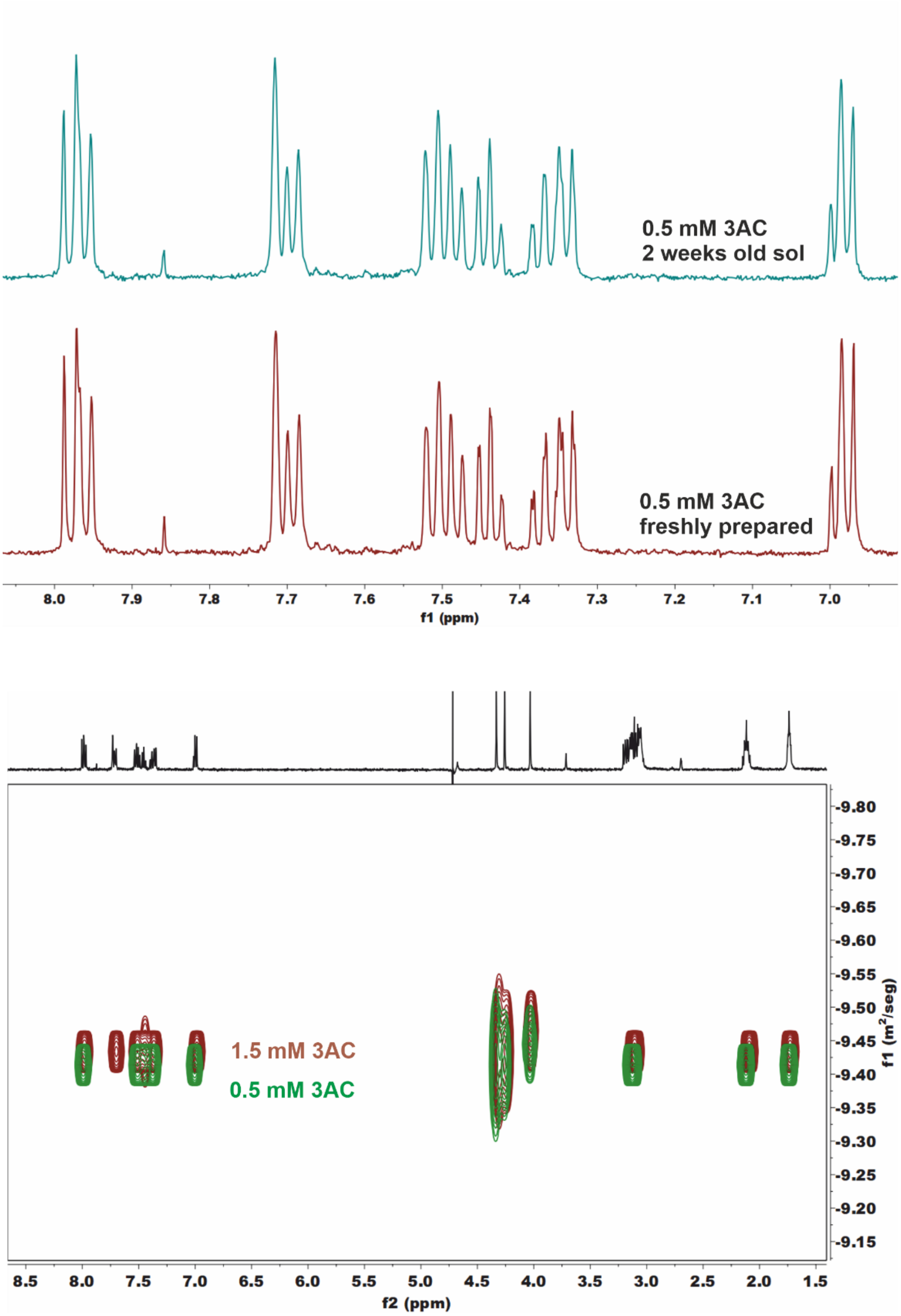

Figure S9. (Top) In contrast with 3FF, 3AC is stable in aqueous solution (0.5 mM, 303, pH* 7.5 mM Tris-d11, 50 mM NaCl) and (bottom) 2D DOSY (Δ = 80 ms) NMR spectra of 3AC at two concentrations. The corresponding D values are displayed in Table S7.

Binding studies on a heparin functionalized SPR chip

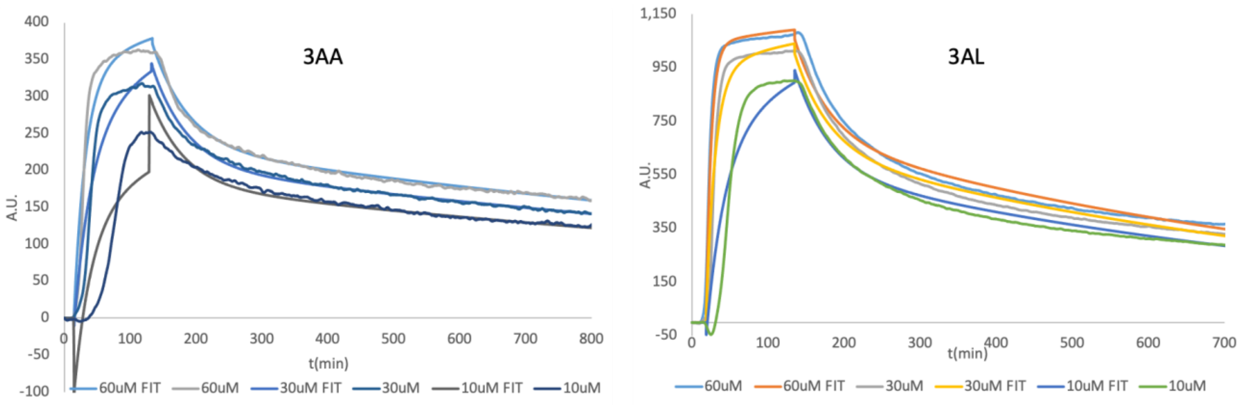

Figure S10. Selected SPR sensorgrams at different concentrations (10 μM, 30 μM and 60 μM, using 25 mM Tris buffer at pH 7.5) of the binders (3AA and 3AL) with the corresponding global fitting to a ‘one-site two -stages’ binding model. Although only one experiment is shown at each concentration, the measurements were done in triplicate and all the corresponding sensorgrams were used for the global fitting.

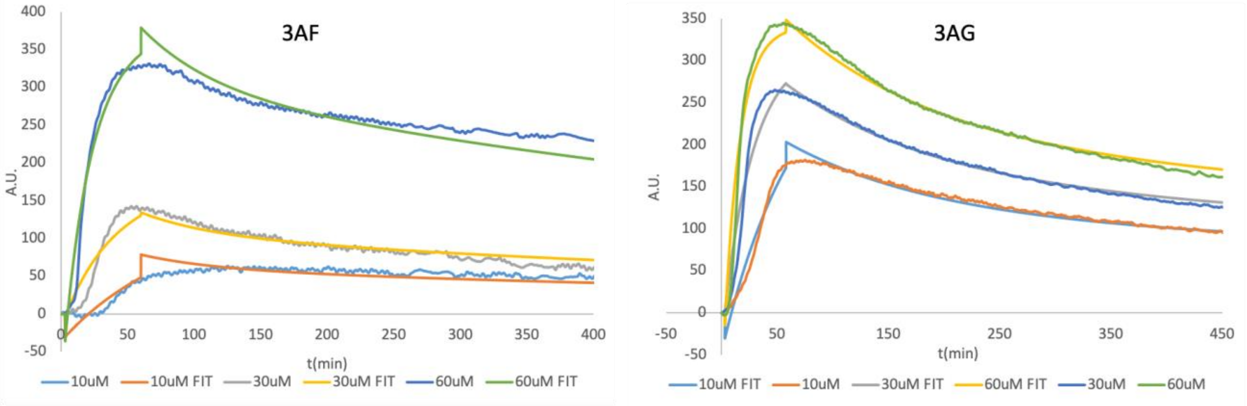

Figure S11. Selected SPR sensorgrams at different concentrations (10 μM, 30 μM and 60 μM, using 25 mM Tris buffer at pH 7.5) of the binders (3AF and 3AG) with the corresponding global fitting to a ‘one-site two-stages’ binding model. Although only one experiment is shown at each concentration, the measurements were done in triplicate and all the corresponding sensorgrams were used for the global fitting.

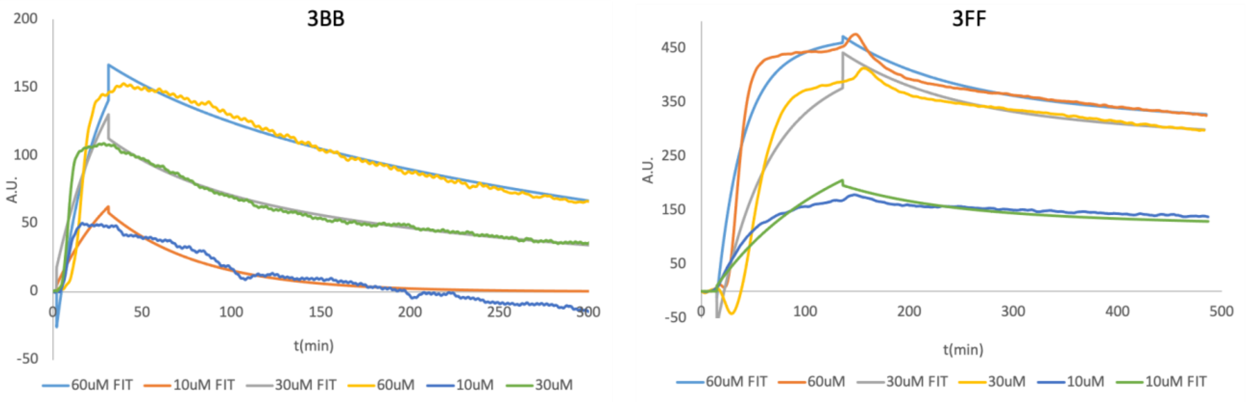

Figure S12. Selected SPR sensorgrams at different concentrations (10 μM, 30 μM and 60 μM, using 25 mM Tris buffer at pH 7.5) of the binders (3BB and 3FF) with the corresponding global fitting to a ‘one-site two-stages’ binding model. Although only one experiment is shown at each concentration, the measurements were done in triplicate and all the corresponding sensorgrams were used for the global fitting.

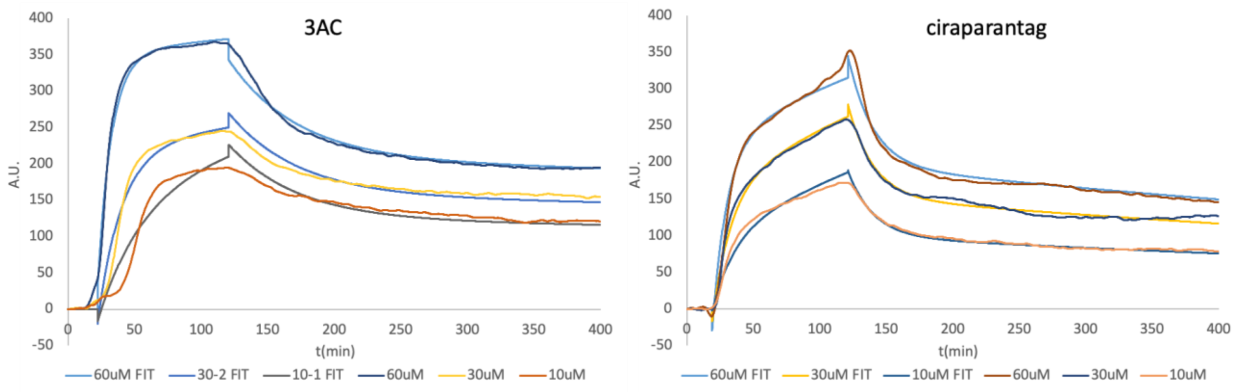

Figure S13. Selected SPR sensorgrams at different concentrations (10 μM, 30 μM and 60 μM, using 25 mM Tris buffer at pH 7.5) of the binders (3AC and ciraparantag) with the corresponding global fitting to a ‘one-site two-stages’ binding model. Although only one experiment is shown at each concentration, the measurements were done in triplicate and all the corresponding sensorgrams were used for the global fitting.

Binding studies in solution

Interaction between 3AC and Hep in solution:

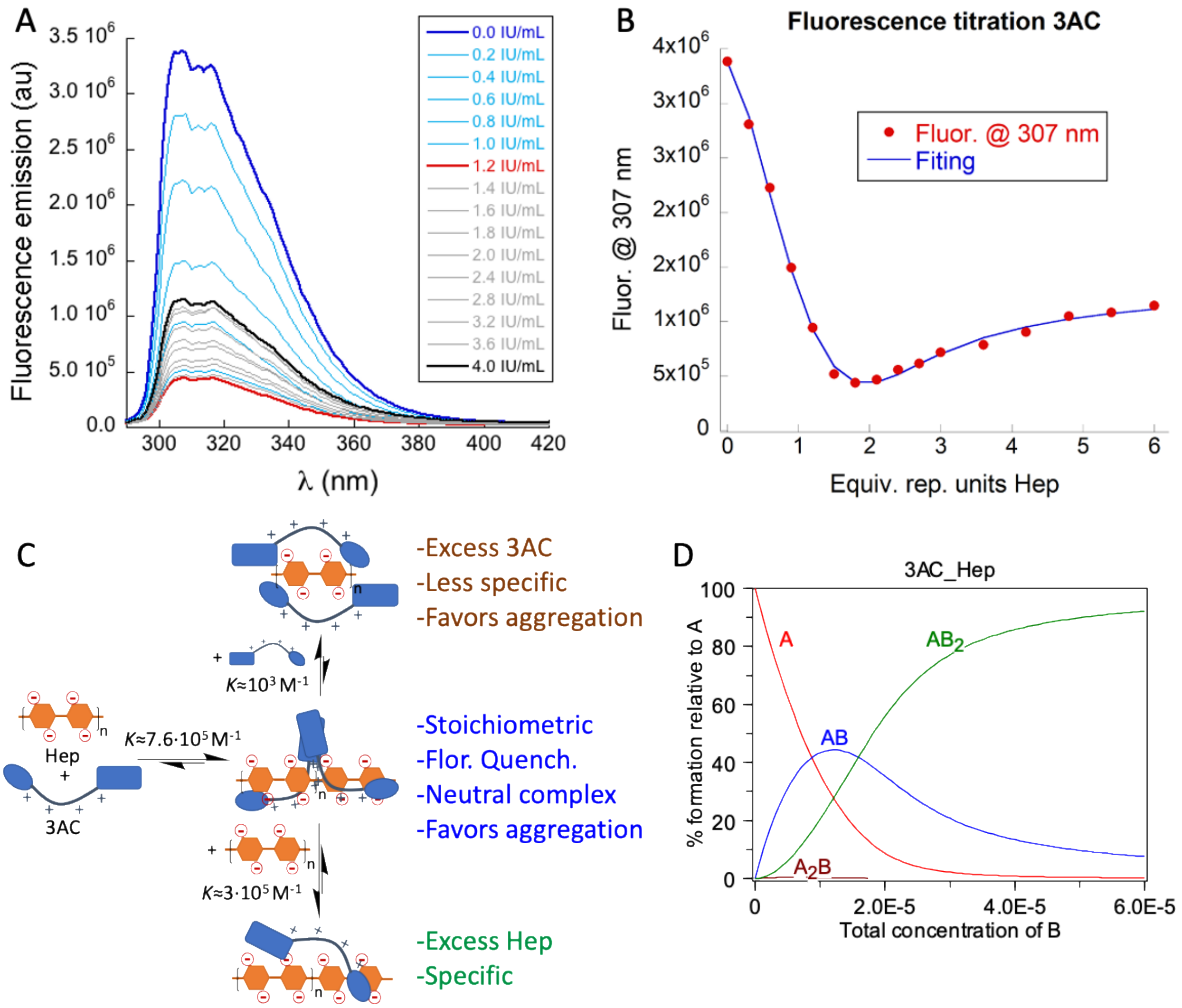

Figure S14. (A) Fluorescence emission spectra of 3AC (10 µM in 1 mM Bis-Tris buffer at pH 7.5) upon the addition of increasing amount of unfractioned Hep. (B) Fluorescence titration isotherm (emission at 307 nm) with the experimental points in red symbols and the non-linear fitting as the blue curve. (C) Schematic representation of the binding mode between 3AC and Hep used for the fitting shown in (B). (D) Simulation plot (Hyss) for the species distribution calculated with the equilibrium constants obtained from the fitting to the model shown in (C)

For the quantitative analysis of the titration data, we considered the disaccharide repeating unit of Hep as the target for the interaction, and we assumed that all the binding sites are equivalents. Despite being an obvious oversimplification, it is very convenient to propose a simplified interaction model. With these approximations, the binding process here studied can be described with three equilibria corresponding to the formation of three different complexes, as depicted below:

$$3AC + Hep \rightleftharpoons (3AC{\cdot}Hep) \qquad \boldsymbol{\beta}(3AC{\cdot}Hep) = \frac{[3AC{\cdot}Hep]}{[3AC]\,[Hep]}$$

$$3AC + 2Hep \rightleftharpoons (3AC{\cdot}Hep_2) \qquad \boldsymbol{\beta}(3AC{\cdot}Hep_2) = \frac{[3AC{\cdot}Hep_2]}{[3AC]\,[Hep]^2}$$

$$2(3AC) + Hep \rightleftharpoons (3AC_2{\cdot}Hep) \qquad \boldsymbol{\beta}(3AC_2{\cdot}Hep) = \frac{[3AC_2{\cdot}Hep]}{[3AC]^2\,[Hep]}$$

Considering these equilibria, the experimental data was successfully fitted using the HypSpec software to the following formation constants values (standard deviation in the last significant figure is given in brackets):

Logβ(3AC-Hep) = 5.882(5)

Logβ(3AC-Hep_2) = 11.356(9)

Logβ($3AC_2$-Hep) = 8.8(1)

We also aimed to estimate the apparent affinity determined by SPR from the results obtained in the fluorescence titration experiments. We rationalized that the global binding measured by SPR is due to all the possible supramolecular species present in the mixture, thus rendering an apparent association constant defined as:

$$K\text{app} \approx \frac{\sum_{i,j}^{n} [3ACi{\cdot}Hepj]}{[3AC]\,[Hep]}$$

(Equation 1)

And, accordingly, the apparent dissociation constant should be K_D^{app} = 1/*K*app. Using the different species defined in the proposed binding mode and the global formation constants:

$$K\text{app} \approx \frac{[3AC{\cdot}Hep]+[3AC{\cdot}Hep_2]+[3AC_2{\cdot}Hep]}{[3AC]\,[Hep]} =$$

$$= \frac{\boldsymbol{\beta}(3AC{\cdot}Hep)[3AC][Hep]+\boldsymbol{\beta}(3AC{\cdot}Hep_2)[3AC][Hep]^2+\boldsymbol{\beta}(3AC_2{\cdot}Hep)[3AC]^2[Hep]}{[3AC]\,[Hep]}$$

Leading to the apparent binding constant as a function of the overall formation constants for each complex:

$$\boxed{K\text{app} = \boldsymbol{\beta}(3AC{\cdot}Hep) + \boldsymbol{\beta}(3AC{\cdot}Hep_2)\,[Hep] + \boldsymbol{\beta}(3AC_2{\cdot}Hep)\,[3AC]}$$

(Equation 2)

Thus, *K*app depends on the concentration of the free species in solution, which are unknown parameters and variable during the fluorescence titration experiment. The concentration of the species in equilibrium at every titration point can be obtained from the fitting performed with HypSpec. These concentrations were used either in Eq. 1 or Eq. 2 to estimate the K_D^{app}. This is shown for every fluorescence titration point in Table S4:

Table S4: concentrations of the species in equilibrium at every fluorescence titration point obtained by the fitting, and apparent dissociation constant calculated either by Eq.1 or Eq.2.

[3AC] (M)	[Hep] (M)	[3AC-Hep] (M)	[3AC-Hep$_2$] (M)	[3AC$_2$-Hep] (M)	K_D^{app} (1) (M)	K_D^{app} (2) (M)
7.5902e-06	3.7022e-07	2.1409e-06	2.3627e-07	1.6320e-08	1.1740e-06	1.1752e-06
5.6344e-06	8.1148e-07	3.4835e-06	8.4264e-07	1.9713e-08	1.0521e-06	1.0528e-06
4.0526e-06	1.3621e-06	4.2056e-06	1.7076e-06	1.7117e-08	9.3082e-07	9.3126e-07
2.8031e-06	2.0749e-06	4.4312e-06	2.7407e-06	1.2475e-08	8.0956e-07	8.0983e-07
1.8593e-06	3.0189e-06	4.2764e-06	3.8483e-06	7.9855e-09	6.9018e-07	6.9037e-07
1.1909e-06	4.2683e-06	3.8726e-06	4.9272e-06	4.6318e-09	5.7734e-07	5.7747e-07
7.5135e-07	5.8714e-06	3.3611e-06	5.8825e-06	2.5363e-09	4.7712e-07	4.7726e-07
4.7953e-07	7.8203e-06	2.8572e-06	6.6605e-06	1.3760e-09	3.9395e-07	3.9409e-07
3.1610e-07	1.0056e-05	2.4220e-06	7.2604e-06	7.6891e-10	3.2827e-07	3.2842e-07
2.1718e-07	1.2505e-05	2.0691e-06	7.7128e-06	4.5131e-10	2.7763e-07	2.7773e-07
1.1559e-07	1.7799e-05	1.5675e-06	8.3166e-06	1.8196e-10	2.0815e-07	2.0823e-07
6.9917e-08	2.3386e-05	1.2457e-06	8.6842e-06	8.7476e-11	1.6466e-07	1.6473e-07
4.6342e-08	2.9121e-05	1.0282e-06	8.9254e-06	4.7855e-11	1.3558e-07	1.3565e-07
3.2802e-08	3.4939e-05	8.7317e-07	9.0940e-06	2.8766e-11	1.1498e-07	1.1504e-07
2.4374e-08	4.0807e-05	7.5780e-07	9.2178e-06	1.8551e-11	9.9706e-08	9.9753e-08

Both Eq. 1 and Eq. 2 render very similar results. From these numbers we calculated the mean value (± standard error) to be:

K_D^{app} (Fluor.) = 0.50 ± 0.09 μM

Which is in very good agreement with the apparent affinity measured by SPR:

K_D^{app} (SPR) = 0.567 ± 0.013 μM

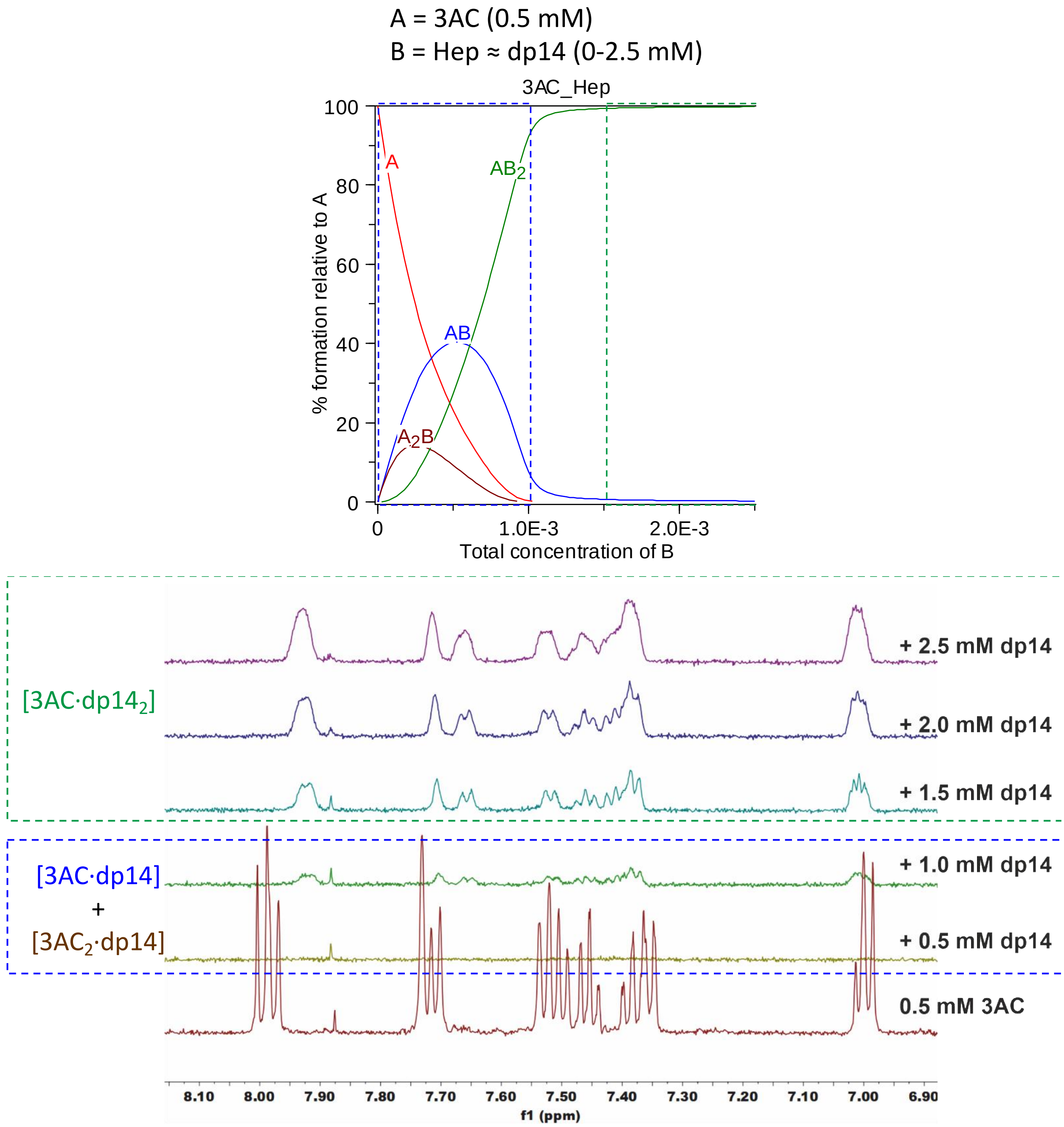

Figure S15. Partial ^{1}H NMR spectra for the titration of 0.5 mM 3AC (pH 7, 303K) with increasing amount of dp14 heparin (concentration at every trace is referred to the corresponding disaccharide units). In the upper part of the figure, we show the species distribution (Hyss) simulated considering the fitted stability constants and the concentration range used in the NMR titration experiments. Selected regions (blue and green dashed rectangles) with the main species are highlighted in both panels for comparison. The presence of the [3AC-dp14] and [3AC$_2$-dp14] species (blue dashed rectangle) leads to disappearance of the signals due to large broadening produced by aggregation. The [3AC-dp14$_2$] complex is observed as a single species at >1 mM dp14 (green dashed rectangle).

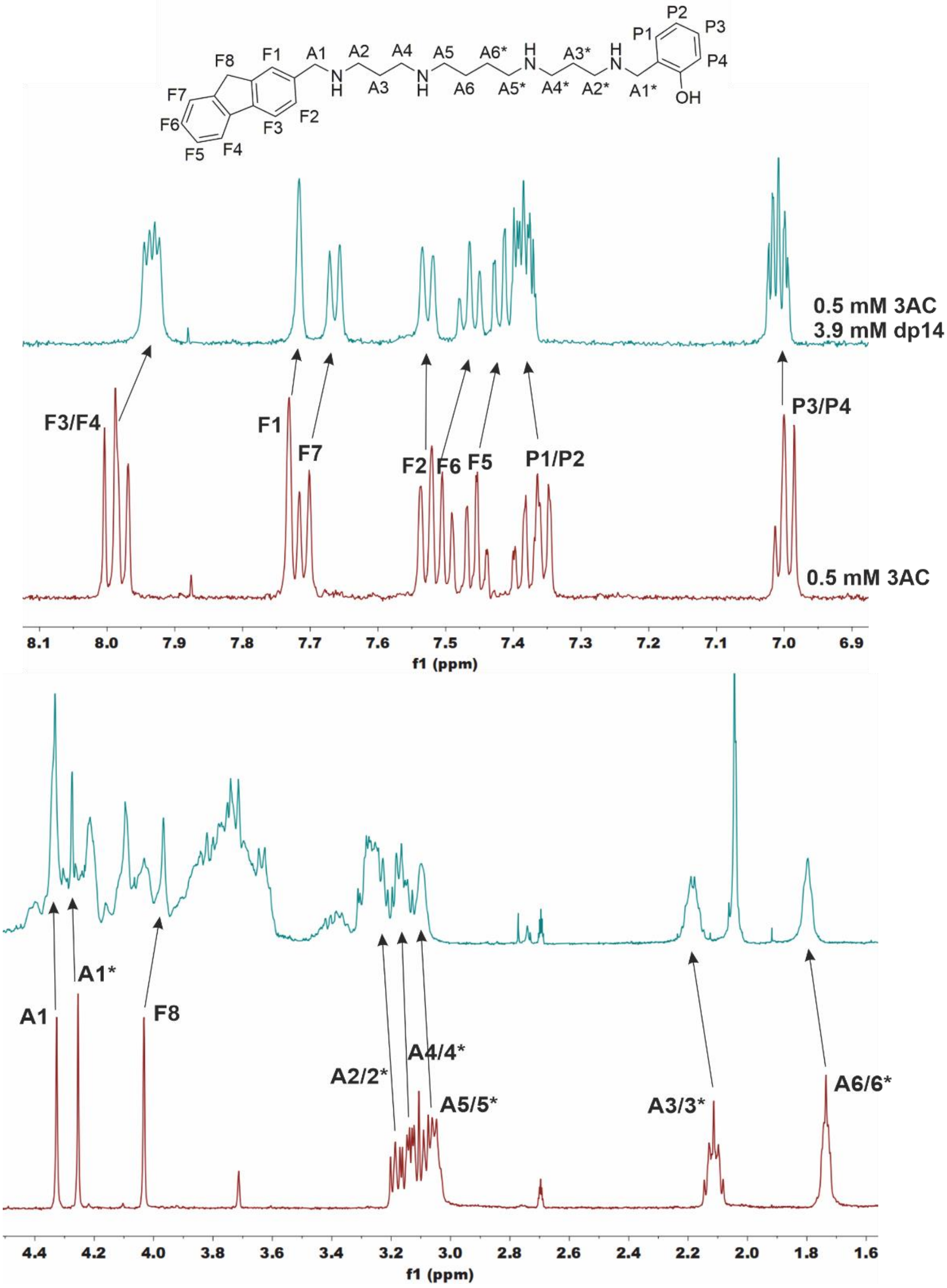

Figure S16. Aromatic (up) and aliphatic (down) regions of the 1D ^{1}H NMR spectra (pH* 7.0, 303K, 5 mM Tris-d11, 50 mM NaCl) of 3AC (0.5 mM) alone (brown trace) and upon addition of an excess of dp14 (3.9 mM in disaccharide repeating units, cyan trace).

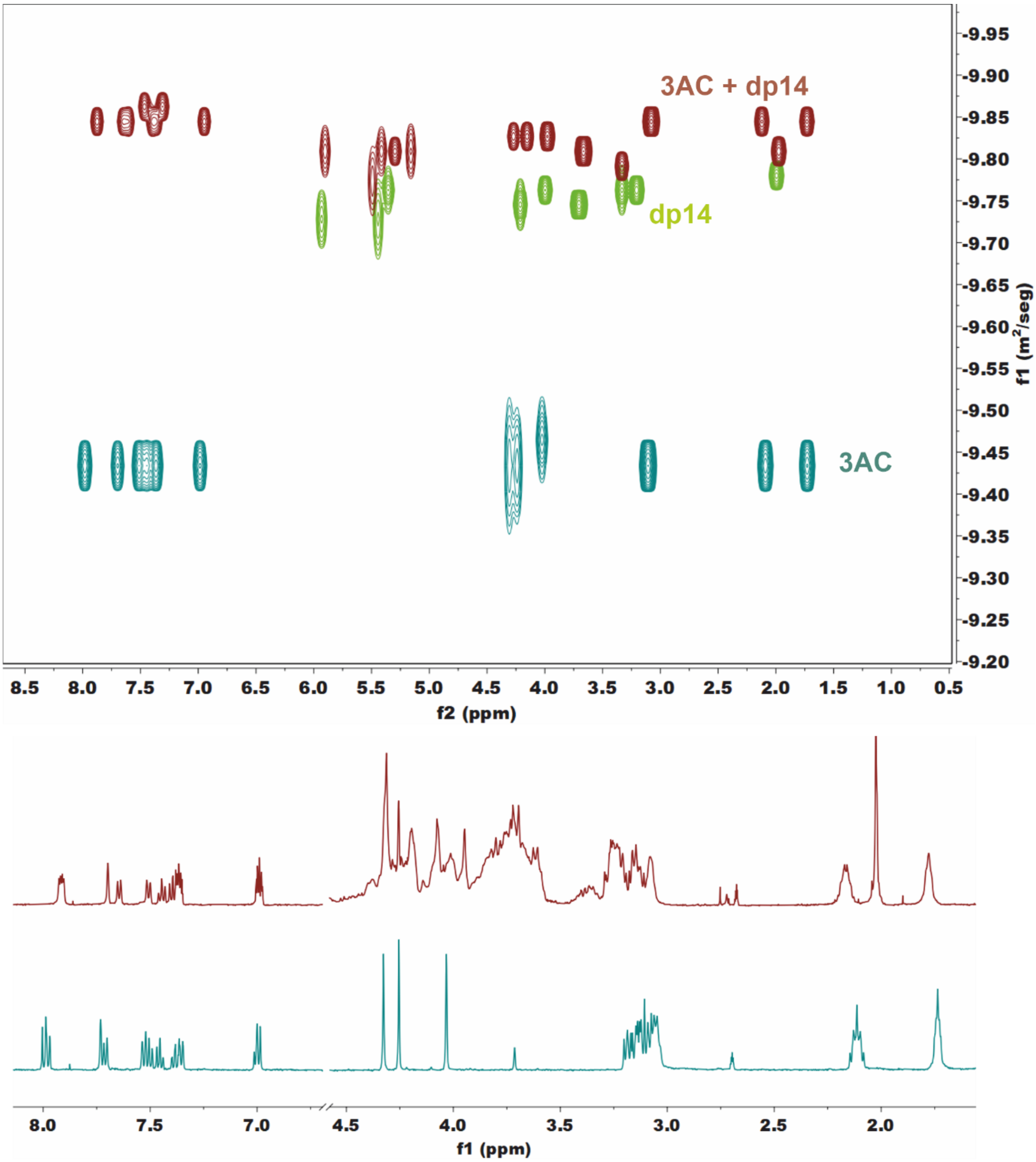

Figure S17. Overlayed 2D DOSY spectra (3AC, dp14 and a mixture of both, color code in the figure, Δ = 140 ms) and 1D ^{1}H NMR spectra (pH* 7, 303K) of 1.5 mM 3AC alone (cyan) and upon the addition of 3.9 mM dp14 (brown). The concentrations were chosen to have [3AC-dp14$_2$] as the main species in solution. The corresponding D values are displayed in Table S7.

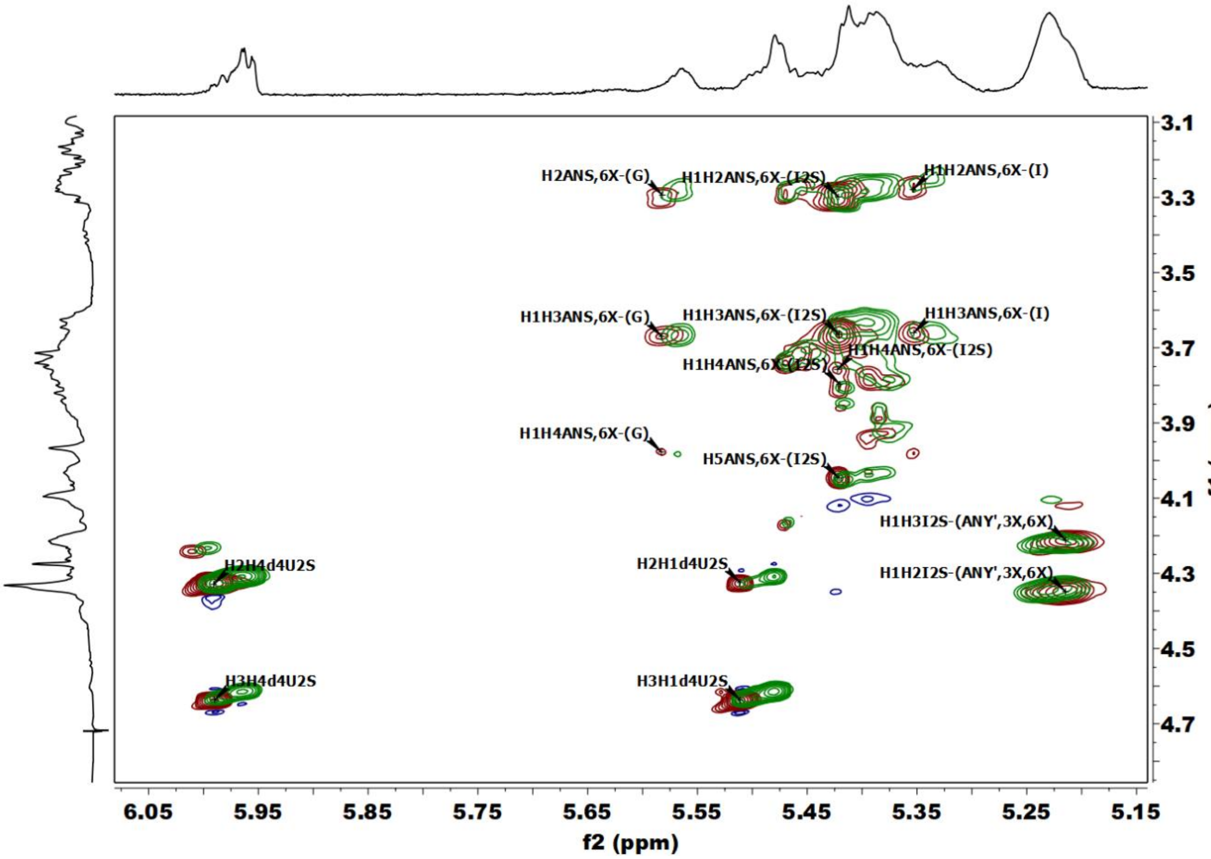

Figure S18. Anomeric region of the overlayed 2D ^{1}H-^{1}H TOCSY spectra of 3.9 mM dp14 (brown) and upon addition of 0.5 mM 3AC (green). The dp14 chemical shift assignment was based on literature data (see Table S5).

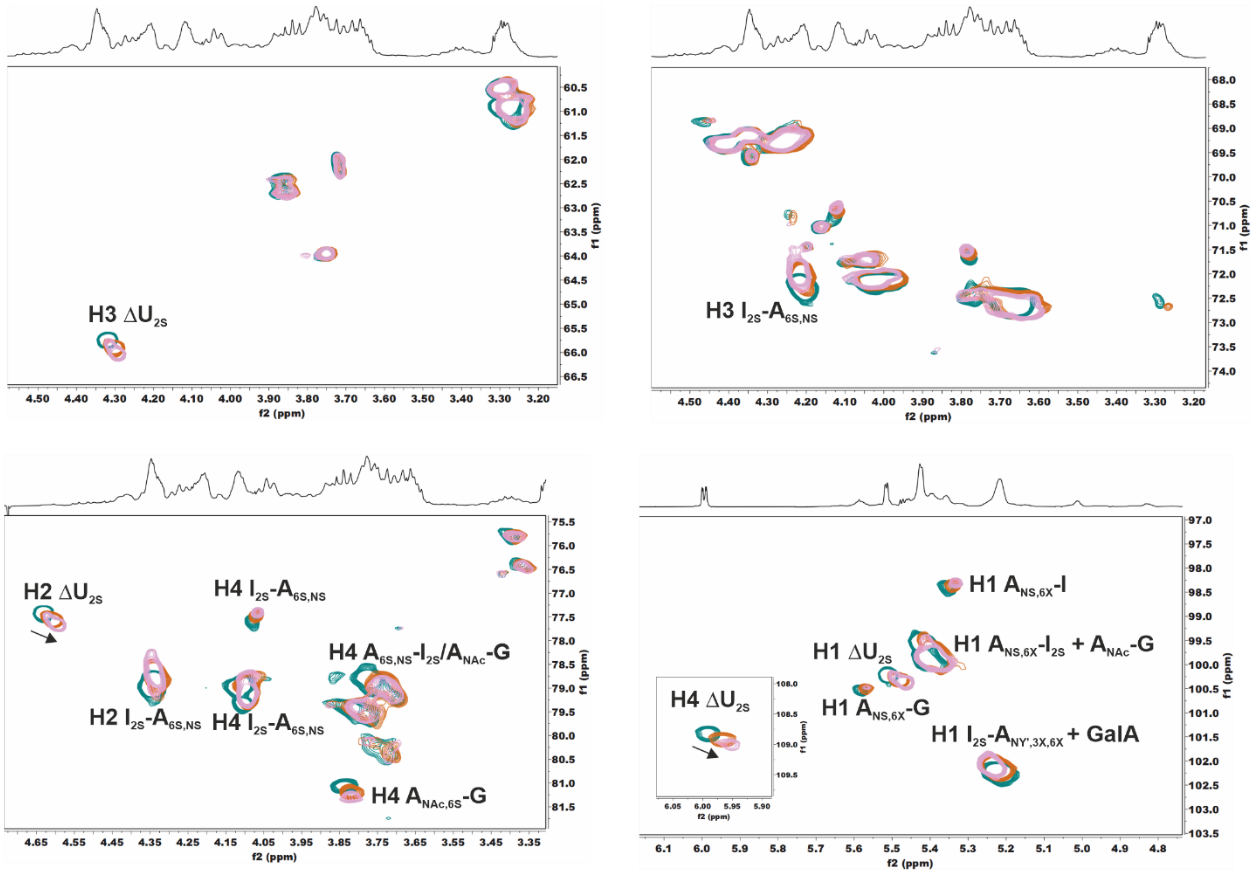

Figure S19. Overlayed 2D ^{1}H-^{13}C HSQC spectra of 3.9 mM dp14 (cyan) titrated with 3AC (0.5 mM, brown; 1.5 mM, pink). The dp14 chemical shift assignment was based on literature data (see Table S5).

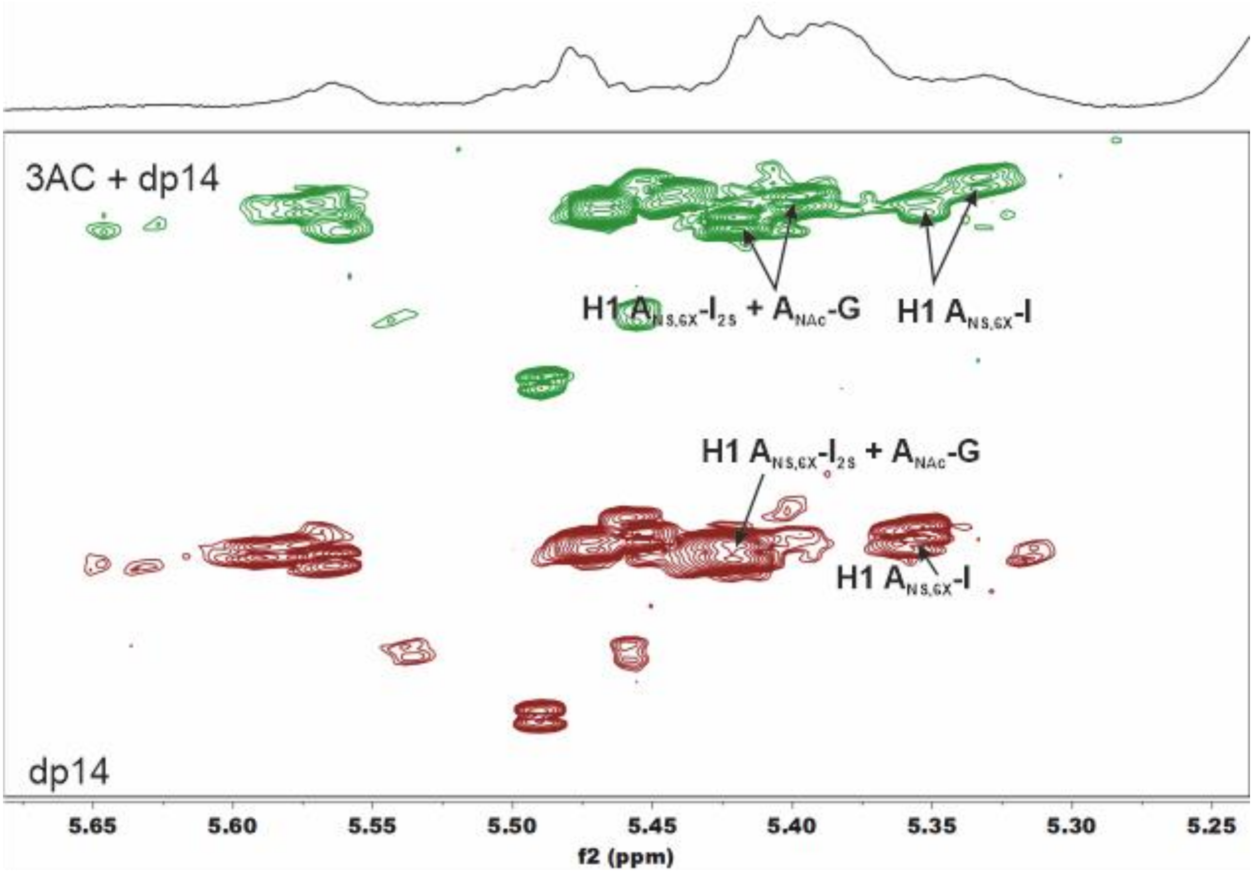

Figure S20. Comparison of the 2D ^1H-^1H COSY NMR spectra of the dp14 anomeric region in the absence (brown) and in the presence (green) of 3AC. Labeled dp14 anomeric resonances split in the presence of 3AC, consistent with slow exchange between different species in solution.

Table S5. dp14 heparin ^{1}H-^{13}C HSQC spectrum assignment.[1]

A = *GlcN*; I = *IdoA*; G = *GlcA*; X = *H or* SO_3^-; Y = H or Ac or SO_3^-; Y' = Ac or SO_3^-; ΔU_{2S}, 2-O-sulfo-4-deoxy-a-L-threo-hex-4-enopyranosyl uronic acid.

	$\nu(F_2)$ ppm	$\nu(F_1)$ ppm
H2 $A_{NAc,6X}$	3.94	56.8
H2 $A_{NS,6X}$	3.30	60.7-60.9
H6 $A_{NY,3X}$	3.88	62.4-62.7
H2 ΔU_{2S}	4.32	65.7
H6 $A_{NY,3X,6S}$	4.35	69.1-69.6
H6 $A_{NS,6S}$-I_{2S}	4.26-4.41	69.3
H5 $A_{6S,NS}$-I_{2S}/A_{NAc}-G	4.05	71.8
H5 $A_{6S,NS}$-I_{2S}/A_{NAc}-G	4.03	72.2
H3 I_{2S}-$A_{6S,NS}$	4.21	72.3
H3 $A_{6S,NS}$-I_{2S}/A_{NAc}-G	3.66	72.6
H2 G-A_{NS}	3.40	75.8
H2 Xylose	3.37	76.4
H2 ΔU_{2S}	4.63	77.4
H4 I_{2S}-$A_{6S,NS}$	4.08	77.5
H4 $A_{6S,NS}$-I_{2S}/A_{NAc}-G	3.77	78.8
H4 I_{2S}-$A_{6S,NS}$	4.10-4.12	79.0-79.3
H2 I_{2S}-$A_{6S,NS}$	4.34	79.0
H3 G-$A_{NS,3S,6S}$	3.70	79.2
H2 G-A_{NAc}	3.82	79.4
H2 I-$A_{6S,NS}$	3.78	79.6
H4 $A_{NAc,6S}$-G	3.84	81.0
H1 $A_{NS,6X}$-αRed + $A_{NH2,6X}$	5.46	94.0
H1 $A_{NS,6X}$-I	5.35	98.4
	5.42	99.5-99.9
H1 ΔU_{2S}	5.51	100.2
H1 $A_{NS,6X}$-G	5.58	100.6
H1 I_{2S}-$A_{NY',3X,6X}$ +GalA	5.21	102.2
H1 Gal-Xyl	4.54	104.3
H1 I-$A_{NY'}$	4.94	104.7
H1 I-$A_{NY',6S}$	5.01	105.0
H1 G-$A_{NS,6X}$	4.60	105.1
H1 G-$A_{NAc,6X}$	4.51	105.3
H1 G-Gal + Gal-Gal	4.68	106.8
H4 ΔU_{2S}	5.99	108.8

Interaction between 3FF and different forms of Hep in solution

Interaction of 3FF with Hep by fluorescence spectroscopy

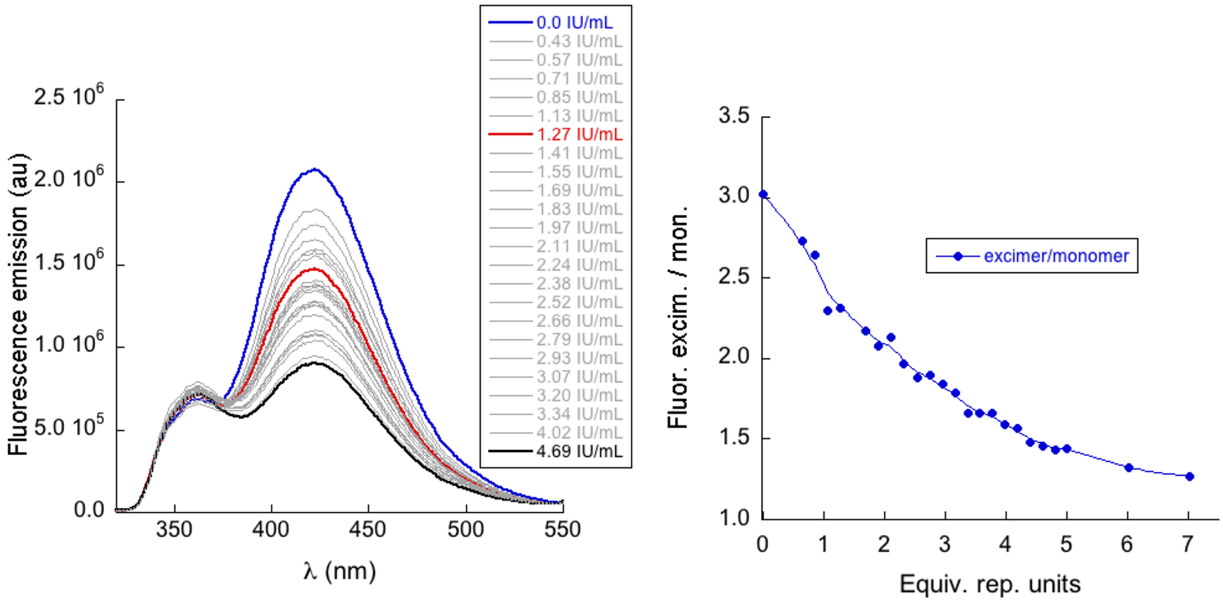

Figure S21. Fluorescence emission spectra (left) of 3FF (10 μM in 1 mM Bis-Tris buffer at pH 7.5) upon the addition of increasing amount of unfractioned heparin (concentration of Hep given in the inset). On the right, we show the plot of the ratio of the excimer/monomer emission bands versus the equivalents of Hep disaccharide repeating units. Since we keep constant the concentration of 3FF and the pH of the medium, the reduction of the relative emission of the excimer implies a strong 3FF-Hep interaction in solution. However, the inherent aggregation of 3FF precludes a quantitative analysis of the titration experiment.

Interaction of 3FF with Hep by DLS

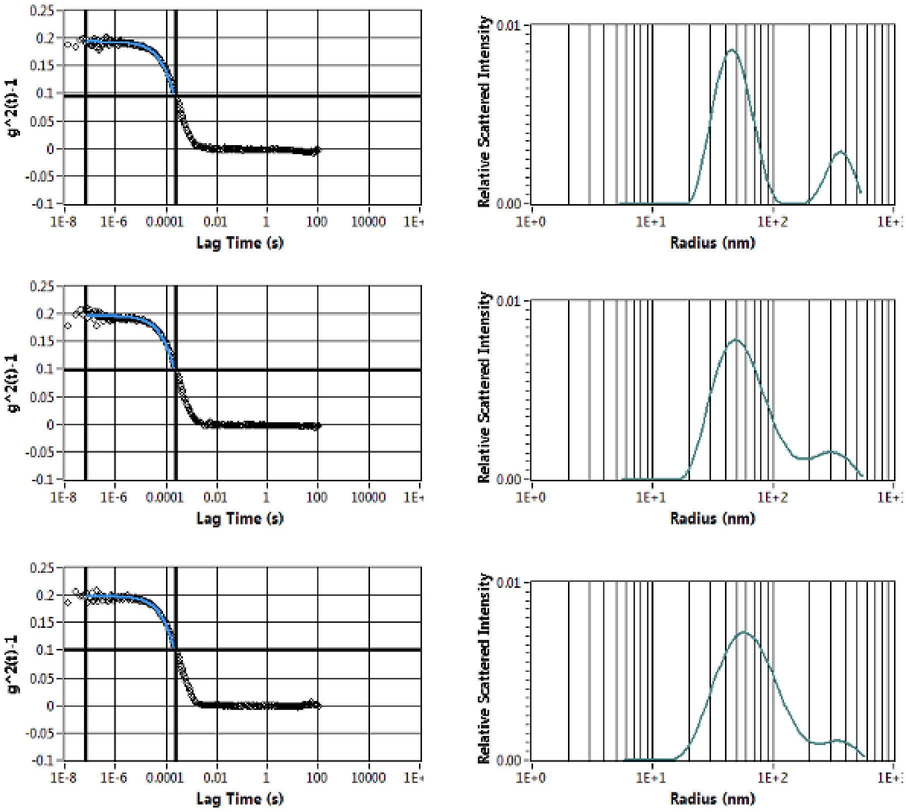

Figure S22: Autocorrelation function (left) and particle size distribution (right) obtained from three DLS experiments performed with samples containing 20 µM of 3FF and 6 mg/L of Hep (10 µM in disaccharide repeating units) dissolved in 3 mM BisTris buffer at pH 7.5.

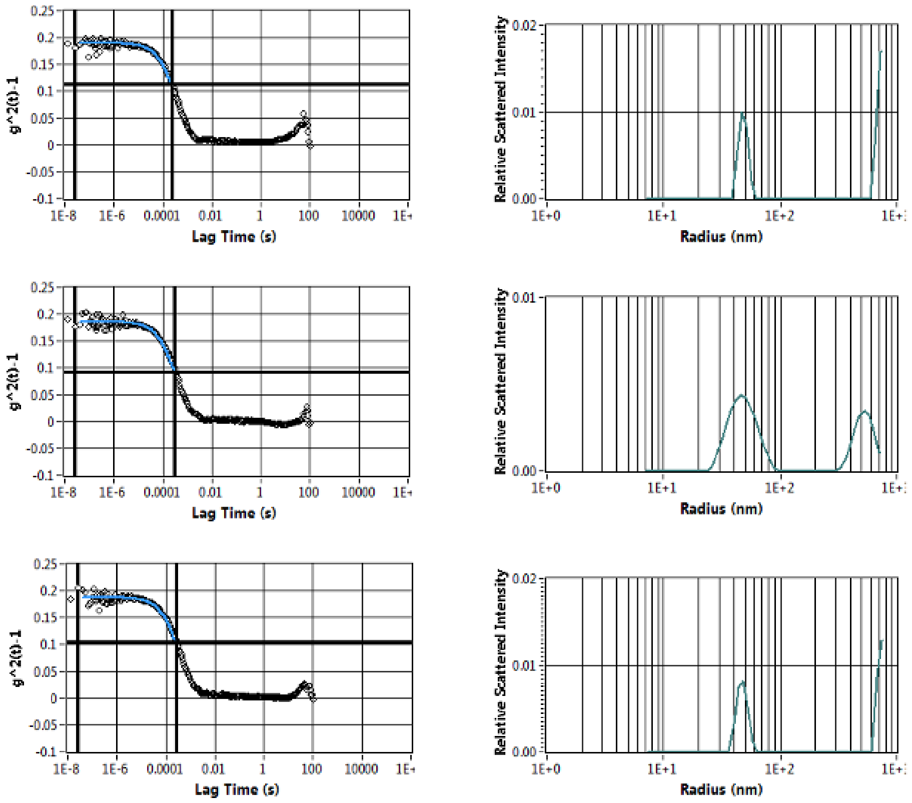

Figure S23. Autocorrelation function (left) and particle size distribution (right) obtained from three DLS experiments with the previous samples but after 48 hours.

Table S6. Hydrodynamic radius and polydispersity index obtained from the analyses of samples in Figures S21-S22.

Sample	Replicate	Rh (nm)	PI
**20 μM 3FF 10 μM UFH (6.7 mg/L)**	**1**	52.8	0.4
	**2**	56.2	0.3
	**3**	56.9	0.3
	**Mean**	**55.1 ± 2.0**	**0.3 ± 0.1**
**After 48 hours**	**1**	73.1	0.4
	**2**	69.3	0.4
	**3**	75.5	0.2
	**Mean**	**72.6 ± 3.1**	**0.3 ± 0.1**

The DLS experiments show that the presence of Hep reduces the apparent size of the 3FF aggregates (compare Tables S3 and S6) suggesting a 3FF-Hep interaction that competes with the 3FF aggregation. Moreover, the apparent size of the species changes with time, implying a dynamic complex behavior of the supramolecular species in solution.

Interaction of 3FF with Hep by NMR

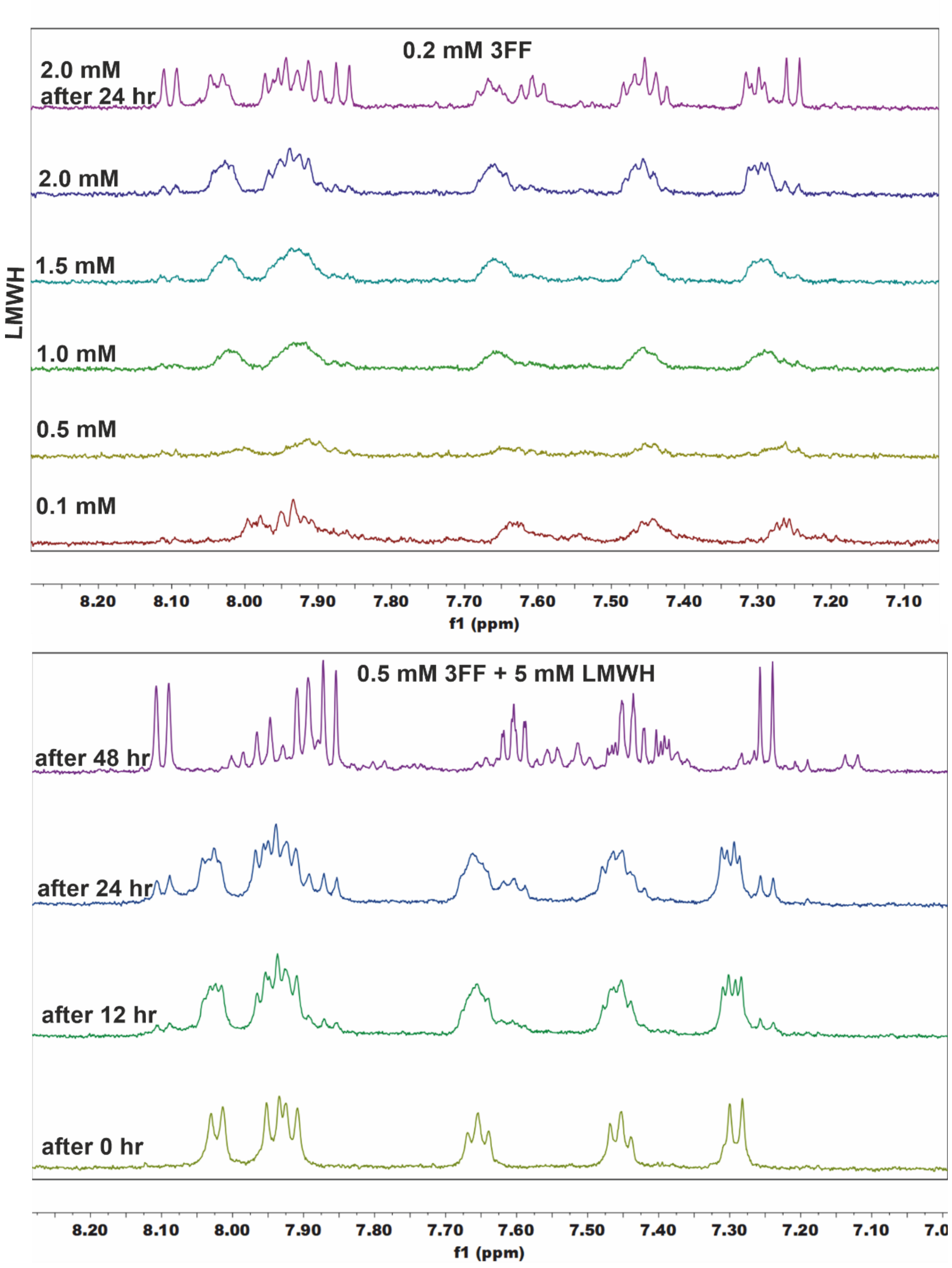

Figure S24. (Top) 1D ^{1}H NMR spectra titration of 0.2 mM 3FF with LMWH (0.1 to 2.0 mM) at neutral pH and (bottom) time evolution (0, 12, 24 and 48 hours) of 0.5 mM 3FF 1D ^{1}H NMR spectrum after addition of 5 mM LMWH (5 mM Tris/50 mM NaCl, 298K, pH* 7.2).

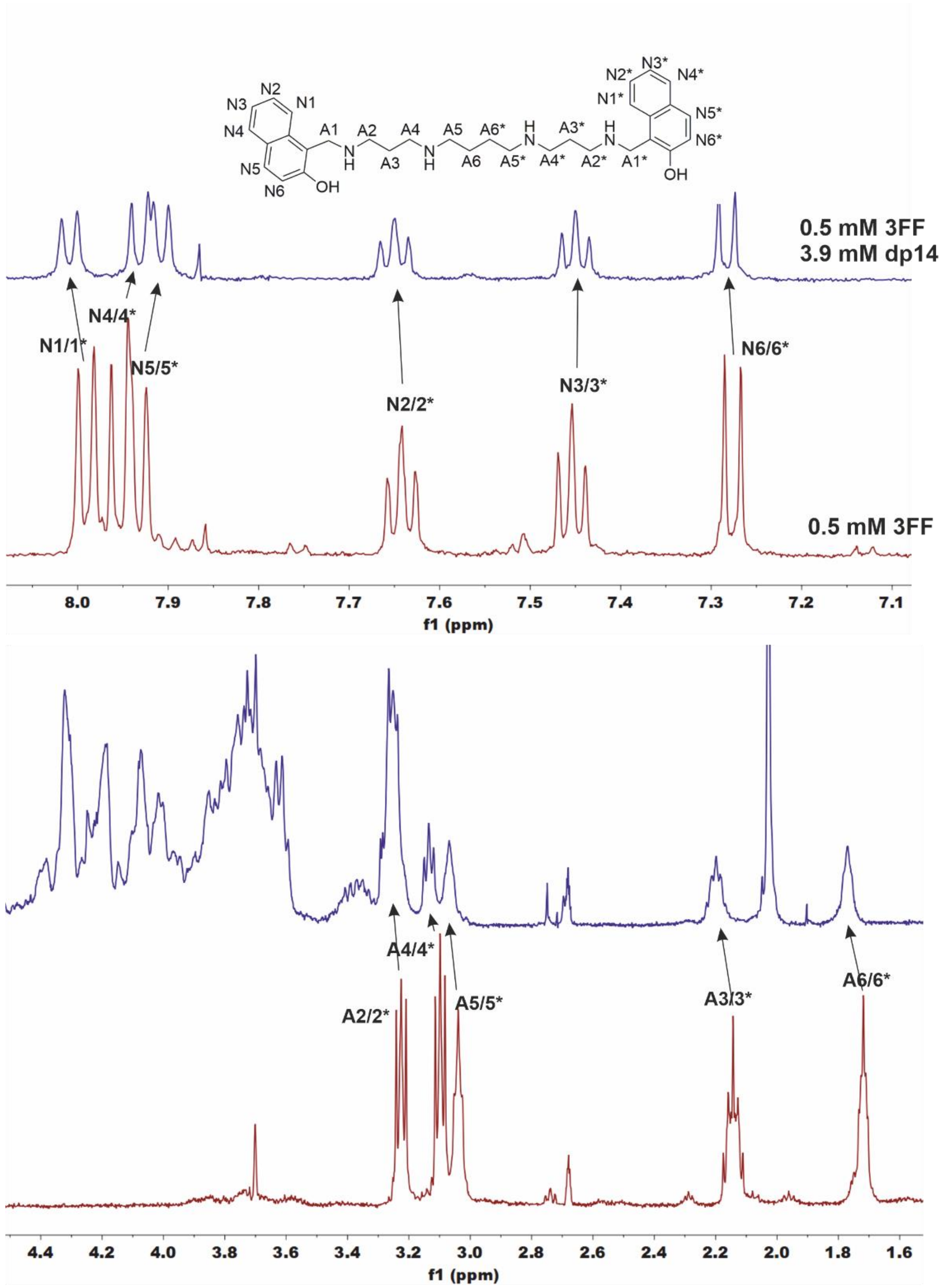

Figure S25. 1D ^{1}H NMR (pH* 7.0, 303K, 5 mM Tris-d11, 50 mM NaCl) spectra of 0.5 mM 3FF (brown) and upon addition of 3.9 mM dp14 heparin (blue).

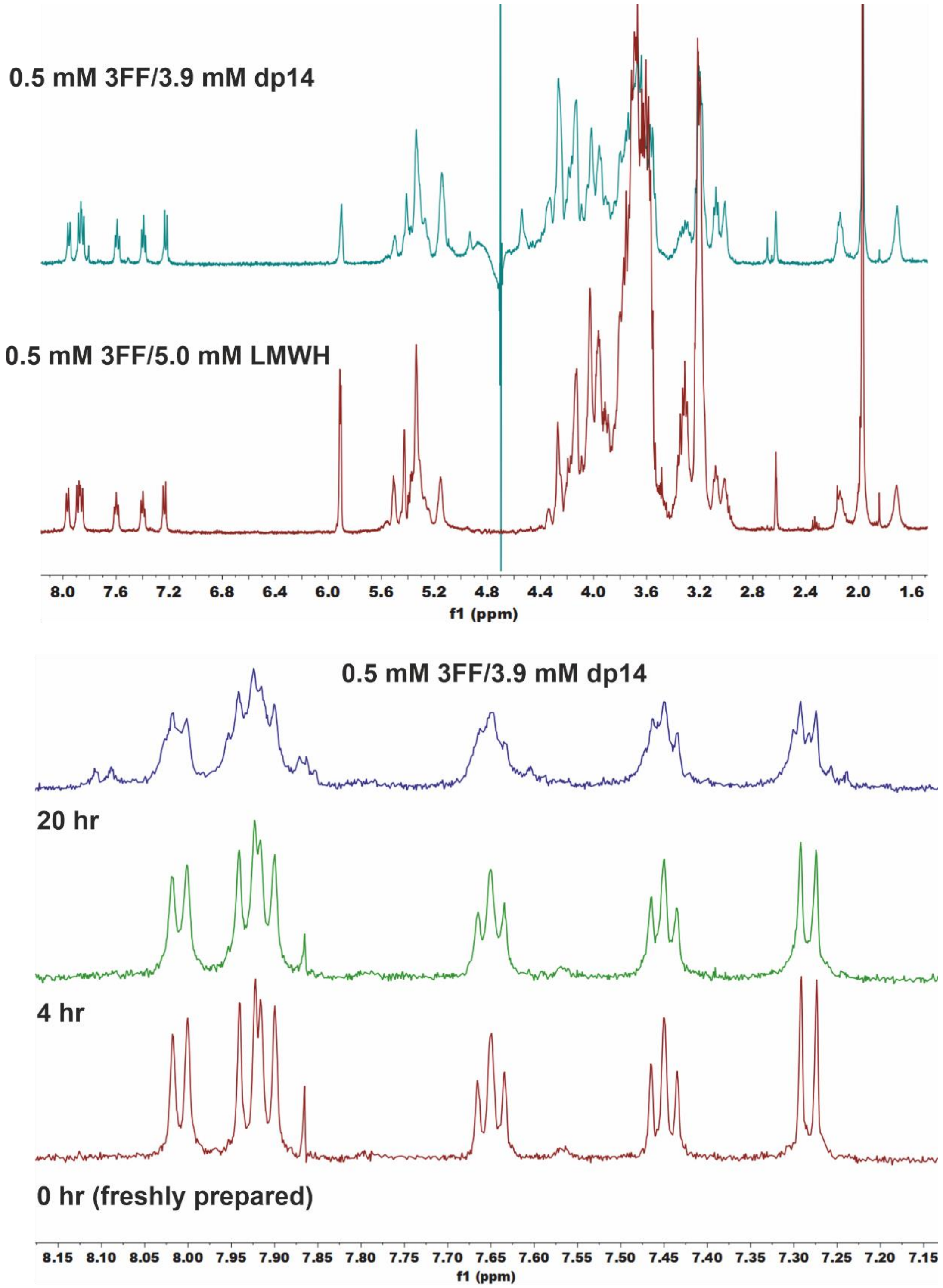

Figure S26. (Top) Comparison 1D ^{1}H NMR spectra of 0.5 mM 3FF (pH* 7.2, 298K, 5 mM Tris-d11, 50 mM NaCl) with LMWH or dp14 heparin oligosaccharides. (Bottom) Time evolution of 0.5 mM 3FF/3.9 mM dp14 1D ^{1}H NMR spectra (pH* 7.2, 303K, 5 mM Tris-d11, 50 mM NaCl).

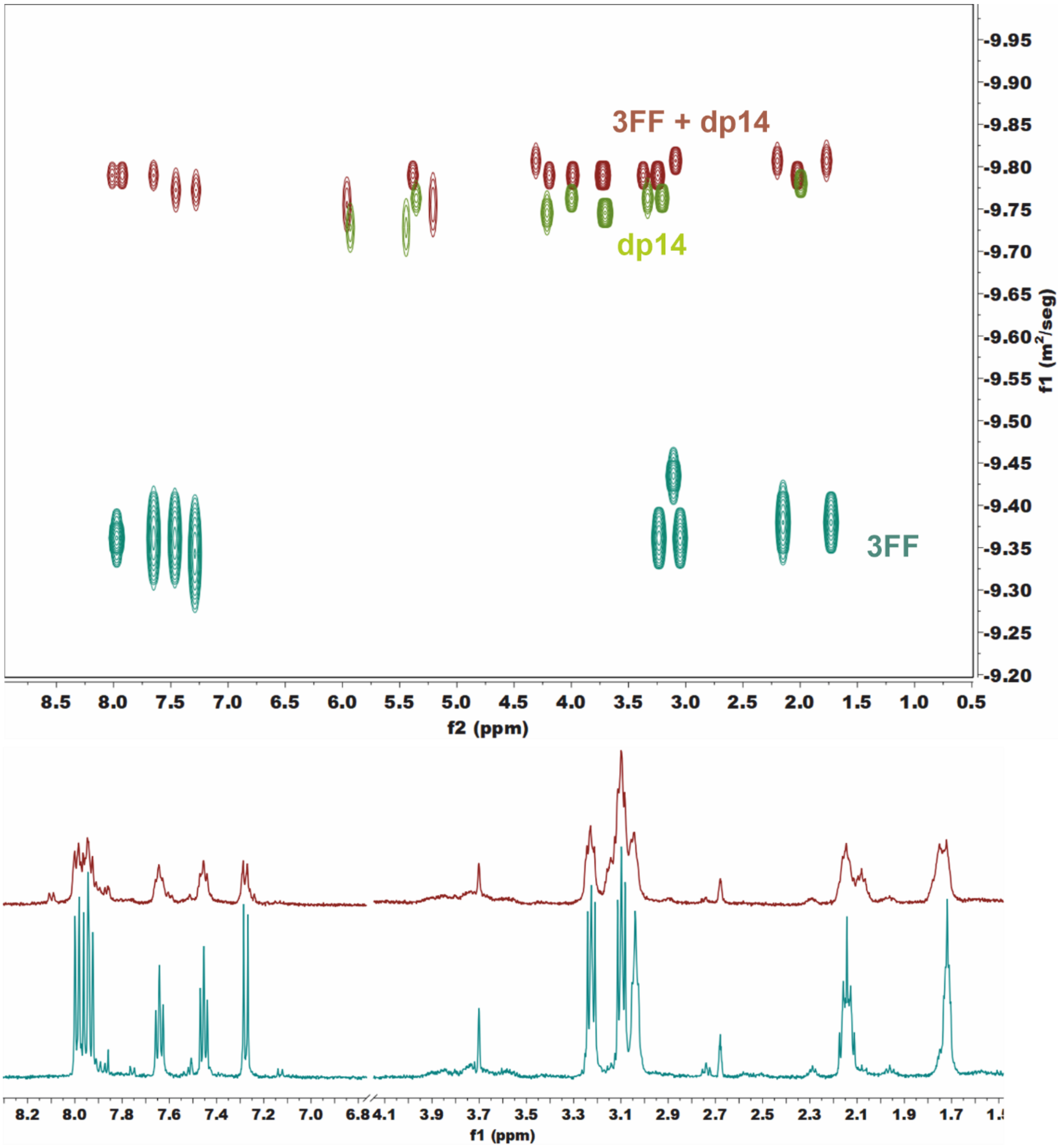

Figure S27. (Top) Superimposed 2D DOSY (Δ = 140 ms) NMR spectra of 0.5 mM 3FF, 3.9 mM dp14 and a mixture of both (color code given in the figure). (Bottom) 1D ^{1}H NMR spectra of 0.5 mM 3FF (cyan) and upon addition of 3.9 mM dp14 (brown). All experiments performed at pH* 7 and 303K. The corresponding D values are displayed in Table S7.

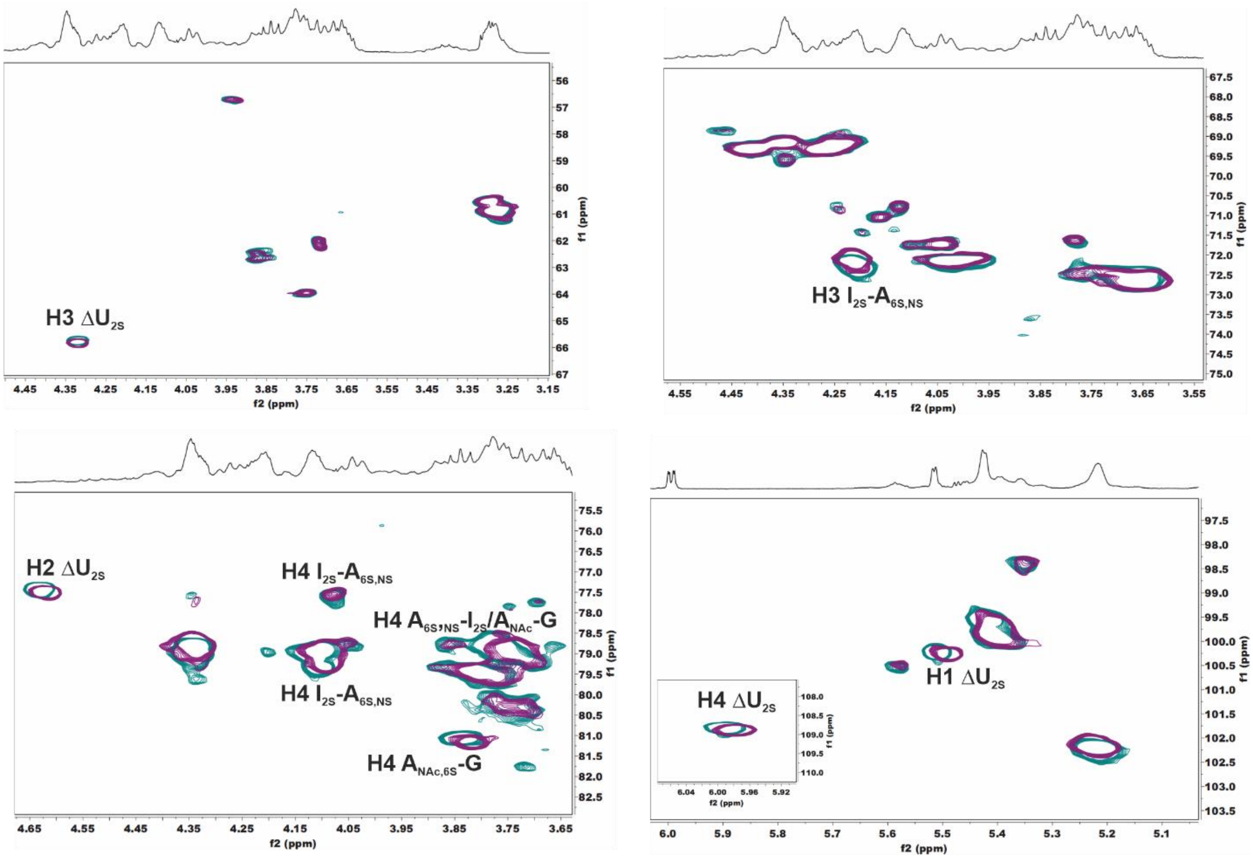

Figure S28. Superimposed 2D ^{1}H-^{13}C HSQC NMR spectra of 3.9 mM dp14 heparin (cyan) and upon addition of 0.5 mM 3FF (purple), dp14 chemical shift assignment based on literature data (see Table S5).

Table S7. DOSY measured self-diffusion rates (D, $m^2 \cdot s^{-1}$) of the signals for different samples.

Sample	ppm	Resonance	Temp	D ($x10^{-10}$ $m^2 \cdot s^{-1}$)
3FF (0.5 mM) pH 7.2 6 hr	1.69	$(CH_2)_2$	298K	3.650 ± 0.005
	2.12	CH_2	298K	3.690 ± 0.006
	3.10	CH_2-N	298K	3.700 ± 0.003
	7.97	Aromatic	298K	4.000 ± 0.006
	3.75	Dioxane	298K	9.180± 0.001
3FF (0.5 mM) pH 7.2 20 h	1.74	$(CH_2)_2$	298K	3.720 ± 0.004
	2.10	CH_2	298K	3.740 ± 0.005
	3.10	CH_2-N	298K	3.890 ± 0.002
	7.93	Aromatic	298K	4.43 ± 0.01
	8.10	Aromatic	298K	4.87 ± 0.05
	3.75	Dioxane	298K	9.110 ± 0.001
3FF (0.1 mM) pH 7.2 6 h	3.12	CH_2-N	298K	5.35± 0.16
		Dioxane	298K	9.430 ± 0.005
3FF (0.5 mM) pH 4.0 6 h	1.76	$(CH_2)_2$	298K	3.020 ± 0.001
	2.17	CH_2	298K	3.080 ± 0.001
	3.10	CH_2-N	298K	3.020 ± 0.001
	7.98	Aromatic	298K	3.090 ± 0.001
	3.75	Dioxane	298K	9.070 ± 0.001
3FF (0.5 mM) pH 4.0 16 h	1.76	$(CH_2)_2$	298K	3.130 ± 0.002
	2.17	CH_2	298K	3.140 ± 0.002
	3.10	CH_2-N	298K	3.120 ± 0.001
	7.98	Aromatic	298K	3.130 ± 0.001
	8.10	Aromatic	298K	4.34 ± 0.02
	3.75	Dioxane	298K	9.130 ± 0.001
3FF (0.5 mM) pH 7 *Fresh*	1.66	$(CH_2)_2$	303K	3.94 ± 0.01
	2.09	CH_2	303K	3.97 ± 0.02
	2.98	CH_2-N	303K	4.13 ± 0.01
	7.91	Aromatic	303K	4.23 ± 0.01
dp14 (0.7 mM)	2.00	Acetyl	303K	1.59 ± 0.01
	3.70	H3/H4 sugars	303K	1.700 ± 0.005
	5.92	H4 ΔU_{2S}	303K	1.78 ± 0.05
3FF (0.5 mM) dp14 (3.9 mM) pH 7 *Fresh*	1.71	$(CH_2)_2$	303K	1.50 ± 0.01
	2.14	CH_2	303K	1.50 ± 0.01
	7.86	Aromatic	303K	1.67 ± 0.01
	7.95	Aromatic	303K	1.590 ± 0.001
3AC (1.5 mM) pH 7 *Fresh*	1.68	$(CH_2)_2$	303K	3.330 ± 0.003
	2.04	CH_2	303K	3.330 ± 0.003
	3.04	CH_2-N	303K	3.320 ± 0.002
	7.92	Aromatic	303K	3.330 ± 0.005
3AC (1.5 mM) dp14 (3.9 mM) pH 7 *Fresh*	1.72	$(CH_2)_2$	303K	1.350 ± 0.003
	2.11	CH_2	303K	1.360 ± 0.004
	3.07	CH_2-N	303K	1.400 ± 0.001
	7.86	Aromatic	303K	1.360 ± 0.005

Interaction of some selected binders with Hep by fluorescence spectroscopy

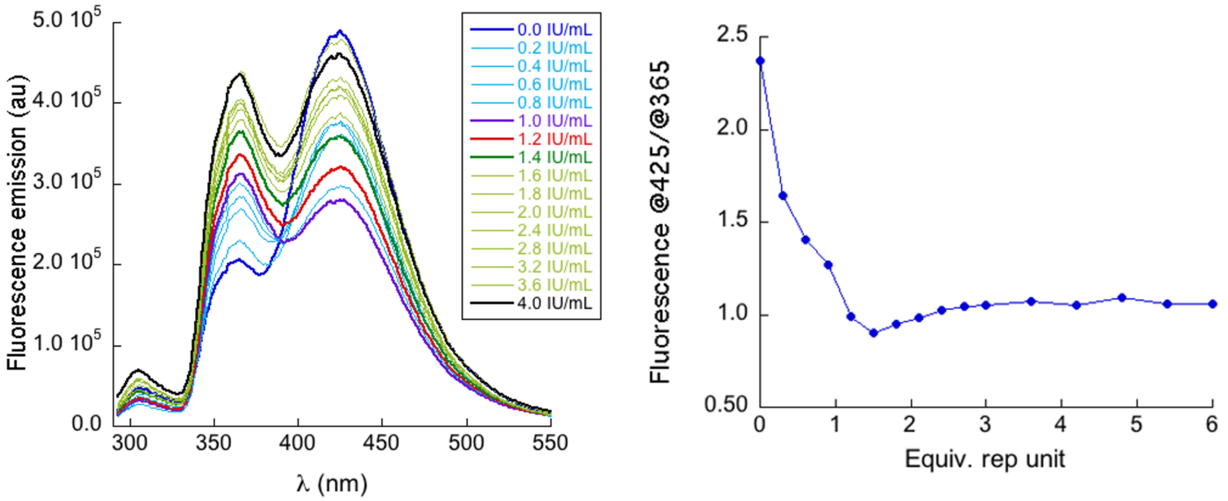

Figure S29. Fluorescence emission spectra (left) of 3AF (10 μM in 1 mM Bis-Tris buffer at pH 7.5) upon the addition of increasing amount of unfractioned heparin (concentration of Hep given in the inset). On the right, we show the plot of the excimer/monomer emission bands ratio versus the equivalents of heparin disaccharide repeating units. The titration suggests a complex binding mechanism implicating the 3AF aggregation in competition with the Hep interaction.

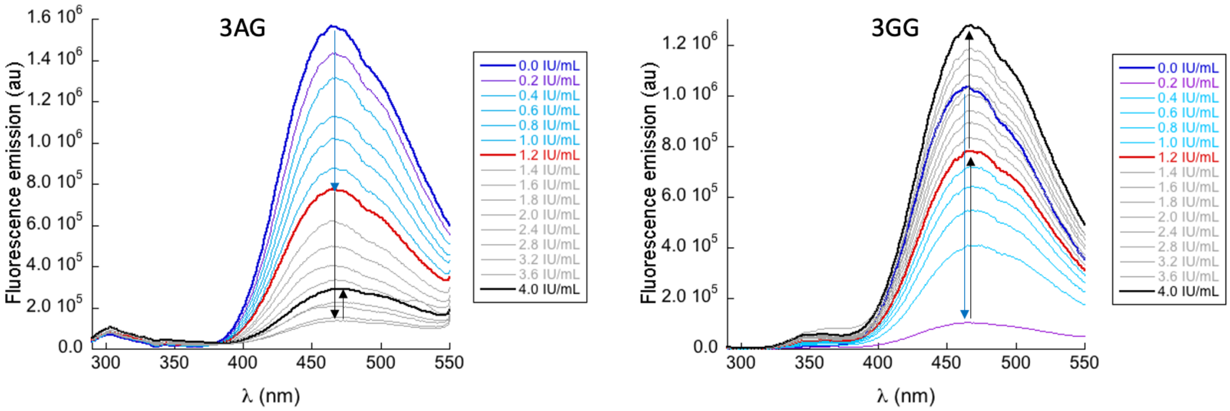

Figure S30. Fluorescence emission spectra of either 3AG (left) or 3GG (right), each at 10 μM in 1 mM Bis-Tris buffer at pH 7.5, upon the addition of increasing amount of unfractioned heparin (concentration of Hep given in the insets). The arrows indicate the evolution of the spectra upon titration. Both experiments support the Hep binding, although a complex process is evident most likely due to the co-existence of different species in solution including the aggregation of the binder. The higher intensity of the excimer band throughout the titration experiments suggests that binding is dominated by aggregation for these two binders.

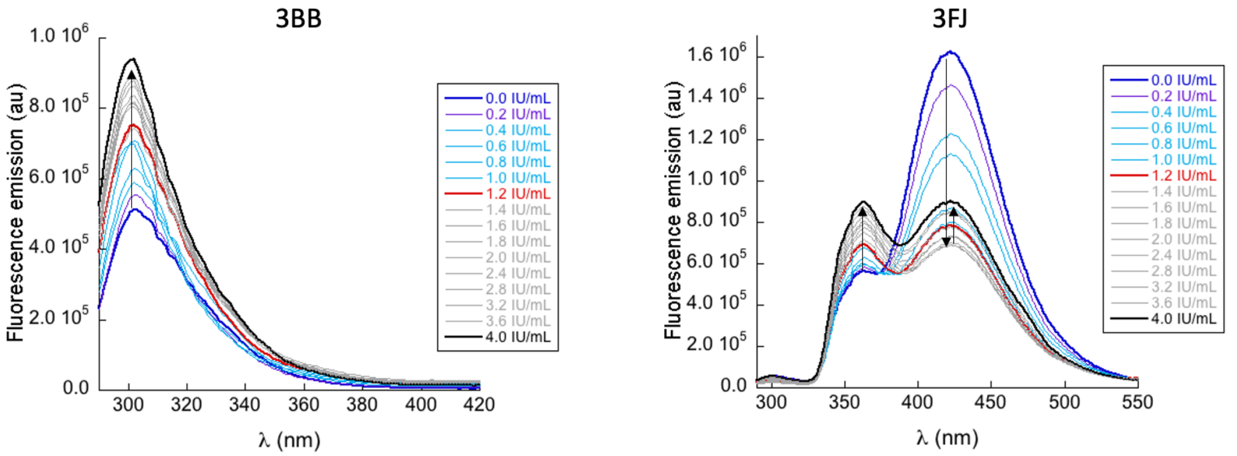

Figure S31. Fluorescence emission spectra of either 3BB (left) or 3FJ (right), each at 10 μM in 1 mM Bis-Tris buffer at pH 7.5, upon the addition of increasing amount of unfractioned heparin (concentration of Hep given in the insets). The arrows indicate the evolution of the spectra upon titration. Both experiments support the Hep binding, although a complex binding process is evident with 3FJ, most likely due to the co-existence of different species in solution including the aggregation of the binder.

In vitro enzymatic assays

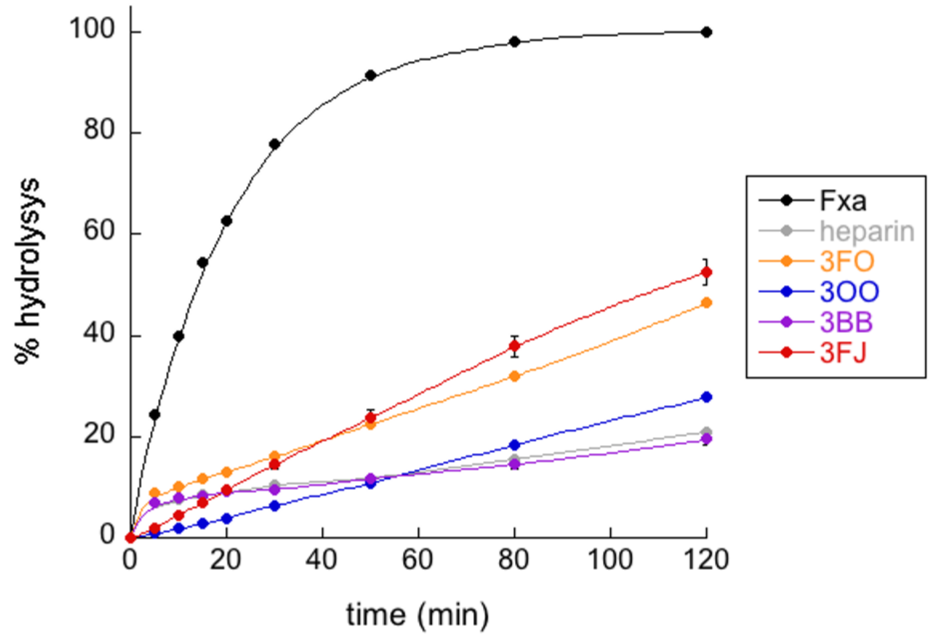

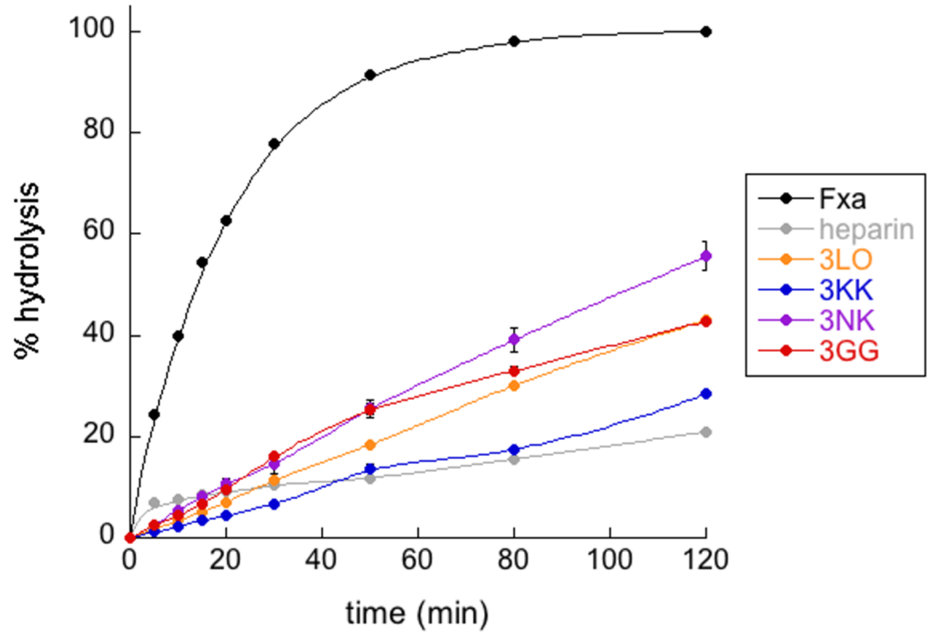

Figure S32. Plot of the time evolution of the hydrolysis of the peptide substrate (485 μM) under different conditions: all traces (0.085 μg/mL of FXa and 0.02 IU/mL of AT_{III}), gray and colored traces (0.1 IU/mL of Hep), colored traces (1 μM of each ligand).

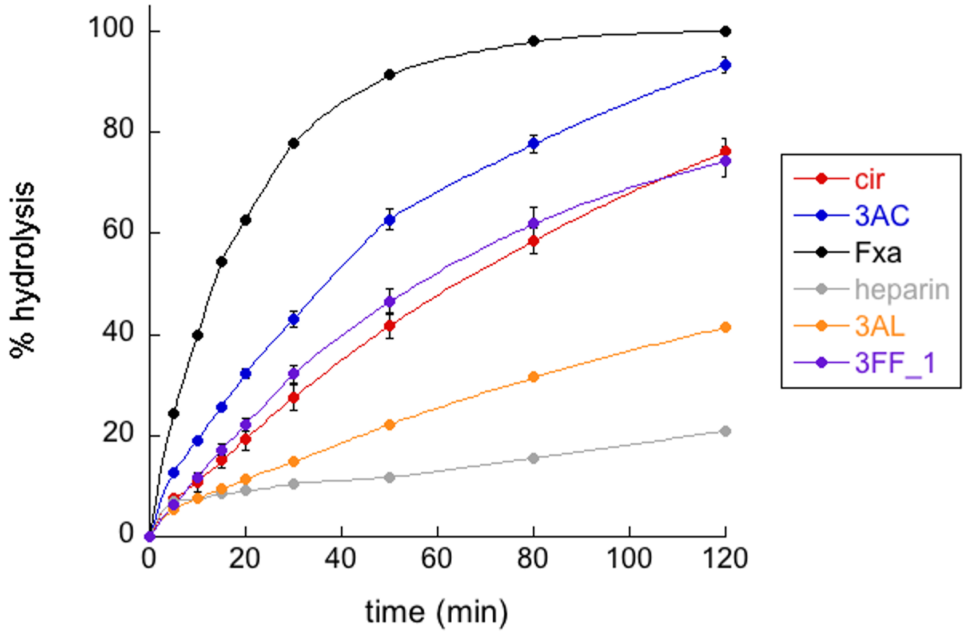

Figure S33. Comparison of selected ligands with the drug *ciraparantag* (cir): Plot of the time evolution of the hydrolysis of the peptide substrate (485 μM) under different conditions: all traces (0.085 μg/mL of FXa and 0.02 IU/mL of AT_{III}), gray and colored traces (0.1 IU/mL of Hep), colored traces (1 μM of each ligand).

Molecular modeling studies

The interaction between heparin and selected ligands, namely 3AC, 3AL and 3FF, was studied by molecular dynamics. Comercial heparin is a polydisperse mixture of fragments of different length, constituted by the repetition of dimers of L-iduronic acid and D-glucosamine which are sulfated to a varying degree. In addition, it has been shown that two conformers of the iduronic acid residue are present in aqueous solutions of heparin-like saccharides, namely $^{1}C_{4}$ and $^{2}S_{O}$.[2] Thus, although a systematic study on the influence of the heparin sequence and conformation on ligand binding goes beyond the scope of our work, we prepared four different heparin models formed by eight dimers that differed on their sequence, namely [D-GlcNS[6S]-α(1-4)-L-IdoA[2S]-α(1-4)]$_8$ and [L-IdoA[2S]-α(1-4)-DGlcNS[6S]-α(1-4)]$_8$. In addition, despite the pyranose rings of the eight DGlcNS[6S] were always in the $^{4}C_{1}$ conformation, two different initial conformations, $^{1}C_{4}$ and $^{2}S_{O}$, for the eight IdoA[2S] pyranose rings were considered. Thus, 4 different models were generated that were named: ($^{4}C_{1}$)Glc-($^{1}C_{4}$)Ido, ($^{1}C_{4}$)Ido-($^{4}C_{1}$)Glc, ($^{4}C_{1}$)Glc-($^{2}S_{O}$)Ido, and ($^{2}S_{O}$)Ido-($^{4}C_{1}$)Glc.

Simulations were performed in which six molecules of ligand and one molecule of Hep dp16 in explicit solvent were allowed to interact. The results showed small differences between ligands and between heparin models (Figure S34), indicating that essentially all ligand molecules could bind to the heparin molecules during most of the simulations, establishing around 5 H-bond interactions per ligand molecule and with most of the atoms of every ligand molecule within close distance of the heparin molecule. In contrast, in the competence simulations between 3AC/3FF and 3AC/3AL a clear decrease in the number of H-bonds and ligand atoms within short distance from heparin is observed for the three ligands (Figures S35 and S36). However, the decrease is in most cases larger for 3FF and 3AL than for 3AC, suggesting that the 3AC molecules have a higher affinity for heparin, in agreement with our experimental results. The differences between binding to the different Hep models are small and difficult to analyze without performing more simulations, thus no conclusions can be drawn in this respect.

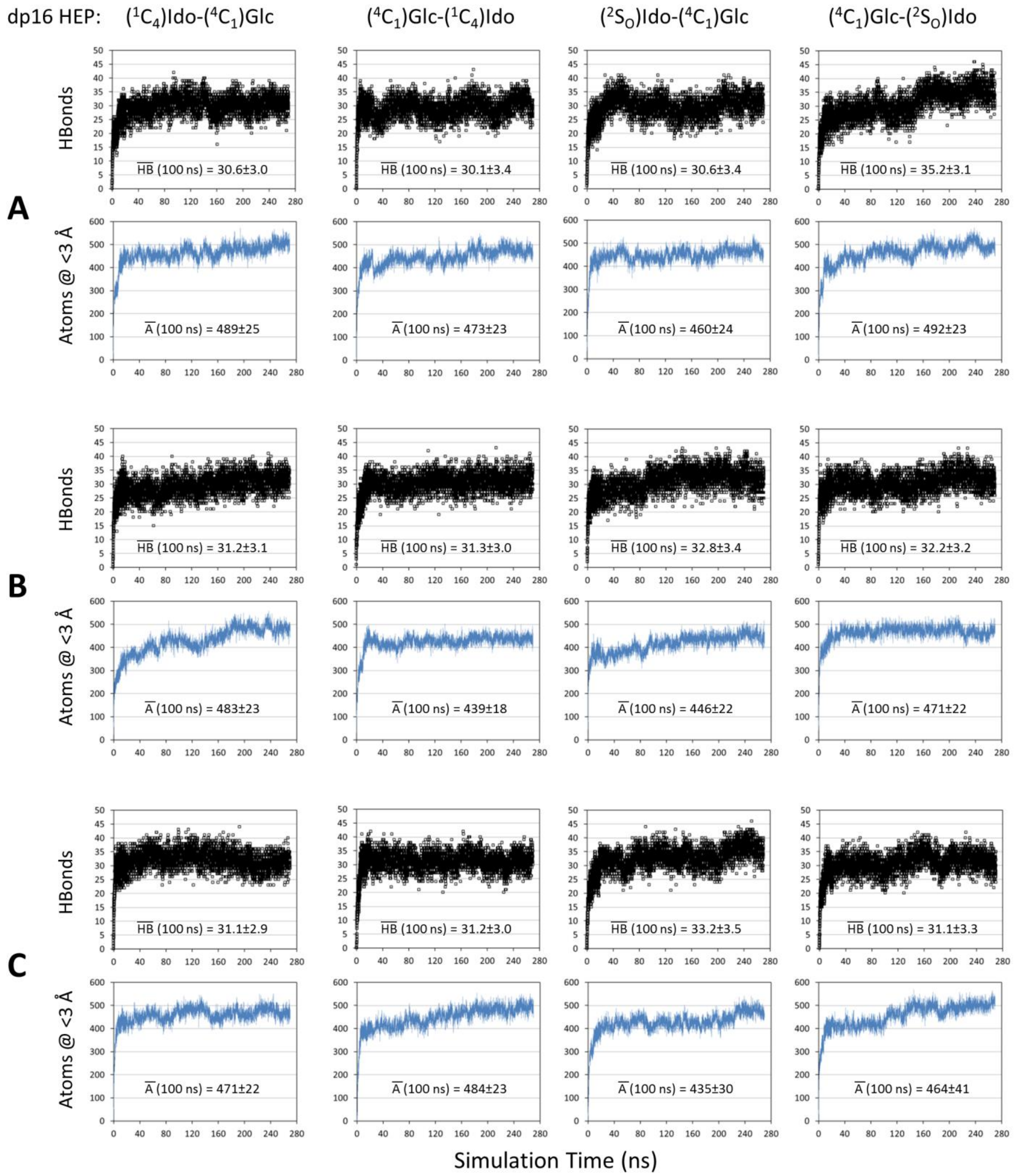

Figure S34. Results from simulations of one molecule of dp16 Hep plus 6 molecules of (A) 3AC, (B) 3FF or (C) 3AL. Graphs represent the total number of hydrogen bonds between ligands and Hep and the total number of ligand atoms within a distance of 3 Å from Hep vs. simulation time. The equilibration (20 ns) plus production (250 ns) phases are shown. Average values for the last 100 ns are displayed on each graph.

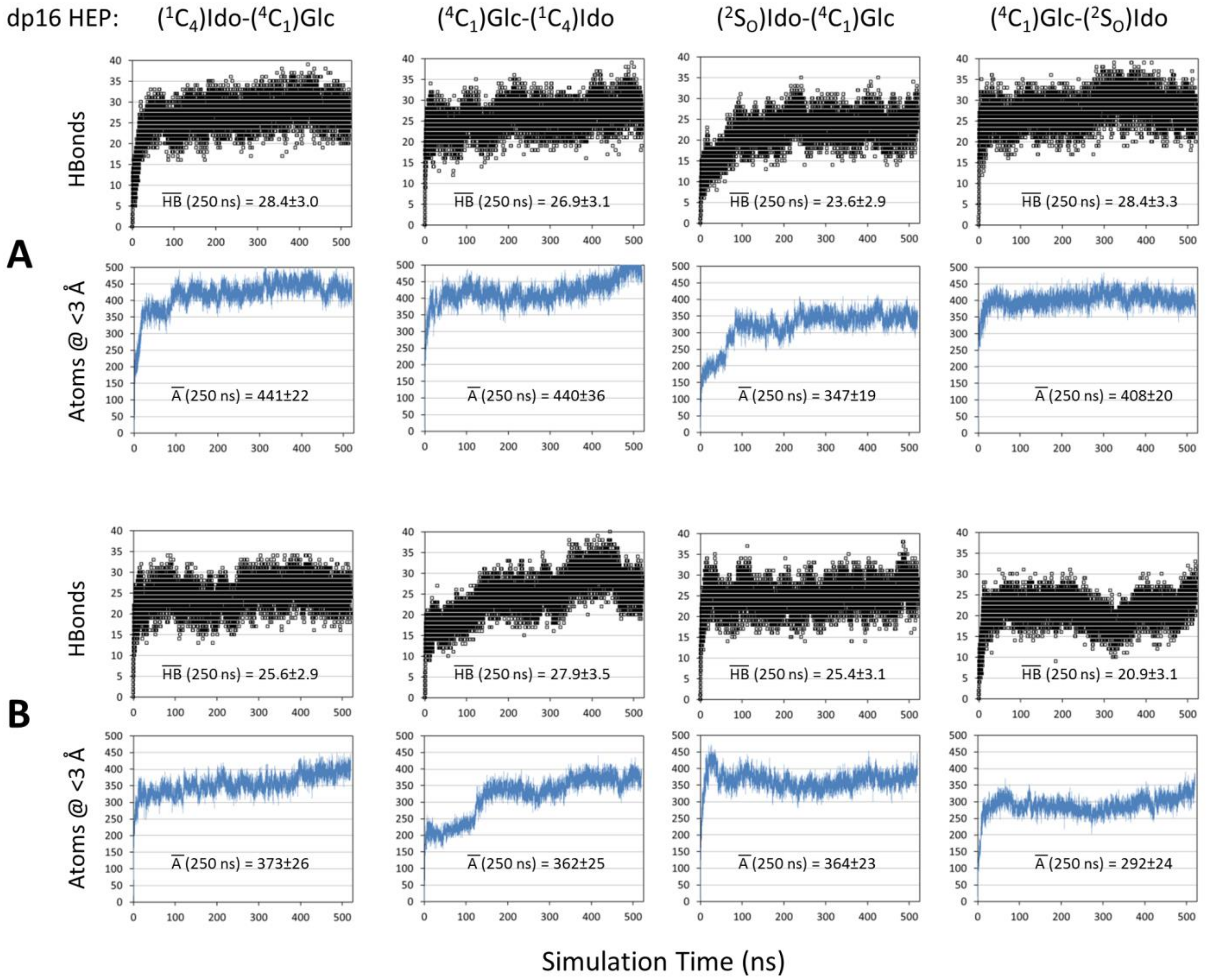

Figure S35. Results from competence simulations between (A) 3AC and (B) 3FF for binding to dp16 Hep. Graphs represent the total number of hydrogen bonds between ligands and Hep and the total number of ligand atoms within a distance of 3 Å from Hep vs. simulation time. The equilibration (20 ns) plus production (500 ns) phases are shown. Average values for the last 250 ns are displayed on each graph.

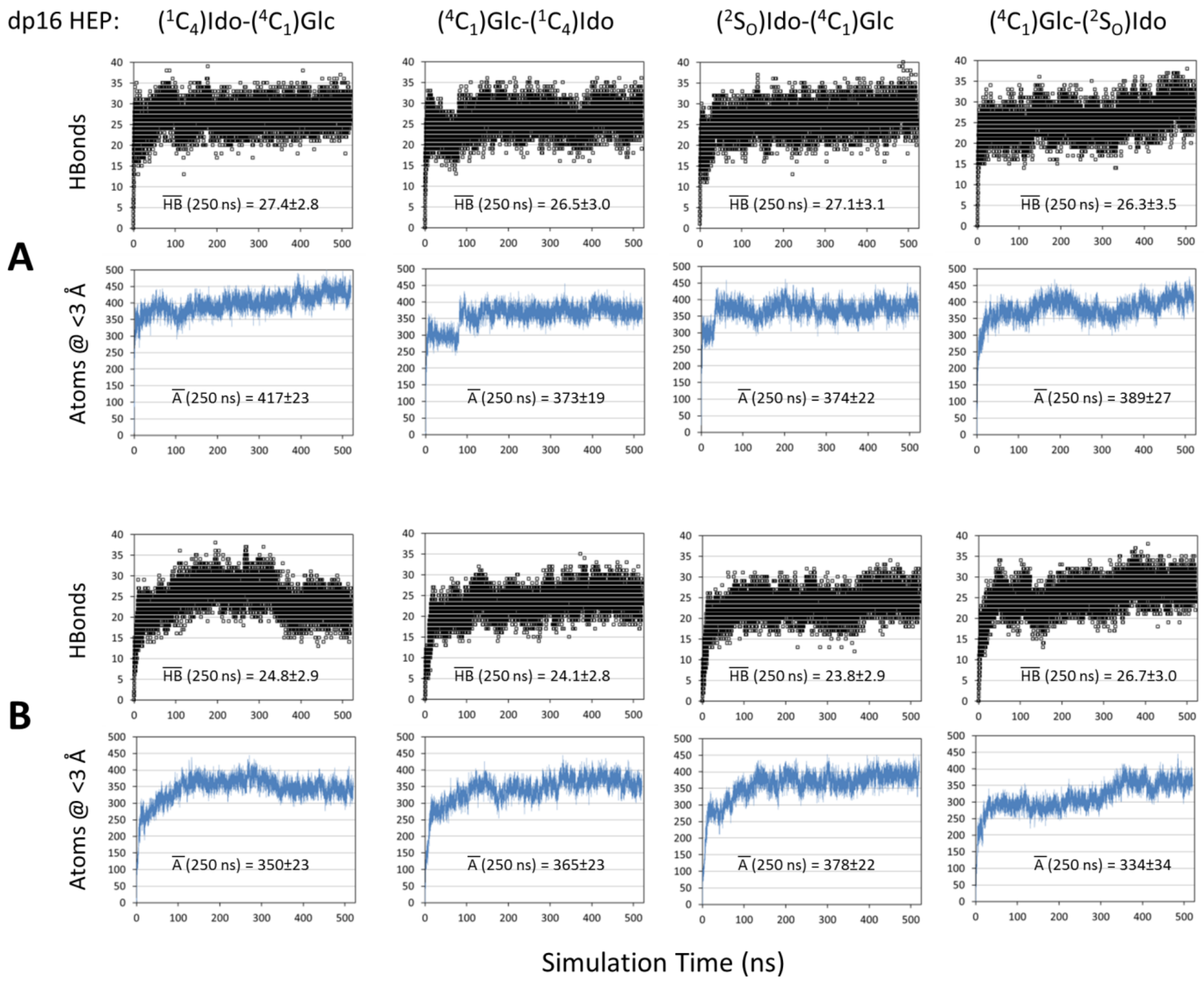

Figure S36. Results from competence simulations between (A) 3AC and (B) 3AL for binding to dp16 Hep. Graphs represent the total number of hydrogen bonds between ligands and Hep and the total number of ligand atoms within a distance of 3 Å from Hep vs. simulation time. The equilibration (20 ns) plus production (500 ns) phases are shown. Average values for the last 250 ns are displayed on each graph.

Video_S1. Representative simulation of the competence between 6 molecules of 3AC (green C-atoms) and 6 molecules of 3FF (cyan C-atoms) for binding to a dp16 Hep model (($^{1}C_{4}$)Ido-($^{4}C_{1}$)Glc, orange C-atoms). Only the initial 170 ns of the simulation are shown.

Video_S2. Representative simulation of the competence between 6 molecules of 3AC (green C-atoms) and 6 molecules of 3AL (pink C-atoms) for binding to a dp16 Hep model (($^{1}C_{4}$)Ido-($^{4}C_{1}$)Glc, orange C-atoms). Only the initial 170 ns of the simulation are shown.

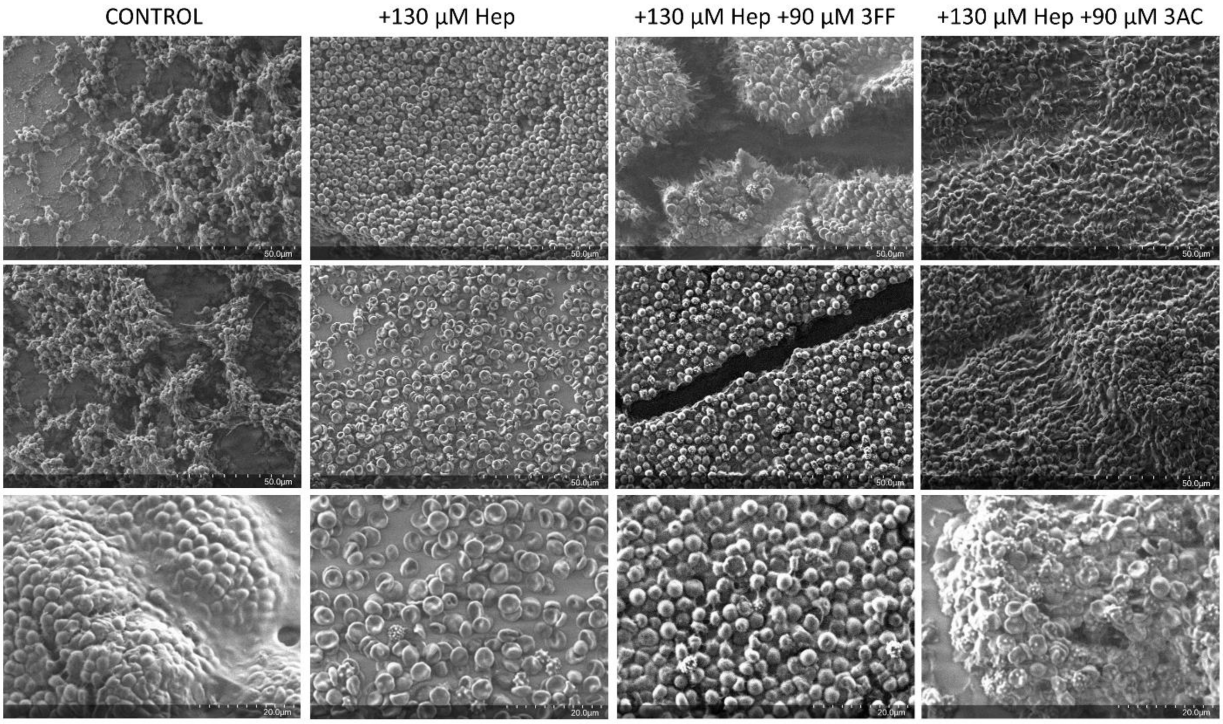

Figure S37. Scanning Electron Microscopy images of blood samples from mice, at different conditions: control sample (first row), samples containing heparin (second row) and heparin plus either 3FF (third row) or 3AC (fourth row). Different regions and magnifications are shown with scale bars at each bottom right corner.

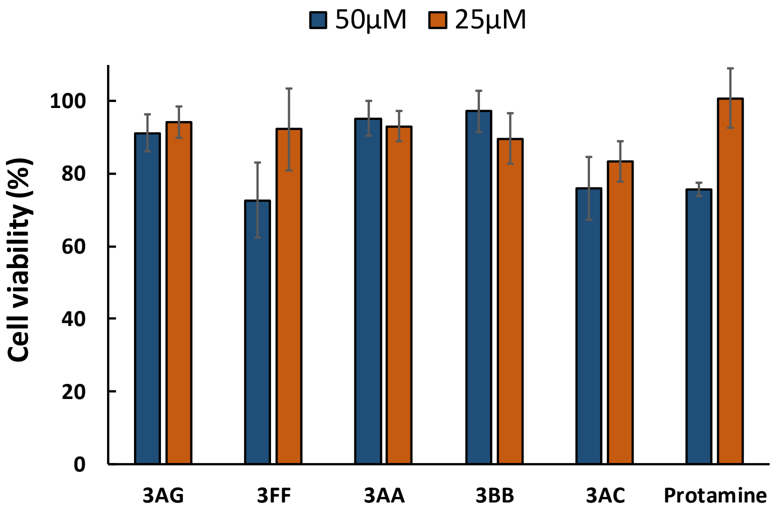

Figure S38. Cell viability plot obtained by MTT assays on A549 cells. The calculated molar concentration for the highest dose used in the *in vivo* experiments was 18 µM.

References:

1. References used for NMR assignment: (a) Šimkovic, I. *et al.* Heparin composition: calculation based on elemental analysis and NMR data. *Chem. Pap.* **74**, 349–355 (2020). (b) Mauri, L. *et al.* Qualification of HSQC methods for quantitative composition of heparin and low molecular weight heparins. *J Pharm Biomed Anal* **136**, 92–105 (2017). (c) Guerrini, M., Naggi, A., Guglieri, S., Santarsiero, R. & Torri, G. Complex glycosaminoglycans: profiling substitution patterns by two-dimensional nuclear magnetic resonance spectroscopy. *Anal Biochem* **337**, 35–47 (2005). (d) Viskov, C. *et al.* Heparin dodecasaccharide containing two antithrombin-binding pentasaccharides: structural features and biological properties. *J Biol Chem* **288**, 25895–25907 (2013). (e) Langeslay, D. J., Beecher, C. N., Dinges, M. M. & Larive, C. K. Glycosaminoglycan structural characterization. *eMagRes* **2**, 205–214 (2013). (f) Guerrini, M., Guglieri, S., Naggi, A., Sasisekharan, R. & Torri, G. Low molecular weight heparins: structural differentiation by bidimensional nuclear magnetic resonance spectroscopy. *Semin Thromb Hemost* **33**, 478–487 (2007). (g) Keire, D. A., Buhse, L. F. & al-Hakim, A. Characterization of currently marketed heparin products: composition analysis by 2D-NMR. *Anal. Methods* **5**, 2984 (2013).
2. (a) Ferro, D. R.; Provasoli, A.; Ragazzi, M.; Torri, G.; Casu, B.; Gatti, G.; Jacquinet, J. C.; Sinay, P.; Petitou, M.; Choay, J., Evidence for conformational equilibrium of the sulfated L-iduronate residue in heparin and in synthetic heparin mono- and oligosaccharides: NMR and force-field studies. *J. Am. Chem. Soc* **1986,** *108*, 6773-6778; (b) Ferro, D. R.; Provasoli, A.; Ragazzi, M.; Casu, B.; Torri, G.; Bossennec, V.; Perly, B.; Sinay, P.; Petitou, M.; Choay, J., Conformer populations of L-iduronic acid residues in glycosaminoglycan sequences. *Carbohydr Res* **1990,** *195* (2), 157-67; (c) Munoz-Garcia, J. C.; Lopez-Prados, J.; Angulo, J.; Diaz-Contreras, I.; Reichardt, N.; de Paz, J. L.; Martin-Lomas, M.; Nieto, P. M., Effect of the substituents of the neighboring ring in the conformational equilibrium of iduronate in heparin-like trisaccharides. *Chemistry* **2012,** *18* (51), 16319-31.
